# A unified approach to model peripheral nerves across different animal species

**DOI:** 10.7717/peerj.4005

**Published:** 2017-11-10

**Authors:** Elisabetta Giannessi, Maria Rita Stornelli, Pier Nicola Sergi

**Affiliations:** 1 Department of Veterinary Science, University of Pisa, Pisa, Italy; 2 Translational Neural Engineering Laboratory, The Biorobotics Institute, Sant’Anna School of Advanced Studies, Pontedera, Italy

**Keywords:** Peripheral nerves, Finite Element Models, Yeoh-like strain energy function, Computational models

## Abstract

Peripheral nerves are extremely complex biological structures. The knowledge of their response to stretch is crucial to better understand physiological and pathological states (e.g., due to overstretch). Since their mechanical response is deterministically related to the nature of the external stimuli, theoretical and computational tools were used to investigate their behaviour. In this work, a Yeoh-like polynomial strain energy function was used to reproduce the response of *in vitro* porcine nerve. Moreover, this approach was applied to different nervous structures coming from different animal species (rabbit, lobster,* Aplysia*) and tested for different amount of stretch (up to extreme ones). Starting from this theoretical background, in silico models of both porcine nerves and cerebro-abdominal connective of *Aplysia* were built to reproduce experimental data (*R*^2^ > 0.9). Finally, bi-dimensional in silico models were provided to reduce computational time of more than 90% with respect to the performances of fully three-dimensional models.

## Introduction

Peripheral nerves are extremely complex biological structures which bridge the central nervous system with the periphery of the body (Sunderland, 1945; Topp & Boyd, 2006). They are able to continuously conduce nervous impulses though axons, which run along the nerve inside a framework of connective tissues (Millesi, Zoch & Reihsner, 1995). Peripheral nerves are sensitive to external perturbations, so unphysiological stretches can result in injuries, which are classified with respect to their degree of damage (Seddon, 1943) or with respect to the ability of axons to regenerate (Sunderland, 1951). Mild stretches can preserve the integrity of connective tissues, but are able to prevent axons from transmitting nervous impulses (neuropraxia), as in the so called “stinger syndrome”, due to the nerve overstretching in arms and legs (Castro, 2003; Greenberg, Leung & Kendall, 2011). Connective tissues are still preserved for further stretches, which result in axonal damage with Wallerian degeneration (axonotmesis), while too large stretches (e.g., due to severe traumas) lead to the nerve rupture with the loss of continuity and a significant distortion of connective tissues (neurotmesis) (Campbell, 2008).

Since the mechanical response of peripheral nerves is deterministically related to the external stimuli (e.g., traction force), anatomical studies (Sunderland, 1945; Sunderland, 1965) were integrated with biomechanical investigations (Millesi, 1986; Millesi, Zoch & Reihsner, 1995). Tissue mechanics was used in combination with finite element analysis (Zienkiewicz, 1977; Cook, 1981; Bathe, 1996; Halloran & Erdemir, 2011; Erdemir et al., 2012) to explore the response of peripheral nerves to different kinds of stimuli, as radial compression (Ju et al., 2006; Main et al., 2011), and intraoperative traction (Ma et al., 2013). The knowledge of the nerve response was, indeed, interesting for several applications, as neuroprosthetics (Navarro et al., 2005; Grill, Norman & Bellamkonda, 2009), neural interfaces design (Sergi et al., 2006; Cutrone et al., 2011) and interaction with biomedical devices (e.g., microneedles (Yoshida et al., 2007; Sergi et al., 2012; Sergi, Jensen & Yoshida, 2016)). However, the modelling of the peripheral nervous tissue (PNT) behaviour is still a challenging task for computational biology. In particular, invariant-based strain energy functions (SEFs) were proposed (Alexander, Barkmeier-Kraemer & Vande Geest, 2010) to model the mild stretch of piglet nerves. In addition, it is not clear whether only one invariant-based SEF could be applied to reproduce large stretches in different animal species and human beings. As a consequence, in this work, a Yeoh-like (Yeoh, 1993) polynomial SEF was applied to different nervous structures belonging to different animal species and for a wide range of stretch (from *λ* = 1.08 (Dilley, Summerhayes & Lynn, 2007; Dilley et al., 2003) to *λ* = 5 (Koike, 1987)).

The logic flow of the text is the following: first, the suitability of the proposed SEF was tested both for a porcine peripheral specimen and for different animal species (lobster, rabbit, *Aplysia*). Then, a three-dimensional in silico model was implemented to reproduce experimental stress–stretch data, while a bidimensional approximation was used to decrease computational times. Similarly, a solid model was created to reproduce the behaviour of a cerebro-abdominal connective of *Aplysia* (Koike, 1987), while a reduced bidimensional approximation was used to speed-up simulations keeping the same amount of information and lowering computational times.

## Materials and Methods

### Stretching experiments

**Figure 1:**
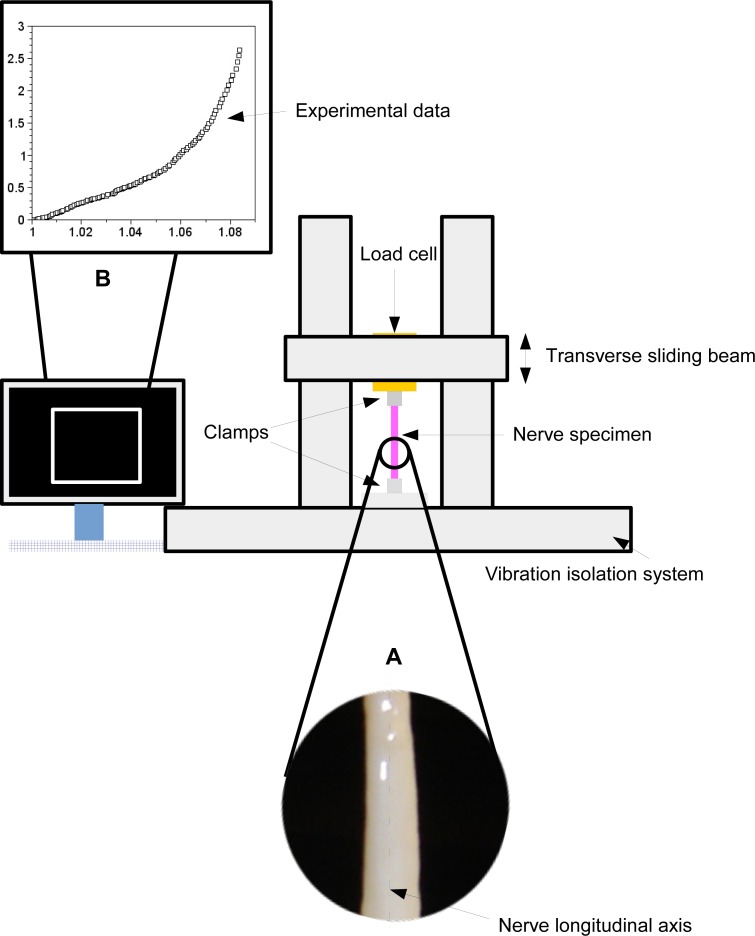
A scheme of the experimental framework used to stretch the nervous specimen (magnification in A). The nerve was fixed between two clamps and stretched through the movement of a transverse sliding beam of a testing material machine (isolated from the environment). A load cell recorded both displacements and forces, which were further elaborated to provide the digital stress/stretch curve (B).

A peroneal nerve was dissected from a posterior limb of a Large White pig (∼10 months old), which was slaughtered in conformity with the Italian National Regulation and frozen until experiments. Before experiments, the nerve specimen (96 mm long with a cross-sectional area of ∼6.39 mm^2^) was gradually defrosted and re-hydrated for about one hour at room temperature in a bath of aqueous saline solution isotonic to the blood (0.9% sodium chloride) to minimize the time dependence of the tissue hydration. The length of the specimen between clamps was 69 mm, and its physiological characteristics were kept by regularly spraying saline moisture on its external surface in order to maintain the initial level of hydration. Stretching experiments were carried out at room temperature (∼25 ± 1 °C), by using an Instron R4464 testing machine (Instron Corporation, Canton, MA, USA) with a standard load cell (Instron load cell, cell type 2525–808, max force 10 N, accuracy 0.25% Full Scale Output; (Instron Corporation, Canton, MA, USA) as shown in Fig. 1.

More specifically, the nerve was stretched (velocity *v* = 10 mm/min (Bora, Richardson & Black, 1980); maximum strain 8%) after preconditioning to minimize viscoelastic effects (Fung, 1993). The axial force was digitally recorded for five extensions of the nerve.

### Theoretical connection between stress and stretch

The peripheral nervous tissue (PNT) was modelled as a homogeneous and incompressible material and, according to previous literature (Alexander, Barkmeier-Kraemer & Vande Geest, 2010), its mechanical behavior was described through an invariant based strain energy function. More specifically, here, a polynomial hyperelastic strain energy function in Yeoh form (Yeoh, 1993) was proposed:

(1) \begin{eqnarray*}\Psi ({I}_{1})={c}_{1}({I}_{1}-3)+{c}_{2}({I}_{1}-3)^{2}+{c}_{3}({I}_{1}-3)^{3}\end{eqnarray*}

where *c*_1_, *c*_2_, *c*_3_ ∈ ℜ were scalar coefficients. The Cauchy stress tensor was expressed in function of both the first strain invariant (*I*_1_) and the deformation gradient **F** as:

(2) \begin{eqnarray*}\sigma =-k\mathbf{I}+2 \frac{\partial \Psi ({I}_{1})}{\partial {I}_{1}} {\mathbf{FF}}^{\mathbf{T}}\end{eqnarray*}

where *k* was an indeterminate Lagrange multiplier, accounting for boundary conditions, while **I** and **F**^**T**^ were, respectively, the unit tensor and the transposed of the deformation gradient. Since *I*_1_ = *tr*(**FF**^**T**^), and **F** were expressed as a function of principal stretches, Eq. (2) provided a theoretical connection between stress and stretch. A homogeneous, triaxial stretch state was assumed, thus the deformation gradient and the first strain invariant were written in function of the longitudinal stretch *λ*. Considering both incompressibility constraint and experimental boundary conditions (through which *k* in Eq. (2) was determined), the theoretical transverse stretch was $1/\sqrt{\lambda }$, while the longitudinal component of the Cauchy stress was expressed as:

(3) \begin{eqnarray*}{\sigma }_{z}= \frac{{\wp }^{[9]}(\lambda ,{c}_{1},{c}_{2},{c}_{3})}{{\lambda }^{3}} \end{eqnarray*}

where ℘^[9]^(*λ*, *c*_1_, *c*_2_, *c*_3_) = 3*c*_3_*λ*^9^ + (2*c*_2_ − 18*c*_3_)*λ*^7^ + 9*c*_3_*λ*^6^ + (27*c*_3_ − 6*c*_2_ + *c*_1_)*λ*^5^ + (2*c*_2_ − 18*c*_3_)*λ*^4^ + (−27*c*_3_ + 6*c*_2_ − *c*_1_)*λ*^2^ + (36*c*_3_ − 4*c*_2_)*λ* − 12*c*_3_.

Experimental data were collected for five consecutive extensions and their mean values were reported as a function of stretch and used to represent the behaviour of the specimen. Equation (3) was used to reproduce experimental data through a non-linear optimization procedure (quasi-Newton algorithm, Scilab, 2015; Scilab Enterprises S.A.S, Versailles, France), allowing the *R*^2^ function to be maximized for each extension. More specifically, guess values for [*c*_1_, *c*_2_] were chosen (i.e., [10.00, 0]; [12.99, 0]; [0, 12.40]; [0, 11.99]; [10.00, 0]), while [*c*_3_], was allowed to vary in the range 6,000–9,000 KPa. To explore the sensitiveness of final vales of *c*_1_ and *c*_2_ to changes of *c*_3_, the difference *c*_1_(*c*_3_) − *c*_2_(*c*_3_) was plotted (for a constant *R*^2^ ≃ 0.99), as shown in Fig. S1. In addition, the influence of *c*_3_ over *R*^2^ was studied for constant values of *c*_1_, *c*_2_, as shown in Fig. S2. Furthermore, the correlation between *c*_1_ and *c*_2_ values was analyzed to test their eventual independency as well as the correlation between these values and the *c*_3_ constant.

Finally, the sensitiveness of the stress function with respect to *c*_1_, *c*_2_, *c*_3_ was expressed as: *SI*(*c*_*i*_) = (*σ*_max_*c*_*i*___ − *σ*_min_*c*_*i*___)∕*σ*_max_*c*_*i*___ (Hamby, 1995; Pannell, 1997), where *i* = 1, 2, 3, *σ*_max_*c*_*i*___ and *σ*_min_*c*_*i*___ were respectively the maximum and the minimum values of stress for the maximum and minimum values of *c*_*i*_, when the other constant had optimal values.

### In silico model of porcine PNT

In silico models were implemented to reproduce the *in vitro* stretching of peripheral nerves. To provide a suitable approximation of the complex shape of the real specimen, two different lateral views were digitally acquired (Fig. S3) and numerically reproduced through ImageJ (Rasband, 1997–2017) together with the mean straight lines for each profile (see Figs. S3A, S3B). The resulting elliptical cylinder (eccentricity 0.77, major axis 3.57 mm and minor axis 2.28 mm) was, then, modelled within a FE software (Ansys ©  Academic; Ansys, Inc., Canonsburg, PA, USA) as an incompressible solid. In particular, the nerve volume was meshed (5,004 nodes and 3,450 elements (Fig. S3)) with solid elements (SOLID185), which were able to model fully incompressible hyperelastic materials with enhanced strain and mixed displacement-pressure formulation (Cescotto & Fonder, 1979; Brink & Stein, 1996). The lower side of the cylinder was fully constrained to account for the lower clamped extremity, while experimental stretches were reproduced increasing the axial displacement of the upper section (further prevented from lateral contractions). Different approaches were also investigated to minimize the time needed to calculate the stress field for the three-dimensional elliptical cylinder. First, exploiting symmetries, only a quarter of the whole volume was meshed (1,330 nodes and 828 elements (Fig. S4A)) with solid elements (SOLID185), while symmetry constraints were imposed to the lateral areas. Then, the mean elliptic cylinder (reproducing the mean surface of the nerve and having a low eccentricity), was rescaled into a circular cylinder, keeping constant the cross sectional area through the additional constraint *r*^2^ = *ab* (where *r* was the radius of the new circular section, while *a*, *b* were the semi-major and semi-minor length of the elliptic section). The axial symmetry of the circular cylinder was furthermore used to study only a rectangular slice (Fig. S4B), which was eshed (210 nodes and 138 elements) with axisymmetric plane elements (PLANE182). These elements were able to model fully incompressible hyperelastic materials using enhanced strain and mixed displacement pressure formulation. Also in this case, to reproduce experimental boundary conditions, the lower line belonging to the rectangular area was fully constrained, while increasing longitudinal displacements were imposed to the upper extremity, which was prevented from radial contractions. The times needed to solve three-dimensional and bidimensional approximations were compared and differences in stress fields and lateral strains were investigated.

### In silico models of cerebro-abdominal connective of *Aplysia*

An in silico model of the right connective of *Aplysia kurodai*, interconnected to the abdominal pleural ganglion, was implemented to test the suitability of the previous procedure in case of different neural structures and for very large stretches. More specifically, the cross section of the cerebro-abdominal connective was reproduced from literature images (Koike, 1987) and approximated with an ellipse (eccentricity 0.64) with semi-major and semi-minor axes respectively of 0.57 mm and 0.44 mm. Similarly, the length of the connective was taken from experimental images (i.e., 50 mm).

The solid model of *Aplysia* connective (Fig. S5A) was meshed with solid elements (SOLID285), which were able to model fully incompressible hyperelastic materials with mixed displacement-pressure formulation. This procedure resulted in 2,086 nodes and 7,192 elements. In this model, the upper side was fully constrained (to account for the experimental clamped extremity), while the stretch was modelled through increasing axial displacements of the lower side, which was prevented from lateral contraction at the level of the middle sphere approximating the ganglion (Fig. S5). Also in this case, the eccentricity of the connective was low, thus the cross sectional area was re-scaled into a circular one with the same size. The resulting axisymmetric circular cylinder was then reduced to a bidimensional slice (Fig. S5B), which was meshed (460 nodes and 173 elements) with plane elements (PLANE183). Indeed, these elements were able to model fully incompressible hyperelastic materials, implementing axial symmetry and large strains with mixed displacement-pressure formulation. Again, the upper side was fully constrained, while the lower one was axially displaced and prevented from radial compressions. The global computatioanl times needed to solve three-dimensional and bidimensional approximations were compared and differences in stress fields and lateral strains were investigated.

## Results

### A single strain energy function to reproduce the nerve behavior across different animal species

**Figure 2:**
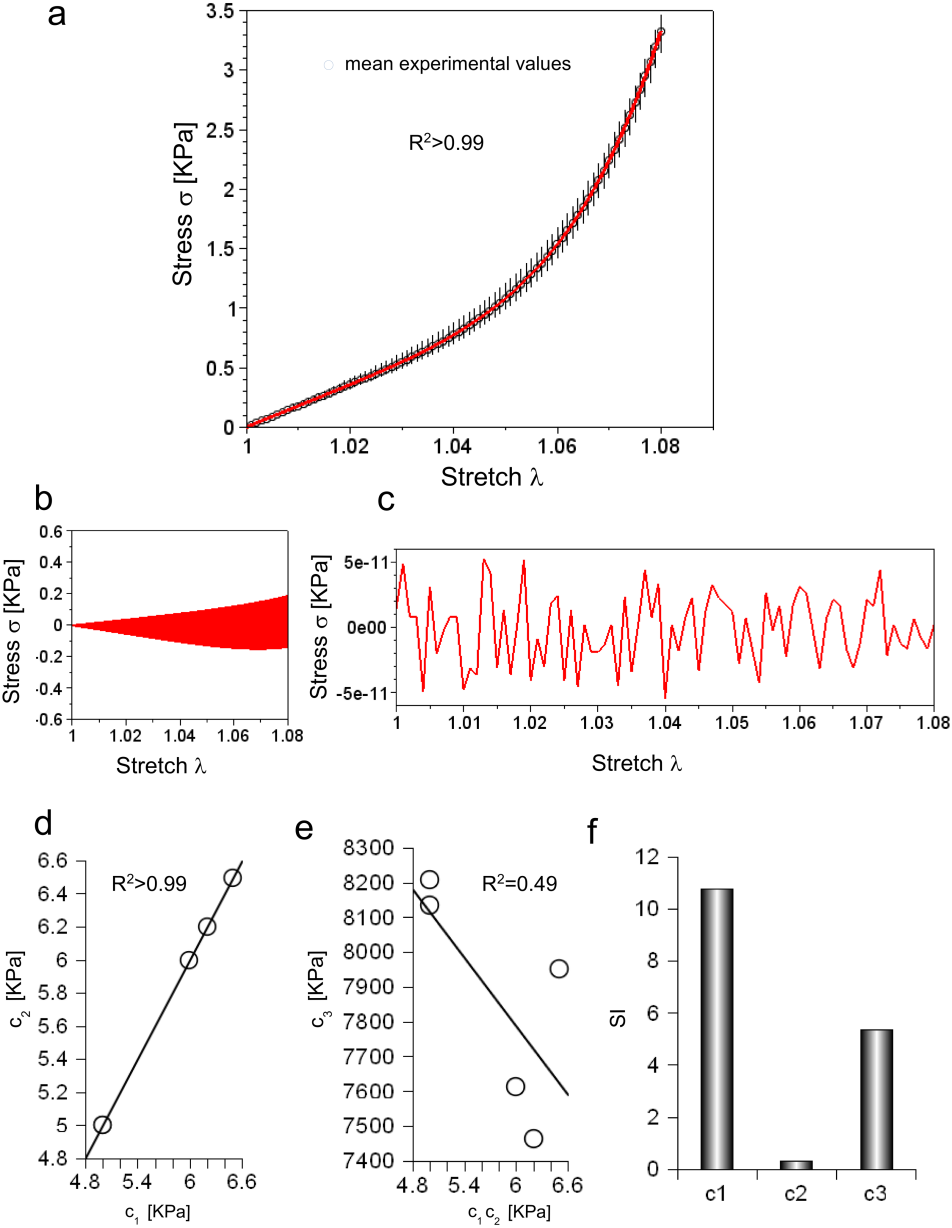
(A) Experimental stress–stretch curve for 5 extensions. The mean values are plotted with circles, while standard deviations are shown through vertical bars. The theoretical curve, reproducing the mean experimental values, is plotted in red. (B) Difference between maximum and minimum values of all cycles as a function of stretch. (C) Difference between theoretical and mean experimental stress as a function of stretch. (D) Values of *c*_1_ and *c*_2_ for each elongation. (E) Values of *c*_3_ for each elongation compared to the corresponding values of *c*_1_ and *c*_2_. (F) Sensitivity index for *c*_1_, *c*_2_, *c*_3_ constants.

Experimental data were collected for five consecutive extensions and their mean values were reported as a function of stretch in Fig. 2A (circles). For each value, the variation range was also plotted as vertical lines (i.e., difference between minimum and maximum values).

Equation (3) was able to reproduce experimental data (*R*^2^ ≃ 0.998) with *c*_1_ = 5.89 KPa, *c*_2_ = 5.89 KPa and *c*_3_ = 7.75 MPa, while both the range of variation of experimental values and the errors between the mean values and the fitting curve were calculated (Figs. 2B and 2C). To further investigate the ability of Eq. (3) in reproducing each single extension, all curves were analysed. In all cases, *c*_1_ and *c*_2_ were almost equal (i.e., error less than 2.422⋅10^−5^ KPa), and had the following values 4.99 KPa , 6.49 KPa, 6.20 KPa, 5.99 KPa, 4.99 KPa, while the *c*_3_ parameter resulted in 8.13 MPa, 7.95 MPa, 7.46 MPa, 7.61 MPa, 8.21 MPa. These values were inserted in Eq. (3), which was, then, able to fit experimental data for each curve (respectively with *R*^2^ of 0.995, 0.995, 0.996, 0.996, 0.995). To test possible further cross correlations among numerical constants in Eq. (3), the *c*_1_ values were plotted versus the corresponding value of *c*_2_ for each extension, while the values of *c*_3_ were also plotted versus *c*_1_, *c*_2_. This procedure showed a positive and very strong correlation in the first case (i.e., *R*^2^ > 0.99, as expected), while a weak correlation in the second one *R*^2^ = 0.49, as shown in Figs. 2D and 2E. Finally, the sensitivity index (SI) for *c*_1_, *c*_2_, *c*_3_ constants resulted respectively in 10.79, 0.32, 5.38 (Fig. 2F).

**Figure 3:**
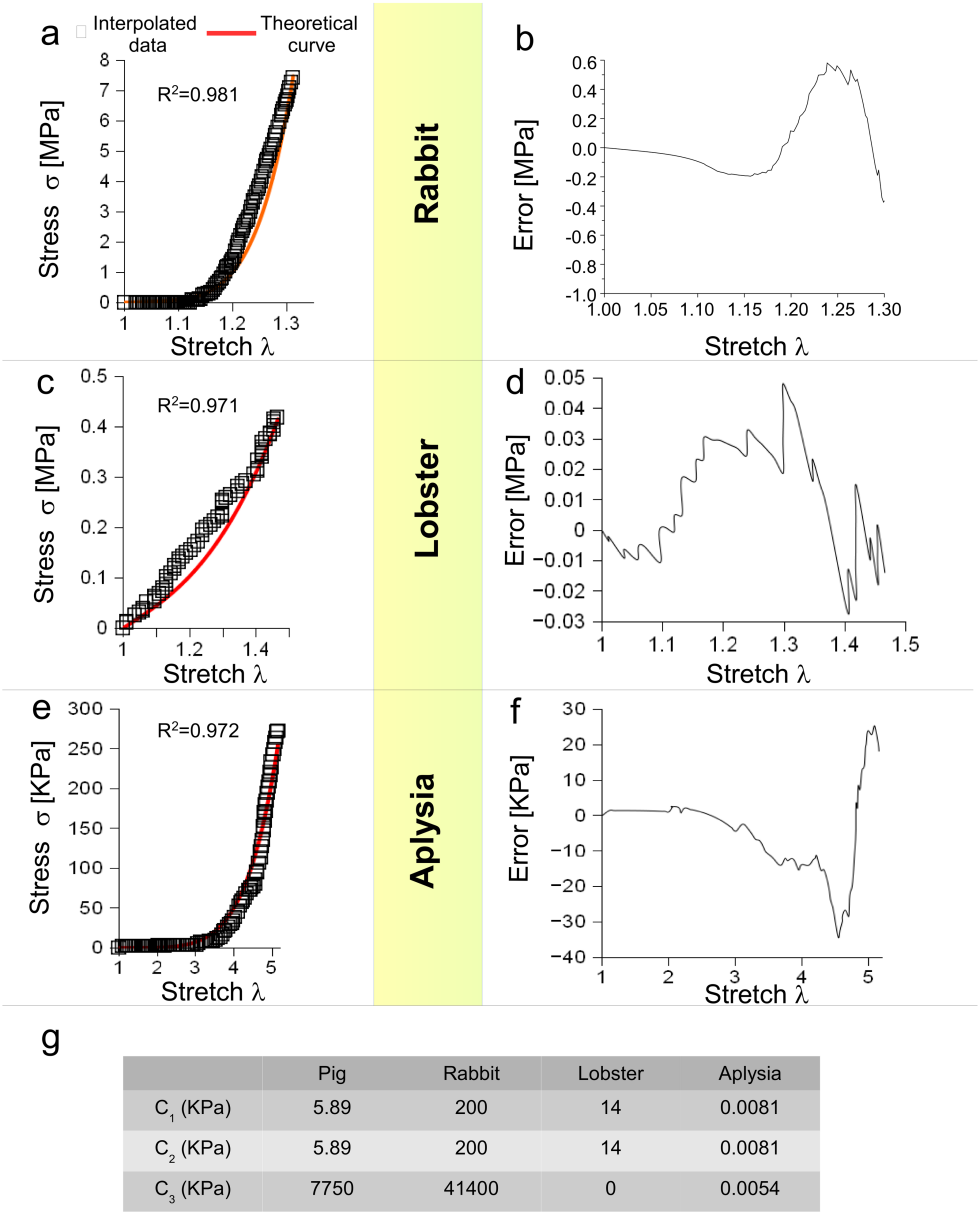
Test of SEF for different animal species. (A) Stress/stretch curve for a nerve of rabbit and theoretical approximation. (B) Error (MPa) between data and approximation for rabbit. (C) Stress/stretch curve for a lobster nerve. (D) Error (MPa) between data and approximation for lobster. (E) Stress/stretch curve for a connective nerve of *Aplysia*. (F) Error (KPa) between data and approximation for *Aplysia*. (G) Values of *c*_1_, *c*_2_, *c*_3_ for different animal species.

Moreover, Eq. (3) was tested for different animal species (vertebrates and invertebrates) and for different levels of stretch. More specifically, the behaviour of a rabbit nerve until *λ* ≃ 1.3 was reproduced (*R*^2^ = 0.981) for *c*_1_, *c*_2_ = 0.2 MPa and *c*_3_ = 43.7 MPa, with errors (between data and theoretical curve) ranging from −0.38 to +0.58 MPa (Figs. 3A and 3B). Similarly, the stress/stretch curve of lobster nerve was approximated (*R*^2^ = 0.971) for *λ* = 1.5 with *c*_1_, *c*_2_ = 0.14 MPa and *c*_3_ = 0, with errors in the range −0.03,  + 0.05 MPa (Figs. 3C and 3D). Finally, the stress/stretch curve of a cerebro-abdominal connective of *Aplysia* (*λ* = 5) was reproduced (*R*^2^ = 0.972 for *c*_1_, *c*_2_ = 0.0081 KPa and *c*_3_ = 0.0054 KPa), with errors ranging between −34.41 and +25.31 KPa (Figs. 3E and 3F). A comparison between coefficients was also performed and shown in Table of Fig. 3G.

### In silico models of PNT

#### Three-dimensional model

**Figure 4:**
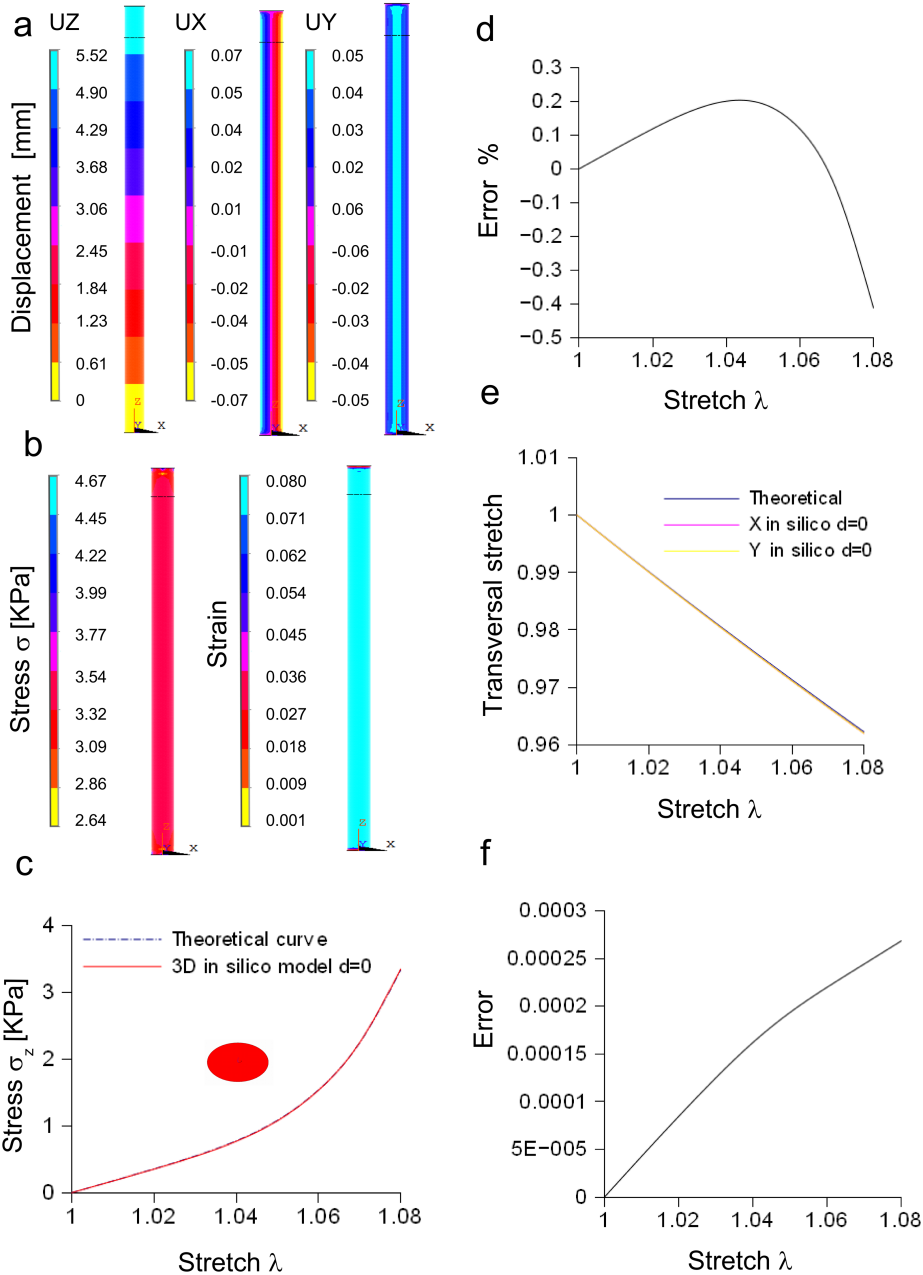
Displacement and stress/stain fields for the three-dimensional elliptic model of nerve. (A) Displacements in *X*, *Y*, *Z* directions at the maximum stretch. (B) Stress and strain fields along the specimen at the maximum stretch. (C) Comparison between theoretical and in silico stress for increasing stretches. (D) Percentage error between theoretical and in silico stress for increasing stretches. (E) Comparison between theoretical and in silico transversal strains for increasing stretches. (F) Percentage errors between theoretical and in silico transversal strains.

In Fig. 4A, the spatial distribution of displacements is shown along the *X*, *Y*, *Z* axes, while in Fig. 4B both the stress and strain fields are shown for the maximum stretch. Theoretical and the computational curves were compared (Fig. 4C), and their difference (within the −0.4%) was plotted as a function of stretch (Fig. 4D). Similarly, theoretical and computational strains along radial directions (*X*, *Y* axes) were compared (Fig. 4E), and their difference (within 0.0003) was plotted as a function of stretch, as shown in Fig. 4F.

Furthermore, the stress/stretch curves resulting from three-dimensional models (elliptic and re-scaled circular cylinders) (Fig. S6A) were compared, and their difference (Fig.  S6B) was within the 0.02%. Similarly, the strain along the radial direction was compared for both approximations (Fig. S6C), showing a maximum difference within −1.92⋅10^−6^ (Fig. S6D). In addition, the distribution of nodal stresses (for the maximum stretch) was compared showing a similar evolution (Fig. S6E) for both approximations as well as similar quantile–quantile plots (uniform distribution). Finally, nodal stresses, deriving from the elliptic cylinder, were plotted versus those coming from the circular approximation (Fig.  S6F). In this case, numerical values were grouped along a straight line and clustered around the value of 3.34 KPa.

#### Bidimensional model of *in vitro* porcine nerve

**Figure 5:**
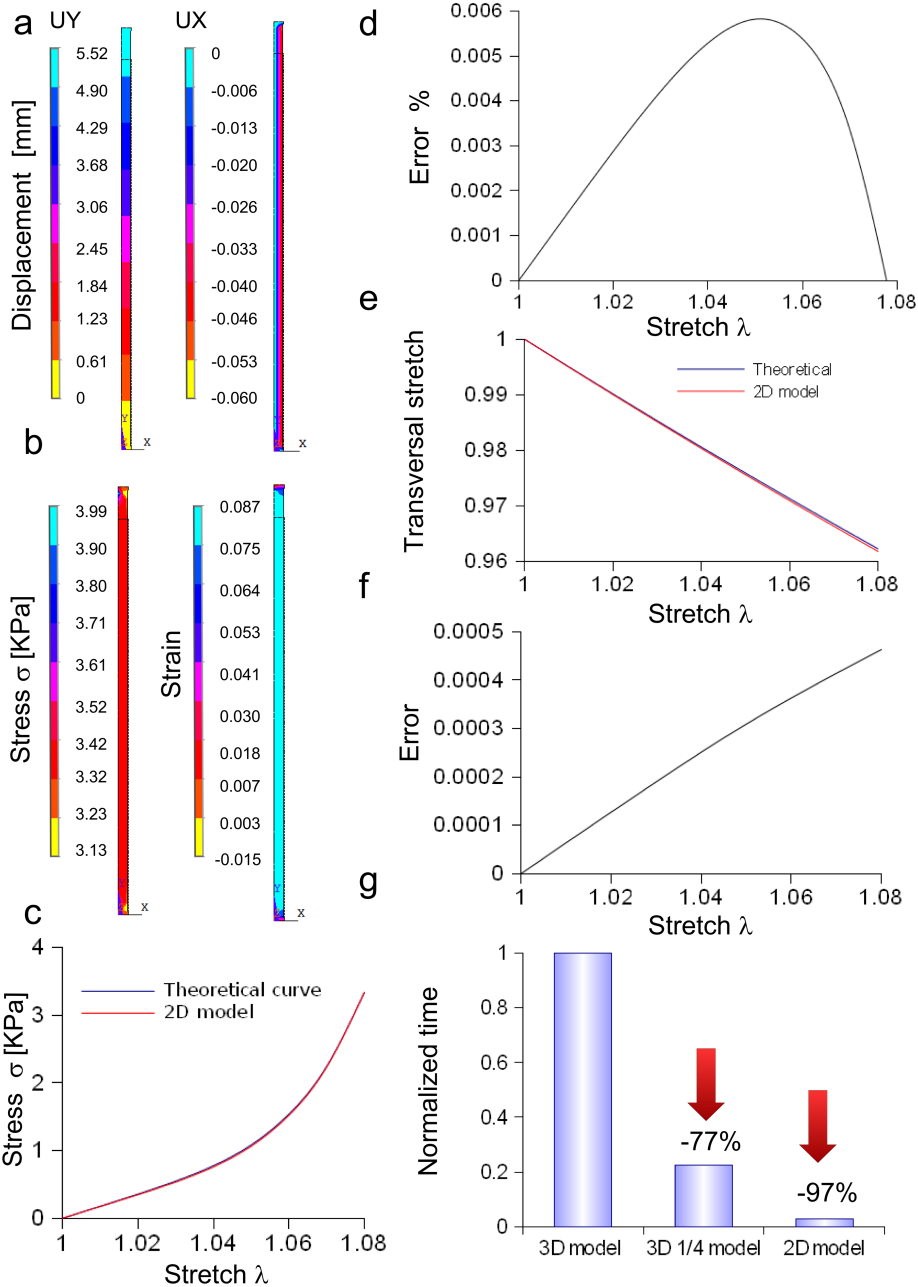
Displacement and stress/stain fields for the bidimensional slice of nerve. (A) Displacements in *X* and *Y* directions for the maximum stretch. (B) Stress and strain fields along the specimen at the maximum stretch. (C) Comparison between theoretical and in silico stress as a function of stretch. (D) Percentage error between theoretical and in silico stress as a function of stretch. (E) Comparison between theoretical and in silico transversal strains as a function of stretch. (F) Percentage errors between theoretical and in silico transversal strains. (G) Percentage decrease in time to solve reduced models (time were normalized over the time needed to solve the full three-dimensional model): the three-dimensional fraction (1∕4 of the whole structure) was able to decrease the time of 77%, while the bidimensional slice further reduced this time of 20%, saving the 97% of the time needed to solve the full solid.

A rectangular slice coming from the axisymmetric re-scaled circular cylinder was used to approximate the nerve specimen (Fig. 5). More specifically, the displacements of the plane section along the *X*, *Y*, *Z* axes as well as the longitudinal stress and strain fields were computed for the maximum stretch (Figs. 5A and 5B). Theoretical and computational stress/stretch curves were compared in Fig. 5C, showing a percentage error within 0.006% (Fig. 5D). Similarly, the comparison between transversal stretches was performed (Fig. 5E), and the error resulted in 0.0005 (Fig. 5F). Finally, the time needed to compute the stress/strain fields was calculated for both the reduced models (i.e., 1∕4 of whole structure and plane axisymmetric slice), and normalized with respect to the total time needed to solve the whole elliptical cylinder. Figure 5G shows that the three-dimensional approximation (1∕4 of the structure) was able to reduce the total normalized time from 1 to 0.225, while the bidimensional one (plane slice) was able to further decrease the normalized time up to 0.029.

#### Three-dimensional model of the *Aplysia* connective

**Figure 6:**
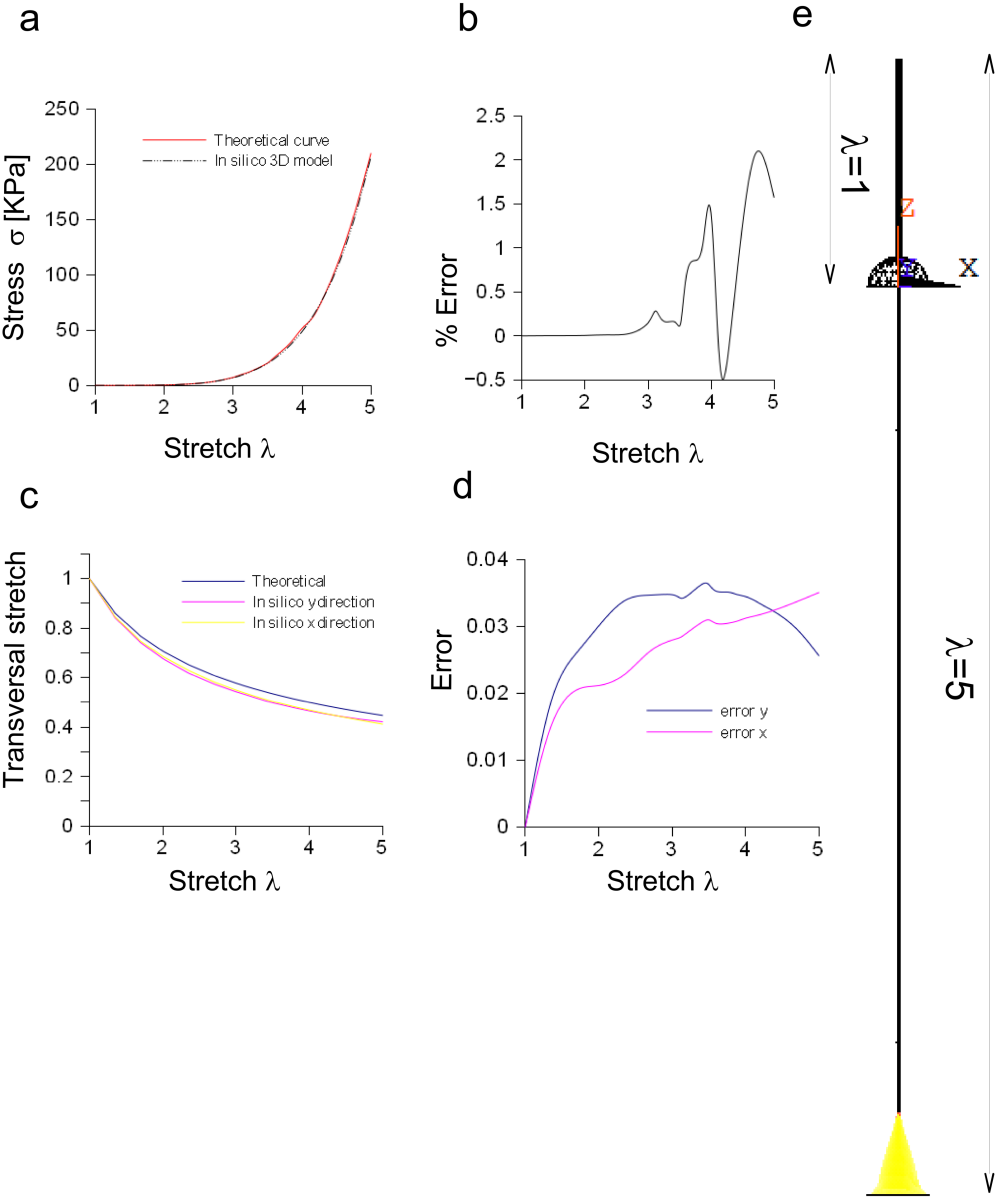
Three-dimensional in silico model of connective of *Aplysia*. (A) Comparison between theoretical and in silico stress/stretch curves. (B) Percentage error between theoretical and in silico curves: the error is zero up to *λ* = 2.5, while the function oscillates in the range *λ* = 2.5–5. Nevertheless, the numerical oscillations were in the range +2.3%,  − 0.5%. (C) Comparison between theoretical and in silico transversal stretch. (D) Difference between theoretical and in silico transversal stretch. Since the in silico data show a dependence on the axis (*X*, *Y*), two different functions are plotted. Nevertheless, in any case, the difference with the theoretical curve is less than 0.04. (E) Deformation of the three-dimensional model of *Aplysia*. The reference configuration *λ* = 1 is plotted in the upper part, while the maximum stretch (for *λ* = 5) is shown in the lower part of the figure.

The performances of the three-dimensional in silico model of the *Aplysia* connective were studied and compared to theoretical results coming from the application of Eq. (3). Both theoretical and computational curves were superimposed (up to *λ* = 5, Fig. 6A), while the percentage difference was between −0.5% and 2.2% (Fig. 6B). Similarly, the transversal stretch deriving from the three-dimensional in silico approximation was compared to the theoretical ones (for both *X* and *Y* axes, Fig. 6C), resulting in differences within 0.039 (Fig.  6D). The deformation of the connective structure is shown in Fig. 6E, where the initial configuration (*λ* = 1) is plotted (upper part, wireframe) together with the final one (*λ* = 5) (lower part).

#### Bidimensional model of the *Aplysia* connective

**Figure 7:**
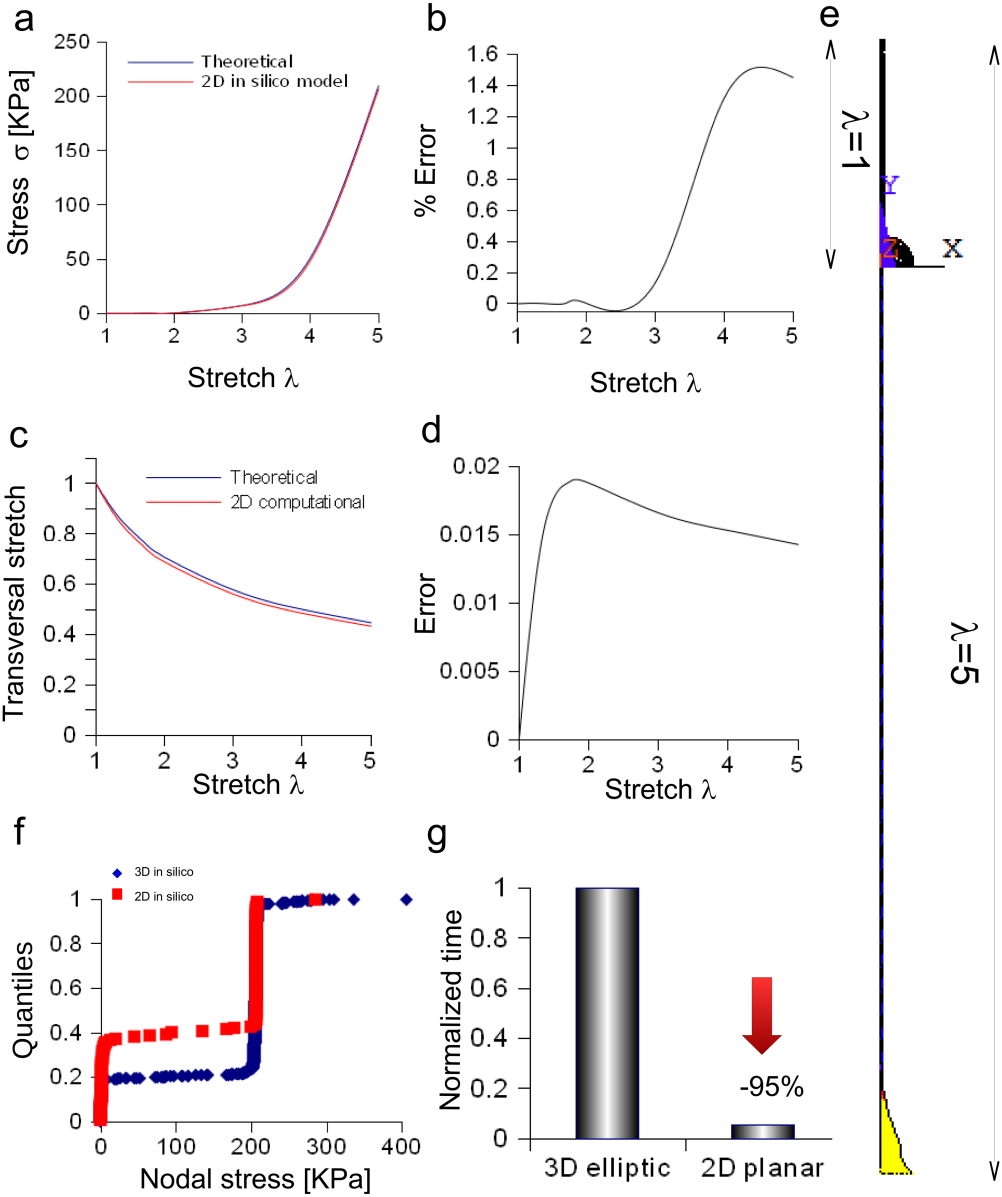
Bidimensional in silico model of connective of *Aplysia*. (A) Comparison between theoretical and in silico stress/stretch curves. (B) Percentage error between theoretical and in silico curves: the error is zero up to *λ* = 1.8, while the function oscillates in the range *λ* = 1.8–5. Nevertheless, the numerical oscillations were lower than 1.6%. (C) Comparison between theoretical and in silico transversal stretch. (D) Difference between theoretical and in silico transversal stretch. Since the in silico data were slightly different and depended on the axis (*X*, *Y*), two different functions are shown. Nevertheless, in any case, the difference is less than 0.04. (E) Deformation of the bidimensional model of *Aplysia*. The reference configuration *λ* = 1 is plotted in the upper part, while the maximum stretch (for *λ* = 5) is shown in the lower part of the figure. (F) Comparison between nodal stress distribution for three-dimensional and bidimensional models (quantile–quantile plot- uniform distribution). The most nodal values correspond to theoretical predictions (about 206.7 KPa) although side effects are present. In other words, the use of the bidimensional slice was equivalent to the use of the three-dimensional structure. (G) Time needed to solve three-dimensional and bidimensional models (normalized over the time needed to solve the fully three-dimensional model): the use of the bidimensional slice allowed a time save of about 95% with very similar results.

The bidimensional approximation of the *Aplysia* connective was able to reproduce the theoretical stress/stretch curve (up to *λ* = 5, Fig. 7A), showing that the percentage error remained within the 1.6% (see also some numerical oscillations in Fig. 7B). The evolution of the theoretical and computational transversal stretches was, then, studied and the resulting two curves were very close along the whole stretch range (Fig. 7C). In particular, the difference between them (within 0.019), was an increasing function up to *λ* = 2, while a slightly decreasing one up to *λ* = 5 (Fig. 7D). The deformation of the connective structure is shown in Fig. 7E, where the initial configuration (*λ* = 1) is plotted (upper part, wireframe), together with the final one (*λ* = 5, lower part). Furthermore, the quantile–quantile plot (uniform distribution) for both three-dimensional and bidimensional models of *Aplysia* connective were provided and compared in Fig. 7F. Although their shape was not totally superimposed, most of values were clustered at 206.7 KPa for both the approximations. Finally, the time needed to solve both three-dimensional and bidimensional models was normalized with respect to the computational time needed to solve the whole three-dimensional structure (Fig. 7G), showing that the use of the axisymmetric slice reduced this time from 1 to 0.056.

## Discussion

### A single strain energy function across different species

In this work, a unified approach was proposed to reproduce the stress/stretch behaviour of neural structures across different animal species (vertebrate/invertebrate) through a classic formulation involving a strain energy function. The chosen function was in Yeoh-like form (Yeoh, 1993), since this formulation was suitable for a wide range of stretches, avoiding numerical instabilities due to a high number of constants (Busfield & Muhr, 2003) and overcoming stretch limitations due to other invariant-based implementations (e.g., Mooney, 1940; Rivlin, 1948). Moreover, it was able to satisfy the Drucker stability criterion (Drucker, 1957; Bergstrom, 2015) (i.e., the resulting stress always increased for increasing strains), allowing boundary value problems to be treated without further numerical complications (e.g., multiple solutions or stress oscillations due to instability). The ability of Eq. (3) in reproducing different responses was due to a certain flexibility, resulting from the interplay between numerical constants. In particular, the values of constants *c*_1_ and *c*_2_ were almost equal for each extension, as well as for different values of the *c*_3_ coefficient. Indeed, a maximum error of 2.422⋅10^−5^ KPa (i.e., 4⋅10^−4^%) was found for all extensions (Fig. S1). Similarly, this difference was 3⋅10^−3^ MPa (i.e., 1.70%) for rabbit, 1.9⋅10^−2^ KPa (i.e., 1.42%) for lobster, and 1⋅10^−7^ KPa (i.e., 1⋅10^−3^%) for *Aplysia*. As a consequence, *c*_1_ and *c*_2_ were assumed to be equal in these animal models. However, the sensitivity of the stress function to *c*_2_ (*SI* = 0.38) was smaller than the sensitivity to *c*_1_ and *c*_3_ (respectively *SI* = 10.79 and *SI* = 5.38). In other words, both *c*_1_ and *c*_3_ seemed to mainly affect the behaviour of the stress function, which was able to reproduce the elastic response of several tissues, ranging from *Aplysia* connective to pig nerves.

Further cross connections between *c*_1_, *c*_2_ and *c*_3_ were investigated (Fig. 2E), but no evidence of a correlation was found (i.e., *R*^2^ = 0.49 and scattered values). Therefore, no important mutual stiffening effects (e.g., due to dehydration) were found during experiments. This was in agreement with the hypothesis of mutual independence between these two constants. As a consequence, Eq. (3) was able to reproduce in a very close way (*R*^2^ > 0.99) experimental data for the large white pig model (both the mean curve and each extension) and for other animal species. However, the percentage errors was around 8–10%, since numerical oscillations arose between theoretical curve and interpolated data.

### In silico models of nerves and *Aplysia* connective

The in silico elliptic cylinder, reproducing the mean surface of the nervous specimen, was able to closely replicate the theoretical behaviour for longitudinal and transversal stretches. Indeed, the percentage error with respect to the theoretical predictions ranged between 0.2% and −0.4% for the axial stretch (Fig. 4D), while the difference between theory and computational results was less than 0.0003 for transversal stretch (Fig. 4F). Moreover, symmetries were used to decrease the computational time needed to find the stress field of this three-dimensional structure. More specifically, both symmetry planes *XZ* and *YZ*, were used to reduce the structure to a quarter of the whole solid. Further reductions (e.g., to 1/8 of the structure) were able to reproduce the stress field (far from extreme sections), while the displacement field was different, since boundary conditions were not symmetric with respect to the *XY* plane. However, just the reduction of the whole structure to a quarter was able to considerably lower the computational time (−77%), as shown in Fig. 5G. A further decrease was achieved through the substitution of the initial elliptic cylinder with a circular one, which was obtained through a re-scaling procedure. Since this cylinder was axisymmetric, the dimensionality of the problem was reduced from three to two dimensions. This approach was based on the equivalence of the two solids, since the eccentricity of the initial cylinder was quite low (0.77). Indeed, not only longitudinal and transversal stretches were almost the same (respectively with percentage errors less than 0.02% for the axial one, and with difference ranging from 0 to −2⋅10^−6^ for the transversal one), but also the nodal stress had the same distribution and the same values, clustered around the theoretical value (Figs. S5E and Figs. S5F). The use of a bidimensional slice instead of the full structure led to computational time decrease of about 97% (Fig. S5G), while it was equivalent for the calculation of both axial and transversal stretches (i.e., percentage errors less than 0.006% and difference less than 0.0005 respectively for axial and transversal directions) (Figs. 5C–5F). The main cause of this decrease of time was the big reduction of nodes and elements, respectively from 5,004 to 210 and from 3,450 to 138, which resulted in the smaller dimension of matrices needed to solve the structure.

Equation (3) was also suitable to model the connective of *Aplysia*. Again, a rescaling procedure and the axial symmetry were used to lower the geometric dimension of the problem together with the time needed to achieve the solution (−95%). The bidimensional approximation provided results similar to those coming from the three-dimensional model. In particular, the percentage error was less than 1.6% and the difference lower than 0.02, respectively for axial and radial stretches. Finally, nodal stresses had a similar distribution in both cases, as shown in Figs. 6 and 7.

### From nerve to bundle mechanics: towards a possible application to the regeneration of nerves

Literature studies on neural-like (Ciofani et al., 2011; Sergi et al., 2013; Sergi, Marino & Ciofani, 2015) or neural cells (Roccasalvo, Micera & Sergi, 2015) investigated the behaviour of single cells in topographical and chemical active environments (Sergi & Cavalcanti-Adam, 2017). Nevertheless, this approach could be effective to study the regeneration of peripheral nerves during the first phases, when axons grow separately. However, when they are grouped into nervous bundles, which are also formed by connective tissues, their global mechanical properties change. Thus, as the mechanical properties of a tissue (which is formed by several thousands of cells) can be mathematically handed using the ”continuum approximation”, in the same way the mechanical behaviour of a nervous bundle can be accounted for through a suitable SEF. The use of Eq. (3) to model peripheral nerves (e.g., pig and rabbit nerves) and the connective of *Aplysia* was not foreseen, since these structures were dissimilar and belonged to different animal species. Indeed, the cerebro-connective was formed by several axons surrounded by a transparent perineurium, so this structure was similar to a nervous bundle of big animal or human nerves (Sunderland & Bradley, 1952). Therefore, the ability of Eq. (3) in reproducing the behaviour of the connective (Koike, 1987) showed that this formulation may be also extended to model the mechanical behaviour of nervous bundles.

### The functional tolerance of nerves to stretch is related to the nerve site

The shape of the stress/stretch curve varied across different species, (e.g., lobster and *Aplysia* curves). Equation (3) was able to account for this variability through the interplay between constants. Nevertheless, the only knowledge of these data was not able to univocally reproduce the response of the material, since this response was related both to the stiffness and to the range of extensibility of nerves (before rupture), which, in its turn, was related to the material toughness. All these characteristics could be resumed as a “functional tolerance” to stretch. Intriguingly, this functional tolerance of nerves to stretch was related to the likelihood of changes in their *in situ* length (Koike, 1987). As a consequence, the rabbit nerves were more extensible than the pig ones, since the rabbit body is, in general, more suitable for running and jumping than the pig body. Similarly, the high (and quite unexpected) resistance of the lobster nerve to extension was related to its very small likelihood of *in situ* extensions, as well as the extreme tolerance to stretch of *Aplysia* connective was related to the high extensibility of its body (Koike, 1987). This hypothesis seems to be supported also by the discovery of the very high stretch tolerance of ventral grooved blubber and tongue nerves in rorqual whales (Balaenopteridae) (Vogl et al., 2015). Indeed, these nerves were able to be easily extended respectively of 75% and 115% before becoming stiff. This very high tolerance to stretch was related to the large deformations of both tongue and ventral grooved blubber needed to implement an efficient feeding. Indeed, other kinds of whale nerves (i.e., intercostal and phrenic nerves) were only able to be normally stretched (12% and 18%) (Vogl et al., 2015). These findings also support the importance of the body location (and the resulting *in situ* stretches) to understand the mechanical response of nerves, since just in a single animal species (rorqual whales) they had a big difference in functional tolerance to stretch.

## Conclusions

In this work, a Yeoh-like SEF was proposed to reproduce the mechanical response of neural structures for a wide range of stretches (from *λ* = 1.08 to *λ* = 5) across different animal species. More specifically, the provided SEF was used to implement in silico FE models of peroneal nerve of large white pig as well as of a cerebro-abdominal connective of *Aplysia*. Symmetries were proposed together with a re-scaling procedure, to simplify in silico models, decreasing the computational time needed to solve stress fields. The provided approach, which was limited to the nerve hypelasticity, could be further enriched accounting for viscous aspects, and used to model the mechanical response of nerves across species and for a wide range of stretches as well as to model the mechanical behaviour of nervous bundles during regeneration processes through suitable scaffolds (Giannessi et al., 2014) and devices (Daly et al., 2011).

##  Supplemental Information

10.7717/peerj.4005/supp-1 Supplemental Information 1 Computational time and stress Raw data.

10.7717/peerj.4005/supp-2 Figure S1 Values of *c*_1_ and *c*_2_ constants as a function of *c*_3_ values On the left side, the evolution of both constants when *c*_3_ varied. On the right side, the evolution of their difference for variations of *c*_3_.

10.7717/peerj.4005/supp-3 Figure S2 Optimization of the *c*_3_ constant values through the optimization of the *R*^2^ value For variations of *c*_3_ the problem has a single solution. In particular, for lobster, the solution is the minimum one maximizing the *R*^2^ value within the allowed range (i.e., *c*_3_ > 0).

10.7717/peerj.4005/supp-4 Figure S3 Different views of the peroneal nerve (A) Global frontal view and extraction of the mean border lines (corresponding to the normalized values = 1) deriving from natural profiles. (B) Global lateral view of specimen and extraction of the mean border lines (corresponding to the normalized values = 1) deriving from natural profiles. (C) Cross section and solid approximation of the nervous specimen built up from the previous mean frontal and lateral border lines. Computational constraints reproduced the experimental boundary conditions.

10.7717/peerj.4005/supp-5 Figure S4 (A) Symmetries in elliptic cylinder. Two planes of symmetry can be used to extract a fraction (1/4) of the whole structure which is representative of the global behaviour. (B) Symmetries in a circular cylinder This cylinder is axisymmetric, thus a single bidimensional slice is representative for the global behaviour of the solid.

10.7717/peerj.4005/supp-6 Figure S5 (A) Scheme of the right connective which interconnected the abdominal and right pleural ganglion as from experimental images in literature (Koike, 1987). Three-dimensional model of the right connective. (B) Rescaled in silico model of the connective: axial symmetry allows a bidimensional slice to be taken as representative for the behaviour of the whole solid

10.7717/peerj.4005/supp-7 Figure S6 From three-dimensional elliptic to three-dimensional circular section (A) Comparison between stress for three-dimensional elliptic and three-dimensional circular approximations when the stretch increased up to *λ* = 1.08. (B) Percentage error between elliptic and circular approximations. (C) Comparison between transversal stretch for elliptic and circular approximations. (D) Percentage error between approximations with the increase of stretch. (E) Quantile-quantile plot of nodal stress for elliptic and circular approximations (uniform distribution). (F) A comparison between nodal stresses (in both approximations) shows a strong correlation together with a clusterization of stress around its mean value.

## Additional Information and Declarations

### Data Availability

The following information was supplied regarding data availability: The raw data has been supplied as a Supplemental File.
