# Supplementary material for: A unified approach to model peripheral nerves across different animal species
Source: PeerJ. 2017 Nov 10;5:e4005. doi: 10.7717/peerj.4005 (PMC5683050; doi:10.7717/peerj.4005)
Supplement: Supplemental Information 1 — Raw data. [file peerj-05-4005-s001.pdf]

Time for simulations (normalized) (porcine nerve)

3D model	1
3D reduced model	0.2256880733945
2D model	0.02935779816514

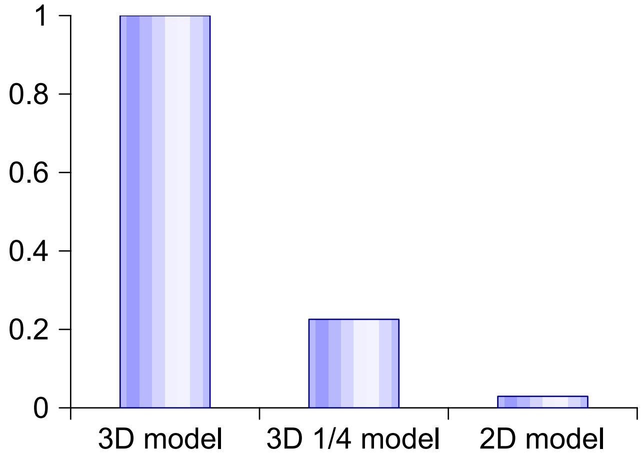

reduction of time for Aplysia

3D elliptic	2D planar
1	0.0562115100051

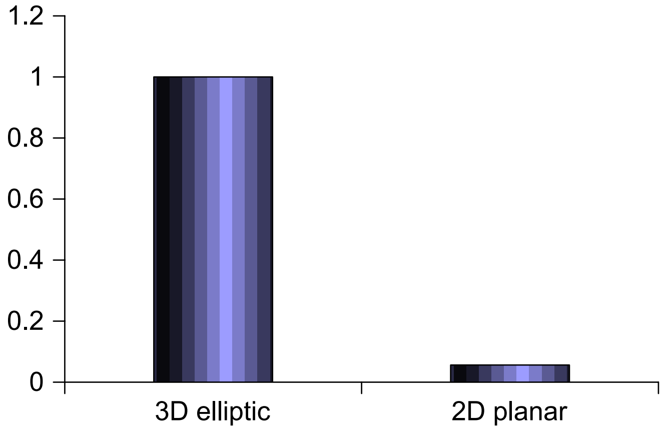

Stress in elliptic section for stretch=1.08

NODE	SX	SY	SZ	SXY	SYZ	SXZ
1	2.7904	2.7745	3.099	1.5078E−010	6.360E−010	−0.70781
2	2.6289	2.6321	2.7574	1.7084E−011	−0.61937	−8.3E−010
3	2.3204	2.2445	2.5891	−0.00035693	−0.14822	−0.68826
4	2.0215	1.9757	2.281	0.016543	−0.31923	−0.49178
5	1.9396	1.9293	2.1942	0.017601	−0.45971	−0.31364
6	2.127	2.142	2.3845	0.0058906	−0.58041	−0.13816
7	2.6289	2.6321	2.7574	1.7359E−011	0.61937	8.31E−010
8	0.026249	−0.2165	2.3334	−2.4414E−010	−0.22348	−2.2E−010
9	0.11256	0.1423	1.7494	6.1696E−012	0.067947	−1.1E−011
10	−0.07944	−0.09547	2.3133	2.927E−011	0.0073217	−7.3E−011
11	0.034082	0.038125	2.2535	−1.555E−011	0.0049274	5.44E−012
12	−0.01951	−0.01491	2.2411	1.3274E−011	−0.0005749	1.95E−011
13	0.0030961	0.006259	2.2447	−5.4858E−013	−0.0001390	1.06E−011
14	−0.00642	−0.00252	2.2306	5.6724E−012	−0.0004762	1.15E−011
15	−0.00100	0.001186	2.23	1.2955E−012	−0.0004526	6.82E−012
16	−0.00254	−0.00047	2.2244	2.47E−012	−0.0002570	5.57E−012
17	−0.00096	0.000252	2.223	9.5786E−013	−0.0002765	3.57E−012
18	−0.00109	−8E−005	2.2207	1.0951E−012	−0.0001317	2.68E−012
19	−0.00055	5.7E−005	2.2198	5.2662E−013	−0.0001397	1.75E−012
20	−0.00049	−1E−005	2.2188	5.0178E−013	−6.6E−005	1.22E−012
21	−0.00028	1.3E−005	2.2183	2.5468E−013	−6.7E−005	8.66E−013
22	−0.00022	1.7E−006	2.2178	2.3468E−013	−3.3E−005	6.66E−013
23	−0.00014	3.0E−006	2.2175	1.3362E−013	−3.1E−005	4.17E−013
24	−0.00010	2.3E−006	2.2173	9.9937E−014	−1.6E−005	3.12E−013
25	−7E−005	7.9E−007	2.2172	7.5925E−014	−1.5E−005	2.03E−013
26	−5E−005	1.4E−006	2.2171	2.8566E−014	−7.9E−006	1.37E−013
27	−3E−005	2.5E−007	2.217	5.5047E−014	−6.8E−006	1.03E−013
28	−2E−005	7.0E−007	2.217	−4.8652E−015	−3.8E−006	1.12E−013
29	−2E−005	1.1E−007	2.217	2.5271E−014	−3.2E−006	1.61E−014
30	−1E−005	3.3E−007	2.217	1.2184E−014	−1.8E−006	5.15E−014
31	−7E−006	5.5E−008	2.2169	3.9835E−014	−1.5E−006	−5.2E−014
32	−5E−006	1.5E−007	2.2169	−3.162E−014	−8.8E−007	−8.0E−014
33	−3E−006	3.0E−008	2.2169	3.685E−015	−6.9E−007	−5.3E−014
34	−2E−006	6.9E−008	2.2169	3.9076E−015	−4.2E−007	5.30E−014
35	−2E−006	1.6E−008	2.2169	9.2251E−015	−3.2E−007	1.06E−013
36	−1E−006	3.1E−008	2.2169	1.0242E−014	−2.0E−007	1.97E−014
37	−8E−007	8.6E−009	2.2169	9.94E−015	−1.5E−007	−1.4E−013
38	−5E−007	1.4E−008	2.2169	−1.1219E−015	−9.6E−008	8.47E−014
39	−4E−007	4.4E−009	2.2169	−3.0398E−014	−7.1E−008	7.93E−014
40	−2E−007	6.4E−009	2.2169	1.9725E−014	−4.6E−008	8.44E−014
41	−2E−007	2.3E−009	2.2169	−6.4112E−015	−3.3E−008	8.28E−014
42	−1E−007	2.9E−009	2.2169	6.231E−015	−2.2E−008	9.07E−014
43	−8E−008	1.1E−009	2.2169	1.3005E−014	−1.6E−008	−9.8E−014

44	−6E−008	1.3E−009	2.2169	1.4839E−014	−1.0E−008	−1.6E−013
45	−4E−008	5.7E−010	2.2169	−1.3448E−014	−7.4E−009	−3.1E−014
46	−3E−008	6.1E−010	2.2169	9.8072E−015	−4.9E−009	1.25E−013
47	−2E−008	2.8E−010	2.2169	−2.6293E−014	−3.5E−009	1.00E−013
48	−1E−008	2.8E−010	2.2169	−3.3565E−014	−2.3E−009	−2.3E−013
49	−9E−009	1.4E−010	2.2169	4.9564E−014	−1.6E−009	−1.8E−013
50	−6E−009	1.3E−010	2.2169	−3.4413E−014	−1.1E−009	−2.8E−013
51	−4E−009	6.6E−011	2.2169	4.1064E−014	−7.7E−010	−2.7E−013
52	−3E−009	6.0E−011	2.2169	1.7799E−014	−5.2E−010	−1.9E−013
53	−2E−009	3.2E−011	2.2169	1.7265E−014	−3.6E−010	−1.0E−013
54	−1E−009	2.8E−011	2.2169	2.3517E−015	−2.5E−010	−7.0E−014
55	−9E−010	1.5E−011	2.2169	1.6306E−014	−1.7E−010	−1.2E−013
56	−6E−010	1.3E−011	2.2169	1.9299E−014	−1.2E−010	4.74E−013
57	−4E−010	6.8E−012	2.2169	1.5806E−014	−8.1E−011	3.37E−013
58	−3E−010	5.6E−012	2.2169	−7.6016E−014	−5.6E−011	1.49E−013
59	−2E−010	4.1E−012	2.2169	7.0369E−014	−3.8E−011	2.79E−014
60	−1E−010	2.2E−012	2.2169	−4.6165E−014	−2.7E−011	6.89E−014
61	−1E−010	2.1E−012	2.2169	2.1347E−014	−1.7E−011	3.01E−013
62	−7E−011	7.7E−013	2.2169	−9.8329E−014	−1.3E−011	9.83E−014
63	−5E−011	3.2E−013	2.2169	−2.5392E−014	−9.0E−012	1.43E−013
64	−3E−011	9.0E−013	2.2169	1.3241E−015	−5.4E−012	1.50E−013
65	−2E−011	8.8E−014	2.2169	−4.772E−015	−3.7E−012	−2.9E−013
66	−1E−011	1.1E−012	2.2169	−6.4892E−015	−2.6E−012	−2.6E−014
67	−1E−011	3.5E−013	2.2169	−3.7139E−016	−9.0E−013	−8.1E−014
68	−7E−012	1.2E−013	2.2169	8.1196E−014	−1.9E−012	−1.6E−013
69	−5E−012	3.8E−013	2.2169	−4.4165E−014	4.569E−013	−1.0E−013
70	−4E−012	−7E−013	2.2169	2.1254E−014	−1.9E−012	2.25E−013
71	−2E−012	7.3E−013	2.2169	5.2601E−015	−1.2E−012	1.33E−013
72	−2E−012	−1E−012	2.2169	−4.9808E−014	−3.5E−013	2.37E−013
73	−1E−012	4.5E−013	2.2169	3.8796E−014	−5.7E−013	−3.6E−014
74	−8E−013	−2E−013	2.2169	−1.3583E−014	1.011E−012	1.78E−013
75	−8E−013	−3E−013	2.2169	−2.8113E−014	−1.5E−012	8.84E−014
76	−4E−013	1.2E−012	2.2169	1.4263E−014	1.044E−012	−5.0E−014
77	−7E−013	−8E−013	2.2169	5.2489E−014	−6.1E−013	1.10E−013
78	−1E−013	9.8E−013	2.2169	3.0086E−014	−3.2E−013	−4.8E−014
79	−2E−012	−1E−012	2.2169	−3.4873E−014	−4.5E−013	1.26E−013
80	−2E−012	−5E−013	2.2169	6.9963E−014	−8.2E−013	2.69E−013
81	−3E−012	2.1E−013	2.2169	−2.4434E−014	3.828E−013	6.68E−013
82	−4E−012	−5E−013	2.2169	5.3879E−014	1.659E−012	−3.2E−014
83	−4E−012	5.2E−013	2.2169	−9.9082E−014	1.060E−012	1.01E−013
84	−7E−012	3.9E−015	2.2169	4.3251E−014	9.258E−013	−4.1E−013
85	−1E−011	4.1E−013	2.2169	−4.0374E−014	2.194E−012	−2.9E−013
86	−1E−011	5.9E−013	2.2169	8.2995E−014	2.596E−012	1.23E−013
87	−2E−011	5.5E−013	2.2169	2.0365E−014	3.795E−012	8.04E−013
88	−3E−011	2.7E−014	2.2169	−7.7063E−014	6.462E−012	5.29E−013
89	−5E−011	5.5E−013	2.2169	−1.4964E−015	8.024E−012	2.59E−013
90	−7E−011	1.3E−012	2.2169	−5.4E−014	1.351E−011	−2.3E−013

91	−1E−010	8.2E−013	2.2169	1.9457E−015	1.756E−011	−2.5E−013
92	−1E−010	3.1E−012	2.2169	2.2114E−014	2.410E−011	5.75E−014
93	−2E−010	3.7E−012	2.2169	1.1173E−014	3.764E−011	2.40E−013
94	−3E−010	6.0E−012	2.2169	−6.4885E−014	5.665E−011	6.09E−014
95	−4E−010	7.1E−012	2.2169	−3.4567E−014	8.121E−011	−7.8E−013
96	−6E−010	1.2E−011	2.2169	2.9587E−014	1.164E−010	−4.2E−014
97	−9E−010	1.5E−011	2.2169	5.095E−015	1.714E−010	2.80E−013
98	−1E−009	2.7E−011	2.2169	−1.6718E−014	2.470E−010	−6.5E−014
99	−2E−009	3.3E−011	2.2169	4.5116E−014	3.653E−010	1.86E−013
100	−3E−009	6.0E−011	2.2169	−1.0877E−014	5.195E−010	−5.3E−014
101	−4E−009	6.7E−011	2.2169	5.4265E−014	7.755E−010	8.10E−015
102	−6E−009	1.3E−010	2.2169	−8.1105E−014	1.096E−009	2.31E−013
103	−9E−009	1.4E−010	2.2169	4.0012E−014	1.643E−009	3.52E−014
104	−1E−008	2.8E−010	2.2169	5.919E−014	2.313E−009	5.96E−013
105	−2E−008	2.8E−010	2.2169	−6.3171E−014	3.482E−009	1.31E−013
106	−3E−008	6.1E−010	2.2169	6.5149E−014	4.875E−009	−2.6E−013
107	−4E−008	5.7E−010	2.2169	3.9459E−014	7.392E−009	1.53E−014
108	−6E−008	1.3E−009	2.2169	−1.1949E−014	1.028E−008	6.60E−013
109	−8E−008	1.1E−009	2.2169	−6.8268E−014	1.570E−008	5.66E−013
110	−1E−007	2.9E−009	2.2169	8.3859E−014	2.164E−008	1.28E−014
111	−2E−007	2.3E−009	2.2169	−9.6497E−014	3.335E−008	−1.3E−013
112	−2E−007	6.4E−009	2.2169	8.9462E−014	4.555E−008	−9.0E−013
113	−4E−007	4.4E−009	2.2169	−7.0413E−014	7.094E−008	−4.8E−013
114	−5E−007	1.4E−008	2.2169	−2.626E−014	9.574E−008	−3.3E−013
115	−8E−007	8.6E−009	2.2169	1.2554E−013	1.510E−007	−5.0E−013
116	−1E−006	3.1E−008	2.2169	−3.4343E−014	2.010E−007	−1.3E−013
117	−2E−006	1.6E−008	2.2169	−8.2061E−014	3.22E−007	2.43E−013
118	−2E−006	6.9E−008	2.2169	1.3319E−013	4.212E−007	3.45E−013
119	−3E−006	3.0E−008	2.2169	8.1198E−015	6.875E−007	3.48E−014
120	−5E−006	1.5E−007	2.2169	−5.7076E−014	8.806E−007	−1.6E−014
121	−7E−006	5.5E−008	2.2169	8.6079E−014	1.470E−006	1.50E−013
122	−1E−005	3.3E−007	2.217	−4.4062E−014	1.836E−006	4.42E−013
123	−2E−005	1.1E−007	2.217	6.109E−015	3.150E−006	−4.4E−013
124	−2E−005	7.0E−007	2.217	1.0914E−013	3.811E−006	−2.7E−013
125	−3E−005	2.5E−007	2.217	1.1006E−014	6.763E−006	9.04E−015
126	−5E−005	1.4E−006	2.2171	1.8702E−013	7.874E−006	6.10E−013
127	−7E−005	7.9E−007	2.2172	5.5077E−014	1.454E−005	4.55E−013
128	−0.00010	2.3E−006	2.2173	7.5896E−014	1.616E−005	−8.8E−013
129	−0.00014	3.0E−006	2.2176	2.1742E−013	3.122E−005	−1.7E−013
130	−0.00022	1.7E−006	2.2178	3.1143E−013	3.289E−005	−5.8E−013
131	−0.00028	1.3E−005	2.2183	2.7785E−013	6.667E−005	−1.9E−013
132	−0.00049	−1E−005	2.2188	5.1754E−013	6.624E−005	−8.6E−013
133	−0.00055	5.7E−005	2.2198	4.8901E−013	0.00013968	−1.6E−012
134	−0.00109	−8E−005	2.2207	1.1476E−012	0.00013167	−2.5E−012
135	−0.00096	0.000252	2.223	9.501E−013	0.00027651	−3.1E−012
136	−0.00254	−0.00047	2.2244	2.451E−012	0.00025696	−5.0E−012
137	−0.00100	0.001186	2.23	1.3421E−012	0.00045262	−6.7E−012

138	−0.00642	−0.00252	2.2306	5.5733E−012	0.00047619	−1.1E−011
139	0.0030961	0.006259	2.2447	−6.2897E−013	0.00013904	−1.1E−011
140	−0.01951	−0.01491	2.2411	1.3382E−011	0.00057488	−2.0E−011
141	0.034082	0.038125	2.2535	−1.5648E−011	−0.0049274	−6.1E−012
142	−0.07944	−0.09547	2.3133	2.9333E−011	−0.0073217	7.22E−011
143	0.11256	0.1423	1.7494	6.2243E−012	−0.067947	1.18E−011
144	0.026249	−0.2165	2.3334	−2.4415E−010	0.22348	2.18E−010
145	2.7904	2.7745	3.099	1.5078E−010	−6.4E−010	0.70781
146	2.3204	2.2445	2.5891	−0.00035693	0.14822	0.68826
147	2.0215	1.9757	2.281	0.016543	0.31923	0.49178
148	1.9396	1.9293	2.1942	0.017601	0.45971	0.31364
149	2.127	2.142	2.3845	0.0058906	0.58041	0.13816
150	−0.15411	−0.09376	2.6371	6.5387E−010	−1.0E−010	−0.37212
151	0.15176	0.10327	1.8677	−1.27E−010	−3.6E−010	0.011648
152	−0.05310	−0.06027	2.0314	−4.0163E−011	1.730E−011	−0.000667
153	0.020061	0.014108	2.1064	−2.1271E−011	6.609E−011	0.0025652
154	−0.01015	−0.01171	2.1343	−1.2151E−011	5.250E−011	0.0034728
155	0.0033302	0.002659	2.1668	−3.2372E−012	3.722E−011	0.0025098
156	−0.00254	−0.00284	2.1815	−3.5241E−012	2.496E−011	0.0021758
157	0.0006067	0.000603	2.1948	−5.5337E−013	1.681E−011	0.001317
158	−0.00070	−0.00075	2.2015	−1.0535E−012	1.082E−011	0.0010461
159	8.0E−005	0.000132	2.2069	−6.9682E−014	7.290E−012	0.00062683
160	−0.00021	−0.00021	2.21	−3.2514E−013	4.697E−012	0.00047967
161	−1E−005	2.2E−005	2.2123	−6.9668E−015	3.247E−012	0.00029316
162	−7E−005	−6E−005	2.2137	−1.0138E−013	2.140E−012	0.00021969
163	−2E−005	−5E−007	2.2148	−1.6195E−014	1.494E−012	0.00013741
164	−2E−005	−2E−005	2.2154	−3.1513E−014	9.488E−013	0.00010144
165	−1E−005	−3E−006	2.2159	−3.7722E−015	6.851E−013	6.47E−005
166	−1E−005	−7E−006	2.2162	−2.038E−014	4.386E−013	4.72E−005
167	−6E−006	−2E−006	2.2164	2.2109E−014	3.349E−013	3.06E−005
168	−4E−006	−3E−006	2.2166	−3.0264E−014	1.718E−013	2.21E−005
169	−3E−006	−1E−006	2.2167	−4.7364E−015	1.505E−013	1.45E−005
170	−2E−006	−1E−006	2.2167	−2.8294E−015	1.363E−013	1.04E−005
171	−1E−006	−6E−007	2.2168	6.535E−015	1.401E−013	6.85E−006
172	−9E−007	−5E−007	2.2168	−7.3296E−015	1.082E−013	4.87E−006
173	−7E−007	−3E−007	2.2169	−1.6238E−014	1.835E−014	3.25E−006
174	−4E−007	−2E−007	2.2169	3.0113E−015	−1.2E−014	2.30E−006
175	−3E−007	−2E−007	2.2169	−1.8319E−014	−1.0E−013	1.54E−006
176	−2E−007	−1E−007	2.2169	3.6014E−014	1.484E−013	1.08E−006
177	−2E−007	−7E−008	2.2169	−3.8874E−015	9.365E−014	7.28E−007
178	−9E−008	−5E−008	2.2169	7.7273E−015	6.394E−014	5.10E−007
179	−7E−008	−3E−008	2.2169	1.3351E−014	2.951E−014	3.44E−007
180	−4E−008	−2E−008	2.2169	−5.2488E−015	−1.3E−013	2.41E−007
181	−3E−008	−2E−008	2.2169	1.7534E−014	−1.5E−013	1.63E−007
182	−2E−008	−1E−008	2.2169	2.6928E−014	−2.6E−015	1.14E−007
183	−2E−008	−8E−009	2.2169	−1.4132E−014	5.580E−014	7.71E−008
184	−1E−008	−5E−009	2.2169	1.1242E−014	6.028E−014	5.37E−008

185	−7E−009	−4E−009	2.2169	−1.0731E−014	1.726E−013	3.65E−008
186	−5E−009	−3E−009	2.2169	−5.2935E−014	−3.0E−014	2.53E−008
187	−3E−009	−2E−009	2.2169	2.5977E−015	1.695E−014	1.73E−008
188	−2E−009	−1E−009	2.2169	−4.7028E−015	1.899E−014	1.20E−008
189	−2E−009	−8E−010	2.2169	1.9198E−014	4.056E−014	8.17E−009
190	−1E−009	−6E−010	2.2169	−2.2892E−014	1.700E−013	5.66E−009
191	−8E−010	−4E−010	2.2169	7.1552E−015	8.189E−014	3.86E−009
192	−5E−010	−3E−010	2.2169	−3.8691E−014	−3.3E−014	2.67E−009
193	−4E−010	−2E−010	2.2169	1.9865E−014	−1.2E−013	1.83E−009
194	−2E−010	−1E−010	2.2169	2.0882E−015	−6.6E−014	1.26E−009
195	−2E−010	−9E−011	2.2169	−3.2413E−014	1.904E−014	8.65E−010
196	−1E−010	−6E−011	2.2169	1.9154E−017	−1.5E−013	5.97E−010
197	−8E−011	−4E−011	2.2169	5.3044E−014	−1.3E−013	4.09E−010
198	−5E−011	−3E−011	2.2169	−7.2972E−015	1.428E−013	2.82E−010
199	−4E−011	−2E−011	2.2169	1.9386E−015	1.374E−013	1.94E−010
200	−3E−011	−1E−011	2.2169	2.9409E−014	2.895E−013	1.33E−010
201	−2E−011	−9E−012	2.2169	−6.7668E−014	2.682E−014	9.18E−011
202	−1E−011	−7E−012	2.2169	4.7793E−014	2.357E−013	6.30E−011
203	−8E−012	−4E−012	2.2169	−7.5192E−014	−6.3E−014	4.29E−011
204	−6E−012	−3E−012	2.2169	9.791E−014	−1.6E−013	2.98E−011
205	−5E−012	−3E−012	2.2169	−6.8267E−015	4.257E−014	2.00E−011
206	−3E−012	−2E−012	2.2169	−4.8553E−015	−7.2E−014	1.42E−011
207	−2E−012	−9E−013	2.2169	8.2086E−014	5.731E−014	1.02E−011
208	−1E−012	−7E−013	2.2169	−4.9687E−014	1.797E−013	6.58E−012
209	−7E−013	−3E−013	2.2169	−1.2774E−014	1.675E−014	4.57E−012
210	−1E−012	−2E−013	2.2169	−5.7363E−014	−1.2E−013	3.18E−012
211	−3E−013	−3E−013	2.2169	1.6661E−014	5.052E−014	2.30E−012
212	−7E−013	−3E−013	2.2169	−4.6843E−014	−1.1E−013	1.25E−012
213	−2E−013	−6E−013	2.2169	5.7629E−014	4.850E−014	1.94E−012
214	−7E−013	−6E−013	2.2169	4.219E−014	1.240E−013	8.63E−013
215	−2E−013	−5E−013	2.2169	4.2721E−014	1.798E−013	−7.5E−013
216	2.6E−013	4.7E−014	2.2169	−1.4944E−014	4.786E−013	1.01E−012
217	−5E−013	−2E−013	2.2169	−5.4532E−015	3.654E−013	−4.9E−013
218	−5E−013	−8E−013	2.2169	−4.6427E−014	1.531E−013	−9.2E−014
219	−5E−013	−4E−013	2.2169	−4.3714E−014	4.007E−014	−4.2E−013
220	1.0E−013	−3E−013	2.2169	7.4274E−014	1.064E−013	−4.5E−013
221	−5E−013	−5E−013	2.2169	1.1816E−014	1.896E−013	−9E−013
222	3.0E−014	6.6E−014	2.2169	1.0084E−014	2.592E−013	−5.0E−013
223	−5E−013	−6E−013	2.2169	−1.8365E−014	2.331E−013	−8.1E−013
224	−1E−013	9.2E−015	2.2169	−1.1067E−014	1.138E−013	−1.4E−012
225	−4E−013	−5E−013	2.2169	−4.5138E−014	2.975E−013	−1.8E−012
226	−1E−012	−9E−013	2.2169	−2.366E−014	−2.8E−013	−3.9E−012
227	−7E−013	−4E−013	2.2169	9.4427E−014	6.266E−013	−4.9E−012
228	−2E−012	−9E−013	2.2169	−1.3887E−014	1.795E−013	−5.4E−012
229	−2E−012	−4E−013	2.2169	−8.2714E−014	2.113E−013	−9.8E−012
230	−2E−012	−8E−013	2.2169	3.6934E−014	3.692E−013	−1.3E−011
231	−4E−012	−2E−012	2.2169	−6.5986E−014	−2.8E−013	−2.2E−011

232	−6E−012	−3E−012	2.2169	6.6107E−014	−3.2E−014	−3.0E−011
233	−9E−012	−5E−012	2.2169	−7.9521E−014	−1.3E−013	−4.3E−011
234	−1E−011	−6E−012	2.2169	−3.3975E−014	−2.1E−013	−6.3E−011
235	−2E−011	−9E−012	2.2169	−1.9715E−014	−3.7E−013	−9.1E−011
236	−3E−011	−1E−011	2.2169	7.533E−014	−1.9E−013	−1.3E−010
237	−4E−011	−2E−011	2.2169	−1.016E−014	5.216E−014	−1.9E−010
238	−5E−011	−3E−011	2.2169	−3.8628E−014	1.288E−013	−2.8E−010
239	−8E−011	−4E−011	2.2169	−4.1801E−014	−4.8E−013	−4.1E−010
240	−1E−010	−6E−011	2.2169	5.5808E−016	−2.8E−013	−6.0E−010
241	−2E−010	−9E−011	2.2169	1.8731E−014	−6.4E−013	−8.7E−010
242	−2E−010	−1E−010	2.2169	−3.0076E−015	−1.6E−013	−1.3E−009
243	−4E−010	−2E−010	2.2169	−2.1228E−014	−5.4E−013	−1.8E−009
244	−5E−010	−3E−010	2.2169	3.892E−014	−4.3E−014	−2.7E−009
245	−8E−010	−4E−010	2.2169	−1.5713E−014	−7.2E−015	−3.9E−009
246	−1E−009	−6E−010	2.2169	−8.7857E−014	2.851E−013	−5.7E−009
247	−2E−009	−8E−010	2.2169	5.7652E−014	3.009E−013	−8.2E−009
248	−2E−009	−1E−009	2.2169	6.0119E−015	2.457E−013	−1.2E−008
249	−3E−009	−2E−009	2.2169	7.5119E−014	6.019E−013	−1.7E−008
250	−5E−009	−3E−009	2.2169	−6.6218E−014	2.703E−013	−2.5E−008
251	−7E−009	−4E−009	2.2169	9.2787E−015	−4.0E−013	−3.6E−008
252	−1E−008	−5E−009	2.2169	5.0126E−014	1.234E−013	−5.4E−008
253	−2E−008	−8E−009	2.2169	−2.5555E−014	1.551E−013	−7.7E−008
254	−2E−008	−1E−008	2.2169	1.562E−014	−1.2E−013	−1.1E−007
255	−3E−008	−2E−008	2.2169	−6.2815E−015	7.502E−014	−1.6E−007
256	−4E−008	−2E−008	2.2169	−8.2874E−014	−4.7E−014	−2.4E−007
257	−7E−008	−3E−008	2.2169	1.4365E−014	7.350E−014	−3.4E−007
258	−9E−008	−5E−008	2.2169	−1.0334E−013	−1.8E−013	−5.1E−007
259	−2E−007	−7E−008	2.2169	−1.2458E−014	−2.7E−013	−7.3E−007
260	−2E−007	−1E−007	2.2169	−2.9278E−014	−9.1E−014	−1.1E−006
261	−3E−007	−2E−007	2.2169	−6.9074E−014	2.082E−013	−1.5E−006
262	−4E−007	−2E−007	2.2169	−3.4995E−014	−4.0E−014	−2.3E−006
263	−7E−007	−3E−007	2.2169	1.294E−013	1.016E−013	−3.2E−006
264	−9E−007	−5E−007	2.2168	1.0364E−013	2.506E−013	−4.9E−006
265	−1E−006	−6E−007	2.2168	−1.8154E−013	−1.0E−013	−6.9E−006
266	−2E−006	−1E−006	2.2167	2.2485E−013	−3.7E−014	−1.0E−005
267	−3E−006	−1E−006	2.2167	−1.8768E−013	−3.5E−013	−1.4E−005
268	−4E−006	−3E−006	2.2166	−1.9423E−014	−3.1E−013	−2.2E−005
269	−6E−006	−2E−006	2.2164	1.8808E−013	−1.4E−013	−3.1E−005
270	−1E−005	−7E−006	2.2162	−1.0788E−013	−5.5E−013	−4.7E−005
271	−1E−005	−3E−006	2.2159	−4.4542E−014	−1.4E−013	−6.5E−005
272	−2E−005	−2E−005	2.2154	−1.0215E−013	−1.0E−012	−0.000101
273	−2E−005	−5E−007	2.2148	1.612E−013	−1.2E−012	−0.000137
274	−7E−005	−6E−005	2.2137	−2.8227E−013	−1.9E−012	−0.000220
275	−1E−005	2.2E−005	2.2123	1.3229E−013	−2.3E−012	−0.000293
276	−0.00021	−0.00021	2.21	−3.8174E−013	−4.3E−012	−0.000480
277	8.0E−005	0.000132	2.2069	−8.6882E−014	−7.6E−012	−0.000627
278	−0.00070	−0.00075	2.2015	−9.9401E−013	−1.1E−011	−0.001046

279	0.0006067	0.000603	2.1948	−4.9299E−013	−1.6E−011	−0.001317
280	−0.00254	−0.00284	2.1815	−3.4391E−012	−2.5E−011	−0.002176
281	0.0033302	0.002659	2.1668	−3.3981E−012	−3.7E−011	−0.002510
282	−0.01015	−0.01171	2.1343	−1.2076E−011	−5.3E−011	−0.003473
283	0.020061	0.014108	2.1064	−2.1321E−011	−6.6E−011	−0.002565
284	−0.05310	−0.06027	2.0314	−4.0182E−011	−1.7E−011	0.00066743
285	0.15176	0.10327	1.8677	−1.2699E−010	3.604E−010	−0.011648
286	−0.15411	−0.09376	2.6371	6.5391E−010	1.008E−010	0.37212
287	0.030674	−0.01169	2.3885	−0.0163	−0.079372	−0.36935
288	0.094931	0.050735	2.0936	0.00918	0.019493	−0.007808
289	−0.02320	−0.03345	2.0938	0.0017863	0.0098895	0.0031496
290	0.01118	0.005256	2.1276	0.00074121	0.0016888	0.0053743
291	−0.00450	−0.00571	2.1536	0.00040646	0.00021518	0.0061701
292	0.0011773	0.000688	2.1732	0.00023895	−0.0008768	0.0046044
293	−0.00146	−0.00135	2.1872	0.000133	−0.0007022	0.003553
294	−2E−005	0.000109	2.1967	8.2683E−005	−0.0006737	0.0023974
295	−0.00055	−0.00038	2.2032	4.625E−005	−0.0004681	0.0017006
296	−0.00012	3.2E−006	2.2076	3.0979E−005	−0.0003539	0.0011385
297	−0.00022	−0.00012	2.2106	1.7394E−005	−0.0002425	0.00078686
298	−8E−005	−1E−005	2.2126	1.2454E−005	−0.0001709	0.00053129
299	−9E−005	−4E−005	2.214	7.1095E−006	−0.0001177	0.00036436
300	−4E−005	−1E−005	2.2149	5.3103E−006	−8.1E−005	0.00024821
301	−4E−005	−2E−005	2.2155	3.1017E−006	−5.6E−005	0.00016991
302	−2E−005	−6E−006	2.216	2.3583E−006	−3.8E−005	0.00011645
303	−2E−005	−6E−006	2.2163	1.41E−006	−2.7E−005	7.97E−005
304	−1E−005	−3E−006	2.2165	1.0737E−006	−1.8E−005	5.48E−005
305	−8E−006	−3E−006	2.2166	6.5557E−007	−1.3E−005	3.75E−005
306	−5E−006	−1E−006	2.2167	4.9583E−007	−8.5E−006	2.59E−005
307	−4E−006	−1E−006	2.2168	3.0829E−007	−5.9E−006	1.77E−005
308	−2E−006	−7E−007	2.2168	2.3084E−007	−4.0E−006	1.22E−005
309	−2E−006	−5E−007	2.2168	1.4576E−007	−2.8E−006	8.37E−006
310	−1E−006	−3E−007	2.2169	1.0799E−007	−1.9E−006	5.77E−006
311	−8E−007	−3E−007	2.2169	6.9082E−008	−1.3E−006	3.95E−006
312	−5E−007	−2E−007	2.2169	5.0664E−008	−9.1E−007	2.73E−006
313	−4E−007	−1E−007	2.2169	3.277E−008	−6.3E−007	1.87E−006
314	−3E−007	−8E−008	2.2169	2.3815E−008	−4.3E−007	1.29E−006
315	−2E−007	−5E−008	2.2169	1.5548E−008	−3.0E−007	8.84E−007
316	−1E−007	−4E−008	2.2169	1.121E−008	−2.0E−007	6.09E−007
317	−8E−008	−3E−008	2.2169	7.3762E−009	−1.4E−007	4.18E−007
318	−6E−008	−2E−008	2.2169	5.2813E−009	−9.6E−008	2.88E−007
319	−4E−008	−1E−008	2.2169	3.4985E−009	−6.7E−008	1.98E−007
320	−3E−008	−8E−009	2.2169	2.49E−009	−4.6E−008	1.36E−007
321	−2E−008	−6E−009	2.2169	1.6588E−009	−3.1E−008	9.35E−008
322	−1E−008	−4E−009	2.2169	1.1747E−009	−2.2E−008	6.44E−008
323	−9E−009	−3E−009	2.2169	7.8627E−010	−1.5E−008	4.42E−008
324	−6E−009	−2E−009	2.2169	5.5438E−010	−1.0E−008	3.04E−008
325	−4E−009	−1E−009	2.2169	3.7264E−010	−7.0E−009	2.09E−008

326	−3E−009	−9E−010	2.2169	2.6174E−010	−4.8E−009	1.44E−008
327	−2E−009	−6E−010	2.2169	1.7651E−010	−3.3E−009	9.89E−009
328	−1E−009	−4E−010	2.2169	1.2365E−010	−2.3E−009	6.81E−009
329	−9E−010	−3E−010	2.2169	8.3569E−011	−1.6E−009	4.68E−009
330	−6E−010	−2E−010	2.2169	5.8336E−011	−1.1E−009	3.22E−009
331	−4E−010	−1E−010	2.2169	3.9621E−011	−7.5E−010	2.21E−009
332	−3E−010	−9E−011	2.2169	2.7565E−011	−5.1E−010	1.52E−009
333	−2E−010	−6E−011	2.2169	1.8795E−011	−3.5E−010	1.05E−009
334	−1E−010	−4E−011	2.2169	1.3114E−011	−2.4E−010	7.20E−010
335	−1E−010	−3E−011	2.2169	8.9052E−012	−1.7E−010	4.95E−010
336	−7E−011	−2E−011	2.2169	6.2151E−012	−1.1E−010	3.40E−010
337	−5E−011	−1E−011	2.2169	4.201E−012	−7.9E−011	2.34E−010
338	−3E−011	−1E−011	2.2169	2.8924E−012	−5.4E−011	1.61E−010
339	−2E−011	−7E−012	2.2169	1.9678E−012	−3.8E−011	1.11E−010
340	−2E−011	−5E−012	2.2169	1.3381E−012	−2.6E−011	7.63E−011
341	−1E−011	−3E−012	2.2169	9.4292E−013	−1.8E−011	5.22E−011
342	−7E−012	−2E−012	2.2169	6.8414E−013	−1.2E−011	3.60E−011
343	−5E−012	−2E−012	2.2169	5.5382E−013	−8.3E−012	2.46E−011
344	−3E−012	−1E−012	2.2169	2.8839E−013	−5.7E−012	1.68E−011
345	−3E−012	−7E−013	2.2169	2.2723E−013	−4.0E−012	1.19E−011
346	−1E−012	−3E−013	2.2169	1.3503E−013	−2.8E−012	8.08E−012
347	−1E−012	−4E−013	2.2169	5.9929E−014	−1.9E−012	5.77E−012
348	−9E−013	−3E−013	2.2169	1.2501E−014	−1.3E−012	3.75E−012
349	−5E−013	−3E−013	2.2169	1.4151E−013	−7.3E−013	2.79E−012
350	−5E−013	−2E−013	2.2169	2.0546E−014	−5.9E−013	1.80E−012
351	−2E−013	−1E−013	2.2169	7.1257E−014	−5.7E−013	8.58E−013
352	1.3E−014	5.4E−014	2.2169	2.7894E−014	−2.3E−013	1.10E−012
353	−5E−013	−3E−013	2.2169	8.1575E−014	−3.8E−013	1.79E−013
354	−3E−013	−1E−013	2.2169	1.076E−014	1.508E−013	1.01E−013
355	−6E−015	1.3E−013	2.2169	−5.0191E−014	−8.1E−014	6.43E−014
356	−8E−014	−6E−014	2.2169	5.5389E−014	1.995E−014	−1.5E−013
357	−7E−014	8.5E−014	2.2169	7.0395E−014	2.437E−013	9.29E−016
358	−2E−013	−2E−013	2.2169	6.3006E−014	1.930E−013	−6.7E−013
359	−1E−013	7.9E−014	2.2169	6.5182E−015	3.099E−013	−1.1E−012
360	−4E−013	−2E−013	2.2169	−1.0487E−014	3.787E−013	−2.1E−012
361	−2E−013	9.4E−014	2.2169	1.4282E−013	6.279E−013	−2.8E−012
362	−8E−013	−2E−013	2.2169	7.8284E−014	1.157E−012	−4.1E−012
363	−1E−012	−5E−013	2.2169	1.1252E−013	1.756E−012	−5.6E−012
364	−2E−012	−5E−013	2.2169	1.9112E−013	2.765E−012	−8.1E−012
365	−3E−012	−8E−013	2.2169	2.3595E−013	3.804E−012	−1.1E−011
366	−4E−012	−1E−012	2.2169	1.5876E−013	5.883E−012	−1.7E−011
367	−5E−012	−1E−012	2.2169	3.6158E−013	8.071E−012	−2.5E−011
368	−7E−012	−2E−012	2.2169	6.1516E−013	1.223E−011	−3.6E−011
369	−1E−011	−3E−012	2.2169	9.569E−013	1.748E−011	−5.3E−011
370	−2E−011	−5E−012	2.2169	1.3484E−012	2.566E−011	−7.6E−011
371	−2E−011	−7E−012	2.2169	1.9717E−012	3.733E−011	−1.1E−010
372	−3E−011	−1E−011	2.2169	3.0195E−012	5.418E−011	−1.6E−010

373	−5E−011	−1E−011	2.2169	4.2384E−012	7.907E−011	−2.3E−010
374	−7E−011	−2E−011	2.2169	6.1728E−012	1.150E−010	−3.4E−010
375	−1E−010	−3E−011	2.2169	8.808E−012	1.670E−010	−5.0E−010
376	−1E−010	−4E−011	2.2169	1.3024E−011	2.426E−010	−7.2E−010
377	−2E−010	−6E−011	2.2169	1.8714E−011	3.529E−010	−1.0E−009
378	−3E−010	−9E−011	2.2169	2.7576E−011	5.124E−010	−1.5E−009
379	−4E−010	−1E−010	2.2169	3.9572E−011	7.461E−010	−2.2E−009
380	−6E−010	−2E−010	2.2169	5.8386E−011	1.083E−009	−3.2E−009
381	−9E−010	−3E−010	2.2169	8.3635E−011	1.576E−009	−4.7E−009
382	−1E−009	−4E−010	2.2169	1.2362E−010	2.288E−009	−6.8E−009
383	−2E−009	−6E−010	2.2169	1.7647E−010	3.333E−009	−9.9E−009
384	−3E−009	−9E−010	2.2169	2.6172E−010	4.833E−009	−1.4E−008
385	−4E−009	−1E−009	2.2169	3.7261E−010	7.045E−009	−2.1E−008
386	−6E−009	−2E−009	2.2169	5.544E−010	1.021E−008	−3.0E−008
387	−9E−009	−3E−009	2.2169	7.8633E−010	1.489E−008	−4.4E−008
388	−1E−008	−4E−009	2.2169	1.1746E−009	2.157E−008	−6.4E−008
389	−2E−008	−6E−009	2.2169	1.6588E−009	3.148E−008	−9.3E−008
390	−3E−008	−8E−009	2.2169	2.49E−009	4.557E−008	−1.4E−007
391	−4E−008	−1E−008	2.2169	3.4984E−009	6.653E−008	−2.0E−007
392	−6E−008	−2E−008	2.2169	5.2813E−009	9.625E−008	−2.9E−007
393	−8E−008	−3E−008	2.2169	7.3762E−009	1.406E−007	−4.2E−007
394	−1E−007	−4E−008	2.2169	1.121E−008	2.033E−007	−6.1E−007
395	−2E−007	−5E−008	2.2169	1.5548E−008	2.972E−007	−8.8E−007
396	−3E−007	−8E−008	2.2169	2.3815E−008	4.292E−007	−1.3E−006
397	−4E−007	−1E−007	2.2169	3.277E−008	6.282E−007	−1.9E−006
398	−5E−007	−2E−007	2.2169	5.0664E−008	9.063E−007	−2.7E−006
399	−8E−007	−3E−007	2.2169	6.9082E−008	1.328E−006	−4.0E−006
400	−1E−006	−3E−007	2.2169	1.0799E−007	1.913E−006	−5.8E−006
401	−2E−006	−5E−007	2.2168	1.4576E−007	2.807E−006	−8.4E−006
402	−2E−006	−7E−007	2.2168	2.3084E−007	4.039E−006	−1.2E−005
403	−4E−006	−1E−006	2.2168	3.0829E−007	5.932E−006	−1.8E−005
404	−5E−006	−1E−006	2.2167	4.9583E−007	8.531E−006	−2.6E−005
405	−8E−006	−3E−006	2.2166	6.5557E−007	1.254E−005	−3.8E−005
406	−1E−005	−3E−006	2.2165	1.0737E−006	1.803E−005	−5.5E−005
407	−2E−005	−6E−006	2.2163	1.41E−006	2.651E−005	−8.0E−005
408	−2E−005	−6E−006	2.216	2.3583E−006	3.817E−005	−0.000116
409	−4E−005	−2E−005	2.2155	3.1017E−006	5.599E−005	−0.000170
410	−4E−005	−1E−005	2.2149	5.3103E−006	8.089E−005	−0.000248
411	−9E−005	−4E−005	2.214	7.1095E−006	0.00011766	−0.000364
412	−8E−005	−1E−005	2.2126	1.2454E−005	0.00017094	−0.000531
413	−0.00022	−0.00012	2.2106	1.7394E−005	0.00024254	−0.000787
414	−0.00012	3.2E−006	2.2076	3.0979E−005	0.00035394	−0.001139
415	−0.00055	−0.00038	2.2032	4.625E−005	0.00046811	−0.001701
416	−2E−005	0.000109	2.1967	8.2683E−005	0.00067373	−0.002397
417	−0.00146	−0.00135	2.1872	0.000133	0.00070216	−0.003553
418	0.0011773	0.000688	2.1732	0.00023895	0.00087678	−0.004604
419	−0.00450	−0.00571	2.1536	0.00040646	−0.0002152	−0.006170

420	0.01118	0.005256	2.1276	0.00074121	−0.0016888	−0.005374
421	−0.02320	−0.03345	2.0938	0.0017863	−0.0098895	−0.003150
422	0.094931	0.050735	2.0936	0.00918	−0.019493	0.007808
423	0.030674	−0.01169	2.3885	−0.0163	0.079372	0.36935
424	0.21345	0.081784	2.2513	−0.011031	−0.13622	−0.29124
425	0.060004	0.015744	2.0938	0.010971	0.049702	−0.032701
426	−0.01477	−0.03114	2.135	0.0029355	0.01726	0.0022618
427	0.0087819	0.004722	2.1721	0.0015101	0.0043947	0.008668
428	−0.00527	−0.00576	2.184	0.00088875	0.00039957	0.0091478
429	0.0005965	0.001551	2.197	0.00070191	−0.0012801	0.0073186
430	−0.00218	−0.00128	2.2022	0.00040918	−0.001229	0.0055141
431	−0.00037	0.000511	2.2074	0.00030054	−0.0011464	0.0039202
432	−0.00090	−0.00031	2.21	0.00017987	−0.0008189	0.002763
433	−0.00030	0.000164	2.2123	0.0001281	−0.0006232	0.0019061
434	−0.00038	−7E−005	2.2136	7.9421E−005	−0.0004259	0.0013197
435	−0.00017	5.3E−005	2.2147	5.582E−005	−0.0003057	0.00090401
436	−0.00016	−2E−005	2.2154	3.5786E−005	−0.0002076	0.00062293
437	−9E−005	1.7E−005	2.2158	2.4969E−005	−0.0001458	0.00042652
438	−7E−005	−3E−006	2.2162	1.643E−005	−9.9E−005	0.0002936
439	−4E−005	6.1E−006	2.2164	1.1404E−005	−6.9E−005	0.00020122
440	−3E−005	−2E−007	2.2166	7.6398E−006	−4.7E−005	0.00013849
441	−2E−005	2.3E−006	2.2167	5.2819E−006	−3.3E−005	9.50E−005
442	−2E−005	2.8E−007	2.2167	3.5795E−006	−2.2E−005	6.54E−005
443	−1E−005	9.0E−007	2.2168	2.4675E−006	−1.5E−005	4.49E−005
444	−7E−006	2.4E−007	2.2168	1.6842E−006	−1.1E−005	3.09E−005
445	−5E−006	3.8E−007	2.2169	1.1587E−006	−7.3E−006	2.12E−005
446	−3E−006	1.5E−007	2.2169	7.9419E−007	−5.0E−006	1.46E−005
447	−2E−006	1.6E−007	2.2169	5.4571E−007	−3.5E−006	1.00E−005
448	−2E−006	7.8E−008	2.2169	3.7496E−007	−2.4E−006	6.90E−006
449	−1E−006	7.4E−008	2.2169	2.5746E−007	−1.6E−006	4.74E−006
450	−7E−007	4E−008	2.2169	1.7714E−007	−1.1E−006	3.26E−006
451	−5E−007	3.4E−008	2.2169	1.2159E−007	−7.8E−007	2.24E−006
452	−4E−007	2.0E−008	2.2169	8.3717E−008	−5.3E−007	1.54E−006
453	−2E−007	1.6E−008	2.2169	5.7458E−008	−3.7E−007	1.06E−006
454	−2E−007	9.6E−009	2.2169	3.9573E−008	−2.5E−007	7.30E−007
455	−1E−007	7.3E−009	2.2169	2.7162E−008	−1.7E−007	5.02E−007
456	−8E−008	4.6E−009	2.2169	1.8709E−008	−1.2E−007	3.45E−007
457	−5E−008	3.4E−009	2.2169	1.2843E−008	−8.2E−008	2.37E−007
458	−4E−008	2.2E−009	2.2169	8.8456E−009	−5.6E−008	1.63E−007
459	−3E−008	1.6E−009	2.2169	6.0732E−009	−3.9E−008	1.12E−007
460	−2E−008	1.1E−009	2.2169	4.1825E−009	−2.7E−008	7.72E−008
461	−1E−008	7.6E−010	2.2169	2.8722E−009	−1.8E−008	5.31E−008
462	−8E−009	5.1E−010	2.2169	1.9777E−009	−1.3E−008	3.65E−008
463	−6E−009	3.6E−010	2.2169	1.3584E−009	−8.7E−009	2.51E−008
464	−4E−009	2.4E−010	2.2169	9.3524E−010	−6.0E−009	1.73E−008
465	−3E−009	1.7E−010	2.2169	6.4246E−010	−4.1E−009	1.19E−008
466	−2E−009	1.1E−010	2.2169	4.4224E−010	−2.8E−009	8.17E−009

467	−1E−009	8.0E−011	2.2169	3.0383E−010	−1.9E−009	5.62E−009
468	−9E−010	5.4E−011	2.2169	2.0918E−010	−1.3E−009	3.86E−009
469	−6E−010	3.8E−011	2.2169	1.4371E−010	−9.2E−010	2.66E−009
470	−4E−010	2.6E−011	2.2169	9.9011E−011	−6.3E−010	1.83E−009
471	−3E−010	1.8E−011	2.2169	6.7989E−011	−4.4E−010	1.26E−009
472	−2E−010	1.2E−011	2.2169	4.6848E−011	−3.0E−010	8.64E−010
473	−1E−010	8.4E−012	2.2169	3.2207E−011	−2.1E−010	5.94E−010
474	−9E−011	5.6E−012	2.2169	2.2156E−011	−1.4E−010	4.09E−010
475	−6E−011	3.8E−012	2.2169	1.5184E−011	−9.7E−011	2.81E−010
476	−4E−011	2.6E−012	2.2169	1.0408E−011	−6.7E−011	1.93E−010
477	−3E−011	1.8E−012	2.2169	7.2201E−012	−4.6E−011	1.33E−010
478	−2E−011	1.1E−012	2.2169	4.9698E−012	−3.2E−011	9.12E−011
479	−1E−011	8.7E−013	2.2169	3.4963E−012	−2.2E−011	6.28E−011
480	−1E−011	5.4E−013	2.2169	2.3782E−012	−1.5E−011	4.32E−011
481	−7E−012	1.4E−013	2.2169	1.5965E−012	−1.0E−011	2.98E−011
482	−5E−012	8.2E−014	2.2169	1.1386E−012	−6.9E−012	2.04E−011
483	−3E−012	3.1E−013	2.2169	6.7278E−013	−5.0E−012	1.41E−011
484	−2E−012	1.1E−013	2.2169	4.6896E−013	−3.5E−012	9.56E−012
485	−1E−012	1.0E−013	2.2169	4.3105E−013	−2.4E−012	6.84E−012
486	−1E−012	1.6E−013	2.2169	2.6512E−013	−1.6E−012	4.46E−012
487	−9E−013	−1E−013	2.2169	2.4093E−013	−9.9E−013	3.15E−012
488	−6E−013	1.9E−015	2.2169	1.1532E−013	−5.5E−013	1.98E−012
489	−1E−013	2.5E−013	2.2169	1.0005E−014	−6.7E−013	1.54E−012
490	−4E−013	−2E−014	2.2169	3.7137E−014	−6.2E−014	6.91E−013
491	−5E−013	−2E−013	2.2169	8.0997E−014	−1.7E−013	5.08E−013
492	−5E−014	5.0E−014	2.2169	4.3613E−015	1.732E−013	7.03E−014
493	−4E−013	−2E−013	2.2169	−1.1374E−014	1.624E−013	−4.6E−013
494	8.5E−014	4.1E−013	2.2169	1.4363E−013	4.089E−013	−7.8E−013
495	−7E−013	−3E−013	2.2169	8.7032E−014	3.962E−013	−1.2E−012
496	−8E−013	−2E−013	2.2169	1.1444E−013	8.722E−013	−2.3E−012
497	−7E−013	−3E−014	2.2169	1.7457E−013	1.280E−012	−3.2E−012
498	−1E−012	−7E−014	2.2169	2.7249E−013	1.617E−012	−4.4E−012
499	−2E−012	1.9E−013	2.2169	3.4551E−013	2.093E−012	−6.8E−012
500	−2E−012	4.8E−013	2.2169	5.5388E−013	3.417E−012	−9.7E−012
501	−3E−012	2.1E−013	2.2169	7.4599E−013	4.675E−012	−1.4E−011
502	−4E−012	6.9E−013	2.2169	1.1783E−012	7.377E−012	−2.0E−011
503	−7E−012	6.8E−013	2.2169	1.5243E−012	1.039E−011	−3.0E−011
504	−1E−011	4.2E−013	2.2169	2.2695E−012	1.526E−011	−4.3E−011
505	−1E−011	6.3E−013	2.2169	3.2945E−012	2.185E−011	−6.3E−011
506	−2E−011	1.4E−012	2.2169	4.9871E−012	3.171E−011	−9.2E−011
507	−3E−011	1.9E−012	2.2169	7.1493E−012	4.629E−011	−1.3E−010
508	−4E−011	2.7E−012	2.2169	1.0406E−011	6.701E−011	−1.9E−010
509	−6E−011	4.2E−012	2.2169	1.5253E−011	9.769E−011	−2.8E−010
510	−9E−011	6.0E−012	2.2169	2.2106E−011	1.419E−010	−4.1E−010
511	−1E−010	8.2E−012	2.2169	3.2143E−011	2.065E−010	−5.9E−010
512	−2E−010	1.2E−011	2.2169	4.6747E−011	2.999E−010	−8.6E−010
513	−3E−010	1.8E−011	2.2169	6.798E−011	4.358E−010	−1.3E−009

514	−4E−010	2.6E−011	2.2169	9.8836E−011	6.338E−010	−1.8E−009
515	−6E−010	3.8E−011	2.2169	1.4374E−010	9.218E−010	−2.7E−009
516	−9E−010	5.4E−011	2.2169	2.0926E−010	1.339E−009	−3.9E−009
517	−1E−009	8.0E−011	2.2169	3.0391E−010	1.948E−009	−5.6E−009
518	−2E−009	1.1E−010	2.2169	4.4216E−010	2.831E−009	−8.2E−009
519	−3E−009	1.7E−010	2.2169	6.4247E−010	4.117E−009	−1.2E−008
520	−4E−009	2.4E−010	2.2169	9.3531E−010	5.982E−009	−1.7E−008
521	−6E−009	3.6E−010	2.2169	1.3584E−009	8.702E−009	−2.5E−008
522	−8E−009	5.1E−010	2.2169	1.9778E−009	1.264E−008	−3.7E−008
523	−1E−008	7.6E−010	2.2169	2.8723E−009	1.839E−008	−5.3E−008
524	−2E−008	1.1E−009	2.2169	4.1825E−009	2.672E−008	−7.7E−008
525	−3E−008	1.6E−009	2.2169	6.0732E−009	3.888E−008	−1.1E−007
526	−4E−008	2.2E−009	2.2169	8.8456E−009	5.646E−008	−1.6E−007
527	−5E−008	3.4E−009	2.2169	1.2843E−008	8.217E−008	−2.4E−007
528	−8E−008	4.6E−009	2.2169	1.8708E−008	1.193E−007	−3.5E−007
529	−1E−007	7.3E−009	2.2169	2.7162E−008	1.737E−007	−5.0E−007
530	−2E−007	9.6E−009	2.2169	3.9573E−008	2.521E−007	−7.3E−007
531	−2E−007	1.6E−008	2.2169	5.7458E−008	3.670E−007	−1.1E−006
532	−4E−007	2.0E−008	2.2169	8.3717E−008	5.326E−007	−1.5E−006
533	−5E−007	3.4E−008	2.2169	1.2159E−007	7.756E−007	−2.2E−006
534	−7E−007	4E−008	2.2169	1.7714E−007	1.125E−006	−3.3E−006
535	−1E−006	7.4E−008	2.2169	2.5746E−007	1.639E−006	−4.7E−006
536	−2E−006	7.8E−008	2.2169	3.7496E−007	2.376E−006	−6.9E−006
537	−2E−006	1.6E−007	2.2169	5.4571E−007	3.463E−006	−1E−005
538	−3E−006	1.5E−007	2.2169	7.9419E−007	5.017E−006	−1.5E−005
539	−5E−006	3.8E−007	2.2169	1.1587E−006	7.317E−006	−2.1E−005
540	−7E−006	2.4E−007	2.2168	1.6842E−006	1.059E−005	−3.1E−005
541	−1E−005	9.0E−007	2.2168	2.4675E−006	1.546E−005	−4.5E−005
542	−2E−005	2.8E−007	2.2167	3.5795E−006	2.235E−005	−6.5E−005
543	−2E−005	2.3E−006	2.2167	5.2819E−006	3.268E−005	−9.5E−005
544	−3E−005	−2E−007	2.2166	7.6398E−006	4.713E−005	−0.000138
545	−4E−005	6.1E−006	2.2164	1.1404E−005	6.908E−005	−0.000201
546	−7E−005	−3E−006	2.2162	1.643E−005	9.920E−005	−0.000294
547	−9E−005	1.7E−005	2.2158	2.4969E−005	0.00014583	−0.000427
548	−0.00016	−2E−005	2.2154	3.5786E−005	0.00020756	−0.000623
549	−0.00017	5.3E−005	2.2147	5.582E−005	0.0003057	−0.000904
550	−0.00038	−7E−005	2.2136	7.9421E−005	0.00042585	−0.001320
551	−0.00030	0.000164	2.2123	0.0001281	0.00062315	−0.001906
552	−0.00090	−0.00031	2.21	0.00017987	0.00081889	−0.002763
553	−0.00037	0.000511	2.2074	0.00030054	0.0011464	−0.003920
554	−0.00218	−0.00128	2.2022	0.00040918	0.001229	−0.005514
555	0.0005965	0.001551	2.197	0.00070191	0.0012801	−0.007319
556	−0.00527	−0.00576	2.184	0.00088875	−0.0003996	−0.009148
557	0.0087819	0.004722	2.1721	0.0015101	−0.0043947	−0.008668
558	−0.01477	−0.03114	2.135	0.0029355	−0.01726	−0.002262
559	0.060004	0.015744	2.0938	0.010971	−0.049702	0.032701
560	0.21345	0.081784	2.2513	−0.011031	0.13622	0.29124

561	0.23633	0.064305	2.1736	−0.0016231	−0.19118	−0.19831
562	0.067165	0.024389	2.0678	0.0096304	0.067023	−0.028917
563	−0.01675	−0.03145	2.1522	0.0017944	0.02513	0.0020775
564	0.0068781	0.005558	2.2062	0.00085584	0.005728	0.0075257
565	−0.00702	−0.00539	2.2127	0.00078072	0.0009545	0.0080781
566	−0.00054	0.001760	2.2204	0.00066706	−0.0008042	0.0063515
567	−0.00317	−0.00105	2.2186	0.00044173	−0.0009840	0.0050638
568	−0.00097	0.000618	2.2194	0.00036042	−0.0009148	0.0036227
569	−0.00139	−0.00018	2.2183	0.00022315	−0.0006879	0.0026441
570	−0.00060	0.000222	2.2181	0.00017179	−0.0005185	0.0018426
571	−0.00061	−1E−005	2.2176	0.000107	−0.0003632	0.0013006
572	−0.00032	8.3E−005	2.2175	7.9334E−005	−0.0002599	0.00089798
573	−0.00027	1.3E−005	2.2173	5.0477E−005	−0.0001788	0.00062507
574	−0.00016	3.3E−005	2.2172	3.6569E−005	−0.0001256	0.00043017
575	−0.00012	1.1E−005	2.2171	2.3753E−005	−8.6E−005	0.0002976
576	−8E−005	1.4E−005	2.217	1.6954E−005	−6.0E−005	0.00020459
577	−6E−005	6.4E−006	2.217	1.1193E−005	−4.1E−005	0.00014114
578	−4E−005	5.9E−006	2.217	7.9092E−006	−2.8E−005	9.70E−005
579	−3E−005	3.4E−006	2.2169	5.283E−006	−2.0E−005	6.68E−005
580	−2E−005	2.7E−006	2.2169	3.707E−006	−1.4E−005	4.59E−005
581	−1E−005	1.7E−006	2.2169	2.4964E−006	−9.3E−006	3.16E−005
582	−8E−006	1.2E−006	2.2169	1.743E−006	−6.4E−006	2.17E−005
583	−6E−006	8.2E−007	2.2169	1.1804E−006	−4.4E−006	1.50E−005
584	−4E−006	5.7E−007	2.2169	8.212E−007	−3.0E−006	1.03E−005
585	−3E−006	3.9E−007	2.2169	5.583E−007	−2.1E−006	7.07E−006
586	−2E−006	2.7E−007	2.2169	3.874E−007	−1.4E−006	4.86E−006
587	−1E−006	1.9E−007	2.2169	2.641E−007	−9.8E−007	3.35E−006
588	−9E−007	1.3E−007	2.2169	1.829E−007	−6.8E−007	2.30E−006
589	−6E−007	8.9E−008	2.2169	1.2493E−007	−4.7E−007	1.58E−006
590	−4E−007	6.0E−008	2.2169	8.6401E−008	−3.2E−007	1.09E−006
591	−3E−007	4.2E−008	2.2169	5.91E−008	−2.2E−007	7.48E−007
592	−2E−007	2.8E−008	2.2169	4.0828E−008	−1.5E−007	5.14E−007
593	−1E−007	2.0E−008	2.2169	2.7957E−008	−1.0E−007	3.54E−007
594	−9E−008	1.3E−008	2.2169	1.9298E−008	−7.2E−008	2.43E−007
595	−6E−008	9.4E−009	2.2169	1.3225E−008	−4.9E−008	1.67E−007
596	−4E−008	6.3E−009	2.2169	9.1226E−009	−3.4E−008	1.15E−007
597	−3E−008	4.4E−009	2.2169	6.2556E−009	−2.3E−008	7.92E−008
598	−2E−008	3.0E−009	2.2169	4.313E−009	−1.6E−008	5.44E−008
599	−1E−008	2.1E−009	2.2169	2.959E−009	−1.1E−008	3.74E−008
600	−1E−008	1.4E−009	2.2169	2.0393E−009	−7.6E−009	2.57E−008
601	−7E−009	9.9E−010	2.2169	1.3996E−009	−5.2E−009	1.77E−008
602	−5E−009	6.7E−010	2.2169	9.6425E−010	−3.6E−009	1.22E−008
603	−3E−009	4.7E−010	2.2169	6.6204E−010	−2.5E−009	8.38E−009
604	−2E−009	3.2E−010	2.2169	4.5603E−010	−1.7E−009	5.76E−009
605	−2E−009	2.2E−010	2.2169	3.1314E−010	−1.2E−009	3.96E−009
606	−1E−009	1.5E−010	2.2169	2.1565E−010	−8.1E−010	2.72E−009
607	−7E−010	1.0E−010	2.2169	1.4813E−010	−5.5E−010	1.87E−009

608	−5E−010	7.1E−011	2.2169	1.0197E−010	−3.8E−010	1.29E−009
609	−3E−010	4.9E−011	2.2169	7.0109E−011	−2.6E−010	8.86E−010
610	−2E−010	3.3E−011	2.2169	4.823E−011	−1.8E−010	6.10E−010
611	−2E−010	2.3E−011	2.2169	3.3179E−011	−1.2E−010	4.19E−010
612	−1E−010	1.6E−011	2.2169	2.2795E−011	−8.5E−011	2.88E−010
613	−8E−011	1.1E−011	2.2169	1.57E−011	−5.9E−011	1.98E−010
614	−5E−011	7.5E−012	2.2169	1.0791E−011	−4.0E−011	1.36E−010
615	−4E−011	5.4E−012	2.2169	7.3924E−012	−2.8E−011	9.39E−011
616	−2E−011	3.7E−012	2.2169	5.1185E−012	−1.9E−011	6.46E−011
617	−2E−011	2.4E−012	2.2169	3.4962E−012	−1.3E−011	4.44E−011
618	−1E−011	2E−012	2.2169	2.4358E−012	−8.9E−012	3.06E−011
619	−8E−012	1.3E−012	2.2169	1.6269E−012	−6.2E−012	2.14E−011
620	−5E−012	8.3E−013	2.2169	1.1376E−012	−4.3E−012	1.44E−011
621	−4E−012	5.7E−013	2.2169	7.9239E−013	−2.8E−012	9.91E−012
622	−3E−012	2.7E−013	2.2169	5.1172E−013	−2.1E−012	6.79E−012
623	−2E−012	−2E−013	2.2169	3.7547E−013	−1.4E−012	4.70E−012
624	−1E−012	4.0E−013	2.2169	2.8786E−013	−9.0E−013	3.21E−012
625	−1E−012	−2E−013	2.2169	1.7153E−013	−7.9E−013	2.13E−012
626	−6E−013	1.7E−013	2.2169	1.1379E−013	−8.3E−014	1.27E−012
627	−7E−013	−1E−013	2.2169	6.5087E−014	−3.1E−013	7.61E−013
628	−2E−013	2.2E−014	2.2169	6.4456E−014	−3.1E−013	3.43E−013
629	−4E−013	6.8E−014	2.2169	7.0253E−015	1.884E−013	−7.7E−014
630	−4E−013	−7E−014	2.2169	1.2793E−013	1.060E−013	−4.4E−013
631	−4E−013	2.0E−013	2.2169	1.2714E−013	−3.0E−014	−1.0E−012
632	−1E−012	−4E−013	2.2169	9.7448E−014	4.847E−013	−1.3E−012
633	−6E−013	2.5E−013	2.2169	2.4958E−013	8.013E−013	−2.1E−012
634	−1E−012	1.5E−013	2.2169	2.1567E−013	7.132E−013	−3.2E−012
635	−2E−012	1.3E−013	2.2169	2.9035E−013	1.400E−012	−4.7E−012
636	−3E−012	8.1E−014	2.2169	5.1943E−013	1.833E−012	−6.7E−012
637	−4E−012	−6E−014	2.2169	7.3919E−013	2.831E−012	−9.8E−012
638	−6E−012	7.8E−013	2.2169	1.145E−012	4.268E−012	−1.4E−011
639	−8E−012	1.2E−012	2.2169	1.6815E−012	6.294E−012	−2.1E−011
640	−1E−011	1.7E−012	2.2169	2.4337E−012	9.088E−012	−3.0E−011
641	−2E−011	2.7E−012	2.2169	3.4806E−012	1.316E−011	−4.4E−011
642	−2E−011	3.5E−012	2.2169	4.9719E−012	1.913E−011	−6.4E−011
643	−4E−011	5.5E−012	2.2169	7.3501E−012	2.746E−011	−9.4E−011
644	−5E−011	7.8E−012	2.2169	1.0766E−011	4.070E−011	−1.4E−010
645	−8E−011	1.1E−011	2.2169	1.5773E−011	5.875E−011	−2.0E−010
646	−1E−010	1.6E−011	2.2169	2.2867E−011	8.532E−011	−2.9E−010
647	−2E−010	2.3E−011	2.2169	3.3134E−011	1.242E−010	−4.2E−010
648	−2E−010	3.4E−011	2.2169	4.8242E−011	1.805E−010	−6.1E−010
649	−3E−010	4.9E−011	2.2169	7.0069E−011	2.621E−010	−8.9E−010
650	−5E−010	7.1E−011	2.2169	1.0201E−010	3.810E−010	−1.3E−009
651	−7E−010	1.0E−010	2.2169	1.4821E−010	5.539E−010	−1.9E−009
652	−1E−009	1.5E−010	2.2169	2.1565E−010	8.058E−010	−2.7E−009
653	−2E−009	2.2E−010	2.2169	3.1325E−010	1.171E−009	−4.0E−009
654	−2E−009	3.2E−010	2.2169	4.5603E−010	1.703E−009	−5.8E−009

655	−3E−009	4.7E−010	2.2169	6.6212E−010	2.475E−009	−8.4E−009
656	−5E−009	6.7E−010	2.2169	9.6436E−010	3.601E−009	−1.2E−008
657	−7E−009	9.9E−010	2.2169	1.3996E−009	5.231E−009	−1.8E−008
658	−1E−008	1.4E−009	2.2169	2.0393E−009	7.611E−009	−2.6E−008
659	−1E−008	2.1E−009	2.2169	2.959E−009	1.106E−008	−3.7E−008
660	−2E−008	3.0E−009	2.2169	4.313E−009	1.609E−008	−5.4E−008
661	−3E−008	4.4E−009	2.2169	6.2555E−009	2.337E−008	−7.9E−008
662	−4E−008	6.3E−009	2.2169	9.1225E−009	3.401E−008	−1.2E−007
663	−6E−008	9.4E−009	2.2169	1.3225E−008	4.94E−008	−1.7E−007
664	−9E−008	1.3E−008	2.2169	1.9298E−008	7.189E−008	−2.4E−007
665	−1E−007	2.0E−008	2.2169	2.7957E−008	1.044E−007	−3.5E−007
666	−2E−007	2.8E−008	2.2169	4.0828E−008	1.520E−007	−5.1E−007
667	−3E−007	4.2E−008	2.2169	5.91E−008	2.206E−007	−7.5E−007
668	−4E−007	6.0E−008	2.2169	8.6401E−008	3.212E−007	−1.1E−006
669	−6E−007	8.9E−008	2.2169	1.2493E−007	4.661E−007	−1.6E−006
670	−9E−007	1.3E−007	2.2169	1.829E−007	6.788E−007	−2.3E−006
671	−1E−006	1.9E−007	2.2169	2.641E−007	9.847E−007	−3.3E−006
672	−2E−006	2.7E−007	2.2169	3.874E−007	1.434E−006	−4.9E−006
673	−3E−006	3.9E−007	2.2169	5.583E−007	2.080E−006	−7.1E−006
674	−4E−006	5.7E−007	2.2169	8.212E−007	3.030E−006	−1.0E−005
675	−6E−006	8.2E−007	2.2169	1.1804E−006	4.390E−006	−1.5E−005
676	−8E−006	1.2E−006	2.2169	1.743E−006	6.399E−006	−2.2E−005
677	−1E−005	1.7E−006	2.2169	2.4964E−006	9.26E−006	−3.2E−005
678	−2E−005	2.7E−006	2.2169	3.707E−006	1.351E−005	−4.6E−005
679	−3E−005	3.4E−006	2.2169	5.283E−006	1.951E−005	−6.7E−005
680	−4E−005	5.9E−006	2.217	7.9092E−006	2.848E−005	−9.7E−005
681	−6E−005	6.4E−006	2.217	1.1193E−005	4.105E−005	−0.000141
682	−8E−005	1.4E−005	2.217	1.6954E−005	5.995E−005	−0.000205
683	−0.00012	1.1E−005	2.2171	2.3753E−005	8.603E−005	−0.000298
684	−0.00016	3.3E−005	2.2172	3.6569E−005	0.00012559	−0.000430
685	−0.00027	1.3E−005	2.2173	5.0477E−005	0.00017879	−0.000625
686	−0.00032	8.3E−005	2.2175	7.9334E−005	0.00025988	−0.000898
687	−0.00061	−1E−005	2.2176	0.000107	0.00036315	−0.001301
688	−0.00060	0.000222	2.2181	0.00017179	0.00051852	−0.001843
689	−0.00139	−0.00018	2.2183	0.00022315	0.00068788	−0.002644
690	−0.00097	0.000618	2.2194	0.00036042	0.00091479	−0.003623
691	−0.00317	−0.00105	2.2186	0.00044173	0.00098396	−0.005064
692	−0.00054	0.001760	2.2204	0.00066706	0.00080425	−0.006352
693	−0.00702	−0.00539	2.2127	0.00078072	−0.0009545	−0.008078
694	0.0068781	0.005558	2.2062	0.00085584	−0.005728	−0.007526
695	−0.01675	−0.03145	2.1522	0.0017944	−0.02513	−0.002077
696	0.067165	0.024389	2.0678	0.0096304	−0.067023	0.028917
697	0.23633	0.064305	2.1736	−0.0016231	0.19118	0.19831
698	0.15843	−0.05350	2.1326	0.00055212	−0.23496	−0.10152
699	0.086993	0.072788	1.9834	0.009555	0.078823	−0.011411
700	−0.02898	−0.04364	2.1831	−0.0017188	0.01464	0.0070673
701	0.010956	0.013716	2.235	0.00090576	0.0029938	0.0025968

702	−0.00902	−0.00710	2.2375	6.5806E−005	0.0016173	0.004393
703	−0.00115	0.002649	2.2352	0.00030426	−0.0008270	0.0029294
704	−0.00388	−0.00123	2.2306	0.00013859	−0.0002905	0.0026419
705	−0.00165	0.000664	2.2269	0.00014357	−0.0006086	0.0018082
706	−0.00179	−0.00022	2.224	8.1867E−005	−0.0003384	0.0013924
707	−0.00098	0.000205	2.2219	6.6889E−005	−0.0003154	0.00095734
708	−0.00083	−3E−005	2.2204	4.1507E−005	−0.0002001	0.00069239
709	−0.00050	7.2E−005	2.2193	3.082E−005	−0.0001534	0.000477
710	−0.00038	2.4E−006	2.2186	2.0101E−005	−0.0001032	0.00033535
711	−0.00024	2.8E−005	2.2181	1.4252E−005	−7.3E−005	0.00023124
712	−0.00018	5.5E−006	2.2177	9.5775E−006	−5.1E−005	0.00016042
713	−0.00012	1.1E−005	2.2175	6.6378E−006	−3.5E−005	0.00011066
714	−8E−005	3.8E−006	2.2173	4.5367E−006	−2.4E−005	7.63E−005
715	−6E−005	4.9E−006	2.2172	3.1104E−006	−1.7E−005	5.26E−005
716	−4E−005	2.1E−006	2.2171	2.1446E−006	−1.2E−005	3.62E−005
717	−3E−005	2.2E−006	2.217	1.4635E−006	−7.9E−006	2.50E−005
718	−2E−005	1.1E−006	2.217	1.0132E−006	−5.5E−006	1.71E−005
719	−1E−005	9.8E−007	2.217	6.9031E−007	−3.7E−006	1.18E−005
720	−9E−006	5.4E−007	2.2169	4.7861E−007	−2.6E−006	8.11E−006
721	−6E−006	4.5E−007	2.2169	3.2607E−007	−1.8E−006	5.59E−006
722	−4E−006	2.6E−007	2.2169	2.261E−007	−1.2E−006	3.84E−006
723	−3E−006	2.1E−007	2.2169	1.5414E−007	−8.4E−007	2.64E−006
724	−2E−006	1.3E−007	2.2169	1.0683E−007	−5.9E−007	1.82E−006
725	−1E−006	9.8E−008	2.2169	7.2893E−008	−4.0E−007	1.25E−006
726	−9E−007	6.1E−008	2.2169	5.0483E−008	−2.8E−007	8.59E−007
727	−6E−007	4.6E−008	2.2169	3.4478E−008	−1.9E−007	5.91E−007
728	−4E−007	2.9E−008	2.2169	2.3859E−008	−1.3E−007	4.06E−007
729	−3E−007	2.1E−008	2.2169	1.631E−008	−8.9E−008	2.80E−007
730	−2E−007	1.4E−008	2.2169	1.1277E−008	−6.2E−008	1.92E−007
731	−1E−007	1.0E−008	2.2169	7.7155E−009	−4.2E−008	1.32E−007
732	−1E−007	6.6E−009	2.2169	5.3309E−009	−2.9E−008	9.09E−008
733	−7E−008	4.8E−009	2.2169	3.6499E−009	−2.0E−008	6.26E−008
734	−5E−008	3.1E−009	2.2169	2.5201E−009	−1.4E−008	4.30E−008
735	−3E−008	2.2E−009	2.2169	1.7266E−009	−9.4E−009	2.96E−008
736	−2E−008	1.5E−009	2.2169	1.1915E−009	−6.5E−009	2.03E−008
737	−1E−008	1.1E−009	2.2169	8.1673E−010	−4.5E−009	1.40E−008
738	−1E−008	7.0E−010	2.2169	5.6338E−010	−3.1E−009	9.62E−009
739	−7E−009	5.0E−010	2.2169	3.8636E−010	−2.1E−009	6.62E−009
740	−5E−009	3.3E−010	2.2169	2.6635E−010	−1.5E−009	4.55E−009
741	−3E−009	2.3E−010	2.2169	1.8275E−010	−1.0E−009	3.13E−009
742	−2E−009	1.6E−010	2.2169	1.2596E−010	−6.9E−010	2.15E−009
743	−2E−009	1.1E−010	2.2169	8.6488E−011	−4.7E−010	1.48E−009
744	−1E−009	7.5E−011	2.2169	5.9549E−011	−3.3E−010	1.02E−009
745	−7E−010	5.3E−011	2.2169	4.0929E−011	−2.2E−010	7.00E−010
746	−5E−010	3.5E−011	2.2169	2.8197E−011	−1.5E−010	4.82E−010
747	−4E−010	2.5E−011	2.2169	1.927E−011	−1.1E−010	3.31E−010
748	−2E−010	1.7E−011	2.2169	1.3295E−011	−7.3E−011	2.28E−010

749	−2E−010	1.2E−011	2.2169	9.1765E−012	−5.0E−011	1.57E−010
750	−1E−010	7.6E−012	2.2169	6.3527E−012	−3.4E−011	1.08E−010
751	−8E−011	5.4E−012	2.2169	4.3356E−012	−2.4E−011	7.42E−011
752	−5E−011	3.5E−012	2.2169	3.0326E−012	−1.6E−011	5.09E−011
753	−4E−011	2.3E−012	2.2169	2.0711E−012	−1.1E−011	3.50E−011
754	−3E−011	1.5E−012	2.2169	1.3837E−012	−7.9E−012	2.40E−011
755	−2E−011	1.0E−012	2.2169	1.0076E−012	−5.4E−012	1.65E−011
756	−1E−011	7.0E−013	2.2169	6.9296E−013	−3.8E−012	1.15E−011
757	−8E−012	4.6E−013	2.2169	4.1984E−013	−2.5E−012	7.92E−012
758	−6E−012	6.9E−013	2.2169	2.7803E−013	−1.5E−012	5.36E−012
759	−4E−012	−8E−014	2.2169	2.3349E−013	−1.2E−012	3.46E−012
760	−3E−012	1.2E−013	2.2169	1.1304E−013	−9.4E−013	2.50E−012
761	−2E−012	2.0E−013	2.2169	1.8403E−013	−4.4E−013	1.88E−012
762	−2E−012	2.2E−014	2.2169	−1.4152E−014	−1.1E−013	1.17E−012
763	−1E−012	−5E−014	2.2169	3.3517E−014	−1.7E−013	6.90E−013
764	−7E−013	5.8E−014	2.2169	2.7366E−014	−1.1E−013	3.45E−013
765	−4E−013	1.9E−013	2.2169	2.6868E−014	1.880E−013	5.15E−013
766	−7E−013	1.7E−014	2.2169	3.7983E−014	−8.1E−014	−2.6E−013
767	−1E−012	−2E−013	2.2169	2.4966E−015	3.702E−013	−2.4E−013
768	−9E−013	−1E−013	2.2169	−8.0987E−015	−2.1E−014	−2.6E−013
769	−9E−013	6.3E−014	2.2169	1.934E−014	7.425E−013	−9.2E−013
770	−2E−012	1.2E−013	2.2169	7.818E−014	1.910E−013	−1.2E−012
771	−2E−012	1.7E−013	2.2169	1.6138E−013	8.752E−013	−1.7E−012
772	−2E−012	6.6E−013	2.2169	1.9304E−013	9.132E−013	−2.5E−012
773	−4E−012	3.8E−013	2.2169	1.8847E−013	1.308E−012	−3.7E−012
774	−6E−012	1.1E−013	2.2169	3.458E−013	2.141E−012	−5.4E−012
775	−8E−012	5.1E−013	2.2169	5.1932E−013	1.877E−012	−7.6E−012
776	−1E−011	7.1E−013	2.2169	7.1298E−013	3.595E−012	−1.1E−011
777	−2E−011	1.3E−012	2.2169	9.7049E−013	5.642E−012	−1.6E−011
778	−3E−011	1.7E−012	2.2169	1.4002E−012	7.154E−012	−2.4E−011
779	−4E−011	2.5E−012	2.2169	2.029E−012	1.098E−011	−3.5E−011
780	−5E−011	3.4E−012	2.2169	2.9587E−012	1.674E−011	−5.1E−011
781	−8E−011	5.1E−012	2.2169	4.3209E−012	2.336E−011	−7.4E−011
782	−1E−010	7.9E−012	2.2169	6.3416E−012	3.439E−011	−1.1E−010
783	−2E−010	1.2E−011	2.2169	9.2215E−012	5.008E−011	−1.6E−010
784	−2E−010	1.7E−011	2.2169	1.3237E−011	7.259E−011	−2.3E−010
785	−4E−010	2.4E−011	2.2169	1.9312E−011	1.062E−010	−3.3E−010
786	−5E−010	3.5E−011	2.2169	2.8166E−011	1.542E−010	−4.8E−010
787	−7E−010	5.2E−011	2.2169	4.0839E−011	2.232E−010	−7E−010
788	−1E−009	7.5E−011	2.2169	5.953E−011	3.260E−010	−1.0E−009
789	−2E−009	1.1E−010	2.2169	8.6417E−011	4.724E−010	−1.5E−009
790	−2E−009	1.6E−010	2.2169	1.2599E−010	6.890E−010	−2.2E−009
791	−3E−009	2.3E−010	2.2169	1.8269E−010	9.977E−010	−3.1E−009
792	−5E−009	3.3E−010	2.2169	2.6642E−010	1.458E−009	−4.5E−009
793	−7E−009	5.0E−010	2.2169	3.8634E−010	2.109E−009	−6.6E−009
794	−1E−008	7.0E−010	2.2169	5.6332E−010	3.084E−009	−9.6E−009
795	−1E−008	1.1E−009	2.2169	8.1678E−010	4.457E−009	−1.4E−008

796	−2E−008	1.5E−009	2.2169	1.1915E−009	6.524E−009	−2.0E−008
797	−3E−008	2.2E−009	2.2169	1.7266E−009	9.419E−009	−3.0E−008
798	−5E−008	3.1E−009	2.2169	2.5201E−009	1.380E−008	−4.3E−008
799	−7E−008	4.8E−009	2.2169	3.6499E−009	1.990E−008	−6.3E−008
800	−1E−007	6.6E−009	2.2169	5.3308E−009	2.920E−008	−9.1E−008
801	−1E−007	1.0E−008	2.2169	7.7155E−009	4.205E−008	−1.3E−007
802	−2E−007	1.4E−008	2.2169	1.1277E−008	6.179E−008	−1.9E−007
803	−3E−007	2.1E−008	2.2169	1.631E−008	8.884E−008	−2.8E−007
804	−4E−007	2.9E−008	2.2169	2.3859E−008	1.308E−007	−4.1E−007
805	−6E−007	4.6E−008	2.2169	3.4478E−008	1.876E−007	−5.9E−007
806	−9E−007	6.1E−008	2.2169	5.0483E−008	2.767E−007	−8.6E−007
807	−1E−006	9.8E−008	2.2169	7.2892E−008	3.963E−007	−1.3E−006
808	−2E−006	1.3E−007	2.2169	1.0683E−007	5.855E−007	−1.8E−006
809	−3E−006	2.1E−007	2.2169	1.5414E−007	8.365E−007	−2.6E−006
810	−4E−006	2.6E−007	2.2169	2.261E−007	1.239E−006	−3.8E−006
811	−6E−006	4.5E−007	2.2169	3.2607E−007	1.766E−006	−5.6E−006
812	−9E−006	5.4E−007	2.2169	4.7861E−007	2.619E−006	−8.1E−006
813	−1E−005	9.8E−007	2.217	6.9031E−007	3.725E−006	−1.2E−005
814	−2E−005	1.1E−006	2.217	1.0132E−006	5.529E−006	−1.7E−005
815	−3E−005	2.2E−006	2.217	1.4635E−006	7.856E−006	−2.5E−005
816	−4E−005	2.1E−006	2.2171	2.1446E−006	1.165E−005	−3.6E−005
817	−6E−005	4.9E−006	2.2172	3.1104E−006	1.656E−005	−5.3E−005
818	−8E−005	3.8E−006	2.2173	4.5367E−006	2.442E−005	−7.6E−005
819	−0.00012	1.1E−005	2.2175	6.6378E−006	3.488E−005	−0.000111
820	−0.00018	5.5E−006	2.2177	9.5775E−006	5.070E−005	−0.000160
821	−0.00024	2.8E−005	2.2181	1.4252E−005	7.334E−005	−0.000231
822	−0.00038	2.4E−006	2.2186	2.0101E−005	0.00010317	−0.000335
823	−0.00050	7.2E−005	2.2193	3.082E−005	0.0001534	−0.000477
824	−0.00083	−3E−005	2.2204	4.1507E−005	0.0002001	−0.000692
825	−0.00098	0.000205	2.2219	6.6889E−005	0.00031539	−0.000957
826	−0.00179	−0.00022	2.224	8.1867E−005	0.00033844	−0.001392
827	−0.00165	0.000664	2.2269	0.00014357	0.00060863	−0.001808
828	−0.00388	−0.00123	2.2306	0.00013859	0.00029046	−0.002642
829	−0.00115	0.002649	2.2352	0.00030426	0.00082705	−0.002929
830	−0.00902	−0.00710	2.2375	6.5806E−005	−0.0016173	−0.004393
831	0.010956	0.013716	2.235	0.00090576	−0.0029938	−0.002597
832	−0.02898	−0.04364	2.1831	−0.0017188	−0.01464	−0.007067
833	0.086993	0.072788	1.9834	0.009555	−0.078823	0.011411
834	0.15843	−0.05350	2.1326	0.00055212	0.23496	0.10152
835	2.7904	2.7745	3.099	1.508E−010	−6.4E−010	0.70781
836	2.127	2.142	2.3845	−0.0058906	−0.58041	0.13816
837	1.9396	1.9293	2.1942	−0.017601	−0.45971	0.31364
838	2.0215	1.9757	2.281	−0.016543	−0.31923	0.49178
839	2.3204	2.2445	2.5891	0.00035693	−0.14822	0.68826
840	2.7904	2.7745	3.099	1.5098E−010	6.353E−010	−0.70781
841	−0.15411	−0.09376	2.6371	6.5386E−010	9.981E−011	0.37212
842	0.15176	0.10327	1.8677	−1.2699E−010	3.605E−010	−0.011648

843	−0.05310	−0.06027	2.0314	−4.0173E−011	−1.7E−011	0.00066743
844	0.020061	0.014108	2.1064	−2.126E−011	−6.6E−011	−0.002565
845	−0.01015	−0.01171	2.1343	−1.2153E−011	−5.2E−011	−0.003473
846	0.0033302	0.002659	2.1668	−3.2273E−012	−3.7E−011	−0.002510
847	−0.00254	−0.00284	2.1815	−3.5382E−012	−2.5E−011	−0.002176
848	0.0006067	0.000603	2.1948	−5.4275E−013	−1.7E−011	−0.001317
849	−0.00070	−0.00075	2.2015	−1.0312E−012	−1.1E−011	−0.001046
850	8.0E−005	0.000132	2.2069	−1.0583E−013	−7.3E−012	−0.000627
851	−0.00021	−0.00021	2.21	−3.3044E−013	−4.7E−012	−0.000480
852	−1E−005	2.2E−005	2.2123	−6.5898E−015	−3.3E−012	−0.000293
853	−7E−005	−6E−005	2.2137	−1.0259E−013	−2.2E−012	−0.000220
854	−2E−005	−5E−007	2.2148	−1.4125E−014	−1.5E−012	−0.000137
855	−2E−005	−2E−005	2.2154	−4.0268E−014	−9.7E−013	−0.000101
856	−1E−005	−3E−006	2.2159	9.331E−015	−6.6E−013	−6.5E−005
857	−1E−005	−7E−006	2.2162	−4.0102E−016	−4.4E−013	−4.7E−005
858	−6E−006	−2E−006	2.2164	−3.7832E−014	−2.7E−013	−3.1E−005
859	−4E−006	−3E−006	2.2166	1.9323E−014	−2.4E−013	−2.2E−005
860	−3E−006	−1E−006	2.2167	−8.9823E−015	−1.1E−013	−1.4E−005
861	−2E−006	−1E−006	2.2167	2.7879E−015	−1.3E−013	−1.0E−005
862	−1E−006	−6E−007	2.2168	3.2873E−014	−3.7E−014	−6.9E−006
863	−9E−007	−5E−007	2.2168	−4.7369E−015	−1.5E−013	−4.9E−006
864	−7E−007	−3E−007	2.2169	−8.689E−015	3.609E−015	−3.2E−006
865	−4E−007	−2E−007	2.2169	−3.1387E−014	1.474E−013	−2.3E−006
866	−3E−007	−2E−007	2.2169	2.4215E−014	2.076E−013	−1.5E−006
867	−2E−007	−1E−007	2.2169	2.0544E−014	−9.9E−014	−1.1E−006
868	−2E−007	−7E−008	2.2169	−4.8631E−015	−8.1E−015	−7.3E−007
869	−9E−008	−5E−008	2.2169	−8.6019E−016	−1.7E−014	−5.1E−007
870	−7E−008	−3E−008	2.2169	−1.9855E−014	9.473E−014	−3.4E−007
871	−4E−008	−2E−008	2.2169	−9.0938E−015	1.852E−013	−2.4E−007
872	−3E−008	−2E−008	2.2169	2.0897E−014	−5.2E−014	−1.6E−007
873	−2E−008	−1E−008	2.2169	2.2125E−014	−5.7E−014	−1.1E−007
874	−2E−008	−8E−009	2.2169	−1.4023E−014	5.181E−014	−7.7E−008
875	−1E−008	−5E−009	2.2169	2.4897E−014	−1.1E−013	−5.4E−008
876	−7E−009	−4E−009	2.2169	−4.4786E−014	6.938E−015	−3.6E−008
877	−5E−009	−3E−009	2.2169	2.219E−014	7.980E−014	−2.5E−008
878	−3E−009	−2E−009	2.2169	2.4264E−015	−3.7E−014	−1.7E−008
879	−2E−009	−1E−009	2.2169	2.1542E−014	3.924E−014	−1.2E−008
880	−2E−009	−8E−010	2.2169	−1.2132E−014	1.142E−013	−8.2E−009
881	−1E−009	−6E−010	2.2169	1.108E−014	1.457E−013	−5.7E−009
882	−8E−010	−4E−010	2.2169	−2.2871E−014	1.439E−013	−3.9E−009
883	−5E−010	−3E−010	2.2169	1.1189E−014	1.213E−013	−2.7E−009
884	−4E−010	−2E−010	2.2169	−1.1893E−014	1.698E−013	−1.8E−009
885	−2E−010	−1E−010	2.2169	−2.9021E−014	9.256E−014	−1.3E−009
886	−2E−010	−9E−011	2.2169	2.8738E−014	1.829E−013	−8.6E−010
887	−1E−010	−6E−011	2.2169	−3.5525E−014	2.494E−013	−6.0E−010
888	−8E−011	−4E−011	2.2169	6.1795E−014	−1.8E−013	−4.1E−010
889	−5E−011	−3E−011	2.2169	−1.0708E−014	−7.1E−014	−2.8E−010

890	−4E−011	−2E−011	2.2169	−3.2045E−014	−2.1E−013	−1.9E−010
891	−3E−011	−1E−011	2.2169	1.0458E−015	−2.3E−014	−1.3E−010
892	−2E−011	−9E−012	2.2169	1.7467E−015	−1.5E−013	−9.2E−011
893	−1E−011	−6E−012	2.2169	−2.8056E−014	1.564E−013	−6.3E−011
894	−9E−012	−4E−012	2.2169	2.7444E−014	1.527E−013	−4.4E−011
895	−6E−012	−3E−012	2.2169	−3.5382E−015	4.034E−013	−2.9E−011
896	−4E−012	−2E−012	2.2169	−2.129E−014	3.649E−014	−2.1E−011
897	−3E−012	−1E−012	2.2169	6.5959E−014	1.336E−013	−1.4E−011
898	−2E−012	−9E−013	2.2169	−3.9143E−014	−2.0E−013	−9.5E−012
899	−1E−012	−1E−012	2.2169	−8.5459E−016	−2.6E−013	−7.0E−012
900	−1E−012	−6E−013	2.2169	8.6067E−015	−2.8E−013	−4.3E−012
901	−7E−013	−5E−013	2.2169	−5.2904E−014	−4.2E−013	−3.3E−012
902	−6E−013	−9E−013	2.2169	3.0477E−014	−2.5E−013	−2.2E−012
903	−1E−013	3.6E−014	2.2169	−4.9996E−014	−1.4E−013	−2E−012
904	4.6E−014	2.3E−013	2.2169	3.2964E−014	1.698E−013	−9.3E−013
905	−1E−013	−7E−014	2.2169	−2.5442E−014	1.110E−013	−4.7E−013
906	−8E−014	−4E−013	2.2169	6.2322E−014	1.095E−013	−2.0E−013
907	−8E−013	−5E−013	2.2169	−8.5636E−016	−3.4E−013	−2.3E−013
908	2.5E−013	4.8E−013	2.2169	6.1798E−014	−2.8E−013	−3.9E−013
909	1.2E−013	−1E−013	2.2169	−9.3836E−014	−4.6E−014	2.41E−013
910	−3E−013	−2E−013	2.2169	2.1677E−014	−6.8E−014	−3.7E−013
911	5.8E−013	4.1E−013	2.2169	2.3362E−014	−2.0E−013	3.63E−013
912	−3E−013	−7E−013	2.2169	−2.4307E−014	−3.1E−013	1.06E−013
913	−7E−013	−7E−013	2.2169	−3.7505E−014	−3.3E−013	3.40E−014
914	−2E−013	−3E−013	2.2169	−6.8778E−014	1.367E−013	1.60E−012
915	−9E−014	1.7E−014	2.2169	2.8981E−014	−6.4E−014	1.19E−012
916	−2E−013	−3E−014	2.2169	−1.205E−014	−1.5E−013	1.84E−012
917	−5E−013	−2E−013	2.2169	−4.543E−014	−2.7E−013	2.81E−012
918	−2E−012	−3E−013	2.2169	9.0871E−014	−2.0E−013	4.92E−012
919	−1E−012	−4E−013	2.2169	−5.0313E−014	1.892E−013	6.85E−012
920	−2E−012	−1E−012	2.2169	9.1546E−014	6.941E−014	9.77E−012
921	−3E−012	−2E−012	2.2169	1.0879E−014	−2.3E−013	1.37E−011
922	−4E−012	−2E−012	2.2169	−6.8975E−014	1.137E−013	2.07E−011
923	−7E−012	−4E−012	2.2169	2.9069E−014	2.664E−014	2.95E−011
924	−8E−012	−4E−012	2.2169	−7.3342E−014	−2.2E−013	4.40E−011
925	−1E−011	−6E−012	2.2169	4.2564E−015	2.333E−013	6.19E−011
926	−2E−011	−1E−011	2.2169	−1.3057E−014	5.159E−013	9.19E−011
927	−3E−011	−1E−011	2.2169	4.8993E−014	−1.3E−013	1.33E−010
928	−4E−011	−2E−011	2.2169	7.2782E−015	−2.4E−013	1.94E−010
929	−5E−011	−3E−011	2.2169	−5.8137E−014	3.743E−014	2.82E−010
930	−8E−011	−4E−011	2.2169	2.6613E−014	2.339E−013	4.09E−010
931	−1E−010	−6E−011	2.2169	−1.3191E−013	−1.4E−013	5.97E−010
932	−2E−010	−9E−011	2.2169	8.1961E−014	3.755E−013	8.65E−010
933	−2E−010	−1E−010	2.2169	−9.6367E−014	3.262E−013	1.26E−009
934	−4E−010	−2E−010	2.2169	1.6364E−013	1.468E−013	1.83E−009
935	−5E−010	−3E−010	2.2169	1.6834E−014	−5.3E−013	2.67E−009
936	−8E−010	−4E−010	2.2169	−1.097E−014	−4.0E−013	3.86E−009

937	−1E−009	−6E−010	2.2169	−6.7053E−014	−2.3E−013	5.66E−009
938	−2E−009	−8E−010	2.2169	2.2226E−014	9.126E−014	8.17E−009
939	−2E−009	−1E−009	2.2169	−5.0048E−014	−2.5E−013	1.20E−008
940	−3E−009	−2E−009	2.2169	9.3051E−014	−4.0E−013	1.73E−008
941	−5E−009	−3E−009	2.2169	−5.6028E−014	−2.8E−014	2.53E−008
942	−7E−009	−4E−009	2.2169	−3.1814E−014	−1.0E−013	3.65E−008
943	−1E−008	−5E−009	2.2169	1.0293E−014	1.351E−014	5.37E−008
944	−2E−008	−8E−009	2.2169	3.0589E−016	3.166E−013	7.71E−008
945	−2E−008	−1E−008	2.2169	1.0012E−013	8.691E−014	1.14E−007
946	−3E−008	−2E−008	2.2169	−2.3676E−014	−4.8E−013	1.63E−007
947	−4E−008	−2E−008	2.2169	1.3851E−014	−1.8E−013	2.41E−007
948	−7E−008	−3E−008	2.2169	−6.1968E−015	−2.0E−013	3.44E−007
949	−9E−008	−5E−008	2.2169	7.9494E−015	−2.7E−013	5.10E−007
950	−2E−007	−7E−008	2.2169	3.3171E−014	−4.9E−013	7.28E−007
951	−2E−007	−1E−007	2.2169	−1.4818E−013	−5.4E−013	1.08E−006
952	−3E−007	−2E−007	2.2169	3.2283E−014	−2.2E−013	1.54E−006
953	−4E−007	−2E−007	2.2169	−1.1201E−014	−4.2E−013	2.30E−006
954	−7E−007	−3E−007	2.2169	6.9832E−014	6.919E−014	3.25E−006
955	−9E−007	−5E−007	2.2168	−9.1165E−014	−2.4E−013	4.87E−006
956	−1E−006	−6E−007	2.2168	5.547E−014	4.422E−013	6.85E−006
957	−2E−006	−1E−006	2.2167	1.2373E−013	−3.4E−013	1.04E−005
958	−3E−006	−1E−006	2.2167	−4.904E−014	−2.8E−013	1.45E−005
959	−4E−006	−3E−006	2.2166	−1.159E−013	4.671E−013	2.21E−005
960	−6E−006	−2E−006	2.2164	5.8023E−014	3.723E−013	3.06E−005
961	−1E−005	−7E−006	2.2162	−5.0638E−014	−3.5E−015	4.72E−005
962	−1E−005	−3E−006	2.2159	−1.2832E−013	5.169E−013	6.47E−005
963	−2E−005	−2E−005	2.2154	5.9419E−014	4.566E−013	0.00010144
964	−2E−005	−5E−007	2.2148	−6.3462E−014	1.659E−012	0.00013741
965	−7E−005	−6E−005	2.2137	−6.1556E−015	2.014E−012	0.00021969
966	−1E−005	2.2E−005	2.2123	−7.6599E−014	2.971E−012	0.00029316
967	−0.00021	−0.00021	2.21	−4.1449E−013	5.428E−012	0.00047967
968	8.0E−005	0.000132	2.2069	1.5525E−013	7.683E−012	0.00062683
969	−0.00070	−0.00075	2.2015	−1.1411E−012	1.030E−011	0.0010461
970	0.0006067	0.000603	2.1948	−5.3056E−013	1.663E−011	0.001317
971	−0.00254	−0.00284	2.1815	−3.5018E−012	2.511E−011	0.0021758
972	0.0033302	0.002659	2.1668	−3.329E−012	3.697E−011	0.0025098
973	−0.01015	−0.01171	2.1343	−1.2191E−011	5.286E−011	0.0034728
974	0.020061	0.014108	2.1064	−2.119E−011	6.626E−011	0.0025652
975	−0.05310	−0.06027	2.0314	−4.0127E−011	1.724E−011	−0.000667
976	0.15176	0.10327	1.8677	−1.2675E−010	−3.6E−010	0.011648
977	−0.15411	−0.09376	2.6371	6.5367E−010	−1.0E−010	−0.37212
978	2.127	2.142	2.3845	−0.0058906	0.58041	−0.13816
979	1.9396	1.9293	2.1942	−0.017601	0.45971	−0.31364
980	2.0215	1.9757	2.281	−0.016543	0.31923	−0.49178
981	2.3204	2.2445	2.5891	0.00035693	0.14822	−0.68826
982	0.15843	−0.05350	2.1326	−0.00055212	−0.23496	0.10152
983	0.086993	0.072788	1.9834	−0.009555	0.078823	0.011411

984	−0.02898	−0.04364	2.1831	0.0017188	0.01464	−0.007067
985	0.010956	0.013716	2.235	−0.00090576	0.0029938	−0.002597
986	−0.00902	−0.00710	2.2375	−6.5806E−005	0.0016173	−0.004393
987	−0.00115	0.002649	2.2352	−0.00030426	−0.0008270	−0.002929
988	−0.00388	−0.00123	2.2306	−0.00013859	−0.0002905	−0.002642
989	−0.00165	0.000664	2.2269	−0.00014357	−0.0006086	−0.001808
990	−0.00179	−0.00022	2.224	−8.1867E−005	−0.0003384	−0.001392
991	−0.00098	0.000205	2.2219	−6.6889E−005	−0.0003154	−0.000957
992	−0.00083	−3E−005	2.2204	−4.1507E−005	−0.0002001	−0.000692
993	−0.00050	7.2E−005	2.2193	−3.082E−005	−0.0001534	−0.000477
994	−0.00038	2.4E−006	2.2186	−2.0101E−005	−0.0001032	−0.000335
995	−0.00024	2.8E−005	2.2181	−1.4252E−005	−7.3E−005	−0.000231
996	−0.00018	5.5E−006	2.2177	−9.5775E−006	−5.1E−005	−0.000160
997	−0.00012	1.1E−005	2.2175	−6.6378E−006	−3.5E−005	−0.000111
998	−8E−005	3.8E−006	2.2173	−4.5367E−006	−2.4E−005	−7.6E−005
999	−6E−005	4.9E−006	2.2172	−3.1104E−006	−1.7E−005	−5.3E−005
1000	−4E−005	2.1E−006	2.2171	−2.1446E−006	−1.2E−005	−3.6E−005
1001	−3E−005	2.2E−006	2.217	−1.4635E−006	−7.9E−006	−2.5E−005
1002	−2E−005	1.1E−006	2.217	−1.0132E−006	−5.5E−006	−1.7E−005
1003	−1E−005	9.8E−007	2.217	−6.9031E−007	−3.7E−006	−1.2E−005
1004	−9E−006	5.4E−007	2.2169	−4.7861E−007	−2.6E−006	−8.1E−006
1005	−6E−006	4.5E−007	2.2169	−3.2607E−007	−1.8E−006	−5.6E−006
1006	−4E−006	2.6E−007	2.2169	−2.261E−007	−1.2E−006	−3.8E−006
1007	−3E−006	2.1E−007	2.2169	−1.5414E−007	−8.4E−007	−2.6E−006
1008	−2E−006	1.3E−007	2.2169	−1.0683E−007	−5.9E−007	−1.8E−006
1009	−1E−006	9.8E−008	2.2169	−7.2893E−008	−4.0E−007	−1.3E−006
1010	−9E−007	6.1E−008	2.2169	−5.0483E−008	−2.8E−007	−8.6E−007
1011	−6E−007	4.6E−008	2.2169	−3.4478E−008	−1.9E−007	−5.9E−007
1012	−4E−007	2.9E−008	2.2169	−2.3859E−008	−1.3E−007	−4.1E−007
1013	−3E−007	2.1E−008	2.2169	−1.631E−008	−8.9E−008	−2.8E−007
1014	−2E−007	1.4E−008	2.2169	−1.1277E−008	−6.2E−008	−1.9E−007
1015	−1E−007	1.0E−008	2.2169	−7.7155E−009	−4.2E−008	−1.3E−007
1016	−1E−007	6.6E−009	2.2169	−5.3308E−009	−2.9E−008	−9.1E−008
1017	−7E−008	4.8E−009	2.2169	−3.6499E−009	−2.0E−008	−6.3E−008
1018	−5E−008	3.1E−009	2.2169	−2.5202E−009	−1.4E−008	−4.3E−008
1019	−3E−008	2.2E−009	2.2169	−1.7266E−009	−9.4E−009	−3.0E−008
1020	−2E−008	1.5E−009	2.2169	−1.1915E−009	−6.5E−009	−2.0E−008
1021	−1E−008	1.1E−009	2.2169	−8.1677E−010	−4.5E−009	−1.4E−008
1022	−1E−008	7.0E−010	2.2169	−5.6337E−010	−3.1E−009	−9.6E−009
1023	−7E−009	5.0E−010	2.2169	−3.8635E−010	−2.1E−009	−6.6E−009
1024	−5E−009	3.3E−010	2.2169	−2.6637E−010	−1.5E−009	−4.5E−009
1025	−3E−009	2.3E−010	2.2169	−1.8277E−010	−1.0E−009	−3.1E−009
1026	−2E−009	1.6E−010	2.2169	−1.2596E−010	−6.9E−010	−2.2E−009
1027	−2E−009	1.1E−010	2.2169	−8.6482E−011	−4.7E−010	−1.5E−009
1028	−1E−009	7.5E−011	2.2169	−5.9547E−011	−3.3E−010	−1.0E−009
1029	−7E−010	5.2E−011	2.2169	−4.0931E−011	−2.2E−010	−7E−010
1030	−5E−010	3.5E−011	2.2169	−2.8153E−011	−1.5E−010	−4.8E−010

1031	−4E−010	2.5E−011	2.2169	−1.9321E−011	−1.1E−010	−3.3E−010
1032	−2E−010	1.7E−011	2.2169	−1.3345E−011	−7.3E−011	−2.3E−010
1033	−2E−010	1.2E−011	2.2169	−9.1412E−012	−5.0E−011	−1.6E−010
1034	−1E−010	8.1E−012	2.2169	−6.3432E−012	−3.4E−011	−1.1E−010
1035	−8E−011	5.5E−012	2.2169	−4.3387E−012	−2.4E−011	−7.4E−011
1036	−5E−011	3.9E−012	2.2169	−2.9845E−012	−1.6E−011	−5.1E−011
1037	−4E−011	2.5E−012	2.2169	−2.0759E−012	−1.1E−011	−3.5E−011
1038	−3E−011	1.9E−012	2.2169	−1.3976E−012	−7.9E−012	−2.4E−011
1039	−2E−011	1.1E−012	2.2169	−9.875E−013	−5.5E−012	−1.7E−011
1040	−1E−011	6.7E−013	2.2169	−6.5912E−013	−3.4E−012	−1.1E−011
1041	−8E−012	5.4E−013	2.2169	−4.3642E−013	−2.7E−012	−7.8E−012
1042	−6E−012	3.8E−013	2.2169	−3.1125E−013	−1.5E−012	−5.4E−012
1043	−4E−012	2.0E−013	2.2169	−1.8277E−013	−1.1E−012	−3.8E−012
1044	−3E−012	−2E−014	2.2169	−1.491E−013	−5.8E−013	−2.7E−012
1045	−2E−012	−3E−013	2.2169	−9.027E−014	−6.9E−013	−1.7E−012
1046	−1E−012	3.6E−013	2.2169	−5.1492E−014	−2.9E−013	−1.0E−012
1047	−9E−013	1.6E−013	2.2169	−1.4234E−014	3.422E−013	−7.2E−013
1048	−5E−013	3.0E−013	2.2169	−1.41E−014	−3.2E−013	−1.9E−013
1049	−5E−013	1.3E−013	2.2169	−2.6112E−014	1.768E−013	−2.7E−013
1050	−5E−013	3.6E−015	2.2169	−7.8315E−014	−1.6E−013	3.42E−013
1051	−6E−013	−2E−013	2.2169	−1.8613E−015	1.639E−014	2.76E−013
1052	−6E−013	1.3E−014	2.2169	−4.2821E−014	5.452E−014	5.21E−013
1053	−8E−013	1.4E−013	2.2169	−3.1644E−014	5.600E−013	8.72E−013
1054	−1E−012	6.9E−014	2.2169	−5.1447E−014	2.266E−013	1.37E−012
1055	−2E−012	5.8E−014	2.2169	−1.6132E−013	6.877E−013	1.97E−012
1056	−3E−012	1.9E−013	2.2169	−1.5875E−013	1.352E−012	2.41E−012
1057	−4E−012	4.1E−013	2.2169	−2.4369E−013	1.466E−012	3.75E−012
1058	−6E−012	3.3E−013	2.2169	−2.9798E−013	9.497E−013	5.20E−012
1059	−8E−012	4.8E−013	2.2169	−5.3813E−013	2.515E−012	7.69E−012
1060	−1E−011	5.7E−013	2.2169	−6.6396E−013	3.605E−012	1.13E−011
1061	−2E−011	1.4E−012	2.2169	−8.9584E−013	5.392E−012	1.68E−011
1062	−3E−011	1.7E−012	2.2169	−1.4113E−012	7.793E−012	2.44E−011
1063	−4E−011	2.4E−012	2.2169	−2.0349E−012	1.142E−011	3.53E−011
1064	−5E−011	3.6E−012	2.2169	−3.0432E−012	1.596E−011	5.11E−011
1065	−8E−011	5.6E−012	2.2169	−4.335E−012	2.333E−011	7.38E−011
1066	−1E−010	8.1E−012	2.2169	−6.3171E−012	3.429E−011	1.08E−010
1067	−2E−010	1.2E−011	2.2169	−9.1219E−012	4.970E−011	1.57E−010
1068	−2E−010	1.7E−011	2.2169	−1.3398E−011	7.277E−011	2.27E−010
1069	−4E−010	2.5E−011	2.2169	−1.9298E−011	1.065E−010	3.31E−010
1070	−5E−010	3.6E−011	2.2169	−2.8151E−011	1.542E−010	4.81E−010
1071	−7E−010	5.3E−011	2.2169	−4.0905E−011	2.237E−010	7.00E−010
1072	−1E−009	7.4E−011	2.2169	−5.9545E−011	3.259E−010	1.02E−009
1073	−2E−009	1.1E−010	2.2169	−8.639E−011	4.722E−010	1.48E−009
1074	−2E−009	1.6E−010	2.2169	−1.2597E−010	6.894E−010	2.15E−009
1075	−3E−009	2.3E−010	2.2169	−1.8278E−010	9.979E−010	3.13E−009
1076	−5E−009	3.3E−010	2.2169	−2.6637E−010	1.458E−009	4.55E−009
1077	−7E−009	5.0E−010	2.2169	−3.864E−010	2.109E−009	6.62E−009

1078	−1E−008	7.0E−010	2.2169	−5.6333E−010	3.084E−009	9.62E−009
1079	−1E−008	1.1E−009	2.2169	−8.1679E−010	4.457E−009	1.40E−008
1080	−2E−008	1.5E−009	2.2169	−1.1915E−009	6.524E−009	2.03E−008
1081	−3E−008	2.2E−009	2.2169	−1.7265E−009	9.419E−009	2.96E−008
1082	−5E−008	3.1E−009	2.2169	−2.5201E−009	1.380E−008	4.30E−008
1083	−7E−008	4.8E−009	2.2169	−3.6499E−009	1.990E−008	6.26E−008
1084	−1E−007	6.6E−009	2.2169	−5.3309E−009	2.920E−008	9.09E−008
1085	−1E−007	1.0E−008	2.2169	−7.7156E−009	4.205E−008	1.32E−007
1086	−2E−007	1.4E−008	2.2169	−1.1277E−008	6.179E−008	1.92E−007
1087	−3E−007	2.1E−008	2.2169	−1.631E−008	8.884E−008	2.80E−007
1088	−4E−007	2.9E−008	2.2169	−2.3859E−008	1.308E−007	4.06E−007
1089	−6E−007	4.6E−008	2.2169	−3.4478E−008	1.876E−007	5.91E−007
1090	−9E−007	6.1E−008	2.2169	−5.0483E−008	2.767E−007	8.59E−007
1091	−1E−006	9.8E−008	2.2169	−7.2893E−008	3.963E−007	1.25E−006
1092	−2E−006	1.3E−007	2.2169	−1.0683E−007	5.855E−007	1.82E−006
1093	−3E−006	2.1E−007	2.2169	−1.5414E−007	8.365E−007	2.64E−006
1094	−4E−006	2.6E−007	2.2169	−2.261E−007	1.239E−006	3.84E−006
1095	−6E−006	4.5E−007	2.2169	−3.2607E−007	1.766E−006	5.59E−006
1096	−9E−006	5.4E−007	2.2169	−4.7861E−007	2.619E−006	8.11E−006
1097	−1E−005	9.8E−007	2.217	−6.9031E−007	3.725E−006	1.18E−005
1098	−2E−005	1.1E−006	2.217	−1.0132E−006	5.529E−006	1.71E−005
1099	−3E−005	2.2E−006	2.217	−1.4635E−006	7.856E−006	2.50E−005
1100	−4E−005	2.1E−006	2.2171	−2.1446E−006	1.165E−005	3.62E−005
1101	−6E−005	4.9E−006	2.2172	−3.1104E−006	1.656E−005	5.26E−005
1102	−8E−005	3.8E−006	2.2173	−4.5367E−006	2.442E−005	7.63E−005
1103	−0.00012	1.1E−005	2.2175	−6.6378E−006	3.488E−005	0.00011066
1104	−0.00018	5.5E−006	2.2177	−9.5775E−006	5.070E−005	0.00016042
1105	−0.00024	2.8E−005	2.2181	−1.4252E−005	7.334E−005	0.00023124
1106	−0.00038	2.4E−006	2.2186	−2.0101E−005	0.00010317	0.00033535
1107	−0.00050	7.2E−005	2.2193	−3.082E−005	0.0001534	0.000477
1108	−0.00083	−3E−005	2.2204	−4.1507E−005	0.0002001	0.00069239
1109	−0.00098	0.000205	2.2219	−6.6889E−005	0.00031539	0.00095734
1110	−0.00179	−0.00022	2.224	−8.1867E−005	0.00033844	0.0013924
1111	−0.00165	0.000664	2.2269	−0.00014357	0.00060863	0.0018082
1112	−0.00388	−0.00123	2.2306	−0.00013859	0.00029046	0.0026419
1113	−0.00115	0.002649	2.2352	−0.00030426	0.00082705	0.0029294
1114	−0.00902	−0.00710	2.2375	−6.5806E−005	−0.0016173	0.004393
1115	0.010956	0.013716	2.235	−0.00090576	−0.0029938	0.0025968
1116	−0.02898	−0.04364	2.1831	0.0017188	−0.01464	0.0070673
1117	0.086993	0.072788	1.9834	−0.009555	−0.078823	−0.011411
1118	0.15843	−0.05350	2.1326	−0.00055212	0.23496	−0.10152
1119	0.23633	0.064305	2.1736	0.0016231	−0.19118	0.19831
1120	0.067165	0.024389	2.0678	−0.0096304	0.067023	0.028917
1121	−0.01675	−0.03145	2.1522	−0.0017944	0.02513	−0.002077
1122	0.0068781	0.005558	2.2062	−0.00085584	0.005728	−0.007526
1123	−0.00702	−0.00539	2.2127	−0.00078072	0.0009545	−0.008078
1124	−0.00054	0.001760	2.2204	−0.00066706	−0.0008042	−0.006352

1125	−0.00317	−0.00105	2.2186	−0.00044173	−0.0009840	−0.005064
1126	−0.00097	0.000618	2.2194	−0.00036042	−0.0009148	−0.003623
1127	−0.00139	−0.00018	2.2183	−0.00022315	−0.0006879	−0.002644
1128	−0.00060	0.000222	2.2181	−0.00017179	−0.0005185	−0.001843
1129	−0.00061	−1E−005	2.2176	−0.000107	−0.0003632	−0.001301
1130	−0.00032	8.3E−005	2.2175	−7.9334E−005	−0.0002599	−0.000898
1131	−0.00027	1.3E−005	2.2173	−5.0477E−005	−0.0001788	−0.000625
1132	−0.00016	3.3E−005	2.2172	−3.6569E−005	−0.0001256	−0.000430
1133	−0.00012	1.1E−005	2.2171	−2.3753E−005	−8.6E−005	−0.000298
1134	−8E−005	1.4E−005	2.217	−1.6954E−005	−6.0E−005	−0.000205
1135	−6E−005	6.4E−006	2.217	−1.1193E−005	−4.1E−005	−0.000141
1136	−4E−005	5.9E−006	2.217	−7.9092E−006	−2.8E−005	−9.7E−005
1137	−3E−005	3.4E−006	2.2169	−5.283E−006	−2.0E−005	−6.7E−005
1138	−2E−005	2.7E−006	2.2169	−3.707E−006	−1.4E−005	−4.6E−005
1139	−1E−005	1.7E−006	2.2169	−2.4964E−006	−9.3E−006	−3.2E−005
1140	−8E−006	1.2E−006	2.2169	−1.743E−006	−6.4E−006	−2.2E−005
1141	−6E−006	8.2E−007	2.2169	−1.1804E−006	−4.4E−006	−1.5E−005
1142	−4E−006	5.7E−007	2.2169	−8.212E−007	−3.0E−006	−1.0E−005
1143	−3E−006	3.9E−007	2.2169	−5.583E−007	−2.1E−006	−7.1E−006
1144	−2E−006	2.7E−007	2.2169	−3.874E−007	−1.4E−006	−4.9E−006
1145	−1E−006	1.9E−007	2.2169	−2.641E−007	−9.8E−007	−3.3E−006
1146	−9E−007	1.3E−007	2.2169	−1.829E−007	−6.8E−007	−2.3E−006
1147	−6E−007	8.9E−008	2.2169	−1.2493E−007	−4.7E−007	−1.6E−006
1148	−4E−007	6.0E−008	2.2169	−8.6401E−008	−3.2E−007	−1.1E−006
1149	−3E−007	4.2E−008	2.2169	−5.91E−008	−2.2E−007	−7.5E−007
1150	−2E−007	2.8E−008	2.2169	−4.0828E−008	−1.5E−007	−5.1E−007
1151	−1E−007	2.0E−008	2.2169	−2.7957E−008	−1.0E−007	−3.5E−007
1152	−9E−008	1.3E−008	2.2169	−1.9298E−008	−7.2E−008	−2.4E−007
1153	−6E−008	9.4E−009	2.2169	−1.3225E−008	−4.9E−008	−1.7E−007
1154	−4E−008	6.3E−009	2.2169	−9.1225E−009	−3.4E−008	−1.2E−007
1155	−3E−008	4.4E−009	2.2169	−6.2556E−009	−2.3E−008	−7.9E−008
1156	−2E−008	3.0E−009	2.2169	−4.313E−009	−1.6E−008	−5.4E−008
1157	−1E−008	2.1E−009	2.2169	−2.959E−009	−1.1E−008	−3.7E−008
1158	−1E−008	1.4E−009	2.2169	−2.0393E−009	−7.6E−009	−2.6E−008
1159	−7E−009	9.9E−010	2.2169	−1.3996E−009	−5.2E−009	−1.8E−008
1160	−5E−009	6.7E−010	2.2169	−9.6425E−010	−3.6E−009	−1.2E−008
1161	−3E−009	4.7E−010	2.2169	−6.6199E−010	−2.5E−009	−8.4E−009
1162	−2E−009	3.2E−010	2.2169	−4.5599E−010	−1.7E−009	−5.8E−009
1163	−2E−009	2.2E−010	2.2169	−3.1313E−010	−1.2E−009	−4.0E−009
1164	−1E−009	1.5E−010	2.2169	−2.1562E−010	−8.1E−010	−2.7E−009
1165	−7E−010	1.0E−010	2.2169	−1.4811E−010	−5.5E−010	−1.9E−009
1166	−5E−010	7.1E−011	2.2169	−1.0199E−010	−3.8E−010	−1.3E−009
1167	−3E−010	4.9E−011	2.2169	−7.0031E−011	−2.6E−010	−8.9E−010
1168	−2E−010	3.3E−011	2.2169	−4.8198E−011	−1.8E−010	−6.1E−010
1169	−2E−010	2.3E−011	2.2169	−3.3185E−011	−1.2E−010	−4.2E−010
1170	−1E−010	1.6E−011	2.2169	−2.2766E−011	−8.5E−011	−2.9E−010
1171	−8E−011	1.1E−011	2.2169	−1.5715E−011	−5.9E−011	−2.0E−010

1172	−5E−011	7.3E−012	2.2169	−1.0796E−011	−4.0E−011	−1.4E−010
1173	−4E−011	5.3E−012	2.2169	−7.456E−012	−2.8E−011	−9.4E−011
1174	−2E−011	3.5E−012	2.2169	−5.1796E−012	−1.9E−011	−6.5E−011
1175	−2E−011	2.4E−012	2.2169	−3.4658E−012	−1.3E−011	−4.4E−011
1176	−1E−011	1.6E−012	2.2169	−2.4359E−012	−8.9E−012	−3.0E−011
1177	−8E−012	7.6E−013	2.2169	−1.6873E−012	−6.3E−012	−2.1E−011
1178	−5E−012	8.5E−013	2.2169	−1.1436E−012	−4.2E−012	−1.4E−011
1179	−4E−012	5.3E−013	2.2169	−7.6633E−013	−2.9E−012	−1.0E−011
1180	−3E−012	4.8E−013	2.2169	−4.8716E−013	−2.2E−012	−6.8E−012
1181	−2E−012	3.7E−013	2.2169	−3.9234E−013	−1.5E−012	−4.7E−012
1182	−1E−012	2.8E−013	2.2169	−2.3418E−013	−1.1E−012	−3.1E−012
1183	−5E−013	3.7E−013	2.2169	−1.8601E−013	−7.5E−013	−2.1E−012
1184	−9E−013	−2E−013	2.2169	−1.2604E−013	−1.7E−013	−1.3E−012
1185	−6E−013	−1E−013	2.2169	−1.1557E−013	−3.2E−013	−1.1E−012
1186	−6E−013	−3E−013	2.2169	−8.9143E−014	−8.9E−014	−4.4E−013
1187	−4E−013	1.9E−013	2.2169	3.2739E−014	−4.3E−013	1.10E−013
1188	−6E−013	−2E−013	2.2169	−1.2309E−013	2.736E−013	4.81E−013
1189	−6E−013	8.9E−016	2.2169	−1.5948E−014	1.428E−013	8.18E−013
1190	−1E−012	−3E−013	2.2169	−7.2928E−014	5.914E−013	1.53E−012
1191	−9E−013	−4E−014	2.2169	−1.2569E−013	2.973E−013	2.17E−012
1192	−1E−012	3.0E−013	2.2169	−2.8591E−013	8.004E−013	3.23E−012
1193	−2E−012	2.1E−013	2.2169	−4.45E−013	1.522E−012	4.44E−012
1194	−3E−012	2.0E−013	2.2169	−6.0097E−013	1.9E−012	6.57E−012
1195	−4E−012	5.7E−013	2.2169	−7.2808E−013	2.885E−012	9.66E−012
1196	−6E−012	8.2E−013	2.2169	−1.1385E−012	4.222E−012	1.44E−011
1197	−8E−012	1.1E−012	2.2169	−1.5931E−012	6.138E−012	2.13E−011
1198	−1E−011	1.9E−012	2.2169	−2.3864E−012	9.350E−012	3.09E−011
1199	−2E−011	2.4E−012	2.2169	−3.5044E−012	1.328E−011	4.43E−011
1200	−2E−011	3.6E−012	2.2169	−5.1876E−012	1.912E−011	6.45E−011
1201	−4E−011	4.8E−012	2.2169	−7.4032E−012	2.743E−011	9.35E−011
1202	−5E−011	7.6E−012	2.2169	−1.0784E−011	4.044E−011	1.36E−010
1203	−8E−011	1.1E−011	2.2169	−1.5632E−011	5.853E−011	1.98E−010
1204	−1E−010	1.5E−011	2.2169	−2.2806E−011	8.539E−011	2.88E−010
1205	−2E−010	2.3E−011	2.2169	−3.3187E−011	1.237E−010	4.19E−010
1206	−2E−010	3.4E−011	2.2169	−4.8132E−011	1.805E−010	6.10E−010
1207	−3E−010	4.9E−011	2.2169	−7.0096E−011	2.619E−010	8.87E−010
1208	−5E−010	7.1E−011	2.2169	−1.0191E−010	3.808E−010	1.29E−009
1209	−7E−010	1.0E−010	2.2169	−1.4816E−010	5.542E−010	1.87E−009
1210	−1E−009	1.5E−010	2.2169	−2.1569E−010	8.059E−010	2.72E−009
1211	−2E−009	2.2E−010	2.2169	−3.1309E−010	1.171E−009	3.96E−009
1212	−2E−009	3.2E−010	2.2169	−4.5596E−010	1.703E−009	5.76E−009
1213	−3E−009	4.7E−010	2.2169	−6.6216E−010	2.475E−009	8.38E−009
1214	−5E−009	6.7E−010	2.2169	−9.6433E−010	3.600E−009	1.22E−008
1215	−7E−009	9.9E−010	2.2169	−1.3996E−009	5.232E−009	1.77E−008
1216	−1E−008	1.4E−009	2.2169	−2.0392E−009	7.611E−009	2.57E−008
1217	−1E−008	2.1E−009	2.2169	−2.9591E−009	1.106E−008	3.74E−008
1218	−2E−008	3.0E−009	2.2169	−4.313E−009	1.609E−008	5.44E−008

1219	−3E−008	4.4E−009	2.2169	−6.2555E−009	2.337E−008	7.92E−008
1220	−4E−008	6.3E−009	2.2169	−9.1225E−009	3.401E−008	1.15E−007
1221	−6E−008	9.4E−009	2.2169	−1.3225E−008	4.94E−008	1.67E−007
1222	−9E−008	1.3E−008	2.2169	−1.9298E−008	7.189E−008	2.43E−007
1223	−1E−007	2.0E−008	2.2169	−2.7957E−008	1.044E−007	3.54E−007
1224	−2E−007	2.8E−008	2.2169	−4.0828E−008	1.520E−007	5.14E−007
1225	−3E−007	4.2E−008	2.2169	−5.91E−008	2.206E−007	7.48E−007
1226	−4E−007	6.0E−008	2.2169	−8.6401E−008	3.212E−007	1.09E−006
1227	−6E−007	8.9E−008	2.2169	−1.2493E−007	4.661E−007	1.58E−006
1228	−9E−007	1.3E−007	2.2169	−1.829E−007	6.788E−007	2.30E−006
1229	−1E−006	1.9E−007	2.2169	−2.641E−007	9.847E−007	3.35E−006
1230	−2E−006	2.7E−007	2.2169	−3.874E−007	1.434E−006	4.86E−006
1231	−3E−006	3.9E−007	2.2169	−5.583E−007	2.080E−006	7.07E−006
1232	−4E−006	5.7E−007	2.2169	−8.212E−007	3.030E−006	1.03E−005
1233	−6E−006	8.2E−007	2.2169	−1.1804E−006	4.390E−006	1.50E−005
1234	−8E−006	1.2E−006	2.2169	−1.743E−006	6.399E−006	2.17E−005
1235	−1E−005	1.7E−006	2.2169	−2.4964E−006	9.26E−006	3.16E−005
1236	−2E−005	2.7E−006	2.2169	−3.707E−006	1.351E−005	4.59E−005
1237	−3E−005	3.4E−006	2.2169	−5.283E−006	1.951E−005	6.68E−005
1238	−4E−005	5.9E−006	2.217	−7.9092E−006	2.848E−005	9.70E−005
1239	−6E−005	6.4E−006	2.217	−1.1193E−005	4.105E−005	0.00014114
1240	−8E−005	1.4E−005	2.217	−1.6954E−005	5.995E−005	0.00020459
1241	−0.00012	1.1E−005	2.2171	−2.3753E−005	8.603E−005	0.0002976
1242	−0.00016	3.3E−005	2.2172	−3.6569E−005	0.00012559	0.00043017
1243	−0.00027	1.3E−005	2.2173	−5.0477E−005	0.00017879	0.00062507
1244	−0.00032	8.3E−005	2.2175	−7.9334E−005	0.00025988	0.00089798
1245	−0.00061	−1E−005	2.2176	−0.000107	0.00036315	0.0013006
1246	−0.00060	0.000222	2.2181	−0.00017179	0.00051852	0.0018426
1247	−0.00139	−0.00018	2.2183	−0.00022315	0.00068788	0.0026441
1248	−0.00097	0.000618	2.2194	−0.00036042	0.00091479	0.0036227
1249	−0.00317	−0.00105	2.2186	−0.00044173	0.00098396	0.0050638
1250	−0.00054	0.001760	2.2204	−0.00066706	0.00080425	0.0063515
1251	−0.00702	−0.00539	2.2127	−0.00078072	−0.0009545	0.0080781
1252	0.0068781	0.005558	2.2062	−0.00085584	−0.005728	0.0075257
1253	−0.01675	−0.03145	2.1522	−0.0017944	−0.02513	0.0020775
1254	0.067165	0.024389	2.0678	−0.0096304	−0.067023	−0.028917
1255	0.23633	0.064305	2.1736	0.0016231	0.19118	−0.19831
1256	0.21345	0.081784	2.2513	0.011031	−0.13622	0.29124
1257	0.060004	0.015744	2.0938	−0.010971	0.049702	0.032701
1258	−0.01477	−0.03114	2.135	−0.0029355	0.01726	−0.002262
1259	0.0087819	0.004722	2.1721	−0.0015101	0.0043947	−0.008668
1260	−0.00527	−0.00576	2.184	−0.00088875	0.00039957	−0.009148
1261	0.0005965	0.001551	2.197	−0.00070191	−0.0012801	−0.007319
1262	−0.00218	−0.00128	2.2022	−0.00040918	−0.001229	−0.005514
1263	−0.00037	0.000511	2.2074	−0.00030054	−0.0011464	−0.003920
1264	−0.00090	−0.00031	2.21	−0.00017987	−0.0008189	−0.002763
1265	−0.00030	0.000164	2.2123	−0.0001281	−0.0006232	−0.001906

1266	−0.00038	−7E−005	2.2136	−7.9421E−005	−0.0004259	−0.001320
1267	−0.00017	5.3E−005	2.2147	−5.582E−005	−0.0003057	−0.000904
1268	−0.00016	−2E−005	2.2154	−3.5786E−005	−0.0002076	−0.000623
1269	−9E−005	1.7E−005	2.2158	−2.4969E−005	−0.0001458	−0.000427
1270	−7E−005	−3E−006	2.2162	−1.643E−005	−9.9E−005	−0.000294
1271	−4E−005	6.1E−006	2.2164	−1.1404E−005	−6.9E−005	−0.000201
1272	−3E−005	−2E−007	2.2166	−7.6398E−006	−4.7E−005	−0.000138
1273	−2E−005	2.3E−006	2.2167	−5.2819E−006	−3.3E−005	−9.5E−005
1274	−2E−005	2.8E−007	2.2167	−3.5795E−006	−2.2E−005	−6.5E−005
1275	−1E−005	9.0E−007	2.2168	−2.4675E−006	−1.5E−005	−4.5E−005
1276	−7E−006	2.4E−007	2.2168	−1.6842E−006	−1.1E−005	−3.1E−005
1277	−5E−006	3.8E−007	2.2169	−1.1587E−006	−7.3E−006	−2.1E−005
1278	−3E−006	1.5E−007	2.2169	−7.9419E−007	−5.0E−006	−1.5E−005
1279	−2E−006	1.6E−007	2.2169	−5.4571E−007	−3.5E−006	−1E−005
1280	−2E−006	7.8E−008	2.2169	−3.7496E−007	−2.4E−006	−6.9E−006
1281	−1E−006	7.4E−008	2.2169	−2.5746E−007	−1.6E−006	−4.7E−006
1282	−7E−007	4E−008	2.2169	−1.7714E−007	−1.1E−006	−3.3E−006
1283	−5E−007	3.4E−008	2.2169	−1.2159E−007	−7.8E−007	−2.2E−006
1284	−4E−007	2.0E−008	2.2169	−8.3717E−008	−5.3E−007	−1.5E−006
1285	−2E−007	1.6E−008	2.2169	−5.7458E−008	−3.7E−007	−1.1E−006
1286	−2E−007	9.6E−009	2.2169	−3.9573E−008	−2.5E−007	−7.3E−007
1287	−1E−007	7.3E−009	2.2169	−2.7162E−008	−1.7E−007	−5.0E−007
1288	−8E−008	4.6E−009	2.2169	−1.8709E−008	−1.2E−007	−3.5E−007
1289	−5E−008	3.4E−009	2.2169	−1.2843E−008	−8.2E−008	−2.4E−007
1290	−4E−008	2.2E−009	2.2169	−8.8455E−009	−5.6E−008	−1.6E−007
1291	−3E−008	1.6E−009	2.2169	−6.0733E−009	−3.9E−008	−1.1E−007
1292	−2E−008	1.1E−009	2.2169	−4.1825E−009	−2.7E−008	−7.7E−008
1293	−1E−008	7.6E−010	2.2169	−2.8722E−009	−1.8E−008	−5.3E−008
1294	−8E−009	5.1E−010	2.2169	−1.9778E−009	−1.3E−008	−3.7E−008
1295	−6E−009	3.6E−010	2.2169	−1.3584E−009	−8.7E−009	−2.5E−008
1296	−4E−009	2.4E−010	2.2169	−9.3522E−010	−6.0E−009	−1.7E−008
1297	−3E−009	1.7E−010	2.2169	−6.4245E−010	−4.1E−009	−1.2E−008
1298	−2E−009	1.1E−010	2.2169	−4.423E−010	−2.8E−009	−8.2E−009
1299	−1E−009	8.0E−011	2.2169	−3.0386E−010	−1.9E−009	−5.6E−009
1300	−9E−010	5.4E−011	2.2169	−2.0919E−010	−1.3E−009	−3.9E−009
1301	−6E−010	3.8E−011	2.2169	−1.4371E−010	−9.2E−010	−2.7E−009
1302	−4E−010	2.6E−011	2.2169	−9.8897E−011	−6.3E−010	−1.8E−009
1303	−3E−010	1.8E−011	2.2169	−6.7953E−011	−4.4E−010	−1.3E−009
1304	−2E−010	1.3E−011	2.2169	−4.6842E−011	−3.0E−010	−8.6E−010
1305	−1E−010	8.5E−012	2.2169	−3.2198E−011	−2.1E−010	−5.9E−010
1306	−9E−011	5.9E−012	2.2169	−2.2192E−011	−1.4E−010	−4.1E−010
1307	−6E−011	4.2E−012	2.2169	−1.5271E−011	−9.8E−011	−2.8E−010
1308	−4E−011	2.5E−012	2.2169	−1.044E−011	−6.7E−011	−1.9E−010
1309	−3E−011	1.9E−012	2.2169	−7.2272E−012	−4.6E−011	−1.3E−010
1310	−2E−011	1.4E−012	2.2169	−4.9547E−012	−3.2E−011	−9.2E−011
1311	−1E−011	6.0E−013	2.2169	−3.3844E−012	−2.2E−011	−6.3E−011
1312	−1E−011	3.6E−013	2.2169	−2.342E−012	−1.5E−011	−4.3E−011

1313	−7E−012	4.1E−013	2.2169	−1.6475E−012	−1.0E−011	−3.0E−011
1314	−5E−012	2.5E−013	2.2169	−1.1443E−012	−7.2E−012	−2.0E−011
1315	−3E−012	1.7E−013	2.2169	−8.2517E−013	−5.0E−012	−1.4E−011
1316	−2E−012	3.3E−013	2.2169	−5.692E−013	−3.3E−012	−9.8E−012
1317	−2E−012	7.5E−014	2.2169	−3.3788E−013	−2.3E−012	−6.7E−012
1318	−1E−012	−8E−014	2.2169	−2.3007E−013	−1.5E−012	−4.6E−012
1319	−7E−013	1.8E−013	2.2169	−1.9105E−013	−9.7E−013	−2.9E−012
1320	−9E−013	−2E−013	2.2169	−1.2384E−013	−4.9E−013	−2.1E−012
1321	−6E−013	−4E−013	2.2169	−5.2362E−014	−7.2E−013	−1.1E−012
1322	−1E−013	1.7E−013	2.2169	−5.0297E−014	−3E−013	−7.4E−013
1323	−2E−013	8.3E−014	2.2169	−1.7791E−014	−1.6E−013	−2.1E−013
1324	−5E−013	−3E−013	2.2169	−3.9173E−014	−1.6E−013	−2E−013
1325	−5E−013	1.4E−014	2.2169	2.0511E−014	1.255E−013	4.38E−013
1326	1.6E−013	3.2E−013	2.2169	−6.4856E−014	1.992E−013	8.88E−013
1327	−3E−013	3.0E−014	2.2169	−1.3445E−013	6.119E−013	1.68E−012
1328	−3E−013	2.9E−013	2.2169	−1.0453E−013	6.333E−013	1.80E−012
1329	−9E−013	−6E−014	2.2169	−1.5392E−013	1.256E−012	3.22E−012
1330	−9E−013	1.1E−013	2.2169	−2.1991E−013	1.766E−012	4.33E−012
1331	−2E−012	8.8E−014	2.2169	−3.2954E−013	2.224E−012	6.61E−012
1332	−2E−012	−9E−014	2.2169	−5.4689E−013	3.346E−012	9.76E−012
1333	−3E−012	4.6E−013	2.2169	−7.2776E−013	4.822E−012	1.40E−011
1334	−5E−012	1.9E−015	2.2169	−1.1098E−012	7.282E−012	2.06E−011
1335	−7E−012	3.8E−013	2.2169	−1.5511E−012	1.034E−011	2.99E−011
1336	−1E−011	7.9E−013	2.2169	−2.3399E−012	1.520E−011	4.34E−011
1337	−1E−011	4.8E−013	2.2169	−3.3496E−012	2.205E−011	6.33E−011
1338	−2E−011	1.3E−012	2.2169	−5.0061E−012	3.151E−011	9.12E−011
1339	−3E−011	1.9E−012	2.2169	−7.1925E−012	4.607E−011	1.33E−010
1340	−4E−011	2.9E−012	2.2169	−1.0528E−011	6.702E−011	1.93E−010
1341	−6E−011	4.2E−012	2.2169	−1.5212E−011	9.782E−011	2.81E−010
1342	−9E−011	6E−012	2.2169	−2.2089E−011	1.419E−010	4.09E−010
1343	−1E−010	8.2E−012	2.2169	−3.2169E−011	2.062E−010	5.94E−010
1344	−2E−010	1.2E−011	2.2169	−4.686E−011	2.998E−010	8.64E−010
1345	−3E−010	1.8E−011	2.2169	−6.7986E−011	4.362E−010	1.26E−009
1346	−4E−010	2.6E−011	2.2169	−9.8914E−011	6.333E−010	1.83E−009
1347	−6E−010	3.8E−011	2.2169	−1.4385E−010	9.219E−010	2.66E−009
1348	−9E−010	5.4E−011	2.2169	−2.0926E−010	1.339E−009	3.86E−009
1349	−1E−009	8.0E−011	2.2169	−3.0387E−010	1.948E−009	5.62E−009
1350	−2E−009	1.1E−010	2.2169	−4.4233E−010	2.830E−009	8.17E−009
1351	−3E−009	1.7E−010	2.2169	−6.4253E−010	4.117E−009	1.19E−008
1352	−4E−009	2.4E−010	2.2169	−9.3536E−010	5.982E−009	1.73E−008
1353	−6E−009	3.6E−010	2.2169	−1.3584E−009	8.703E−009	2.51E−008
1354	−8E−009	5.1E−010	2.2169	−1.9777E−009	1.264E−008	3.65E−008
1355	−1E−008	7.6E−010	2.2169	−2.8723E−009	1.839E−008	5.31E−008
1356	−2E−008	1.1E−009	2.2169	−4.1825E−009	2.672E−008	7.72E−008
1357	−3E−008	1.6E−009	2.2169	−6.0732E−009	3.888E−008	1.12E−007
1358	−4E−008	2.2E−009	2.2169	−8.8456E−009	5.646E−008	1.63E−007
1359	−5E−008	3.4E−009	2.2169	−1.2843E−008	8.217E−008	2.37E−007

1360	−8E−008	4.6E−009	2.2169	−1.8709E−008	1.193E−007	3.45E−007
1361	−1E−007	7.3E−009	2.2169	−2.7162E−008	1.737E−007	5.02E−007
1362	−2E−007	9.6E−009	2.2169	−3.9573E−008	2.521E−007	7.30E−007
1363	−2E−007	1.6E−008	2.2169	−5.7458E−008	3.670E−007	1.06E−006
1364	−4E−007	2.0E−008	2.2169	−8.3717E−008	5.326E−007	1.54E−006
1365	−5E−007	3.4E−008	2.2169	−1.2159E−007	7.756E−007	2.24E−006
1366	−7E−007	4E−008	2.2169	−1.7714E−007	1.125E−006	3.26E−006
1367	−1E−006	7.4E−008	2.2169	−2.5746E−007	1.639E−006	4.74E−006
1368	−2E−006	7.8E−008	2.2169	−3.7496E−007	2.376E−006	6.90E−006
1369	−2E−006	1.6E−007	2.2169	−5.4571E−007	3.463E−006	1.00E−005
1370	−3E−006	1.5E−007	2.2169	−7.9419E−007	5.017E−006	1.46E−005
1371	−5E−006	3.8E−007	2.2169	−1.1587E−006	7.317E−006	2.12E−005
1372	−7E−006	2.4E−007	2.2168	−1.6842E−006	1.059E−005	3.09E−005
1373	−1E−005	9.0E−007	2.2168	−2.4675E−006	1.546E−005	4.49E−005
1374	−2E−005	2.8E−007	2.2167	−3.5795E−006	2.235E−005	6.54E−005
1375	−2E−005	2.3E−006	2.2167	−5.2819E−006	3.268E−005	9.50E−005
1376	−3E−005	−2E−007	2.2166	−7.6398E−006	4.713E−005	0.00013849
1377	−4E−005	6.1E−006	2.2164	−1.1404E−005	6.908E−005	0.00020122
1378	−7E−005	−3E−006	2.2162	−1.643E−005	9.920E−005	0.0002936
1379	−9E−005	1.7E−005	2.2158	−2.4969E−005	0.00014583	0.00042652
1380	−0.00016	−2E−005	2.2154	−3.5786E−005	0.00020756	0.00062293
1381	−0.00017	5.3E−005	2.2147	−5.582E−005	0.0003057	0.00090401
1382	−0.00038	−7E−005	2.2136	−7.9421E−005	0.00042585	0.0013197
1383	−0.00030	0.000164	2.2123	−0.0001281	0.00062315	0.0019061
1384	−0.00090	−0.00031	2.21	−0.00017987	0.00081889	0.002763
1385	−0.00037	0.000511	2.2074	−0.00030054	0.0011464	0.0039202
1386	−0.00218	−0.00128	2.2022	−0.00040918	0.001229	0.0055141
1387	0.0005965	0.001551	2.197	−0.00070191	0.0012801	0.0073186
1388	−0.00527	−0.00576	2.184	−0.00088875	−0.0003996	0.0091478
1389	0.0087819	0.004722	2.1721	−0.0015101	−0.0043947	0.008668
1390	−0.01477	−0.03114	2.135	−0.0029355	−0.01726	0.0022618
1391	0.060004	0.015744	2.0938	−0.010971	−0.049702	−0.032701
1392	0.21345	0.081784	2.2513	0.011031	0.13622	−0.29124
1393	0.030674	−0.01169	2.3885	0.0163	−0.079372	0.36935
1394	0.094931	0.050735	2.0936	−0.00918	0.019493	0.007808
1395	−0.02320	−0.03345	2.0938	−0.0017863	0.0098895	−0.003150
1396	0.01118	0.005256	2.1276	−0.00074121	0.0016888	−0.005374
1397	−0.00450	−0.00571	2.1536	−0.00040646	0.00021518	−0.006170
1398	0.0011773	0.000688	2.1732	−0.00023895	−0.0008768	−0.004604
1399	−0.00146	−0.00135	2.1872	−0.000133	−0.0007022	−0.003553
1400	−2E−005	0.000109	2.1967	−8.2683E−005	−0.0006737	−0.002397
1401	−0.00055	−0.00038	2.2032	−4.625E−005	−0.0004681	−0.001701
1402	−0.00012	3.2E−006	2.2076	−3.0979E−005	−0.0003539	−0.001139
1403	−0.00022	−0.00012	2.2106	−1.7394E−005	−0.0002425	−0.000787
1404	−8E−005	−1E−005	2.2126	−1.2454E−005	−0.0001709	−0.000531
1405	−9E−005	−4E−005	2.214	−7.1095E−006	−0.0001177	−0.000364
1406	−4E−005	−1E−005	2.2149	−5.3103E−006	−8.1E−005	−0.000248

1407	−4E−005	−2E−005	2.2155	−3.1017E−006	−5.6E−005	−0.000170
1408	−2E−005	−6E−006	2.216	−2.3583E−006	−3.8E−005	−0.000116
1409	−2E−005	−6E−006	2.2163	−1.41E−006	−2.7E−005	−8.0E−005
1410	−1E−005	−3E−006	2.2165	−1.0737E−006	−1.8E−005	−5.5E−005
1411	−8E−006	−3E−006	2.2166	−6.5557E−007	−1.3E−005	−3.8E−005
1412	−5E−006	−1E−006	2.2167	−4.9583E−007	−8.5E−006	−2.6E−005
1413	−4E−006	−1E−006	2.2168	−3.0829E−007	−5.9E−006	−1.8E−005
1414	−2E−006	−7E−007	2.2168	−2.3084E−007	−4.0E−006	−1.2E−005
1415	−2E−006	−5E−007	2.2168	−1.4576E−007	−2.8E−006	−8.4E−006
1416	−1E−006	−3E−007	2.2169	−1.0799E−007	−1.9E−006	−5.8E−006
1417	−8E−007	−3E−007	2.2169	−6.9082E−008	−1.3E−006	−4.0E−006
1418	−5E−007	−2E−007	2.2169	−5.0664E−008	−9.1E−007	−2.7E−006
1419	−4E−007	−1E−007	2.2169	−3.277E−008	−6.3E−007	−1.9E−006
1420	−3E−007	−8E−008	2.2169	−2.3815E−008	−4.3E−007	−1.3E−006
1421	−2E−007	−5E−008	2.2169	−1.5548E−008	−3.0E−007	−8.8E−007
1422	−1E−007	−4E−008	2.2169	−1.121E−008	−2.0E−007	−6.1E−007
1423	−8E−008	−3E−008	2.2169	−7.3763E−009	−1.4E−007	−4.2E−007
1424	−6E−008	−2E−008	2.2169	−5.2813E−009	−9.6E−008	−2.9E−007
1425	−4E−008	−1E−008	2.2169	−3.4985E−009	−6.7E−008	−2.0E−007
1426	−3E−008	−8E−009	2.2169	−2.49E−009	−4.6E−008	−1.4E−007
1427	−2E−008	−6E−009	2.2169	−1.6588E−009	−3.1E−008	−9.3E−008
1428	−1E−008	−4E−009	2.2169	−1.1747E−009	−2.2E−008	−6.4E−008
1429	−9E−009	−3E−009	2.2169	−7.863E−010	−1.5E−008	−4.4E−008
1430	−6E−009	−2E−009	2.2169	−5.5439E−010	−1.0E−008	−3.0E−008
1431	−4E−009	−1E−009	2.2169	−3.7262E−010	−7.0E−009	−2.1E−008
1432	−3E−009	−9E−010	2.2169	−2.6175E−010	−4.8E−009	−1.4E−008
1433	−2E−009	−6E−010	2.2169	−1.7647E−010	−3.3E−009	−9.9E−009
1434	−1E−009	−4E−010	2.2169	−1.2359E−010	−2.3E−009	−6.8E−009
1435	−9E−010	−3E−010	2.2169	−8.3589E−011	−1.6E−009	−4.7E−009
1436	−6E−010	−2E−010	2.2169	−5.8345E−011	−1.1E−009	−3.2E−009
1437	−4E−010	−1E−010	2.2169	−3.9578E−011	−7.5E−010	−2.2E−009
1438	−3E−010	−9E−011	2.2169	−2.7634E−011	−5.1E−010	−1.5E−009
1439	−2E−010	−6E−011	2.2169	−1.8759E−011	−3.5E−010	−1.0E−009
1440	−1E−010	−4E−011	2.2169	−1.3024E−011	−2.4E−010	−7.2E−010
1441	−1E−010	−3E−011	2.2169	−8.8987E−012	−1.7E−010	−4.9E−010
1442	−7E−011	−2E−011	2.2169	−6.2422E−012	−1.1E−010	−3.4E−010
1443	−5E−011	−1E−011	2.2169	−4.2355E−012	−7.9E−011	−2.3E−010
1444	−3E−011	−1E−011	2.2169	−2.9143E−012	−5.4E−011	−1.6E−010
1445	−2E−011	−7E−012	2.2169	−1.9271E−012	−3.7E−011	−1.1E−010
1446	−2E−011	−5E−012	2.2169	−1.2981E−012	−2.6E−011	−7.6E−011
1447	−1E−011	−3E−012	2.2169	−9.4097E−013	−1.7E−011	−5.2E−011
1448	−7E−012	−2E−012	2.2169	−6.3596E−013	−1.2E−011	−3.6E−011
1449	−5E−012	−2E−012	2.2169	−3.9588E−013	−8.3E−012	−2.5E−011
1450	−3E−012	−1E−012	2.2169	−3.0531E−013	−5.7E−012	−1.7E−011
1451	−2E−012	−9E−013	2.2169	−1.9714E−013	−4.3E−012	−1.2E−011
1452	−2E−012	−7E−013	2.2169	−2.2179E−013	−3.1E−012	−8.2E−012
1453	−1E−012	−3E−013	2.2169	−1.4506E−013	−2.0E−012	−5.5E−012

1454	−9E−013	−5E−013	2.2169	−8.6387E−014	−1.2E−012	−3.9E−012
1455	−7E−013	−2E−013	2.2169	−9.4821E−014	−1.1E−012	−2.7E−012
1456	−9E−013	−5E−013	2.2169	−6.6075E−014	−2.9E−013	−1.6E−012
1457	−2E−013	−4E−014	2.2169	−3.9453E−014	−8.3E−014	−1.2E−012
1458	−9E−014	−2E−013	2.2169	4.8048E−014	−8.4E−014	−8.3E−013
1459	−2E−013	9.0E−014	2.2169	−4.9387E−014	−3.1E−013	−5.8E−013
1460	−2E−013	−1E−013	2.2169	−8.3665E−015	5.190E−014	−4.2E−013
1461	−2E−013	−8E−014	2.2169	4.0788E−014	8.914E−014	−3.2E−013
1462	−4E−013	−3E−013	2.2169	−6.5931E−014	−1.2E−013	5.16E−013
1463	−1E−013	−6E−014	2.2169	−8.2951E−014	2.050E−013	3.22E−013
1464	−5E−013	−4E−013	2.2169	−9.4271E−014	5.545E−013	5.70E−013
1465	−5E−013	−4E−013	2.2169	−1.105E−013	4.874E−013	1.39E−012
1466	−6E−013	−4E−013	2.2169	4.2869E−015	5.496E−013	1.52E−012
1467	−6E−013	−2E−013	2.2169	−9.9948E−014	4.855E−013	2.72E−012
1468	−1E−012	−6E−013	2.2169	−9.0401E−014	1.128E−012	3.60E−012
1469	−1E−012	−6E−013	2.2169	−7.3272E−014	1.722E−012	5.28E−012
1470	−2E−012	−4E−013	2.2169	−4.0837E−014	2.701E−012	7.92E−012
1471	−2E−012	−8E−013	2.2169	−1.4138E−013	3.872E−012	1.19E−011
1472	−4E−012	−1E−012	2.2169	−2.0894E−013	5.483E−012	1.70E−011
1473	−5E−012	−1E−012	2.2169	−5.062E−013	8.012E−012	2.48E−011
1474	−7E−012	−2E−012	2.2169	−7.459E−013	1.21E−011	3.67E−011
1475	−1E−011	−3E−012	2.2169	−1.0391E−012	1.737E−011	5.22E−011
1476	−2E−011	−5E−012	2.2169	−1.351E−012	2.591E−011	7.6E−011
1477	−2E−011	−6E−012	2.2169	−2.071E−012	3.737E−011	1.11E−010
1478	−3E−011	−1E−011	2.2169	−2.9134E−012	5.454E−011	1.61E−010
1479	−5E−011	−1E−011	2.2169	−4.1954E−012	7.866E−011	2.34E−010
1480	−7E−011	−2E−011	2.2169	−6.2027E−012	1.147E−010	3.40E−010
1481	−1E−010	−3E−011	2.2169	−8.9328E−012	1.671E−010	4.95E−010
1482	−1E−010	−4E−011	2.2169	−1.3013E−011	2.428E−010	7.20E−010
1483	−2E−010	−6E−011	2.2169	−1.8792E−011	3.530E−010	1.05E−009
1484	−3E−010	−9E−011	2.2169	−2.7609E−011	5.124E−010	1.52E−009
1485	−4E−010	−1E−010	2.2169	−3.9713E−011	7.461E−010	2.21E−009
1486	−6E−010	−2E−010	2.2169	−5.8393E−011	1.083E−009	3.22E−009
1487	−9E−010	−3E−010	2.2169	−8.3673E−011	1.577E−009	4.68E−009
1488	−1E−009	−4E−010	2.2169	−1.2371E−010	2.287E−009	6.81E−009
1489	−2E−009	−6E−010	2.2169	−1.7654E−010	3.333E−009	9.89E−009
1490	−3E−009	−9E−010	2.2169	−2.6178E−010	4.834E−009	1.44E−008
1491	−4E−009	−1E−009	2.2169	−3.7266E−010	7.045E−009	2.09E−008
1492	−6E−009	−2E−009	2.2169	−5.5434E−010	1.021E−008	3.04E−008
1493	−9E−009	−3E−009	2.2169	−7.8637E−010	1.489E−008	4.42E−008
1494	−1E−008	−4E−009	2.2169	−1.1747E−009	2.157E−008	6.44E−008
1495	−2E−008	−6E−009	2.2169	−1.6588E−009	3.148E−008	9.35E−008
1496	−3E−008	−8E−009	2.2169	−2.4902E−009	4.557E−008	1.36E−007
1497	−4E−008	−1E−008	2.2169	−3.4985E−009	6.653E−008	1.98E−007
1498	−6E−008	−2E−008	2.2169	−5.2813E−009	9.625E−008	2.88E−007
1499	−8E−008	−3E−008	2.2169	−7.3762E−009	1.406E−007	4.18E−007
1500	−1E−007	−4E−008	2.2169	−1.121E−008	2.033E−007	6.09E−007

1501	−2E−007	−5E−008	2.2169	−1.5548E−008	2.972E−007	8.84E−007
1502	−3E−007	−8E−008	2.2169	−2.3815E−008	4.292E−007	1.29E−006
1503	−4E−007	−1E−007	2.2169	−3.277E−008	6.282E−007	1.87E−006
1504	−5E−007	−2E−007	2.2169	−5.0664E−008	9.063E−007	2.73E−006
1505	−8E−007	−3E−007	2.2169	−6.9083E−008	1.328E−006	3.95E−006
1506	−1E−006	−3E−007	2.2169	−1.0799E−007	1.913E−006	5.77E−006
1507	−2E−006	−5E−007	2.2168	−1.4576E−007	2.807E−006	8.37E−006
1508	−2E−006	−7E−007	2.2168	−2.3084E−007	4.039E−006	1.22E−005
1509	−4E−006	−1E−006	2.2168	−3.0829E−007	5.932E−006	1.77E−005
1510	−5E−006	−1E−006	2.2167	−4.9583E−007	8.531E−006	2.59E−005
1511	−8E−006	−3E−006	2.2166	−6.5557E−007	1.254E−005	3.75E−005
1512	−1E−005	−3E−006	2.2165	−1.0737E−006	1.803E−005	5.48E−005
1513	−2E−005	−6E−006	2.2163	−1.41E−006	2.651E−005	7.97E−005
1514	−2E−005	−6E−006	2.216	−2.3583E−006	3.817E−005	0.00011645
1515	−4E−005	−2E−005	2.2155	−3.1017E−006	5.599E−005	0.00016991
1516	−4E−005	−1E−005	2.2149	−5.3103E−006	8.089E−005	0.00024821
1517	−9E−005	−4E−005	2.214	−7.1095E−006	0.00011766	0.00036436
1518	−8E−005	−1E−005	2.2126	−1.2454E−005	0.00017094	0.00053129
1519	−0.00022	−0.00012	2.2106	−1.7394E−005	0.00024254	0.00078686
1520	−0.00012	3.2E−006	2.2076	−3.0979E−005	0.00035394	0.0011385
1521	−0.00055	−0.00038	2.2032	−4.625E−005	0.00046811	0.0017006
1522	−2E−005	0.000109	2.1967	−8.2683E−005	0.00067373	0.0023974
1523	−0.00146	−0.00135	2.1872	−0.000133	0.00070216	0.003553
1524	0.0011773	0.000688	2.1732	−0.00023895	0.00087678	0.0046044
1525	−0.00450	−0.00571	2.1536	−0.00040646	−0.0002152	0.0061701
1526	0.01118	0.005256	2.1276	−0.00074121	−0.0016888	0.0053743
1527	−0.02320	−0.03345	2.0938	−0.0017863	−0.0098895	0.0031496
1528	0.094931	0.050735	2.0936	−0.00918	−0.019493	−0.007808
1529	0.030674	−0.01169	2.3885	0.0163	0.079372	−0.36935
1530	2.6289	2.6321	2.7574	1.7095E−011	0.61937	8.31E−010
1531	2.3204	2.2445	2.5891	−0.00035693	0.14822	0.68826
1532	2.0215	1.9757	2.281	0.016543	0.31923	0.49178
1533	1.9396	1.9293	2.1942	0.017601	0.45971	0.31364
1534	2.127	2.142	2.3845	0.0058906	0.58041	0.13816
1535	2.6289	2.6321	2.7574	1.6931E−011	−0.61937	−8.3E−010
1536	0.026249	−0.2165	2.3334	−2.4415E−010	0.22348	2.17E−010
1537	0.11256	0.1423	1.7494	6.1787E−012	−0.067947	1.12E−011
1538	−0.07944	−0.09547	2.3133	2.9271E−011	−0.0073217	7.29E−011
1539	0.034082	0.038125	2.2535	−1.5552E−011	−0.0049274	−5.5E−012
1540	−0.01951	−0.01491	2.2411	1.3262E−011	0.00057488	−2.0E−011
1541	0.0030961	0.006259	2.2447	−5.5125E−013	0.00013904	−1.1E−011
1542	−0.00642	−0.00252	2.2306	5.6696E−012	0.00047619	−1.1E−011
1543	−0.00100	0.001186	2.23	1.2887E−012	0.00045262	−6.9E−012
1544	−0.00254	−0.00047	2.2244	2.4888E−012	0.00025696	−5.6E−012
1545	−0.00096	0.000252	2.223	9.4918E−013	0.00027651	−3.5E−012
1546	−0.00109	−8E−005	2.2207	1.1026E−012	0.00013167	−2.6E−012
1547	−0.00055	5.7E−005	2.2198	5.3886E−013	0.00013968	−1.7E−012

1548	−0.00049	−1E−005	2.2188	5.0428E−013	6.624E−005	−1.2E−012
1549	−0.00028	1.3E−005	2.2183	2.7457E−013	6.667E−005	−9.0E−013
1550	−0.00022	1.7E−006	2.2178	2.2984E−013	3.289E−005	−6.3E−013
1551	−0.00014	3.0E−006	2.2175	1.3714E−013	3.122E−005	−4.4E−013
1552	−0.00010	2.3E−006	2.2173	9.9515E−014	1.616E−005	−3.3E−013
1553	−7E−005	7.9E−007	2.2172	5.3305E−014	1.454E−005	−2.4E−013
1554	−5E−005	1.4E−006	2.2171	6.344E−014	7.874E−006	−7.9E−014
1555	−3E−005	2.5E−007	2.217	7.0804E−015	6.763E−006	−1.3E−013
1556	−2E−005	7.0E−007	2.217	3.8411E−014	3.811E−006	−1.7E−013
1557	−2E−005	1.1E−007	2.217	2.1845E−014	3.150E−006	−1.7E−013
1558	−1E−005	3.3E−007	2.217	−7.9018E−015	1.836E−006	1.34E−014
1559	−7E−006	5.5E−008	2.2169	4.3698E−015	1.470E−006	−2.5E−014
1560	−5E−006	1.5E−007	2.2169	2.8724E−014	8.806E−007	4.32E−014
1561	−3E−006	3.0E−008	2.2169	−1.177E−014	6.875E−007	1.13E−013
1562	−2E−006	6.9E−008	2.2169	1.7066E−014	4.212E−007	−1.5E−014
1563	−2E−006	1.6E−008	2.2169	6.552E−015	3.22E−007	−2.5E−013
1564	−1E−006	3.1E−008	2.2169	−1.9141E−014	2.010E−007	−8.5E−014
1565	−8E−007	8.6E−009	2.2169	3.1907E−015	1.510E−007	7.04E−014
1566	−5E−007	1.4E−008	2.2169	1.646E−014	9.574E−008	−2.0E−013
1567	−4E−007	4.4E−009	2.2169	−4.093E−015	7.094E−008	−2.6E−013
1568	−2E−007	6.4E−009	2.2169	−5.3723E−015	4.555E−008	−2.3E−013
1569	−2E−007	2.3E−009	2.2169	−1.3286E−014	3.335E−008	−2.0E−013
1570	−1E−007	2.9E−009	2.2169	−1.9446E−014	2.164E−008	2.90E−015
1571	−8E−008	1.1E−009	2.2169	−7.5622E−015	1.570E−008	1.63E−013
1572	−6E−008	1.3E−009	2.2169	−1.4099E−014	1.028E−008	−5.6E−014
1573	−4E−008	5.7E−010	2.2169	−1.663E−014	7.392E−009	1.88E−014
1574	−3E−008	6.1E−010	2.2169	1.8623E−014	4.876E−009	−1.8E−014
1575	−2E−008	2.8E−010	2.2169	−8.9385E−015	3.483E−009	−1.4E−013
1576	−1E−008	2.8E−010	2.2169	−2.1069E−014	2.313E−009	−1.1E−013
1577	−9E−009	1.4E−010	2.2169	7.1867E−014	1.642E−009	1.24E−013
1578	−6E−009	1.3E−010	2.2169	−2.9288E−014	1.097E−009	−6.4E−015
1579	−4E−009	6.6E−011	2.2169	1.5387E−014	7.742E−010	−6.2E−014
1580	−3E−009	5.9E−011	2.2169	−2.5545E−014	5.196E−010	6.15E−014
1581	−2E−009	3.3E−011	2.2169	−2.443E−014	3.665E−010	−2.1E−013
1582	−1E−009	2.8E−011	2.2169	5.9728E−014	2.459E−010	−2.8E−014
1583	−9E−010	1.5E−011	2.2169	−7.9869E−015	1.728E−010	−1.6E−013
1584	−6E−010	1.4E−011	2.2169	−3.7226E−014	1.166E−010	−5.2E−014
1585	−4E−010	7.1E−012	2.2169	5.1661E−014	8.103E−011	1.42E−013
1586	−3E−010	5.9E−012	2.2169	−3.2652E−014	5.562E−011	8.34E−014
1587	−2E−010	3.1E−012	2.2169	3.2242E−015	3.854E−011	−2.0E−014
1588	−1E−010	3.3E−012	2.2169	5.7978E−014	2.577E−011	1.09E−013
1589	−1E−010	1.5E−012	2.2169	−1.0363E−014	1.820E−011	9.61E−014
1590	−7E−011	1.3E−012	2.2169	7.1678E−014	1.309E−011	−7.6E−014
1591	−5E−011	7.5E−013	2.2169	−4.26E−014	7.883E−012	−2.4E−013
1592	−3E−011	−2E−013	2.2169	5.0817E−014	6.349E−012	−2.5E−013
1593	−2E−011	3.5E−013	2.2169	−3.3788E−014	3.198E−012	−2.8E−013
1594	−2E−011	8.6E−014	2.2169	−2.7883E−014	3.360E−012	−4.8E−014

1595	−1E−011	1.6E−013	2.2169	−2.2154E−014	3.172E−012	3.12E−013
1596	−7E−012	5.5E−013	2.2169	5.0291E−014	1.320E−012	2.42E−013
1597	−5E−012	−7E−013	2.2169	−6.3133E−017	1.742E−013	−2.9E−013
1598	−3E−012	3.0E−013	2.2169	−1.7649E−014	1.594E−012	−2.2E−013
1599	−2E−012	2.7E−013	2.2169	1.0428E−013	−6.3E−014	4.15E−014
1600	−2E−012	−8E−013	2.2169	−4.4133E−014	1.079E−012	−4.7E−013
1601	−1E−012	6.1E−013	2.2169	5.5643E−014	4.622E−013	−1.2E−014
1602	−1E−012	2.9E−014	2.2169	9.4641E−015	−9.4E−013	1.12E−013
1603	−1E−012	−5E−013	2.2169	−3.2457E−014	−2.5E−013	−1.3E−013
1604	−7E−013	4.0E−013	2.2169	−3.224E−015	−1.1E−015	7.41E−014
1605	−1E−012	−5E−013	2.2169	−5.8483E−014	−2.1E−012	2.97E−014
1606	−1E−012	−3E−013	2.2169	−5.1123E−015	6.375E−013	2.49E−013
1607	−1E−012	2.8E−015	2.2169	2.4121E−014	−7.4E−013	1.09E−013
1608	−2E−012	−5E−013	2.2169	−8.1414E−016	−4.9E−013	−1.7E−013
1609	−2E−012	5.8E−013	2.2169	4.6902E−015	−7.5E−013	−1.7E−013
1610	−3E−012	−2E−013	2.2169	−1.0342E−014	−3.8E−014	1.57E−013
1611	−5E−012	−5E−014	2.2169	−7.0665E−014	−1.9E−012	−6.2E−014
1612	−7E−012	−4E−013	2.2169	1.1516E−013	−2.0E−012	−9.5E−014
1613	−1E−011	1.8E−013	2.2169	−1.5289E−014	−1.2E−013	−3.4E−013
1614	−1E−011	5.7E−013	2.2169	2.1049E−014	−3.8E−012	−4.1E−013
1615	−2E−011	1.1E−012	2.2169	−1.3557E−013	−4.4E−012	−3.1E−014
1616	−3E−011	1.1E−012	2.2169	5.9058E−014	−5.7E−012	1.44E−015
1617	−4E−011	9.0E−013	2.2169	−2.8316E−014	−5.7E−012	1.29E−013
1618	−7E−011	1E−012	2.2169	−3.9406E−014	−1.4E−011	1.92E−013
1619	−1E−010	2.3E−012	2.2169	−3.729E−014	−1.8E−011	2.72E−013
1620	−1E−010	2.7E−012	2.2169	5.3922E−014	−2.8E−011	5.24E−013
1621	−2E−010	3.4E−012	2.2169	3.3496E−014	−3.8E−011	−1.3E−013
1622	−3E−010	5.6E−012	2.2169	−7.5007E−014	−5.6E−011	6.61E−014
1623	−4E−010	8.0E−012	2.2169	9.9102E−014	−8.0E−011	−2.8E−013
1624	−6E−010	1.3E−011	2.2169	−2.4859E−014	−1.2E−010	−5.8E−013
1625	−9E−010	1.7E−011	2.2169	5.7629E−014	−1.7E−010	−4.1E−013
1626	−1E−009	2.8E−011	2.2169	−1.0893E−013	−2.5E−010	−2.5E−015
1627	−2E−009	3.2E−011	2.2169	−3.3961E−015	−3.6E−010	−2.9E−013
1628	−3E−009	6.0E−011	2.2169	−5.8968E−015	−5.2E−010	−2.0E−013
1629	−4E−009	6.5E−011	2.2169	5.2758E−014	−7.7E−010	−4.6E−013
1630	−6E−009	1.3E−010	2.2169	8.8884E−016	−1.1E−009	−1.4E−013
1631	−9E−009	1.3E−010	2.2169	−1.8691E−014	−1.6E−009	−1.2E−013
1632	−1E−008	2.8E−010	2.2169	7.9833E−014	−2.3E−009	6.77E−014
1633	−2E−008	2.8E−010	2.2169	−5.3182E−014	−3.5E−009	−5.3E−013
1634	−3E−008	6.1E−010	2.2169	6.6265E−014	−4.9E−009	−1.6E−013
1635	−4E−008	5.7E−010	2.2169	−1.051E−014	−7.4E−009	−5.9E−013
1636	−6E−008	1.3E−009	2.2169	−1.009E−013	−1.0E−008	−6.6E−014
1637	−8E−008	1.1E−009	2.2169	5.5306E−014	−1.6E−008	−1.7E−013
1638	−1E−007	2.9E−009	2.2169	5.8026E−014	−2.2E−008	−4.5E−013
1639	−2E−007	2.3E−009	2.2169	−1.5321E−014	−3.3E−008	−1E−013
1640	−2E−007	6.4E−009	2.2169	−1.5353E−014	−4.6E−008	9.75E−014
1641	−4E−007	4.4E−009	2.2169	1.2871E−013	−7.1E−008	6.90E−013

1642	−5E−007	1.4E−008	2.2169	−2.1025E−014	−9.6E−008	3.34E−014
1643	−8E−007	8.5E−009	2.2169	4.3016E−014	−1.5E−007	−2.9E−013
1644	−1E−006	3.1E−008	2.2169	−1.6087E−014	−2.0E−007	−1.5E−014
1645	−2E−006	1.6E−008	2.2169	7.4842E−014	−3.2E−007	−9.5E−013
1646	−2E−006	6.9E−008	2.2169	1.1369E−014	−4.2E−007	−4.5E−013
1647	−3E−006	3.0E−008	2.2169	−4.981E−014	−6.9E−007	−6.2E−013
1648	−5E−006	1.5E−007	2.2169	7.9775E−014	−8.8E−007	8.13E−014
1649	−7E−006	5.5E−008	2.2169	3.3897E−014	−1.5E−006	1.77E−013
1650	−1E−005	3.3E−007	2.217	−9.4286E−014	−1.8E−006	−3.5E−013
1651	−2E−005	1.1E−007	2.217	2.0326E−014	−3.2E−006	−9.1E−014
1652	−2E−005	7.0E−007	2.217	−7.2115E−016	−3.8E−006	−1.6E−013
1653	−3E−005	2.5E−007	2.217	9.8671E−015	−6.8E−006	9.83E−014
1654	−5E−005	1.4E−006	2.2171	9.0574E−014	−7.9E−006	−1.9E−013
1655	−7E−005	7.9E−007	2.2172	−1.14E−013	−1.5E−005	4.28E−013
1656	−0.00010	2.3E−006	2.2173	1.6334E−013	−1.6E−005	2.26E−013
1657	−0.00014	3.0E−006	2.2176	1.1808E−013	−3.1E−005	2.43E−013
1658	−0.00022	1.7E−006	2.2178	1.9783E−013	−3.3E−005	7.50E−013
1659	−0.00028	1.3E−005	2.2183	3.5192E−013	−6.7E−005	6.85E−013
1660	−0.00049	−1E−005	2.2188	4.7977E−013	−6.6E−005	1.05E−013
1661	−0.00055	5.7E−005	2.2198	5.2611E−013	−0.0001397	1.71E−012
1662	−0.00109	−8E−005	2.2207	1.1372E−012	−0.0001317	2.01E−012
1663	−0.00096	0.000252	2.223	8.4488E−013	−0.0002765	2.87E−012
1664	−0.00254	−0.00047	2.2244	2.4505E−012	−0.0002570	5.69E−012
1665	−0.00100	0.001186	2.23	1.2395E−012	−0.0004526	7.93E−012
1666	−0.00642	−0.00252	2.2306	5.6228E−012	−0.0004762	1.25E−011
1667	0.0030961	0.006259	2.2447	−4.3963E−013	−0.0001390	1.12E−011
1668	−0.01951	−0.01491	2.2411	1.3204E−011	−0.0005749	1.98E−011
1669	0.034082	0.038125	2.2535	−1.5422E−011	0.0049274	5.35E−012
1670	−0.07944	−0.09547	2.3133	2.918E−011	0.0073217	−7.3E−011
1671	0.11256	0.1423	1.7494	6.2205E−012	0.067947	−1.2E−011
1672	0.026249	−0.2165	2.3334	−2.442E−010	−0.22348	−2.2E−010
1673	2.3204	2.2445	2.5891	−0.00035693	−0.14822	−0.68826
1674	2.0215	1.9757	2.281	0.016543	−0.31923	−0.49178
1675	1.9396	1.9293	2.1942	0.017601	−0.45971	−0.31364
1676	2.127	2.142	2.3845	0.0058906	−0.58041	−0.13816
1677	0.030674	−0.01169	2.3885	−0.0163	0.079372	0.36935
1678	0.094931	0.050735	2.0936	0.00918	−0.019493	0.007808
1679	−0.02320	−0.03345	2.0938	0.0017863	−0.0098895	−0.003150
1680	0.01118	0.005256	2.1276	0.00074121	−0.0016888	−0.005374
1681	−0.00450	−0.00571	2.1536	0.00040646	−0.0002152	−0.006170
1682	0.0011773	0.000688	2.1732	0.00023895	0.00087678	−0.004604
1683	−0.00146	−0.00135	2.1872	0.000133	0.00070216	−0.003553
1684	−2E−005	0.000109	2.1967	8.2683E−005	0.00067373	−0.002397
1685	−0.00055	−0.00038	2.2032	4.625E−005	0.00046811	−0.001701
1686	−0.00012	3.2E−006	2.2076	3.0979E−005	0.00035394	−0.001139
1687	−0.00022	−0.00012	2.2106	1.7394E−005	0.00024254	−0.000787
1688	−8E−005	−1E−005	2.2126	1.2454E−005	0.00017094	−0.000531

1689	−9E−005	−4E−005	2.214	7.1095E−006	0.00011766	−0.000364
1690	−4E−005	−1E−005	2.2149	5.3103E−006	8.089E−005	−0.000248
1691	−4E−005	−2E−005	2.2155	3.1017E−006	5.599E−005	−0.000170
1692	−2E−005	−6E−006	2.216	2.3583E−006	3.817E−005	−0.000116
1693	−2E−005	−6E−006	2.2163	1.41E−006	2.651E−005	−8.0E−005
1694	−1E−005	−3E−006	2.2165	1.0737E−006	1.803E−005	−5.5E−005
1695	−8E−006	−3E−006	2.2166	6.5557E−007	1.254E−005	−3.8E−005
1696	−5E−006	−1E−006	2.2167	4.9583E−007	8.531E−006	−2.6E−005
1697	−4E−006	−1E−006	2.2168	3.0829E−007	5.932E−006	−1.8E−005
1698	−2E−006	−7E−007	2.2168	2.3084E−007	4.039E−006	−1.2E−005
1699	−2E−006	−5E−007	2.2168	1.4576E−007	2.807E−006	−8.4E−006
1700	−1E−006	−3E−007	2.2169	1.0799E−007	1.913E−006	−5.8E−006
1701	−8E−007	−3E−007	2.2169	6.9082E−008	1.328E−006	−4.0E−006
1702	−5E−007	−2E−007	2.2169	5.0664E−008	9.063E−007	−2.7E−006
1703	−4E−007	−1E−007	2.2169	3.277E−008	6.282E−007	−1.9E−006
1704	−3E−007	−8E−008	2.2169	2.3815E−008	4.292E−007	−1.3E−006
1705	−2E−007	−5E−008	2.2169	1.5548E−008	2.972E−007	−8.8E−007
1706	−1E−007	−4E−008	2.2169	1.121E−008	2.033E−007	−6.1E−007
1707	−8E−008	−3E−008	2.2169	7.3763E−009	1.406E−007	−4.2E−007
1708	−6E−008	−2E−008	2.2169	5.2813E−009	9.625E−008	−2.9E−007
1709	−4E−008	−1E−008	2.2169	3.4985E−009	6.653E−008	−2.0E−007
1710	−3E−008	−8E−009	2.2169	2.4901E−009	4.557E−008	−1.4E−007
1711	−2E−008	−6E−009	2.2169	1.6588E−009	3.148E−008	−9.3E−008
1712	−1E−008	−4E−009	2.2169	1.1746E−009	2.157E−008	−6.4E−008
1713	−9E−009	−3E−009	2.2169	7.8631E−010	1.489E−008	−4.4E−008
1714	−6E−009	−2E−009	2.2169	5.5441E−010	1.021E−008	−3.0E−008
1715	−4E−009	−1E−009	2.2169	3.7259E−010	7.045E−009	−2.1E−008
1716	−3E−009	−9E−010	2.2169	2.6174E−010	4.833E−009	−1.4E−008
1717	−2E−009	−6E−010	2.2169	1.7649E−010	3.333E−009	−9.9E−009
1718	−1E−009	−4E−010	2.2169	1.2359E−010	2.287E−009	−6.8E−009
1719	−9E−010	−3E−010	2.2169	8.3616E−011	1.577E−009	−4.7E−009
1720	−6E−010	−2E−010	2.2169	5.8345E−011	1.083E−009	−3.2E−009
1721	−4E−010	−1E−010	2.2169	3.96E−011	7.460E−010	−2.2E−009
1722	−3E−010	−9E−011	2.2169	2.7571E−011	5.124E−010	−1.5E−009
1723	−2E−010	−6E−011	2.2169	1.8781E−011	3.530E−010	−1.0E−009
1724	−1E−010	−4E−011	2.2169	1.311E−011	2.424E−010	−7.2E−010
1725	−1E−010	−3E−011	2.2169	8.9207E−012	1.668E−010	−4.9E−010
1726	−7E−011	−2E−011	2.2169	6.146E−012	1.145E−010	−3.4E−010
1727	−5E−011	−1E−011	2.2169	4.2026E−012	7.905E−011	−2.3E−010
1728	−3E−011	−1E−011	2.2169	2.8697E−012	5.413E−011	−1.6E−010
1729	−2E−011	−7E−012	2.2169	1.912E−012	3.731E−011	−1.1E−010
1730	−2E−011	−5E−012	2.2169	1.3199E−012	2.582E−011	−7.6E−011
1731	−1E−011	−3E−012	2.2169	9.388E−013	1.773E−011	−5.2E−011
1732	−7E−012	−2E−012	2.2169	6.2808E−013	1.226E−011	−3.6E−011
1733	−5E−012	−1E−012	2.2169	4.6775E−013	8.229E−012	−2.5E−011
1734	−3E−012	−1E−012	2.2169	3.1155E−013	5.774E−012	−1.7E−011
1735	−3E−012	−1E−012	2.2169	2.1504E−013	3.920E−012	−1.2E−011

1736	−2E−012	−5E−013	2.2169	2.4697E−013	2.861E−012	−7.9E−012
1737	−1E−012	−2E−013	2.2169	1.1693E−013	1.818E−012	−5.5E−012
1738	−6E−013	−7E−014	2.2169	6.2104E−014	1.291E−012	−4.0E−012
1739	−6E−013	−2E−013	2.2169	1.097E−013	9.245E−013	−2.8E−012
1740	−7E−013	−3E−013	2.2169	7.1416E−014	6.169E−013	−1.7E−012
1741	−2E−013	−8E−014	2.2169	3.6024E−014	3.032E−013	−1.2E−012
1742	7.3E−014	1E−013	2.2169	4.5453E−014	3.829E−013	−6.5E−013
1743	−5E−013	−5E−013	2.2169	7.7961E−014	2.369E−013	−3.0E−013
1744	−1E−013	−9E−014	2.2169	4.7896E−014	−1.6E−014	−3.0E−013
1745	−2E−013	−1E−013	2.2169	−1.1416E−014	−1.5E−013	−3.1E−013
1746	−3E−013	−2E−013	2.2169	4.2032E−014	−4.6E−014	7.60E−013
1747	−2E−014	1.0E−013	2.2169	1.0447E−013	−3.5E−013	4.06E−013
1748	−3E−013	−7E−014	2.2169	6.3992E−014	−3.6E−013	5.16E−013
1749	−1E−013	−1E−015	2.2169	7.0808E−014	−3.9E−013	1.10E−012
1750	−7E−013	−4E−013	2.2169	5.9197E−014	−6.0E−013	1.95E−012
1751	−4E−013	−7E−014	2.2169	5.163E−014	−8.0E−013	2.60E−012
1752	−7E−013	−7E−014	2.2169	8.664E−014	−1.1E−012	3.71E−012
1753	−1E−012	−6E−013	2.2169	1.6877E−014	−1.3E−012	5.30E−012
1754	−1E−012	−4E−014	2.2169	1.137E−013	−2.7E−012	7.88E−012
1755	−2E−012	−6E−013	2.2169	2.8362E−014	−3.7E−012	1.17E−011
1756	−4E−012	−1E−012	2.2169	2.9334E−013	−5.5E−012	1.72E−011
1757	−5E−012	−1E−012	2.2169	4.5077E−013	−8.0E−012	2.55E−011
1758	−7E−012	−2E−012	2.2169	7.637E−013	−1.2E−011	3.57E−011
1759	−1E−011	−3E−012	2.2169	1.0396E−012	−1.8E−011	5.23E−011
1760	−2E−011	−5E−012	2.2169	1.3397E−012	−2.6E−011	7.64E−011
1761	−2E−011	−7E−012	2.2169	1.975E−012	−3.7E−011	1.10E−010
1762	−3E−011	−1E−011	2.2169	2.9429E−012	−5.4E−011	1.60E−010
1763	−5E−011	−1E−011	2.2169	4.2474E−012	−7.9E−011	2.34E−010
1764	−7E−011	−2E−011	2.2169	6.1607E−012	−1.1E−010	3.40E−010
1765	−1E−010	−3E−011	2.2169	8.8821E−012	−1.7E−010	4.95E−010
1766	−1E−010	−4E−011	2.2169	1.3061E−011	−2.4E−010	7.20E−010
1767	−2E−010	−6E−011	2.2169	1.876E−011	−3.5E−010	1.05E−009
1768	−3E−010	−9E−011	2.2169	2.7673E−011	−5.1E−010	1.52E−009
1769	−4E−010	−1E−010	2.2169	3.9556E−011	−7.5E−010	2.21E−009
1770	−6E−010	−2E−010	2.2169	5.8564E−011	−1.1E−009	3.22E−009
1771	−9E−010	−3E−010	2.2169	8.367E−011	−1.6E−009	4.68E−009
1772	−1E−009	−4E−010	2.2169	1.2365E−010	−2.3E−009	6.81E−009
1773	−2E−009	−6E−010	2.2169	1.7654E−010	−3.3E−009	9.89E−009
1774	−3E−009	−9E−010	2.2169	2.6174E−010	−4.8E−009	1.44E−008
1775	−4E−009	−1E−009	2.2169	3.7265E−010	−7.0E−009	2.09E−008
1776	−6E−009	−2E−009	2.2169	5.5443E−010	−1.0E−008	3.04E−008
1777	−9E−009	−3E−009	2.2169	7.8634E−010	−1.5E−008	4.42E−008
1778	−1E−008	−4E−009	2.2169	1.1746E−009	−2.2E−008	6.44E−008
1779	−2E−008	−6E−009	2.2169	1.6589E−009	−3.1E−008	9.35E−008
1780	−3E−008	−8E−009	2.2169	2.4902E−009	−4.6E−008	1.36E−007
1781	−4E−008	−1E−008	2.2169	3.4986E−009	−6.7E−008	1.98E−007
1782	−6E−008	−2E−008	2.2169	5.2812E−009	−9.6E−008	2.88E−007

1783	−8E−008	−3E−008	2.2169	7.3763E−009	−1.4E−007	4.18E−007
1784	−1E−007	−4E−008	2.2169	1.121E−008	−2.0E−007	6.09E−007
1785	−2E−007	−5E−008	2.2169	1.5548E−008	−3.0E−007	8.84E−007
1786	−3E−007	−8E−008	2.2169	2.3815E−008	−4.3E−007	1.29E−006
1787	−4E−007	−1E−007	2.2169	3.277E−008	−6.3E−007	1.87E−006
1788	−5E−007	−2E−007	2.2169	5.0664E−008	−9.1E−007	2.73E−006
1789	−8E−007	−3E−007	2.2169	6.9082E−008	−1.3E−006	3.95E−006
1790	−1E−006	−3E−007	2.2169	1.0799E−007	−1.9E−006	5.77E−006
1791	−2E−006	−5E−007	2.2168	1.4576E−007	−2.8E−006	8.37E−006
1792	−2E−006	−7E−007	2.2168	2.3084E−007	−4.0E−006	1.22E−005
1793	−4E−006	−1E−006	2.2168	3.0829E−007	−5.9E−006	1.77E−005
1794	−5E−006	−1E−006	2.2167	4.9583E−007	−8.5E−006	2.59E−005
1795	−8E−006	−3E−006	2.2166	6.5557E−007	−1.3E−005	3.75E−005
1796	−1E−005	−3E−006	2.2165	1.0737E−006	−1.8E−005	5.48E−005
1797	−2E−005	−6E−006	2.2163	1.41E−006	−2.7E−005	7.97E−005
1798	−2E−005	−6E−006	2.216	2.3583E−006	−3.8E−005	0.00011645
1799	−4E−005	−2E−005	2.2155	3.1017E−006	−5.6E−005	0.00016991
1800	−4E−005	−1E−005	2.2149	5.3103E−006	−8.1E−005	0.00024821
1801	−9E−005	−4E−005	2.214	7.1095E−006	−0.0001177	0.00036436
1802	−8E−005	−1E−005	2.2126	1.2454E−005	−0.0001709	0.00053129
1803	−0.00022	−0.00012	2.2106	1.7394E−005	−0.0002425	0.00078686
1804	−0.00012	3.2E−006	2.2076	3.0979E−005	−0.0003539	0.0011385
1805	−0.00055	−0.00038	2.2032	4.625E−005	−0.0004681	0.0017006
1806	−2E−005	0.000109	2.1967	8.2683E−005	−0.0006737	0.0023974
1807	−0.00146	−0.00135	2.1872	0.000133	−0.0007022	0.003553
1808	0.0011773	0.000688	2.1732	0.00023895	−0.0008768	0.0046044
1809	−0.00450	−0.00571	2.1536	0.00040646	0.00021518	0.0061701
1810	0.01118	0.005256	2.1276	0.00074121	0.0016888	0.0053743
1811	−0.02320	−0.03345	2.0938	0.0017863	0.0098895	0.0031496
1812	0.094931	0.050735	2.0936	0.00918	0.019493	−0.007808
1813	0.030674	−0.01169	2.3885	−0.0163	−0.079372	−0.36935
1814	0.21345	0.081784	2.2513	−0.011031	0.13622	0.29124
1815	0.060004	0.015744	2.0938	0.010971	−0.049702	0.032701
1816	−0.01477	−0.03114	2.135	0.0029355	−0.01726	−0.002262
1817	0.0087819	0.004722	2.1721	0.0015101	−0.0043947	−0.008668
1818	−0.00527	−0.00576	2.184	0.00088875	−0.0003996	−0.009148
1819	0.0005965	0.001551	2.197	0.00070191	0.0012801	−0.007319
1820	−0.00218	−0.00128	2.2022	0.00040918	0.001229	−0.005514
1821	−0.00037	0.000511	2.2074	0.00030054	0.0011464	−0.003920
1822	−0.00090	−0.00031	2.21	0.00017987	0.00081889	−0.002763
1823	−0.00030	0.000164	2.2123	0.0001281	0.00062315	−0.001906
1824	−0.00038	−7E−005	2.2136	7.9421E−005	0.00042585	−0.001320
1825	−0.00017	5.3E−005	2.2147	5.582E−005	0.0003057	−0.000904
1826	−0.00016	−2E−005	2.2154	3.5786E−005	0.00020756	−0.000623
1827	−9E−005	1.7E−005	2.2158	2.4969E−005	0.00014583	−0.000427
1828	−7E−005	−3E−006	2.2162	1.643E−005	9.920E−005	−0.000294
1829	−4E−005	6.1E−006	2.2164	1.1404E−005	6.908E−005	−0.000201

1830	−3E−005	−2E−007	2.2166	7.6398E−006	4.713E−005	−0.000138
1831	−2E−005	2.3E−006	2.2167	5.2819E−006	3.268E−005	−9.5E−005
1832	−2E−005	2.8E−007	2.2167	3.5795E−006	2.235E−005	−6.5E−005
1833	−1E−005	9.0E−007	2.2168	2.4675E−006	1.546E−005	−4.5E−005
1834	−7E−006	2.4E−007	2.2168	1.6842E−006	1.059E−005	−3.1E−005
1835	−5E−006	3.8E−007	2.2169	1.1587E−006	7.317E−006	−2.1E−005
1836	−3E−006	1.5E−007	2.2169	7.9419E−007	5.017E−006	−1.5E−005
1837	−2E−006	1.6E−007	2.2169	5.4571E−007	3.463E−006	−1E−005
1838	−2E−006	7.8E−008	2.2169	3.7496E−007	2.376E−006	−6.9E−006
1839	−1E−006	7.4E−008	2.2169	2.5746E−007	1.639E−006	−4.7E−006
1840	−7E−007	4E−008	2.2169	1.7714E−007	1.125E−006	−3.3E−006
1841	−5E−007	3.4E−008	2.2169	1.2159E−007	7.756E−007	−2.2E−006
1842	−4E−007	2.0E−008	2.2169	8.3717E−008	5.326E−007	−1.5E−006
1843	−2E−007	1.6E−008	2.2169	5.7458E−008	3.670E−007	−1.1E−006
1844	−2E−007	9.6E−009	2.2169	3.9573E−008	2.521E−007	−7.3E−007
1845	−1E−007	7.3E−009	2.2169	2.7162E−008	1.737E−007	−5.0E−007
1846	−8E−008	4.6E−009	2.2169	1.8709E−008	1.193E−007	−3.5E−007
1847	−5E−008	3.4E−009	2.2169	1.2843E−008	8.217E−008	−2.4E−007
1848	−4E−008	2.2E−009	2.2169	8.8456E−009	5.646E−008	−1.6E−007
1849	−3E−008	1.6E−009	2.2169	6.0733E−009	3.888E−008	−1.1E−007
1850	−2E−008	1.1E−009	2.2169	4.1825E−009	2.672E−008	−7.7E−008
1851	−1E−008	7.6E−010	2.2169	2.8722E−009	1.839E−008	−5.3E−008
1852	−8E−009	5.1E−010	2.2169	1.9777E−009	1.264E−008	−3.7E−008
1853	−6E−009	3.6E−010	2.2169	1.3584E−009	8.702E−009	−2.5E−008
1854	−4E−009	2.4E−010	2.2169	9.3519E−010	5.982E−009	−1.7E−008
1855	−3E−009	1.7E−010	2.2169	6.4249E−010	4.117E−009	−1.2E−008
1856	−2E−009	1.1E−010	2.2169	4.4232E−010	2.830E−009	−8.2E−009
1857	−1E−009	8.0E−011	2.2169	3.039E−010	1.948E−009	−5.6E−009
1858	−9E−010	5.4E−011	2.2169	2.0914E−010	1.339E−009	−3.9E−009
1859	−6E−010	3.8E−011	2.2169	1.4375E−010	9.215E−010	−2.7E−009
1860	−4E−010	2.6E−011	2.2169	9.8902E−011	6.336E−010	−1.8E−009
1861	−3E−010	1.8E−011	2.2169	6.7978E−011	4.361E−010	−1.3E−009
1862	−2E−010	1.2E−011	2.2169	4.6876E−011	2.996E−010	−8.6E−010
1863	−1E−010	8.4E−012	2.2169	3.2135E−011	2.063E−010	−5.9E−010
1864	−9E−011	5.6E−012	2.2169	2.2046E−011	1.418E−010	−4.1E−010
1865	−6E−011	4.0E−012	2.2169	1.5235E−011	9.767E−011	−2.8E−010
1866	−4E−011	2.6E−012	2.2169	1.0474E−011	6.706E−011	−1.9E−010
1867	−3E−011	2.0E−012	2.2169	7.1313E−012	4.612E−011	−1.3E−010
1868	−2E−011	1.6E−012	2.2169	4.9386E−012	3.183E−011	−9.1E−011
1869	−1E−011	8.6E−013	2.2169	3.3381E−012	2.165E−011	−6.3E−011
1870	−1E−011	5.6E−014	2.2169	2.3494E−012	1.488E−011	−4.3E−011
1871	−7E−012	3.3E−013	2.2169	1.5954E−012	1.035E−011	−3.0E−011
1872	−5E−012	2.2E−013	2.2169	1.0363E−012	7.428E−012	−2.1E−011
1873	−3E−012	7.8E−014	2.2169	8.2712E−013	5.008E−012	−1.4E−011
1874	−3E−012	−4E−014	2.2169	4.9447E−013	3.510E−012	−9.6E−012
1875	−2E−012	−2E−013	2.2169	4.1516E−013	2.176E−012	−6.7E−012
1876	−2E−012	−4E−013	2.2169	3.076E−013	1.759E−012	−4.3E−012

1877	−1E−012	−3E−013	2.2169	2.6529E−013	9.080E−013	−3.0E−012
1878	−7E−013	−2E−013	2.2169	1.3277E−013	7.036E−013	−1.9E−012
1879	−4E−013	−7E−014	2.2169	1.1969E−013	2.075E−013	−1.3E−012
1880	−3E−013	−3E−014	2.2169	1.1865E−013	3.237E−013	−6.6E−013
1881	−4E−014	2.5E−013	2.2169	3.734E−014	2.320E−013	−3.7E−013
1882	−2E−013	−2E−014	2.2169	1.1574E−013	3.238E−014	2.04E−013
1883	−4E−013	−9E−014	2.2169	1.2973E−013	−1.9E−013	2.03E−013
1884	−3E−013	−2E−013	2.2169	4.4555E−014	−4.8E−013	8.41E−013
1885	−8E−013	−3E−013	2.2169	8.2939E−014	−4.9E−013	1.35E−012
1886	−4E−013	1.5E−013	2.2169	2.2895E−013	−7.9E−013	2.13E−012
1887	−7E−013	8.4E−014	2.2169	1.9309E−013	−1.1E−012	2.84E−012
1888	−1E−012	−3E−013	2.2169	1.4272E−013	−1.6E−012	4.47E−012
1889	−1E−012	1.2E−013	2.2169	3.439E−013	−2.1E−012	6.76E−012
1890	−3E−012	−1E−013	2.2169	3.6383E−013	−3.4E−012	9.45E−012
1891	−3E−012	2.3E−013	2.2169	8.0954E−013	−4.8E−012	1.41E−011
1892	−5E−012	4.0E−013	2.2169	1.1185E−012	−7.1E−012	2.05E−011
1893	−7E−012	2.1E−013	2.2169	1.4706E−012	−1.1E−011	2.97E−011
1894	−1E−011	7.8E−013	2.2169	2.2958E−012	−1.5E−011	4.30E−011
1895	−1E−011	6.1E−013	2.2169	3.3834E−012	−2.2E−011	6.29E−011
1896	−2E−011	1.1E−012	2.2169	5.0476E−012	−3.2E−011	9.12E−011
1897	−3E−011	1.9E−012	2.2169	7.1819E−012	−4.6E−011	1.33E−010
1898	−4E−011	2.6E−012	2.2169	1.0427E−011	−6.7E−011	1.93E−010
1899	−6E−011	4.0E−012	2.2169	1.5156E−011	−9.7E−011	2.81E−010
1900	−9E−011	6.1E−012	2.2169	2.2198E−011	−1.4E−010	4.09E−010
1901	−1E−010	8.7E−012	2.2169	3.2163E−011	−2.1E−010	5.94E−010
1902	−2E−010	1.2E−011	2.2169	4.6778E−011	−3.0E−010	8.64E−010
1903	−3E−010	1.8E−011	2.2169	6.8004E−011	−4.4E−010	1.26E−009
1904	−4E−010	2.6E−011	2.2169	9.8881E−011	−6.3E−010	1.83E−009
1905	−6E−010	3.8E−011	2.2169	1.4374E−010	−9.2E−010	2.66E−009
1906	−9E−010	5.5E−011	2.2169	2.0932E−010	−1.3E−009	3.86E−009
1907	−1E−009	8.0E−011	2.2169	3.039E−010	−1.9E−009	5.62E−009
1908	−2E−009	1.1E−010	2.2169	4.4224E−010	−2.8E−009	8.17E−009
1909	−3E−009	1.7E−010	2.2169	6.4248E−010	−4.1E−009	1.19E−008
1910	−4E−009	2.4E−010	2.2169	9.3524E−010	−6.0E−009	1.73E−008
1911	−6E−009	3.6E−010	2.2169	1.3584E−009	−8.7E−009	2.51E−008
1912	−8E−009	5.1E−010	2.2169	1.9778E−009	−1.3E−008	3.65E−008
1913	−1E−008	7.6E−010	2.2169	2.8722E−009	−1.8E−008	5.31E−008
1914	−2E−008	1.1E−009	2.2169	4.1825E−009	−2.7E−008	7.72E−008
1915	−3E−008	1.6E−009	2.2169	6.0733E−009	−3.9E−008	1.12E−007
1916	−4E−008	2.2E−009	2.2169	8.8456E−009	−5.6E−008	1.63E−007
1917	−5E−008	3.4E−009	2.2169	1.2843E−008	−8.2E−008	2.37E−007
1918	−8E−008	4.6E−009	2.2169	1.8709E−008	−1.2E−007	3.45E−007
1919	−1E−007	7.3E−009	2.2169	2.7162E−008	−1.7E−007	5.02E−007
1920	−2E−007	9.6E−009	2.2169	3.9573E−008	−2.5E−007	7.30E−007
1921	−2E−007	1.6E−008	2.2169	5.7458E−008	−3.7E−007	1.06E−006
1922	−4E−007	2.0E−008	2.2169	8.3717E−008	−5.3E−007	1.54E−006
1923	−5E−007	3.4E−008	2.2169	1.2159E−007	−7.8E−007	2.24E−006

1924	−7E−007	4E−008	2.2169	1.7714E−007	−1.1E−006	3.26E−006
1925	−1E−006	7.4E−008	2.2169	2.5746E−007	−1.6E−006	4.74E−006
1926	−2E−006	7.8E−008	2.2169	3.7496E−007	−2.4E−006	6.90E−006
1927	−2E−006	1.6E−007	2.2169	5.4571E−007	−3.5E−006	1.00E−005
1928	−3E−006	1.5E−007	2.2169	7.9419E−007	−5.0E−006	1.46E−005
1929	−5E−006	3.8E−007	2.2169	1.1587E−006	−7.3E−006	2.12E−005
1930	−7E−006	2.4E−007	2.2168	1.6842E−006	−1.1E−005	3.09E−005
1931	−1E−005	9.0E−007	2.2168	2.4675E−006	−1.5E−005	4.49E−005
1932	−2E−005	2.8E−007	2.2167	3.5795E−006	−2.2E−005	6.54E−005
1933	−2E−005	2.3E−006	2.2167	5.2819E−006	−3.3E−005	9.50E−005
1934	−3E−005	−2E−007	2.2166	7.6398E−006	−4.7E−005	0.00013849
1935	−4E−005	6.1E−006	2.2164	1.1404E−005	−6.9E−005	0.00020122
1936	−7E−005	−3E−006	2.2162	1.643E−005	−9.9E−005	0.0002936
1937	−9E−005	1.7E−005	2.2158	2.4969E−005	−0.0001458	0.00042652
1938	−0.00016	−2E−005	2.2154	3.5786E−005	−0.0002076	0.00062293
1939	−0.00017	5.3E−005	2.2147	5.582E−005	−0.0003057	0.00090401
1940	−0.00038	−7E−005	2.2136	7.9421E−005	−0.0004259	0.0013197
1941	−0.00030	0.000164	2.2123	0.0001281	−0.0006232	0.0019061
1942	−0.00090	−0.00031	2.21	0.00017987	−0.0008189	0.002763
1943	−0.00037	0.000511	2.2074	0.00030054	−0.0011464	0.0039202
1944	−0.00218	−0.00128	2.2022	0.00040918	−0.001229	0.0055141
1945	0.0005965	0.001551	2.197	0.00070191	−0.0012801	0.0073186
1946	−0.00527	−0.00576	2.184	0.00088875	0.00039957	0.0091478
1947	0.0087819	0.004722	2.1721	0.0015101	0.0043947	0.008668
1948	−0.01477	−0.03114	2.135	0.0029355	0.01726	0.0022618
1949	0.060004	0.015744	2.0938	0.010971	0.049702	−0.032701
1950	0.21345	0.081784	2.2513	−0.011031	−0.13622	−0.29124
1951	0.23633	0.064305	2.1736	−0.0016231	0.19118	0.19831
1952	0.067165	0.024389	2.0678	0.0096304	−0.067023	0.028917
1953	−0.01675	−0.03145	2.1522	0.0017944	−0.02513	−0.002077
1954	0.0068781	0.005558	2.2062	0.00085584	−0.005728	−0.007526
1955	−0.00702	−0.00539	2.2127	0.00078072	−0.0009545	−0.008078
1956	−0.00054	0.001760	2.2204	0.00066706	0.00080425	−0.006352
1957	−0.00317	−0.00105	2.2186	0.00044173	0.00098396	−0.005064
1958	−0.00097	0.000618	2.2194	0.00036042	0.00091479	−0.003623
1959	−0.00139	−0.00018	2.2183	0.00022315	0.00068788	−0.002644
1960	−0.00060	0.000222	2.2181	0.00017179	0.00051852	−0.001843
1961	−0.00061	−1E−005	2.2176	0.000107	0.00036315	−0.001301
1962	−0.00032	8.3E−005	2.2175	7.9334E−005	0.00025988	−0.000898
1963	−0.00027	1.3E−005	2.2173	5.0477E−005	0.00017879	−0.000625
1964	−0.00016	3.3E−005	2.2172	3.6569E−005	0.00012559	−0.000430
1965	−0.00012	1.1E−005	2.2171	2.3753E−005	8.603E−005	−0.000298
1966	−8E−005	1.4E−005	2.217	1.6954E−005	5.995E−005	−0.000205
1967	−6E−005	6.4E−006	2.217	1.1193E−005	4.105E−005	−0.000141
1968	−4E−005	5.9E−006	2.217	7.9092E−006	2.848E−005	−9.7E−005
1969	−3E−005	3.4E−006	2.2169	5.283E−006	1.951E−005	−6.7E−005
1970	−2E−005	2.7E−006	2.2169	3.707E−006	1.351E−005	−4.6E−005

1971	−1E−005	1.7E−006	2.2169	2.4964E−006	9.26E−006	−3.2E−005
1972	−8E−006	1.2E−006	2.2169	1.743E−006	6.399E−006	−2.2E−005
1973	−6E−006	8.2E−007	2.2169	1.1804E−006	4.390E−006	−1.5E−005
1974	−4E−006	5.7E−007	2.2169	8.212E−007	3.030E−006	−1.0E−005
1975	−3E−006	3.9E−007	2.2169	5.583E−007	2.080E−006	−7.1E−006
1976	−2E−006	2.7E−007	2.2169	3.874E−007	1.434E−006	−4.9E−006
1977	−1E−006	1.9E−007	2.2169	2.641E−007	9.847E−007	−3.3E−006
1978	−9E−007	1.3E−007	2.2169	1.829E−007	6.788E−007	−2.3E−006
1979	−6E−007	8.9E−008	2.2169	1.2493E−007	4.661E−007	−1.6E−006
1980	−4E−007	6.0E−008	2.2169	8.6401E−008	3.212E−007	−1.1E−006
1981	−3E−007	4.2E−008	2.2169	5.91E−008	2.206E−007	−7.5E−007
1982	−2E−007	2.8E−008	2.2169	4.0828E−008	1.520E−007	−5.1E−007
1983	−1E−007	2.0E−008	2.2169	2.7957E−008	1.044E−007	−3.5E−007
1984	−9E−008	1.3E−008	2.2169	1.9298E−008	7.189E−008	−2.4E−007
1985	−6E−008	9.4E−009	2.2169	1.3225E−008	4.94E−008	−1.7E−007
1986	−4E−008	6.3E−009	2.2169	9.1226E−009	3.401E−008	−1.2E−007
1987	−3E−008	4.4E−009	2.2169	6.2556E−009	2.337E−008	−7.9E−008
1988	−2E−008	3.0E−009	2.2169	4.313E−009	1.609E−008	−5.4E−008
1989	−1E−008	2.1E−009	2.2169	2.959E−009	1.106E−008	−3.7E−008
1990	−1E−008	1.4E−009	2.2169	2.0392E−009	7.611E−009	−2.6E−008
1991	−7E−009	9.9E−010	2.2169	1.3996E−009	5.232E−009	−1.8E−008
1992	−5E−009	6.7E−010	2.2169	9.6429E−010	3.600E−009	−1.2E−008
1993	−3E−009	4.7E−010	2.2169	6.6202E−010	2.475E−009	−8.4E−009
1994	−2E−009	3.2E−010	2.2169	4.5597E−010	1.703E−009	−5.8E−009
1995	−2E−009	2.2E−010	2.2169	3.1311E−010	1.171E−009	−4.0E−009
1996	−1E−009	1.5E−010	2.2169	2.1562E−010	8.057E−010	−2.7E−009
1997	−7E−010	1.0E−010	2.2169	1.4814E−010	5.541E−010	−1.9E−009
1998	−5E−010	7.1E−011	2.2169	1.0204E−010	3.812E−010	−1.3E−009
1999	−3E−010	4.9E−011	2.2169	7.0054E−011	2.620E−010	−8.9E−010
2000	−2E−010	3.4E−011	2.2169	4.8262E−011	1.802E−010	−6.1E−010
2001	−2E−010	2.3E−011	2.2169	3.3107E−011	1.240E−010	−4.2E−010
2002	−1E−010	1.6E−011	2.2169	2.2831E−011	8.507E−011	−2.9E−010
2003	−8E−011	1.1E−011	2.2169	1.5736E−011	5.856E−011	−2.0E−010
2004	−5E−011	7.2E−012	2.2169	1.0838E−011	4.022E−011	−1.4E−010
2005	−4E−011	4.8E−012	2.2169	7.4074E−012	2.774E−011	−9.4E−011
2006	−2E−011	3.7E−012	2.2169	5.1062E−012	1.903E−011	−6.4E−011
2007	−2E−011	2.6E−012	2.2169	3.5027E−012	1.310E−011	−4.4E−011
2008	−1E−011	1.8E−012	2.2169	2.3314E−012	9.153E−012	−3.1E−011
2009	−8E−012	1.1E−012	2.2169	1.6508E−012	6.370E−012	−2.1E−011
2010	−6E−012	3.8E−013	2.2169	1.1672E−012	4.334E−012	−1.5E−011
2011	−4E−012	8.6E−013	2.2169	8.4298E−013	2.832E−012	−1.0E−011
2012	−3E−012	4.5E−013	2.2169	5.61E−013	2.117E−012	−6.8E−012
2013	−2E−012	4.0E−013	2.2169	3.5022E−013	1.439E−012	−4.7E−012
2014	−2E−012	4.0E−014	2.2169	2.7473E−013	1.089E−012	−3.2E−012
2015	−2E−012	−4E−013	2.2169	2.8513E−013	8.464E−013	−2.1E−012
2016	−1E−012	−1E−013	2.2169	2.0094E−013	2.712E−013	−1.4E−012
2017	−3E−013	2.7E−013	2.2169	1.7122E−013	−5.8E−014	−1.2E−012

2018	−8E−013	−4E−013	2.2169	3.7313E−014	−3.0E−013	−1.0E−013
2019	−7E−013	−2E−013	2.2169	6.8844E−014	1.001E−013	1.05E−013
2020	−2E−013	2.0E−013	2.2169	1.4389E−013	−8.8E−014	4.43E−013
2021	−6E−013	−7E−014	2.2169	5.2292E−014	−2.3E−013	9.35E−013
2022	−7E−013	1.7E−013	2.2169	1.95E−013	−1.6E−013	1.33E−012
2023	−1E−012	−2E−013	2.2169	2.6729E−013	−1.0E−012	1.91E−012
2024	−1E−012	2.2E−014	2.2169	2.1377E−013	−1.2E−012	2.59E−012
2025	−2E−012	6.0E−013	2.2169	2.7111E−013	−1.1E−012	4.50E−012
2026	−3E−012	2.3E−013	2.2169	5.3276E−013	−1.9E−012	6.45E−012
2027	−4E−012	6.4E−013	2.2169	7.9515E−013	−3.0E−012	1.02E−011
2028	−5E−012	9.4E−013	2.2169	1.1916E−012	−4.4E−012	1.45E−011
2029	−8E−012	1.6E−012	2.2169	1.761E−012	−6.4E−012	2.08E−011
2030	−1E−011	1.4E−012	2.2169	2.3152E−012	−9.1E−012	3.04E−011
2031	−2E−011	2.6E−012	2.2169	3.4542E−012	−1.3E−011	4.44E−011
2032	−2E−011	3.6E−012	2.2169	5.0726E−012	−1.9E−011	6.46E−011
2033	−4E−011	5.1E−012	2.2169	7.332E−012	−2.8E−011	9.36E−011
2034	−5E−011	7.8E−012	2.2169	1.0681E−011	−4.0E−011	1.36E−010
2035	−8E−011	1.1E−011	2.2169	1.5578E−011	−5.9E−011	1.98E−010
2036	−1E−010	1.6E−011	2.2169	2.276E−011	−8.5E−011	2.88E−010
2037	−2E−010	2.3E−011	2.2169	3.3206E−011	−1.2E−010	4.19E−010
2038	−2E−010	3.4E−011	2.2169	4.8211E−011	−1.8E−010	6.10E−010
2039	−3E−010	4.9E−011	2.2169	7.0158E−011	−2.6E−010	8.86E−010
2040	−5E−010	7.1E−011	2.2169	1.0193E−010	−3.8E−010	1.29E−009
2041	−7E−010	1.0E−010	2.2169	1.4809E−010	−5.5E−010	1.87E−009
2042	−1E−009	1.5E−010	2.2169	2.1557E−010	−8.1E−010	2.72E−009
2043	−2E−009	2.2E−010	2.2169	3.1322E−010	−1.2E−009	3.96E−009
2044	−2E−009	3.2E−010	2.2169	4.561E−010	−1.7E−009	5.76E−009
2045	−3E−009	4.7E−010	2.2169	6.6203E−010	−2.5E−009	8.38E−009
2046	−5E−009	6.7E−010	2.2169	9.6423E−010	−3.6E−009	1.22E−008
2047	−7E−009	9.9E−010	2.2169	1.3997E−009	−5.2E−009	1.77E−008
2048	−1E−008	1.4E−009	2.2169	2.0393E−009	−7.6E−009	2.57E−008
2049	−1E−008	2.1E−009	2.2169	2.9591E−009	−1.1E−008	3.74E−008
2050	−2E−008	3.0E−009	2.2169	4.313E−009	−1.6E−008	5.44E−008
2051	−3E−008	4.4E−009	2.2169	6.2556E−009	−2.3E−008	7.92E−008
2052	−4E−008	6.3E−009	2.2169	9.1225E−009	−3.4E−008	1.15E−007
2053	−6E−008	9.4E−009	2.2169	1.3225E−008	−4.9E−008	1.67E−007
2054	−9E−008	1.3E−008	2.2169	1.9297E−008	−7.2E−008	2.43E−007
2055	−1E−007	2.0E−008	2.2169	2.7957E−008	−1.0E−007	3.54E−007
2056	−2E−007	2.8E−008	2.2169	4.0828E−008	−1.5E−007	5.14E−007
2057	−3E−007	4.2E−008	2.2169	5.91E−008	−2.2E−007	7.48E−007
2058	−4E−007	6.0E−008	2.2169	8.6401E−008	−3.2E−007	1.09E−006
2059	−6E−007	8.9E−008	2.2169	1.2493E−007	−4.7E−007	1.58E−006
2060	−9E−007	1.3E−007	2.2169	1.829E−007	−6.8E−007	2.30E−006
2061	−1E−006	1.9E−007	2.2169	2.641E−007	−9.8E−007	3.35E−006
2062	−2E−006	2.7E−007	2.2169	3.874E−007	−1.4E−006	4.86E−006
2063	−3E−006	3.9E−007	2.2169	5.583E−007	−2.1E−006	7.07E−006
2064	−4E−006	5.7E−007	2.2169	8.212E−007	−3.0E−006	1.03E−005

2065	−6E−006	8.2E−007	2.2169	1.1804E−006	−4.4E−006	1.50E−005
2066	−8E−006	1.2E−006	2.2169	1.743E−006	−6.4E−006	2.17E−005
2067	−1E−005	1.7E−006	2.2169	2.4964E−006	−9.3E−006	3.16E−005
2068	−2E−005	2.7E−006	2.2169	3.707E−006	−1.4E−005	4.59E−005
2069	−3E−005	3.4E−006	2.2169	5.283E−006	−2.0E−005	6.68E−005
2070	−4E−005	5.9E−006	2.217	7.9092E−006	−2.8E−005	9.70E−005
2071	−6E−005	6.4E−006	2.217	1.1193E−005	−4.1E−005	0.00014114
2072	−8E−005	1.4E−005	2.217	1.6954E−005	−6.0E−005	0.00020459
2073	−0.00012	1.1E−005	2.2171	2.3753E−005	−8.6E−005	0.0002976
2074	−0.00016	3.3E−005	2.2172	3.6569E−005	−0.0001256	0.00043017
2075	−0.00027	1.3E−005	2.2173	5.0477E−005	−0.0001788	0.00062507
2076	−0.00032	8.3E−005	2.2175	7.9334E−005	−0.0002599	0.00089798
2077	−0.00061	−1E−005	2.2176	0.000107	−0.0003632	0.0013006
2078	−0.00060	0.000222	2.2181	0.00017179	−0.0005185	0.0018426
2079	−0.00139	−0.00018	2.2183	0.00022315	−0.0006879	0.0026441
2080	−0.00097	0.000618	2.2194	0.00036042	−0.0009148	0.0036227
2081	−0.00317	−0.00105	2.2186	0.00044173	−0.0009840	0.0050638
2082	−0.00054	0.001760	2.2204	0.00066706	−0.0008042	0.0063515
2083	−0.00702	−0.00539	2.2127	0.00078072	0.0009545	0.0080781
2084	0.0068781	0.005558	2.2062	0.00085584	0.005728	0.0075257
2085	−0.01675	−0.03145	2.1522	0.0017944	0.02513	0.0020775
2086	0.067165	0.024389	2.0678	0.0096304	0.067023	−0.028917
2087	0.23633	0.064305	2.1736	−0.0016231	−0.19118	−0.19831
2088	0.15843	−0.05350	2.1326	0.00055212	0.23496	0.10152
2089	0.086993	0.072788	1.9834	0.009555	−0.078823	0.011411
2090	−0.02898	−0.04364	2.1831	−0.0017188	−0.01464	−0.007067
2091	0.010956	0.013716	2.235	0.00090576	−0.0029938	−0.002597
2092	−0.00902	−0.00710	2.2375	6.5806E−005	−0.0016173	−0.004393
2093	−0.00115	0.002649	2.2352	0.00030426	0.00082705	−0.002929
2094	−0.00388	−0.00123	2.2306	0.00013859	0.00029046	−0.002642
2095	−0.00165	0.000664	2.2269	0.00014357	0.00060863	−0.001808
2096	−0.00179	−0.00022	2.224	8.1867E−005	0.00033844	−0.001392
2097	−0.00098	0.000205	2.2219	6.6889E−005	0.00031539	−0.000957
2098	−0.00083	−3E−005	2.2204	4.1507E−005	0.0002001	−0.000692
2099	−0.00050	7.2E−005	2.2193	3.082E−005	0.0001534	−0.000477
2100	−0.00038	2.4E−006	2.2186	2.0101E−005	0.00010317	−0.000335
2101	−0.00024	2.8E−005	2.2181	1.4252E−005	7.334E−005	−0.000231
2102	−0.00018	5.5E−006	2.2177	9.5775E−006	5.070E−005	−0.000160
2103	−0.00012	1.1E−005	2.2175	6.6378E−006	3.488E−005	−0.000111
2104	−8E−005	3.8E−006	2.2173	4.5367E−006	2.442E−005	−7.6E−005
2105	−6E−005	4.9E−006	2.2172	3.1104E−006	1.656E−005	−5.3E−005
2106	−4E−005	2.1E−006	2.2171	2.1446E−006	1.165E−005	−3.6E−005
2107	−3E−005	2.2E−006	2.217	1.4635E−006	7.856E−006	−2.5E−005
2108	−2E−005	1.1E−006	2.217	1.0132E−006	5.529E−006	−1.7E−005
2109	−1E−005	9.8E−007	2.217	6.9031E−007	3.725E−006	−1.2E−005
2110	−9E−006	5.4E−007	2.2169	4.7861E−007	2.619E−006	−8.1E−006
2111	−6E−006	4.5E−007	2.2169	3.2607E−007	1.766E−006	−5.6E−006

2112	−4E−006	2.6E−007	2.2169	2.261E−007	1.239E−006	−3.8E−006
2113	−3E−006	2.1E−007	2.2169	1.5414E−007	8.365E−007	−2.6E−006
2114	−2E−006	1.3E−007	2.2169	1.0683E−007	5.855E−007	−1.8E−006
2115	−1E−006	9.8E−008	2.2169	7.2893E−008	3.963E−007	−1.3E−006
2116	−9E−007	6.1E−008	2.2169	5.0483E−008	2.767E−007	−8.6E−007
2117	−6E−007	4.6E−008	2.2169	3.4478E−008	1.876E−007	−5.9E−007
2118	−4E−007	2.9E−008	2.2169	2.3859E−008	1.308E−007	−4.1E−007
2119	−3E−007	2.1E−008	2.2169	1.631E−008	8.884E−008	−2.8E−007
2120	−2E−007	1.4E−008	2.2169	1.1277E−008	6.179E−008	−1.9E−007
2121	−1E−007	1.0E−008	2.2169	7.7155E−009	4.205E−008	−1.3E−007
2122	−1E−007	6.6E−009	2.2169	5.3308E−009	2.920E−008	−9.1E−008
2123	−7E−008	4.8E−009	2.2169	3.6499E−009	1.990E−008	−6.3E−008
2124	−5E−008	3.1E−009	2.2169	2.5202E−009	1.380E−008	−4.3E−008
2125	−3E−008	2.2E−009	2.2169	1.7266E−009	9.419E−009	−3.0E−008
2126	−2E−008	1.5E−009	2.2169	1.1915E−009	6.523E−009	−2.0E−008
2127	−1E−008	1.1E−009	2.2169	8.1676E−010	4.457E−009	−1.4E−008
2128	−1E−008	7.0E−010	2.2169	5.6337E−010	3.083E−009	−9.6E−009
2129	−7E−009	5.0E−010	2.2169	3.8636E−010	2.109E−009	−6.6E−009
2130	−5E−009	3.3E−010	2.2169	2.6637E−010	1.458E−009	−4.5E−009
2131	−3E−009	2.3E−010	2.2169	1.8278E−010	9.976E−010	−3.1E−009
2132	−2E−009	1.6E−010	2.2169	1.2594E−010	6.892E−010	−2.2E−009
2133	−2E−009	1.1E−010	2.2169	8.647E−011	4.722E−010	−1.5E−009
2134	−1E−009	7.4E−011	2.2169	5.9563E−011	3.257E−010	−1.0E−009
2135	−7E−010	5.2E−011	2.2169	4.0897E−011	2.232E−010	−7E−010
2136	−5E−010	3.5E−011	2.2169	2.8155E−011	1.543E−010	−4.8E−010
2137	−4E−010	2.5E−011	2.2169	1.9366E−011	1.057E−010	−3.3E−010
2138	−2E−010	1.7E−011	2.2169	1.3385E−011	7.276E−011	−2.3E−010
2139	−2E−010	1.2E−011	2.2169	9.2033E−012	4.995E−011	−1.6E−010
2140	−1E−010	7.9E−012	2.2169	6.2952E−012	3.449E−011	−1.1E−010
2141	−8E−011	5.5E−012	2.2169	4.3614E−012	2.389E−011	−7.4E−011
2142	−5E−011	3.9E−012	2.2169	2.9243E−012	1.627E−011	−5.1E−011
2143	−4E−011	2.5E−012	2.2169	2.0433E−012	1.165E−011	−3.5E−011
2144	−3E−011	2.1E−012	2.2169	1.5054E−012	7.299E−012	−2.4E−011
2145	−2E−011	1.4E−012	2.2169	9.3362E−013	5.550E−012	−1.6E−011
2146	−1E−011	4.4E−013	2.2169	6.4858E−013	3.477E−012	−1.2E−011
2147	−8E−012	5.2E−013	2.2169	4.6816E−013	2.616E−012	−7.8E−012
2148	−6E−012	5.6E−013	2.2169	3.5364E−013	1.733E−012	−5.4E−012
2149	−4E−012	−4E−014	2.2169	2.1047E−013	1.009E−012	−3.8E−012
2150	−3E−012	1.7E−013	2.2169	1.2101E−013	8.971E−013	−2.5E−012
2151	−2E−012	3.3E−013	2.2169	1.3556E−013	2.445E−013	−1.5E−012
2152	−2E−012	7.6E−014	2.2169	9.4958E−014	2.927E−013	−1.2E−012
2153	−1E−012	1.1E−013	2.2169	3.3221E−014	−1.1E−013	−8.1E−013
2154	−8E−013	3.1E−013	2.2169	7.5938E−014	1.933E−013	−3.2E−013
2155	−9E−013	−1E−013	2.2169	2.1632E−014	−5.5E−014	1.01E−013
2156	−6E−013	−2E−013	2.2169	8.3309E−014	1.071E−014	3.43E−013
2157	−2E−013	3.6E−013	2.2169	3.0503E−014	1.654E−013	3.28E−013
2158	−6E−013	1.5E−013	2.2169	9.6922E−015	−6.7E−013	6.96E−013

2159	−9E−013	2.2E−013	2.2169	1.2835E−013	5.307E−014	8.91E−013
2160	−1E−012	1.3E−013	2.2169	1.3799E−013	−5.5E−013	1.13E−012
2161	−2E−012	6.9E−014	2.2169	1.1665E−013	−4.8E−013	1.67E−012
2162	−3E−012	−1E−013	2.2169	1.6501E−013	−1.0E−012	2.28E−012
2163	−4E−012	1E−013	2.2169	1.7678E−013	−8.0E−013	3.74E−012
2164	−6E−012	3.7E−013	2.2169	3.0164E−013	−1.9E−012	5.43E−012
2165	−8E−012	5.9E−013	2.2169	4.7157E−013	−2.3E−012	7.72E−012
2166	−1E−011	1E−012	2.2169	6.553E−013	−3.2E−012	1.14E−011
2167	−2E−011	9.1E−013	2.2169	8.8082E−013	−5.4E−012	1.68E−011
2168	−3E−011	1.9E−012	2.2169	1.3491E−012	−7.8E−012	2.47E−011
2169	−4E−011	2.1E−012	2.2169	2.1135E−012	−1.1E−011	3.49E−011
2170	−5E−011	3.6E−012	2.2169	3.0154E−012	−1.7E−011	5.07E−011
2171	−8E−011	5.5E−012	2.2169	4.2779E−012	−2.5E−011	7.40E−011
2172	−1E−010	7.6E−012	2.2169	6.3175E−012	−3.4E−011	1.08E−010
2173	−2E−010	1.2E−011	2.2169	9.2079E−012	−5.0E−011	1.57E−010
2174	−2E−010	1.7E−011	2.2169	1.3292E−011	−7.3E−011	2.28E−010
2175	−4E−010	2.5E−011	2.2169	1.936E−011	−1.1E−010	3.31E−010
2176	−5E−010	3.5E−011	2.2169	2.8164E−011	−1.5E−010	4.82E−010
2177	−7E−010	5.2E−011	2.2169	4.095E−011	−2.2E−010	7.00E−010
2178	−1E−009	7.4E−011	2.2169	5.962E−011	−3.3E−010	1.02E−009
2179	−2E−009	1.1E−010	2.2169	8.6494E−011	−4.7E−010	1.48E−009
2180	−2E−009	1.6E−010	2.2169	1.2594E−010	−6.9E−010	2.15E−009
2181	−3E−009	2.3E−010	2.2169	1.8268E−010	−1.0E−009	3.13E−009
2182	−5E−009	3.3E−010	2.2169	2.6641E−010	−1.5E−009	4.55E−009
2183	−7E−009	5.0E−010	2.2169	3.8625E−010	−2.1E−009	6.62E−009
2184	−1E−008	7.0E−010	2.2169	5.6341E−010	−3.1E−009	9.62E−009
2185	−1E−008	1.1E−009	2.2169	8.1675E−010	−4.5E−009	1.40E−008
2186	−2E−008	1.5E−009	2.2169	1.1915E−009	−6.5E−009	2.03E−008
2187	−3E−008	2.2E−009	2.2169	1.7267E−009	−9.4E−009	2.96E−008
2188	−5E−008	3.1E−009	2.2169	2.5203E−009	−1.4E−008	4.30E−008
2189	−7E−008	4.8E−009	2.2169	3.65E−009	−2.0E−008	6.26E−008
2190	−1E−007	6.6E−009	2.2169	5.331E−009	−2.9E−008	9.09E−008
2191	−1E−007	1.0E−008	2.2169	7.7155E−009	−4.2E−008	1.32E−007
2192	−2E−007	1.4E−008	2.2169	1.1277E−008	−6.2E−008	1.92E−007
2193	−3E−007	2.1E−008	2.2169	1.631E−008	−8.9E−008	2.80E−007
2194	−4E−007	2.9E−008	2.2169	2.3859E−008	−1.3E−007	4.06E−007
2195	−6E−007	4.6E−008	2.2169	3.4478E−008	−1.9E−007	5.91E−007
2196	−9E−007	6.1E−008	2.2169	5.0483E−008	−2.8E−007	8.59E−007
2197	−1E−006	9.8E−008	2.2169	7.2893E−008	−4.0E−007	1.25E−006
2198	−2E−006	1.3E−007	2.2169	1.0683E−007	−5.9E−007	1.82E−006
2199	−3E−006	2.1E−007	2.2169	1.5414E−007	−8.4E−007	2.64E−006
2200	−4E−006	2.6E−007	2.2169	2.261E−007	−1.2E−006	3.84E−006
2201	−6E−006	4.5E−007	2.2169	3.2607E−007	−1.8E−006	5.59E−006
2202	−9E−006	5.4E−007	2.2169	4.7861E−007	−2.6E−006	8.11E−006
2203	−1E−005	9.8E−007	2.217	6.9031E−007	−3.7E−006	1.18E−005
2204	−2E−005	1.1E−006	2.217	1.0132E−006	−5.5E−006	1.71E−005
2205	−3E−005	2.2E−006	2.217	1.4635E−006	−7.9E−006	2.50E−005

2206	−4E−005	2.1E−006	2.2171	2.1446E−006	−1.2E−005	3.62E−005
2207	−6E−005	4.9E−006	2.2172	3.1104E−006	−1.7E−005	5.26E−005
2208	−8E−005	3.8E−006	2.2173	4.5367E−006	−2.4E−005	7.63E−005
2209	−0.00012	1.1E−005	2.2175	6.6378E−006	−3.5E−005	0.00011066
2210	−0.00018	5.5E−006	2.2177	9.5775E−006	−5.1E−005	0.00016042
2211	−0.00024	2.8E−005	2.2181	1.4252E−005	−7.3E−005	0.00023124
2212	−0.00038	2.4E−006	2.2186	2.0101E−005	−0.0001032	0.00033535
2213	−0.00050	7.2E−005	2.2193	3.082E−005	−0.0001534	0.000477
2214	−0.00083	−3E−005	2.2204	4.1507E−005	−0.0002001	0.00069239
2215	−0.00098	0.000205	2.2219	6.6889E−005	−0.0003154	0.00095734
2216	−0.00179	−0.00022	2.224	8.1867E−005	−0.0003384	0.0013924
2217	−0.00165	0.000664	2.2269	0.00014357	−0.0006086	0.0018082
2218	−0.00388	−0.00123	2.2306	0.00013859	−0.0002905	0.0026419
2219	−0.00115	0.002649	2.2352	0.00030426	−0.0008270	0.0029294
2220	−0.00902	−0.00710	2.2375	6.5806E−005	0.0016173	0.004393
2221	0.010956	0.013716	2.235	0.00090576	0.0029938	0.0025968
2222	−0.02898	−0.04364	2.1831	−0.0017188	0.01464	0.0070673
2223	0.086993	0.072788	1.9834	0.009555	0.078823	−0.011411
2224	0.15843	−0.05350	2.1326	0.00055212	−0.23496	−0.10152
2225	2.127	2.142	2.3845	−0.0058906	0.58041	−0.13816
2226	1.9396	1.9293	2.1942	−0.017601	0.45971	−0.31364
2227	2.0215	1.9757	2.281	−0.016543	0.31923	−0.49178
2228	2.3204	2.2445	2.5891	0.00035693	0.14822	−0.68826
2229	2.127	2.142	2.3845	−0.0058906	−0.58041	0.13816
2230	1.9396	1.9293	2.1942	−0.017601	−0.45971	0.31364
2231	2.0215	1.9757	2.281	−0.016543	−0.31923	0.49178
2232	2.3204	2.2445	2.5891	0.00035693	−0.14822	0.68826
2233	0.15843	−0.05350	2.1326	−0.00055212	0.23496	−0.10152
2234	0.086993	0.072788	1.9834	−0.009555	−0.078823	−0.011411
2235	−0.02898	−0.04364	2.1831	0.0017188	−0.01464	0.0070673
2236	0.010956	0.013716	2.235	−0.00090576	−0.0029938	0.0025968
2237	−0.00902	−0.00710	2.2375	−6.5806E−005	−0.0016173	0.004393
2238	−0.00115	0.002649	2.2352	−0.00030426	0.00082705	0.0029294
2239	−0.00388	−0.00123	2.2306	−0.00013859	0.00029046	0.0026419
2240	−0.00165	0.000664	2.2269	−0.00014357	0.00060863	0.0018082
2241	−0.00179	−0.00022	2.224	−8.1867E−005	0.00033844	0.0013924
2242	−0.00098	0.000205	2.2219	−6.6889E−005	0.00031539	0.00095734
2243	−0.00083	−3E−005	2.2204	−4.1507E−005	0.0002001	0.00069239
2244	−0.00050	7.2E−005	2.2193	−3.082E−005	0.0001534	0.000477
2245	−0.00038	2.4E−006	2.2186	−2.0101E−005	0.00010317	0.00033535
2246	−0.00024	2.8E−005	2.2181	−1.4252E−005	7.334E−005	0.00023124
2247	−0.00018	5.5E−006	2.2177	−9.5775E−006	5.070E−005	0.00016042
2248	−0.00012	1.1E−005	2.2175	−6.6378E−006	3.488E−005	0.00011066
2249	−8E−005	3.8E−006	2.2173	−4.5367E−006	2.442E−005	7.63E−005
2250	−6E−005	4.9E−006	2.2172	−3.1104E−006	1.656E−005	5.26E−005
2251	−4E−005	2.1E−006	2.2171	−2.1446E−006	1.165E−005	3.62E−005
2252	−3E−005	2.2E−006	2.217	−1.4635E−006	7.856E−006	2.50E−005

2253	−2E−005	1.1E−006	2.217	−1.0132E−006	5.529E−006	1.71E−005
2254	−1E−005	9.8E−007	2.217	−6.9031E−007	3.725E−006	1.18E−005
2255	−9E−006	5.4E−007	2.2169	−4.7861E−007	2.619E−006	8.11E−006
2256	−6E−006	4.5E−007	2.2169	−3.2607E−007	1.766E−006	5.59E−006
2257	−4E−006	2.6E−007	2.2169	−2.261E−007	1.239E−006	3.84E−006
2258	−3E−006	2.1E−007	2.2169	−1.5414E−007	8.365E−007	2.64E−006
2259	−2E−006	1.3E−007	2.2169	−1.0683E−007	5.855E−007	1.82E−006
2260	−1E−006	9.8E−008	2.2169	−7.2893E−008	3.963E−007	1.25E−006
2261	−9E−007	6.1E−008	2.2169	−5.0483E−008	2.767E−007	8.59E−007
2262	−6E−007	4.6E−008	2.2169	−3.4478E−008	1.876E−007	5.91E−007
2263	−4E−007	2.9E−008	2.2169	−2.3859E−008	1.308E−007	4.06E−007
2264	−3E−007	2.1E−008	2.2169	−1.631E−008	8.884E−008	2.80E−007
2265	−2E−007	1.4E−008	2.2169	−1.1277E−008	6.179E−008	1.92E−007
2266	−1E−007	1.0E−008	2.2169	−7.7155E−009	4.205E−008	1.32E−007
2267	−1E−007	6.6E−009	2.2169	−5.3309E−009	2.920E−008	9.09E−008
2268	−7E−008	4.8E−009	2.2169	−3.6499E−009	1.990E−008	6.26E−008
2269	−5E−008	3.1E−009	2.2169	−2.5202E−009	1.380E−008	4.30E−008
2270	−3E−008	2.2E−009	2.2169	−1.7267E−009	9.419E−009	2.96E−008
2271	−2E−008	1.5E−009	2.2169	−1.1915E−009	6.523E−009	2.03E−008
2272	−1E−008	1.1E−009	2.2169	−8.1682E−010	4.457E−009	1.40E−008
2273	−1E−008	7.0E−010	2.2169	−5.6334E−010	3.084E−009	9.62E−009
2274	−7E−009	5.0E−010	2.2169	−3.864E−010	2.109E−009	6.62E−009
2275	−5E−009	3.3E−010	2.2169	−2.6636E−010	1.458E−009	4.55E−009
2276	−3E−009	2.3E−010	2.2169	−1.8271E−010	9.976E−010	3.13E−009
2277	−2E−009	1.6E−010	2.2169	−1.2594E−010	6.895E−010	2.15E−009
2278	−2E−009	1.1E−010	2.2169	−8.6434E−011	4.719E−010	1.48E−009
2279	−1E−009	7.4E−011	2.2169	−5.9574E−011	3.259E−010	1.02E−009
2280	−7E−010	5.2E−011	2.2169	−4.0894E−011	2.231E−010	7.00E−010
2281	−5E−010	3.5E−011	2.2169	−2.8192E−011	1.542E−010	4.81E−010
2282	−4E−010	2.5E−011	2.2169	−1.9351E−011	1.056E−010	3.31E−010
2283	−2E−010	1.7E−011	2.2169	−1.338E−011	7.274E−011	2.28E−010
2284	−2E−010	1.2E−011	2.2169	−9.259E−012	4.971E−011	1.57E−010
2285	−1E−010	8.1E−012	2.2169	−6.3065E−012	3.463E−011	1.08E−010
2286	−8E−011	5.3E−012	2.2169	−4.3316E−012	2.345E−011	7.40E−011
2287	−5E−011	3.6E−012	2.2169	−2.9734E−012	1.677E−011	5.10E−011
2288	−4E−011	2.5E−012	2.2169	−2.0584E−012	1.112E−011	3.46E−011
2289	−3E−011	1.7E−012	2.2169	−1.4189E−012	7.885E−012	2.41E−011
2290	−2E−011	1.6E−012	2.2169	−1.0435E−012	5.267E−012	1.63E−011
2291	−1E−011	5.2E−013	2.2169	−6.3839E−013	3.667E−012	1.17E−011
2292	−9E−012	4.7E−013	2.2169	−4.8016E−013	2.375E−012	8.01E−012
2293	−5E−012	6.9E−013	2.2169	−3.1216E−013	1.845E−012	5.57E−012
2294	−4E−012	2.4E−013	2.2169	−2.0779E−013	1.028E−012	3.78E−012
2295	−3E−012	2E−013	2.2169	−1.0423E−013	8.969E−013	2.48E−012
2296	−2E−012	−9E−014	2.2169	−6.1485E−014	2.686E−013	1.32E−012
2297	−2E−012	−1E−013	2.2169	−1.1801E−013	1.279E−013	1.10E−012
2298	−1E−012	1.3E−013	2.2169	−1.2286E−013	2.223E−013	6.91E−013
2299	−8E−013	1.3E−013	2.2169	−6.1658E−014	3.466E−013	3.87E−013

2300	−1E−012	−3E−013	2.2169	−6.2472E−014	−2.6E−013	−1.2E−013
2301	−4E−013	8.0E−014	2.2169	−7.0141E−014	3.229E−013	−2.1E−013
2302	−5E−013	2.8E−013	2.2169	3.2389E−014	2.788E−013	−3.0E−013
2303	−1E−012	−4E−014	2.2169	−4.3939E−014	1.03E−013	−3.8E−013
2304	−9E−013	2.0E−013	2.2169	−5.2216E−014	−5.5E−013	−1.1E−012
2305	−1E−012	2.3E−013	2.2169	−1.1549E−013	4.302E−014	−1.0E−012
2306	−2E−012	−2E−013	2.2169	−1.3639E−013	−1.1E−012	−1.7E−012
2307	−3E−012	−2E−013	2.2169	−1.6466E−013	−4.5E−013	−2.1E−012
2308	−4E−012	5.0E−014	2.2169	−1.695E−013	−9.5E−013	−3.4E−012
2309	−6E−012	2.7E−013	2.2169	−2.7945E−013	−1.6E−012	−5.5E−012
2310	−8E−012	6.3E−013	2.2169	−4.5781E−013	−2.4E−012	−7.8E−012
2311	−1E−011	5.4E−013	2.2169	−6.7971E−013	−3.1E−012	−1.2E−011
2312	−2E−011	8.4E−013	2.2169	−9.2527E−013	−5.8E−012	−1.6E−011
2313	−3E−011	1.5E−012	2.2169	−1.3271E−012	−7.5E−012	−2.4E−011
2314	−4E−011	2.7E−012	2.2169	−1.9984E−012	−1.2E−011	−3.5E−011
2315	−5E−011	3.7E−012	2.2169	−3.0306E−012	−1.6E−011	−5.1E−011
2316	−8E−011	5.5E−012	2.2169	−4.2866E−012	−2.4E−011	−7.4E−011
2317	−1E−010	7.4E−012	2.2169	−6.3206E−012	−3.4E−011	−1.1E−010
2318	−2E−010	1.2E−011	2.2169	−9.1827E−012	−5.0E−011	−1.6E−010
2319	−2E−010	1.6E−011	2.2169	−1.3322E−011	−7.3E−011	−2.3E−010
2320	−4E−010	2.5E−011	2.2169	−1.9291E−011	−1.1E−010	−3.3E−010
2321	−5E−010	3.5E−011	2.2169	−2.8176E−011	−1.5E−010	−4.8E−010
2322	−7E−010	5.2E−011	2.2169	−4.0866E−011	−2.2E−010	−7E−010
2323	−1E−009	7.5E−011	2.2169	−5.9698E−011	−3.3E−010	−1.0E−009
2324	−2E−009	1.1E−010	2.2169	−8.6438E−011	−4.7E−010	−1.5E−009
2325	−2E−009	1.6E−010	2.2169	−1.2594E−010	−6.9E−010	−2.2E−009
2326	−3E−009	2.4E−010	2.2169	−1.8273E−010	−1.0E−009	−3.1E−009
2327	−5E−009	3.3E−010	2.2169	−2.664E−010	−1.5E−009	−4.5E−009
2328	−7E−009	5.0E−010	2.2169	−3.8638E−010	−2.1E−009	−6.6E−009
2329	−1E−008	7.0E−010	2.2169	−5.6338E−010	−3.1E−009	−9.6E−009
2330	−1E−008	1.1E−009	2.2169	−8.1672E−010	−4.5E−009	−1.4E−008
2331	−2E−008	1.5E−009	2.2169	−1.1916E−009	−6.5E−009	−2.0E−008
2332	−3E−008	2.2E−009	2.2169	−1.7267E−009	−9.4E−009	−3.0E−008
2333	−5E−008	3.1E−009	2.2169	−2.5202E−009	−1.4E−008	−4.3E−008
2334	−7E−008	4.8E−009	2.2169	−3.65E−009	−2.0E−008	−6.3E−008
2335	−1E−007	6.6E−009	2.2169	−5.3309E−009	−2.9E−008	−9.1E−008
2336	−1E−007	1.0E−008	2.2169	−7.7155E−009	−4.2E−008	−1.3E−007
2337	−2E−007	1.4E−008	2.2169	−1.1277E−008	−6.2E−008	−1.9E−007
2338	−3E−007	2.1E−008	2.2169	−1.631E−008	−8.9E−008	−2.8E−007
2339	−4E−007	2.9E−008	2.2169	−2.3859E−008	−1.3E−007	−4.1E−007
2340	−6E−007	4.6E−008	2.2169	−3.4478E−008	−1.9E−007	−5.9E−007
2341	−9E−007	6.1E−008	2.2169	−5.0483E−008	−2.8E−007	−8.6E−007
2342	−1E−006	9.8E−008	2.2169	−7.2893E−008	−4.0E−007	−1.3E−006
2343	−2E−006	1.3E−007	2.2169	−1.0683E−007	−5.9E−007	−1.8E−006
2344	−3E−006	2.1E−007	2.2169	−1.5414E−007	−8.4E−007	−2.6E−006
2345	−4E−006	2.6E−007	2.2169	−2.261E−007	−1.2E−006	−3.8E−006
2346	−6E−006	4.5E−007	2.2169	−3.2607E−007	−1.8E−006	−5.6E−006

2347	−9E−006	5.4E−007	2.2169	−4.7861E−007	−2.6E−006	−8.1E−006
2348	−1E−005	9.8E−007	2.217	−6.9031E−007	−3.7E−006	−1.2E−005
2349	−2E−005	1.1E−006	2.217	−1.0132E−006	−5.5E−006	−1.7E−005
2350	−3E−005	2.2E−006	2.217	−1.4635E−006	−7.9E−006	−2.5E−005
2351	−4E−005	2.1E−006	2.2171	−2.1446E−006	−1.2E−005	−3.6E−005
2352	−6E−005	4.9E−006	2.2172	−3.1104E−006	−1.7E−005	−5.3E−005
2353	−8E−005	3.8E−006	2.2173	−4.5367E−006	−2.4E−005	−7.6E−005
2354	−0.00012	1.1E−005	2.2175	−6.6378E−006	−3.5E−005	−0.000111
2355	−0.00018	5.5E−006	2.2177	−9.5775E−006	−5.1E−005	−0.000160
2356	−0.00024	2.8E−005	2.2181	−1.4252E−005	−7.3E−005	−0.000231
2357	−0.00038	2.4E−006	2.2186	−2.0101E−005	−0.0001032	−0.000335
2358	−0.00050	7.2E−005	2.2193	−3.082E−005	−0.0001534	−0.000477
2359	−0.00083	−3E−005	2.2204	−4.1507E−005	−0.0002001	−0.000692
2360	−0.00098	0.000205	2.2219	−6.6889E−005	−0.0003154	−0.000957
2361	−0.00179	−0.00022	2.224	−8.1867E−005	−0.0003384	−0.001392
2362	−0.00165	0.000664	2.2269	−0.00014357	−0.0006086	−0.001808
2363	−0.00388	−0.00123	2.2306	−0.00013859	−0.0002905	−0.002642
2364	−0.00115	0.002649	2.2352	−0.00030426	−0.0008270	−0.002929
2365	−0.00902	−0.00710	2.2375	−6.5806E−005	0.0016173	−0.004393
2366	0.010956	0.013716	2.235	−0.00090576	0.0029938	−0.002597
2367	−0.02898	−0.04364	2.1831	0.0017188	0.01464	−0.007067
2368	0.086993	0.072788	1.9834	−0.009555	0.078823	0.011411
2369	0.15843	−0.05350	2.1326	−0.00055212	−0.23496	0.10152
2370	0.23633	0.064305	2.1736	0.0016231	0.19118	−0.19831
2371	0.067165	0.024389	2.0678	−0.0096304	−0.067023	−0.028917
2372	−0.01675	−0.03145	2.1522	−0.0017944	−0.02513	0.0020775
2373	0.0068781	0.005558	2.2062	−0.00085584	−0.005728	0.0075257
2374	−0.00702	−0.00539	2.2127	−0.00078072	−0.0009545	0.0080781
2375	−0.00054	0.001760	2.2204	−0.00066706	0.00080425	0.0063515
2376	−0.00317	−0.00105	2.2186	−0.00044173	0.00098396	0.0050638
2377	−0.00097	0.000618	2.2194	−0.00036042	0.00091479	0.0036227
2378	−0.00139	−0.00018	2.2183	−0.00022315	0.00068788	0.0026441
2379	−0.00060	0.000222	2.2181	−0.00017179	0.00051852	0.0018426
2380	−0.00061	−1E−005	2.2176	−0.000107	0.00036315	0.0013006
2381	−0.00032	8.3E−005	2.2175	−7.9334E−005	0.00025988	0.00089798
2382	−0.00027	1.3E−005	2.2173	−5.0477E−005	0.00017879	0.00062507
2383	−0.00016	3.3E−005	2.2172	−3.6569E−005	0.00012559	0.00043017
2384	−0.00012	1.1E−005	2.2171	−2.3753E−005	8.603E−005	0.0002976
2385	−8E−005	1.4E−005	2.217	−1.6954E−005	5.995E−005	0.00020459
2386	−6E−005	6.4E−006	2.217	−1.1193E−005	4.105E−005	0.00014114
2387	−4E−005	5.9E−006	2.217	−7.9092E−006	2.848E−005	9.70E−005
2388	−3E−005	3.4E−006	2.2169	−5.283E−006	1.951E−005	6.68E−005
2389	−2E−005	2.7E−006	2.2169	−3.707E−006	1.351E−005	4.59E−005
2390	−1E−005	1.7E−006	2.2169	−2.4964E−006	9.26E−006	3.16E−005
2391	−8E−006	1.2E−006	2.2169	−1.743E−006	6.399E−006	2.17E−005
2392	−6E−006	8.2E−007	2.2169	−1.1804E−006	4.390E−006	1.50E−005
2393	−4E−006	5.7E−007	2.2169	−8.212E−007	3.030E−006	1.03E−005

2394	−3E−006	3.9E−007	2.2169	−5.583E−007	2.080E−006	7.07E−006
2395	−2E−006	2.7E−007	2.2169	−3.874E−007	1.434E−006	4.86E−006
2396	−1E−006	1.9E−007	2.2169	−2.641E−007	9.847E−007	3.35E−006
2397	−9E−007	1.3E−007	2.2169	−1.829E−007	6.788E−007	2.30E−006
2398	−6E−007	8.9E−008	2.2169	−1.2493E−007	4.661E−007	1.58E−006
2399	−4E−007	6.0E−008	2.2169	−8.6401E−008	3.212E−007	1.09E−006
2400	−3E−007	4.2E−008	2.2169	−5.91E−008	2.206E−007	7.48E−007
2401	−2E−007	2.8E−008	2.2169	−4.0828E−008	1.520E−007	5.14E−007
2402	−1E−007	2.0E−008	2.2169	−2.7957E−008	1.044E−007	3.54E−007
2403	−9E−008	1.3E−008	2.2169	−1.9298E−008	7.189E−008	2.43E−007
2404	−6E−008	9.4E−009	2.2169	−1.3225E−008	4.94E−008	1.67E−007
2405	−4E−008	6.3E−009	2.2169	−9.1225E−009	3.401E−008	1.15E−007
2406	−3E−008	4.4E−009	2.2169	−6.2556E−009	2.337E−008	7.92E−008
2407	−2E−008	3.0E−009	2.2169	−4.313E−009	1.609E−008	5.44E−008
2408	−1E−008	2.1E−009	2.2169	−2.959E−009	1.106E−008	3.74E−008
2409	−1E−008	1.4E−009	2.2169	−2.0393E−009	7.611E−009	2.57E−008
2410	−7E−009	9.9E−010	2.2169	−1.3996E−009	5.232E−009	1.77E−008
2411	−5E−009	6.7E−010	2.2169	−9.6427E−010	3.600E−009	1.22E−008
2412	−3E−009	4.7E−010	2.2169	−6.6204E−010	2.475E−009	8.38E−009
2413	−2E−009	3.2E−010	2.2169	−4.5599E−010	1.703E−009	5.76E−009
2414	−2E−009	2.2E−010	2.2169	−3.1319E−010	1.171E−009	3.96E−009
2415	−1E−009	1.5E−010	2.2169	−2.1559E−010	8.057E−010	2.72E−009
2416	−7E−010	1.0E−010	2.2169	−1.4816E−010	5.541E−010	1.87E−009
2417	−5E−010	7.1E−011	2.2169	−1.0199E−010	3.811E−010	1.29E−009
2418	−3E−010	5.0E−011	2.2169	−7.0075E−011	2.622E−010	8.86E−010
2419	−2E−010	3.4E−011	2.2169	−4.8161E−011	1.803E−010	6.09E−010
2420	−2E−010	2.3E−011	2.2169	−3.3155E−011	1.240E−010	4.19E−010
2421	−1E−010	1.6E−011	2.2169	−2.2828E−011	8.514E−011	2.88E−010
2422	−8E−011	1.1E−011	2.2169	−1.5553E−011	5.858E−011	1.98E−010
2423	−5E−011	7.4E−012	2.2169	−1.0727E−011	4.034E−011	1.36E−010
2424	−4E−011	5.3E−012	2.2169	−7.3719E−012	2.782E−011	9.37E−011
2425	−2E−011	3.7E−012	2.2169	−5.1048E−012	1.898E−011	6.45E−011
2426	−2E−011	2.6E−012	2.2169	−3.4891E−012	1.317E−011	4.42E−011
2427	−1E−011	1.5E−012	2.2169	−2.4164E−012	8.981E−012	3.06E−011
2428	−8E−012	1.2E−012	2.2169	−1.6729E−012	6.153E−012	2.09E−011
2429	−6E−012	6.0E−013	2.2169	−1.1485E−012	4.278E−012	1.42E−011
2430	−4E−012	5.4E−013	2.2169	−7.6821E−013	3.063E−012	1.01E−011
2431	−2E−012	5.7E−013	2.2169	−5.3407E−013	1.793E−012	6.93E−012
2432	−2E−012	1.8E−013	2.2169	−3.2397E−013	1.602E−012	4.56E−012
2433	−1E−012	8.1E−015	2.2169	−2.6443E−013	9.758E−013	3.13E−012
2434	−1E−012	4.9E−014	2.2169	−2.5224E−013	4.038E−013	2.25E−012
2435	−7E−013	1.7E−013	2.2169	−1.3519E−013	4.205E−013	1.41E−012
2436	−1E−012	−3E−013	2.2169	−8.396E−014	3.017E−013	9.4E−013
2437	−7E−013	−9E−014	2.2169	−1.4188E−013	−1.7E−013	1.05E−013
2438	−6E−013	−1E−013	2.2169	−6.7696E−014	−1.1E−013	−7E−014
2439	−4E−013	−8E−014	2.2169	−6.6829E−014	7.723E−014	−3.3E−013
2440	−5E−013	3.5E−013	2.2169	−1.3854E−013	−1.3E−013	−8.4E−013

2441	−7E−013	−2E−013	2.2169	−2.1249E−013	−6.4E−013	−1.3E−012
2442	−8E−013	1.0E−013	2.2169	−1.6135E−013	−7.8E−013	−2.0E−012
2443	−1E−012	1.9E−013	2.2169	−2.4077E−013	−6.9E−013	−3.1E−012
2444	−2E−012	3.4E−013	2.2169	−4.7592E−013	−1.2E−012	−4.8E−012
2445	−3E−012	5.2E−013	2.2169	−5.0141E−013	−2.1E−012	−6.5E−012
2446	−4E−012	5.9E−013	2.2169	−7.7039E−013	−3.1E−012	−9.8E−012
2447	−6E−012	9.5E−013	2.2169	−1.115E−012	−3.9E−012	−1.4E−011
2448	−8E−012	1.1E−012	2.2169	−1.7401E−012	−6.4E−012	−2.1E−011
2449	−1E−011	1.6E−012	2.2169	−2.4665E−012	−9.0E−012	−3.1E−011
2450	−2E−011	1.8E−012	2.2169	−3.5047E−012	−1.3E−011	−4.4E−011
2451	−2E−011	3.4E−012	2.2169	−5.0607E−012	−1.9E−011	−6.5E−011
2452	−4E−011	4.9E−012	2.2169	−7.4623E−012	−2.7E−011	−9.4E−011
2453	−5E−011	7.3E−012	2.2169	−1.08E−011	−4.0E−011	−1.4E−010
2454	−8E−011	1.1E−011	2.2169	−1.5714E−011	−5.8E−011	−2.0E−010
2455	−1E−010	1.6E−011	2.2169	−2.2791E−011	−8.6E−011	−2.9E−010
2456	−2E−010	2.3E−011	2.2169	−3.3164E−011	−1.2E−010	−4.2E−010
2457	−2E−010	3.4E−011	2.2169	−4.8239E−011	−1.8E−010	−6.1E−010
2458	−3E−010	4.9E−011	2.2169	−7.0028E−011	−2.6E−010	−8.9E−010
2459	−5E−010	7.1E−011	2.2169	−1.0188E−010	−3.8E−010	−1.3E−009
2460	−7E−010	1.0E−010	2.2169	−1.4812E−010	−5.5E−010	−1.9E−009
2461	−1E−009	1.5E−010	2.2169	−2.1563E−010	−8.1E−010	−2.7E−009
2462	−2E−009	2.2E−010	2.2169	−3.1313E−010	−1.2E−009	−4.0E−009
2463	−2E−009	3.2E−010	2.2169	−4.561E−010	−1.7E−009	−5.8E−009
2464	−3E−009	4.7E−010	2.2169	−6.6208E−010	−2.5E−009	−8.4E−009
2465	−5E−009	6.7E−010	2.2169	−9.6434E−010	−3.6E−009	−1.2E−008
2466	−7E−009	9.9E−010	2.2169	−1.3996E−009	−5.2E−009	−1.8E−008
2467	−1E−008	1.4E−009	2.2169	−2.0392E−009	−7.6E−009	−2.6E−008
2468	−1E−008	2.1E−009	2.2169	−2.9589E−009	−1.1E−008	−3.7E−008
2469	−2E−008	3.0E−009	2.2169	−4.3129E−009	−1.6E−008	−5.4E−008
2470	−3E−008	4.4E−009	2.2169	−6.2557E−009	−2.3E−008	−7.9E−008
2471	−4E−008	6.3E−009	2.2169	−9.1226E−009	−3.4E−008	−1.2E−007
2472	−6E−008	9.4E−009	2.2169	−1.3225E−008	−4.9E−008	−1.7E−007
2473	−9E−008	1.3E−008	2.2169	−1.9298E−008	−7.2E−008	−2.4E−007
2474	−1E−007	2.0E−008	2.2169	−2.7957E−008	−1.0E−007	−3.5E−007
2475	−2E−007	2.8E−008	2.2169	−4.0828E−008	−1.5E−007	−5.1E−007
2476	−3E−007	4.2E−008	2.2169	−5.91E−008	−2.2E−007	−7.5E−007
2477	−4E−007	6.0E−008	2.2169	−8.6401E−008	−3.2E−007	−1.1E−006
2478	−6E−007	8.9E−008	2.2169	−1.2493E−007	−4.7E−007	−1.6E−006
2479	−9E−007	1.3E−007	2.2169	−1.829E−007	−6.8E−007	−2.3E−006
2480	−1E−006	1.9E−007	2.2169	−2.641E−007	−9.8E−007	−3.3E−006
2481	−2E−006	2.7E−007	2.2169	−3.874E−007	−1.4E−006	−4.9E−006
2482	−3E−006	3.9E−007	2.2169	−5.583E−007	−2.1E−006	−7.1E−006
2483	−4E−006	5.7E−007	2.2169	−8.212E−007	−3.0E−006	−1.0E−005
2484	−6E−006	8.2E−007	2.2169	−1.1804E−006	−4.4E−006	−1.5E−005
2485	−8E−006	1.2E−006	2.2169	−1.743E−006	−6.4E−006	−2.2E−005
2486	−1E−005	1.7E−006	2.2169	−2.4964E−006	−9.3E−006	−3.2E−005
2487	−2E−005	2.7E−006	2.2169	−3.707E−006	−1.4E−005	−4.6E−005

2488	−3E−005	3.4E−006	2.2169	−5.283E−006	−2.0E−005	−6.7E−005
2489	−4E−005	5.9E−006	2.217	−7.9092E−006	−2.8E−005	−9.7E−005
2490	−6E−005	6.4E−006	2.217	−1.1193E−005	−4.1E−005	−0.000141
2491	−8E−005	1.4E−005	2.217	−1.6954E−005	−6.0E−005	−0.000205
2492	−0.00012	1.1E−005	2.2171	−2.3753E−005	−8.6E−005	−0.000298
2493	−0.00016	3.3E−005	2.2172	−3.6569E−005	−0.0001256	−0.000430
2494	−0.00027	1.3E−005	2.2173	−5.0477E−005	−0.0001788	−0.000625
2495	−0.00032	8.3E−005	2.2175	−7.9334E−005	−0.0002599	−0.000898
2496	−0.00061	−1E−005	2.2176	−0.000107	−0.0003632	−0.001301
2497	−0.00060	0.000222	2.2181	−0.00017179	−0.0005185	−0.001843
2498	−0.00139	−0.00018	2.2183	−0.00022315	−0.0006879	−0.002644
2499	−0.00097	0.000618	2.2194	−0.00036042	−0.0009148	−0.003623
2500	−0.00317	−0.00105	2.2186	−0.00044173	−0.0009840	−0.005064
2501	−0.00054	0.001760	2.2204	−0.00066706	−0.0008042	−0.006352
2502	−0.00702	−0.00539	2.2127	−0.00078072	0.0009545	−0.008078
2503	0.0068781	0.005558	2.2062	−0.00085584	0.005728	−0.007526
2504	−0.01675	−0.03145	2.1522	−0.0017944	0.02513	−0.002077
2505	0.067165	0.024389	2.0678	−0.0096304	0.067023	0.028917
2506	0.23633	0.064305	2.1736	0.0016231	−0.19118	0.19831
2507	0.21345	0.081784	2.2513	0.011031	0.13622	−0.29124
2508	0.060004	0.015744	2.0938	−0.010971	−0.049702	−0.032701
2509	−0.01477	−0.03114	2.135	−0.0029355	−0.01726	0.0022618
2510	0.0087819	0.004722	2.1721	−0.0015101	−0.0043947	0.008668
2511	−0.00527	−0.00576	2.184	−0.00088875	−0.0003996	0.0091478
2512	0.0005965	0.001551	2.197	−0.00070191	0.0012801	0.0073186
2513	−0.00218	−0.00128	2.2022	−0.00040918	0.001229	0.0055141
2514	−0.00037	0.000511	2.2074	−0.00030054	0.0011464	0.0039202
2515	−0.00090	−0.00031	2.21	−0.00017987	0.00081889	0.002763
2516	−0.00030	0.000164	2.2123	−0.0001281	0.00062315	0.0019061
2517	−0.00038	−7E−005	2.2136	−7.9421E−005	0.00042585	0.0013197
2518	−0.00017	5.3E−005	2.2147	−5.582E−005	0.0003057	0.00090401
2519	−0.00016	−2E−005	2.2154	−3.5786E−005	0.00020756	0.00062293
2520	−9E−005	1.7E−005	2.2158	−2.4969E−005	0.00014583	0.00042652
2521	−7E−005	−3E−006	2.2162	−1.643E−005	9.920E−005	0.0002936
2522	−4E−005	6.1E−006	2.2164	−1.1404E−005	6.908E−005	0.00020122
2523	−3E−005	−2E−007	2.2166	−7.6398E−006	4.713E−005	0.00013849
2524	−2E−005	2.3E−006	2.2167	−5.2819E−006	3.268E−005	9.50E−005
2525	−2E−005	2.8E−007	2.2167	−3.5795E−006	2.235E−005	6.54E−005
2526	−1E−005	9.0E−007	2.2168	−2.4675E−006	1.546E−005	4.49E−005
2527	−7E−006	2.4E−007	2.2168	−1.6842E−006	1.059E−005	3.09E−005
2528	−5E−006	3.8E−007	2.2169	−1.1587E−006	7.317E−006	2.12E−005
2529	−3E−006	1.5E−007	2.2169	−7.9419E−007	5.017E−006	1.46E−005
2530	−2E−006	1.6E−007	2.2169	−5.4571E−007	3.463E−006	1.00E−005
2531	−2E−006	7.8E−008	2.2169	−3.7496E−007	2.376E−006	6.90E−006
2532	−1E−006	7.4E−008	2.2169	−2.5746E−007	1.639E−006	4.74E−006
2533	−7E−007	4E−008	2.2169	−1.7714E−007	1.125E−006	3.26E−006
2534	−5E−007	3.4E−008	2.2169	−1.2159E−007	7.756E−007	2.24E−006

2535	−4E−007	2.0E−008	2.2169	−8.3717E−008	5.326E−007	1.54E−006
2536	−2E−007	1.6E−008	2.2169	−5.7458E−008	3.670E−007	1.06E−006
2537	−2E−007	9.6E−009	2.2169	−3.9573E−008	2.521E−007	7.30E−007
2538	−1E−007	7.3E−009	2.2169	−2.7162E−008	1.737E−007	5.02E−007
2539	−8E−008	4.6E−009	2.2169	−1.8709E−008	1.193E−007	3.45E−007
2540	−5E−008	3.4E−009	2.2169	−1.2843E−008	8.217E−008	2.37E−007
2541	−4E−008	2.2E−009	2.2169	−8.8456E−009	5.646E−008	1.63E−007
2542	−3E−008	1.6E−009	2.2169	−6.0733E−009	3.888E−008	1.12E−007
2543	−2E−008	1.1E−009	2.2169	−4.1826E−009	2.672E−008	7.72E−008
2544	−1E−008	7.6E−010	2.2169	−2.8722E−009	1.839E−008	5.31E−008
2545	−8E−009	5.1E−010	2.2169	−1.9777E−009	1.264E−008	3.65E−008
2546	−6E−009	3.6E−010	2.2169	−1.3584E−009	8.702E−009	2.51E−008
2547	−4E−009	2.4E−010	2.2169	−9.3527E−010	5.982E−009	1.73E−008
2548	−3E−009	1.7E−010	2.2169	−6.4246E−010	4.117E−009	1.19E−008
2549	−2E−009	1.1E−010	2.2169	−4.4218E−010	2.830E−009	8.17E−009
2550	−1E−009	8.1E−011	2.2169	−3.0391E−010	1.948E−009	5.62E−009
2551	−9E−010	5.4E−011	2.2169	−2.091E−010	1.339E−009	3.86E−009
2552	−6E−010	3.8E−011	2.2169	−1.437E−010	9.216E−010	2.66E−009
2553	−4E−010	2.6E−011	2.2169	−9.8998E−011	6.334E−010	1.83E−009
2554	−3E−010	1.8E−011	2.2169	−6.7991E−011	4.358E−010	1.26E−009
2555	−2E−010	1.2E−011	2.2169	−4.6813E−011	2.997E−010	8.64E−010
2556	−1E−010	8.5E−012	2.2169	−3.2109E−011	2.063E−010	5.94E−010
2557	−9E−011	5.7E−012	2.2169	−2.2124E−011	1.418E−010	4.09E−010
2558	−6E−011	3.7E−012	2.2169	−1.5254E−011	9.763E−011	2.81E−010
2559	−4E−011	2.8E−012	2.2169	−1.0514E−011	6.705E−011	1.93E−010
2560	−3E−011	1.8E−012	2.2169	−7.1591E−012	4.608E−011	1.33E−010
2561	−2E−011	1.4E−012	2.2169	−4.9718E−012	3.172E−011	9.14E−011
2562	−1E−011	1E−012	2.2169	−3.3842E−012	2.182E−011	6.29E−011
2563	−1E−011	6.3E−013	2.2169	−2.2858E−012	1.502E−011	4.31E−011
2564	−7E−012	5.3E−013	2.2169	−1.5647E−012	1.042E−011	2.99E−011
2565	−5E−012	2.3E−013	2.2169	−1.0965E−012	7.119E−012	2.04E−011
2566	−3E−012	−2E−013	2.2169	−7.0491E−013	4.943E−012	1.41E−011
2567	−2E−012	2.4E−013	2.2169	−5.389E−013	3.365E−012	9.77E−012
2568	−2E−012	1.3E−013	2.2169	−3.4759E−013	2.450E−012	6.56E−012
2569	−1E−012	2.4E−013	2.2169	−2.77E−013	1.592E−012	4.43E−012
2570	−3E−013	2.8E−013	2.2169	−2.1337E−013	1.090E−012	3.05E−012
2571	−6E−013	−1E−014	2.2169	−9.3005E−014	8.547E−013	1.88E−012
2572	−3E−013	1.6E−013	2.2169	−1.1609E−013	7.404E−013	1.32E−012
2573	−4E−013	−2E−013	2.2169	−6.0849E−014	2.709E−013	7.35E−013
2574	−4E−013	9.4E−015	2.2169	−1.7987E−013	8.329E−015	2.72E−013
2575	−5E−013	−3E−013	2.2169	−1.7378E−013	−1.0E−013	6.94E−016
2576	−3E−013	−1E−013	2.2169	−8.9293E−014	−4.1E−014	−3.4E−013
2577	−8E−014	3.0E−013	2.2169	−2.4038E−014	−1.8E−013	−7.9E−013
2578	−5E−013	−3E−013	2.2169	−1.0836E−013	−8.1E−013	−1.5E−012
2579	−3E−013	2.2E−013	2.2169	−2.238E−014	−5.3E−013	−1.8E−012
2580	−6E−013	4.4E−014	2.2169	−1.883E−013	−9.6E−013	−2.9E−012
2581	−1E−012	−6E−014	2.2169	−2.5145E−013	−1.5E−012	−4.4E−012

2582	−2E−012	−7E−014	2.2169	−3.2265E−013	−1.9E−012	−6.4E−012
2583	−2E−012	6.5E−014	2.2169	−5.7219E−013	−3.6E−012	−1.0E−011
2584	−3E−012	4.0E−013	2.2169	−7.6974E−013	−4.7E−012	−1.4E−011
2585	−5E−012	6.2E−013	2.2169	−1.1283E−012	−7.3E−012	−2.1E−011
2586	−7E−012	6.6E−013	2.2169	−1.7076E−012	−1.0E−011	−3.0E−011
2587	−1E−011	2.8E−013	2.2169	−2.2787E−012	−1.5E−011	−4.3E−011
2588	−1E−011	9.7E−013	2.2169	−3.3412E−012	−2.2E−011	−6.3E−011
2589	−2E−011	1.5E−012	2.2169	−4.8148E−012	−3.1E−011	−9.1E−011
2590	−3E−011	2.1E−012	2.2169	−7.3258E−012	−4.6E−011	−1.3E−010
2591	−4E−011	2.6E−012	2.2169	−1.0438E−011	−6.7E−011	−1.9E−010
2592	−6E−011	3.8E−012	2.2169	−1.5185E−011	−9.7E−011	−2.8E−010
2593	−9E−011	6E−012	2.2169	−2.2199E−011	−1.4E−010	−4.1E−010
2594	−1E−010	8.4E−012	2.2169	−3.2219E−011	−2.1E−010	−5.9E−010
2595	−2E−010	1.2E−011	2.2169	−4.672E−011	−3.0E−010	−8.6E−010
2596	−3E−010	1.8E−011	2.2169	−6.794E−011	−4.4E−010	−1.3E−009
2597	−4E−010	2.6E−011	2.2169	−9.8844E−011	−6.3E−010	−1.8E−009
2598	−6E−010	3.8E−011	2.2169	−1.4373E−010	−9.2E−010	−2.7E−009
2599	−9E−010	5.4E−011	2.2169	−2.0924E−010	−1.3E−009	−3.9E−009
2600	−1E−009	8.0E−011	2.2169	−3.0387E−010	−1.9E−009	−5.6E−009
2601	−2E−009	1.1E−010	2.2169	−4.4228E−010	−2.8E−009	−8.2E−009
2602	−3E−009	1.7E−010	2.2169	−6.4246E−010	−4.1E−009	−1.2E−008
2603	−4E−009	2.4E−010	2.2169	−9.3529E−010	−6.0E−009	−1.7E−008
2604	−6E−009	3.6E−010	2.2169	−1.3584E−009	−8.7E−009	−2.5E−008
2605	−8E−009	5.1E−010	2.2169	−1.9777E−009	−1.3E−008	−3.7E−008
2606	−1E−008	7.6E−010	2.2169	−2.8722E−009	−1.8E−008	−5.3E−008
2607	−2E−008	1.1E−009	2.2169	−4.1825E−009	−2.7E−008	−7.7E−008
2608	−3E−008	1.6E−009	2.2169	−6.0733E−009	−3.9E−008	−1.1E−007
2609	−4E−008	2.2E−009	2.2169	−8.8458E−009	−5.6E−008	−1.6E−007
2610	−5E−008	3.4E−009	2.2169	−1.2843E−008	−8.2E−008	−2.4E−007
2611	−8E−008	4.6E−009	2.2169	−1.8708E−008	−1.2E−007	−3.5E−007
2612	−1E−007	7.3E−009	2.2169	−2.7162E−008	−1.7E−007	−5.0E−007
2613	−2E−007	9.6E−009	2.2169	−3.9573E−008	−2.5E−007	−7.3E−007
2614	−2E−007	1.6E−008	2.2169	−5.7458E−008	−3.7E−007	−1.1E−006
2615	−4E−007	2.0E−008	2.2169	−8.3717E−008	−5.3E−007	−1.5E−006
2616	−5E−007	3.4E−008	2.2169	−1.2159E−007	−7.8E−007	−2.2E−006
2617	−7E−007	4E−008	2.2169	−1.7714E−007	−1.1E−006	−3.3E−006
2618	−1E−006	7.4E−008	2.2169	−2.5746E−007	−1.6E−006	−4.7E−006
2619	−2E−006	7.8E−008	2.2169	−3.7496E−007	−2.4E−006	−6.9E−006
2620	−2E−006	1.6E−007	2.2169	−5.4571E−007	−3.5E−006	−1E−005
2621	−3E−006	1.5E−007	2.2169	−7.9419E−007	−5.0E−006	−1.5E−005
2622	−5E−006	3.8E−007	2.2169	−1.1587E−006	−7.3E−006	−2.1E−005
2623	−7E−006	2.4E−007	2.2168	−1.6842E−006	−1.1E−005	−3.1E−005
2624	−1E−005	9.0E−007	2.2168	−2.4675E−006	−1.5E−005	−4.5E−005
2625	−2E−005	2.8E−007	2.2167	−3.5795E−006	−2.2E−005	−6.5E−005
2626	−2E−005	2.3E−006	2.2167	−5.2819E−006	−3.3E−005	−9.5E−005
2627	−3E−005	−2E−007	2.2166	−7.6398E−006	−4.7E−005	−0.000138
2628	−4E−005	6.1E−006	2.2164	−1.1404E−005	−6.9E−005	−0.000201

2629	−7E−005	−3E−006	2.2162	−1.643E−005	−9.9E−005	−0.000294
2630	−9E−005	1.7E−005	2.2158	−2.4969E−005	−0.0001458	−0.000427
2631	−0.00016	−2E−005	2.2154	−3.5786E−005	−0.0002076	−0.000623
2632	−0.00017	5.3E−005	2.2147	−5.582E−005	−0.0003057	−0.000904
2633	−0.00038	−7E−005	2.2136	−7.9421E−005	−0.0004259	−0.001320
2634	−0.00030	0.000164	2.2123	−0.0001281	−0.0006232	−0.001906
2635	−0.00090	−0.00031	2.21	−0.00017987	−0.0008189	−0.002763
2636	−0.00037	0.000511	2.2074	−0.00030054	−0.0011464	−0.003920
2637	−0.00218	−0.00128	2.2022	−0.00040918	−0.001229	−0.005514
2638	0.0005965	0.001551	2.197	−0.00070191	−0.0012801	−0.007319
2639	−0.00527	−0.00576	2.184	−0.00088875	0.00039957	−0.009148
2640	0.0087819	0.004722	2.1721	−0.0015101	0.0043947	−0.008668
2641	−0.01477	−0.03114	2.135	−0.0029355	0.01726	−0.002262
2642	0.060004	0.015744	2.0938	−0.010971	0.049702	0.032701
2643	0.21345	0.081784	2.2513	0.011031	−0.13622	0.29124
2644	0.030674	−0.01169	2.3885	0.0163	0.079372	−0.36935
2645	0.094931	0.050735	2.0936	−0.00918	−0.019493	−0.007808
2646	−0.02320	−0.03345	2.0938	−0.0017863	−0.0098895	0.0031496
2647	0.01118	0.005256	2.1276	−0.00074121	−0.0016888	0.0053743
2648	−0.00450	−0.00571	2.1536	−0.00040646	−0.0002152	0.0061701
2649	0.0011773	0.000688	2.1732	−0.00023895	0.00087678	0.0046044
2650	−0.00146	−0.00135	2.1872	−0.000133	0.00070216	0.003553
2651	−2E−005	0.000109	2.1967	−8.2683E−005	0.00067373	0.0023974
2652	−0.00055	−0.00038	2.2032	−4.625E−005	0.00046811	0.0017006
2653	−0.00012	3.2E−006	2.2076	−3.0979E−005	0.00035394	0.0011385
2654	−0.00022	−0.00012	2.2106	−1.7394E−005	0.00024254	0.00078686
2655	−8E−005	−1E−005	2.2126	−1.2454E−005	0.00017094	0.00053129
2656	−9E−005	−4E−005	2.214	−7.1095E−006	0.00011766	0.00036436
2657	−4E−005	−1E−005	2.2149	−5.3103E−006	8.089E−005	0.00024821
2658	−4E−005	−2E−005	2.2155	−3.1017E−006	5.599E−005	0.00016991
2659	−2E−005	−6E−006	2.216	−2.3583E−006	3.817E−005	0.00011645
2660	−2E−005	−6E−006	2.2163	−1.41E−006	2.651E−005	7.97E−005
2661	−1E−005	−3E−006	2.2165	−1.0737E−006	1.803E−005	5.48E−005
2662	−8E−006	−3E−006	2.2166	−6.5557E−007	1.254E−005	3.75E−005
2663	−5E−006	−1E−006	2.2167	−4.9583E−007	8.531E−006	2.59E−005
2664	−4E−006	−1E−006	2.2168	−3.0829E−007	5.932E−006	1.77E−005
2665	−2E−006	−7E−007	2.2168	−2.3084E−007	4.039E−006	1.22E−005
2666	−2E−006	−5E−007	2.2168	−1.4576E−007	2.807E−006	8.37E−006
2667	−1E−006	−3E−007	2.2169	−1.0799E−007	1.913E−006	5.77E−006
2668	−8E−007	−3E−007	2.2169	−6.9082E−008	1.328E−006	3.95E−006
2669	−5E−007	−2E−007	2.2169	−5.0664E−008	9.063E−007	2.73E−006
2670	−4E−007	−1E−007	2.2169	−3.277E−008	6.282E−007	1.87E−006
2671	−3E−007	−8E−008	2.2169	−2.3815E−008	4.292E−007	1.29E−006
2672	−2E−007	−5E−008	2.2169	−1.5548E−008	2.972E−007	8.84E−007
2673	−1E−007	−4E−008	2.2169	−1.121E−008	2.033E−007	6.09E−007
2674	−8E−008	−3E−008	2.2169	−7.3762E−009	1.406E−007	4.18E−007
2675	−6E−008	−2E−008	2.2169	−5.2813E−009	9.625E−008	2.88E−007

2676	−4E−008	−1E−008	2.2169	−3.4985E−009	6.653E−008	1.98E−007
2677	−3E−008	−8E−009	2.2169	−2.49E−009	4.557E−008	1.36E−007
2678	−2E−008	−6E−009	2.2169	−1.6588E−009	3.148E−008	9.35E−008
2679	−1E−008	−4E−009	2.2169	−1.1747E−009	2.157E−008	6.44E−008
2680	−9E−009	−3E−009	2.2169	−7.8631E−010	1.489E−008	4.42E−008
2681	−6E−009	−2E−009	2.2169	−5.544E−010	1.021E−008	3.04E−008
2682	−4E−009	−1E−009	2.2169	−3.7257E−010	7.045E−009	2.09E−008
2683	−3E−009	−9E−010	2.2169	−2.6176E−010	4.833E−009	1.44E−008
2684	−2E−009	−6E−010	2.2169	−1.7658E−010	3.333E−009	9.89E−009
2685	−1E−009	−4E−010	2.2169	−1.2362E−010	2.287E−009	6.81E−009
2686	−9E−010	−3E−010	2.2169	−8.3558E−011	1.577E−009	4.68E−009
2687	−6E−010	−2E−010	2.2169	−5.8395E−011	1.082E−009	3.22E−009
2688	−4E−010	−1E−010	2.2169	−3.9587E−011	7.459E−010	2.21E−009
2689	−3E−010	−9E−011	2.2169	−2.7613E−011	5.122E−010	1.52E−009
2690	−2E−010	−6E−011	2.2169	−1.8767E−011	3.528E−010	1.05E−009
2691	−1E−010	−4E−011	2.2169	−1.307E−011	2.424E−010	7.20E−010
2692	−1E−010	−3E−011	2.2169	−8.8915E−012	1.668E−010	4.95E−010
2693	−7E−011	−2E−011	2.2169	−6.1575E−012	1.148E−010	3.40E−010
2694	−5E−011	−1E−011	2.2169	−4.1791E−012	7.894E−011	2.34E−010
2695	−3E−011	−1E−011	2.2169	−2.9657E−012	5.435E−011	1.61E−010
2696	−2E−011	−7E−012	2.2169	−2.0209E−012	3.740E−011	1.11E−010
2697	−2E−011	−5E−012	2.2169	−1.3376E−012	2.586E−011	7.63E−011
2698	−1E−011	−3E−012	2.2169	−9.2987E−013	1.773E−011	5.25E−011
2699	−7E−012	−2E−012	2.2169	−6.3706E−013	1.198E−011	3.59E−011
2700	−5E−012	−2E−012	2.2169	−4.6902E−013	8.337E−012	2.47E−011
2701	−3E−012	−1E−012	2.2169	−3.2087E−013	5.900E−012	1.70E−011
2702	−3E−012	−8E−013	2.2169	−1.8486E−013	3.928E−012	1.19E−011
2703	−2E−012	−5E−013	2.2169	−1.0397E−013	2.907E−012	8.12E−012
2704	−1E−012	−6E−013	2.2169	−1.3865E−013	1.918E−012	5.52E−012
2705	−9E−013	−3E−013	2.2169	1.0961E−015	1.322E−012	4.02E−012
2706	−5E−013	−2E−013	2.2169	−8.4737E−014	8.809E−013	2.69E−012
2707	−5E−013	−1E−013	2.2169	−7.4194E−014	5.546E−013	1.68E−012
2708	−5E−013	−4E−013	2.2169	−4.3329E−014	5.162E−013	1.13E−012
2709	−2E−013	−4E−015	2.2169	−2.0225E−014	2.435E−013	7.25E−013
2710	9.2E−014	9.6E−014	2.2169	−3.2571E−014	2.503E−013	5.40E−013
2711	−7E−014	1.3E−013	2.2169	−9.1457E−014	−1.3E−013	3.81E−013
2712	−3E−014	4.4E−014	2.2169	−4.3597E−015	8.137E−014	−2.3E−014
2713	7.8E−014	2.6E−014	2.2169	−1.0273E−013	−1.1E−013	−2.3E−013
2714	−7E−014	1.5E−013	2.2169	−5.8645E−015	−3.5E−013	−3.3E−013
2715	−6E−013	−4E−013	2.2169	−5.4119E−014	−4.0E−013	−6.8E−013
2716	−2E−013	−5E−014	2.2169	8.9927E−015	−4.7E−013	−8.2E−013
2717	−6E−013	−3E−013	2.2169	−7.3898E−014	−5.0E−013	−1.6E−012
2718	−3E−013	−5E−014	2.2169	−3.5145E−014	−7.0E−013	−3.0E−012
2719	−8E−013	−2E−013	2.2169	−1.1086E−013	−7.2E−013	−3.3E−012
2720	−1E−012	−3E−013	2.2169	−2.2067E−013	−2.1E−012	−5.7E−012
2721	−2E−012	−5E−013	2.2169	−1.3709E−013	−2.9E−012	−7.7E−012
2722	−3E−012	−1E−012	2.2169	−1.7818E−013	−3.9E−012	−1.2E−011

2723	−3E−012	−1E−012	2.2169	−3.0198E−013	−5.8E−012	−1.7E−011
2724	−5E−012	−2E−012	2.2169	−3.24E−013	−8.3E−012	−2.5E−011
2725	−7E−012	−2E−012	2.2169	−6.3662E−013	−1.2E−011	−3.6E−011
2726	−1E−011	−3E−012	2.2169	−9.483E−013	−1.7E−011	−5.2E−011
2727	−2E−011	−4E−012	2.2169	−1.4713E−012	−2.6E−011	−7.6E−011
2728	−2E−011	−7E−012	2.2169	−1.9627E−012	−3.7E−011	−1.1E−010
2729	−3E−011	−1E−011	2.2169	−2.9366E−012	−5.5E−011	−1.6E−010
2730	−5E−011	−1E−011	2.2169	−4.201E−012	−7.9E−011	−2.3E−010
2731	−7E−011	−2E−011	2.2169	−6.2354E−012	−1.2E−010	−3.4E−010
2732	−1E−010	−3E−011	2.2169	−8.8755E−012	−1.7E−010	−4.9E−010
2733	−1E−010	−4E−011	2.2169	−1.3042E−011	−2.4E−010	−7.2E−010
2734	−2E−010	−6E−011	2.2169	−1.867E−011	−3.5E−010	−1.0E−009
2735	−3E−010	−9E−011	2.2169	−2.7589E−011	−5.1E−010	−1.5E−009
2736	−4E−010	−1E−010	2.2169	−3.9535E−011	−7.5E−010	−2.2E−009
2737	−6E−010	−2E−010	2.2169	−5.8404E−011	−1.1E−009	−3.2E−009
2738	−9E−010	−3E−010	2.2169	−8.3601E−011	−1.6E−009	−4.7E−009
2739	−1E−009	−4E−010	2.2169	−1.2363E−010	−2.3E−009	−6.8E−009
2740	−2E−009	−6E−010	2.2169	−1.765E−010	−3.3E−009	−9.9E−009
2741	−3E−009	−9E−010	2.2169	−2.6173E−010	−4.8E−009	−1.4E−008
2742	−4E−009	−1E−009	2.2169	−3.7259E−010	−7.0E−009	−2.1E−008
2743	−6E−009	−2E−009	2.2169	−5.5432E−010	−1.0E−008	−3.0E−008
2744	−9E−009	−3E−009	2.2169	−7.8628E−010	−1.5E−008	−4.4E−008
2745	−1E−008	−4E−009	2.2169	−1.1747E−009	−2.2E−008	−6.4E−008
2746	−2E−008	−6E−009	2.2169	−1.6588E−009	−3.1E−008	−9.3E−008
2747	−3E−008	−8E−009	2.2169	−2.4901E−009	−4.6E−008	−1.4E−007
2748	−4E−008	−1E−008	2.2169	−3.4984E−009	−6.7E−008	−2.0E−007
2749	−6E−008	−2E−008	2.2169	−5.2813E−009	−9.6E−008	−2.9E−007
2750	−8E−008	−3E−008	2.2169	−7.3764E−009	−1.4E−007	−4.2E−007
2751	−1E−007	−4E−008	2.2169	−1.121E−008	−2.0E−007	−6.1E−007
2752	−2E−007	−5E−008	2.2169	−1.5548E−008	−3.0E−007	−8.8E−007
2753	−3E−007	−8E−008	2.2169	−2.3815E−008	−4.3E−007	−1.3E−006
2754	−4E−007	−1E−007	2.2169	−3.277E−008	−6.3E−007	−1.9E−006
2755	−5E−007	−2E−007	2.2169	−5.0664E−008	−9.1E−007	−2.7E−006
2756	−8E−007	−3E−007	2.2169	−6.9083E−008	−1.3E−006	−4.0E−006
2757	−1E−006	−3E−007	2.2169	−1.0799E−007	−1.9E−006	−5.8E−006
2758	−2E−006	−5E−007	2.2168	−1.4576E−007	−2.8E−006	−8.4E−006
2759	−2E−006	−7E−007	2.2168	−2.3084E−007	−4.0E−006	−1.2E−005
2760	−4E−006	−1E−006	2.2168	−3.0829E−007	−5.9E−006	−1.8E−005
2761	−5E−006	−1E−006	2.2167	−4.9583E−007	−8.5E−006	−2.6E−005
2762	−8E−006	−3E−006	2.2166	−6.5557E−007	−1.3E−005	−3.8E−005
2763	−1E−005	−3E−006	2.2165	−1.0737E−006	−1.8E−005	−5.5E−005
2764	−2E−005	−6E−006	2.2163	−1.41E−006	−2.7E−005	−8.0E−005
2765	−2E−005	−6E−006	2.216	−2.3583E−006	−3.8E−005	−0.000116
2766	−4E−005	−2E−005	2.2155	−3.1017E−006	−5.6E−005	−0.000170
2767	−4E−005	−1E−005	2.2149	−5.3103E−006	−8.1E−005	−0.000248
2768	−9E−005	−4E−005	2.214	−7.1095E−006	−0.0001177	−0.000364
2769	−8E−005	−1E−005	2.2126	−1.2454E−005	−0.0001709	−0.000531

2770	−0.00022	−0.00012	2.2106	−1.7394E−005	−0.0002425	−0.000787
2771	−0.00012	3.2E−006	2.2076	−3.0979E−005	−0.0003539	−0.001139
2772	−0.00055	−0.00038	2.2032	−4.625E−005	−0.0004681	−0.001701
2773	−2E−005	0.000109	2.1967	−8.2683E−005	−0.0006737	−0.002397
2774	−0.00146	−0.00135	2.1872	−0.000133	−0.0007022	−0.003553
2775	0.0011773	0.000688	2.1732	−0.00023895	−0.0008768	−0.004604
2776	−0.00450	−0.00571	2.1536	−0.00040646	0.00021518	−0.006170
2777	0.01118	0.005256	2.1276	−0.00074121	0.0016888	−0.005374
2778	−0.02320	−0.03345	2.0938	−0.0017863	0.0098895	−0.003150
2779	0.094931	0.050735	2.0936	−0.00918	0.019493	0.007808
2780	0.030674	−0.01169	2.3885	0.0163	−0.079372	0.36935
2781	1.9775	1.9035	2.1844	−3.3904E−010	1.996E−009	−0.55117
2782	1.8387	1.8003	2.0424	−0.01443	0.19593	−0.34813
2783	1.7815	1.7717	1.9958	−0.012005	0.34072	−0.17557
2784	1.8204	1.8326	2.0678	−1.2474E−011	0.48486	3.74E−010
2785	1.8387	1.8003	2.0424	0.01443	−0.19593	−0.34813
2786	1.8768	1.846	2.0556	3.7623E−011	−1.2E−009	−0.14656
2787	1.8578	1.8373	2.039	−1.674E−011	0.14849	−1.1E−010
2788	1.7815	1.7717	1.9958	0.012005	0.34072	0.17557

stress for circular section for stretch=1.08

NODE	SX	SY	SZ	SXY	SYZ	SXZ
1	2.732	2.7235	2.9466	−8.88E−012	−7.431E−011	−0.67014
2	2.7235	2.732	2.9466	8.862E−012	−0.67014	7.431E−011
3	2.2543	2.2088	2.5136	0.0050237	−0.14985	−0.64668
4	1.9985	1.9808	2.2709	0.01887	−0.33185	−0.49479
5	1.9808	1.9985	2.2709	0.01887	−0.49479	−0.33185
6	2.2088	2.2543	2.5136	0.0050237	−0.64668	−0.14985
7	2.7235	2.732	2.9466	8.887E−012	0.67014	−7.5E−011
8	−0.05959	−0.19284	2.4502	3.824E−011	−0.28885	1.118E−014
9	0.073965	0.14614	1.7645	−1.09E−011	0.053813	−1.5E−011
10	−0.094119	−0.07982	2.181	−6.67E−012	0.0034843	8.563E−012
11	0.018333	0.030904	2.1937	−1.42E−012	0.003213	2.806E−012
12	−0.017994	−0.01294	2.1929	−1.63E−012	0.00084663	7.762E−013
13	0.002631	0.0050713	2.2097	−2.94E−013	0.00060514	5.184E−013
14	−0.004275	−0.00259	2.2089	−4.97E−013	0.00036013	1.823E−013
15	0.00031609	0.0010282	2.214	−8.90E−014	0.00014616	1.571E−013
16	−0.001157	−0.00059	2.214	−1.53E−013	0.00014228	5.380E−014
17	−1.4E−005	0.0002221	2.2155	−3.12E−014	4.095E−005	2.215E−014
18	−0.000335	−0.00014	2.2157	−5.61E−014	5.3435E−005	6.658E−014
19	−3.4E−005	4.7E−005	2.2162	−1.38E−014	1.2427E−005	−2.7E−014
20	−0.000101	−3E−005	2.2162	−2.32E−014	1.9491E−005	−5.9E−014
21	−1.9E−005	9.2E−006	2.2164	4.35E−015	3.9189E−006	−4.7E−014
22	−3.1E−005	−8E−006	2.2164	−7.17E−015	6.9949E−006	−1.6E−014
23	−8.4E−006	1.4E−006	2.2165	8.933E−016	1.256E−006	9.209E−014
24	−9.9E−006	−2E−006	2.2165	−1.19E−014	2.4903E−006	2.917E−014
25	−3.4E−006	2.7E−008	2.2165	1.470E−014	4.0324E−007	8.211E−014
26	−3.2E−006	−5E−007	2.2165	−1.40E−014	8.8405E−007	−3.0E−014
27	−1.3E−006	−1E−007	2.2165	7.395E−015	1.2822E−007	−5.9E−014
28	−1.1E−006	−1E−007	2.2165	1.675E−014	3.1397E−007	−8.1E−015
29	−4.8E−007	−7E−008	2.2165	−5.70E−015	3.9915E−008	−8.6E−014
30	−3.5E−007	−2E−008	2.2165	2.466E−014	1.1178E−007	8.698E−016
31	−1.7E−007	−3E−008	2.2165	−1.36E−014	1.1988E−008	−4.8E−014
32	−1.2E−007	−1E−009	2.2165	−9.17E−015	3.9948E−008	−1.2E−013
33	−6.2E−008	−1E−008	2.2165	1.461E−015	3.3924E−009	−1.8E−013
34	−4E−008	9.4E−010	2.2165	1.487E−015	1.4342E−008	7.655E−015
35	−2.2E−008	−6E−009	2.2165	2.535E−014	8.6101E−010	7.267E−015
36	−1.4E−008	7.8E−010	2.2165	7.896E−015	5.1749E−009	1.301E−013
37	−7.7E−009	−2E−009	2.2165	−2.12E−015	1.6907E−010	−9.5E−014
38	−4.7E−009	4.2E−010	2.2165	−6.63E−015	1.8768E−009	−9.8E−014
39	−2.7E−009	−9E−010	2.2165	−1.87E−014	6.3176E−012	−4.8E−014
40	−1.6E−009	2.0E−010	2.2165	1.456E−014	6.8411E−010	−9.7E−014
41	−9.5E−010	−3E−010	2.2165	1.613E−014	−1.879E−011	1.257E−013
42	−5.5E−010	8.7E−011	2.2165	−2.17E−014	2.5058E−010	1.142E−013
43	−3.3E−010	−1E−010	2.2165	−4.67E−015	−1.500E−011	−2.8E−014
44	−1.9E−010	3.7E−011	2.2165	2.256E−014	9.2501E−011	−5.1E−014

45	−1.2E−010	−5E−011	2.2165	−8.58E−015	−8.417E−012	1.324E−013
46	−6.6E−011	1.6E−011	2.2165	−6.42E−016	3.4527E−011	9.506E−014
47	−4.0E−011	−2E−011	2.2165	1.018E−014	−4.382E−012	8.527E−014
48	−2.3E−011	6.2E−012	2.2165	−6.04E−014	1.2498E−011	−8.1E−014
49	−1.4E−011	−7E−012	2.2165	1.703E−014	−2.002E−012	−2.5E−013
50	−7.9E−012	2.5E−012	2.2165	−2.34E−015	4.7806E−012	4.651E−014
51	−5.3E−012	−3E−012	2.2165	6.315E−015	−1.124E−012	3.063E−014
52	−2.6E−012	1.2E−012	2.2165	−1.93E−014	2.1423E−012	2.843E−014
53	−2.0E−012	−1E−012	2.2165	4.314E−015	−1.795E−013	2.172E−014
54	−8.2E−013	3.1E−013	2.2165	4.832E−014	5.9769E−013	−4.2E−014
55	−6E−013	−2E−013	2.2165	−2.83E−014	−8.355E−013	−6.3E−014
56	−4.0E−013	3.3E−013	2.2165	4.222E−014	6.6318E−013	1.647E−013
57	−4.6E−013	−4E−013	2.2165	1.27E−014	1.7585E−013	−1.4E−014
58	−4.8E−013	−5E−013	2.2165	−1.88E−014	2.494E−013	−5.2E−014
59	−1.7E−014	−1E−013	2.2165	−1.35E−014	−2.744E−013	−3.3E−013
60	9.15E−014	9.9E−014	2.2165	1.312E−014	−1.209E−013	−2.5E−014
61	−6.2E−013	−4E−014	2.2165	1.373E−014	−1.497E−013	1.898E−013
62	−3.2E−013	4.0E−013	2.2165	4.787E−014	3.2349E−015	2.153E−013
63	−8.1E−013	−6E−013	2.2165	−3.36E−014	2.8626E−013	5.246E−014
64	−6.0E−013	3.0E−014	2.2165	−2.94E−014	−6.860E−013	−9.6E−014
65	1.12E−013	2.3E−014	2.2165	3.155E−014	2.474E−013	−4.8E−014
66	1.71E−013	−6E−017	2.2165	5.976E−015	−1.253E−013	5.213E−014
67	5.15E−013	6.1E−013	2.2165	3.623E−014	−1.505E−014	2.448E−013
68	2.49E−013	−6E−013	2.2165	−3.53E−014	9.9421E−013	2.376E−013
69	1.84E−014	−2E−013	2.2165	−2.85E−015	7.8307E−013	3.491E−013
70	1.82E−014	−2E−013	2.2165	−6.76E−014	1.3644E−012	1.977E−015
71	−9.5E−013	−7E−013	2.2165	1.139E−013	−6.452E−013	−8.1E−014
72	5.32E−013	2.9E−013	2.2165	−8.94E−014	7.5217E−013	−1.5E−013
73	6.31E−013	2.5E−013	2.2165	2.741E−014	−6.466E−013	−2.8E−013
74	−8.1E−014	1.6E−013	2.2165	2.897E−014	1.1829E−013	−1.8E−013
75	−7.4E−013	−4E−013	2.2165	6.687E−014	5.4699E−013	1.576E−013
76	−4.5E−013	−4E−013	2.2165	−6.52E−014	−8.502E−013	−1.9E−013
77	1.49E−013	5.3E−013	2.2165	−5.23E−015	−1.938E−013	2.360E−013
78	−3.8E−013	−4E−013	2.2165	−4.65E−014	1.8458E−013	−2.1E−013
79	−6.9E−013	−4E−013	2.2165	7.615E−015	−6.116E−013	−3.8E−014
80	−3.2E−013	−6E−014	2.2165	3.535E−014	1.3201E−012	2.993E−013
81	−3.7E−013	−2E−013	2.2165	−1.18E−013	−8.737E−013	−2.6E−014
82	−3.8E−013	2.6E−013	2.2165	−9.14E−016	1.303E−012	−2.7E−013
83	2.50E−013	−2E−013	2.2165	−4.75E−014	5.68E−014	−4.2E−013
84	−4.5E−013	4.2E−013	2.2165	2.766E−015	5.9432E−015	−8.3E−014
85	1.44E−013	5.4E−013	2.2165	−2.77E−014	−5.358E−013	−5.5E−014
86	4.10E−013	4.5E−013	2.2165	4.982E−014	−3.147E−013	−1.6E−014
87	3.50E−013	3.1E−013	2.2165	−1.37E−014	1.4051E−012	2.747E−013
88	−2.5E−013	−2E−013	2.2165	−8.86E−015	−2.535E−014	−2.2E−013
89	−2.6E−013	−2E−013	2.2165	−9.97E−014	−5.338E−013	−2.4E−013
90	5.58E−013	2.7E−013	2.2165	8.619E−014	−1.610E−013	−3.5E−013
91	−7.3E−013	−9E−013	2.2165	−6.04E−014	−4.166E−013	−8.3E−014

92	−7.0E−013	2.9E−013	2.2165	−1.60E−015	−6.286E−013	−3.3E−013
93	1.13E−014	4.3E−013	2.2165	1.723E−014	4.3817E−013	−1.2E−013
94	−8.4E−013	−4E−013	2.2165	3.765E−014	4.585E−013	−4.4E−013
95	−4.2E−013	9.9E−014	2.2165	−3.71E−014	−6.402E−013	−4.8E−014
96	−4.0E−013	−2E−013	2.2165	3.742E−015	−2.457E−013	1.332E−013
97	−1.9E−013	−3E−013	2.2165	−2.87E−014	−4.678E−013	1.010E−014
98	−9.4E−013	1.9E−013	2.2165	−1.41E−014	−3.953E−013	−4.9E−014
99	−1.1E−012	−8E−013	2.2165	−8.60E−015	1.4722E−013	−2.4E−014
100	−1.3E−012	2.1E−012	2.2165	4.963E−014	−1.639E−012	1.778E−013
101	−4.5E−012	−3E−012	2.2165	−4.34E−014	6.059E−013	1.629E−013
102	−7.5E−012	3.6E−012	2.2165	2.140E−015	−4.768E−012	5.262E−013
103	−1.4E−011	−8E−012	2.2165	−5.83E−014	1.869E−012	3.734E−013
104	−2.3E−011	5.6E−012	2.2165	4.183E−015	−1.300E−011	2.918E−013
105	−4.1E−011	−2E−011	2.2165	3.667E−014	3.7991E−012	−1.1E−013
106	−6.6E−011	1.6E−011	2.2165	3.101E−014	−3.490E−011	1.194E−013
107	−1.2E−010	−5E−011	2.2165	1.857E−014	9.6478E−012	5.026E−013
108	−1.9E−010	3.8E−011	2.2165	1.554E−014	−9.255E−011	2.186E−013
109	−3.3E−010	−1E−010	2.2165	7.753E−015	1.5213E−011	2.537E−013
110	−5.5E−010	8.6E−011	2.2165	−4.42E−014	−2.505E−010	5.869E−014
111	−9.5E−010	−3E−010	2.2165	6.981E−015	1.912E−011	4.795E−014
112	−1.6E−009	2.0E−010	2.2165	−3.05E−015	−6.836E−010	−2.4E−013
113	−2.7E−009	−9E−010	2.2165	8.188E−014	−6.808E−012	3.791E−013
114	−4.7E−009	4.2E−010	2.2165	9.595E−014	−1.878E−009	−6.2E−014
115	−7.7E−009	−2E−009	2.2165	−9.17E−014	−1.694E−010	−2.1E−014
116	−1.4E−008	7.8E−010	2.2165	6.982E−014	−5.175E−009	−3.9E−014
117	−2.2E−008	−6E−009	2.2165	−9.89E−014	−8.591E−010	3.527E−013
118	−4E−008	9.4E−010	2.2165	7.335E−014	−1.434E−008	−1.7E−013
119	−6.2E−008	−1E−008	2.2165	1.265E−014	−3.393E−009	1.904E−013
120	−1.2E−007	−1E−009	2.2165	−9.33E−014	−3.995E−008	−5.9E−014
121	−1.7E−007	−3E−008	2.2165	1.020E−013	−1.199E−008	−3.0E−013
122	−3.5E−007	−2E−008	2.2165	−8.91E−015	−1.118E−007	6.417E−013
123	−4.8E−007	−7E−008	2.2165	1.011E−013	−3.991E−008	−1.5E−013
124	−1.1E−006	−1E−007	2.2165	5.772E−014	−3.140E−007	−1.4E−013
125	−1.3E−006	−1E−007	2.2165	−1.17E−014	−1.282E−007	3.821E−013
126	−3.2E−006	−5E−007	2.2165	9.118E−015	−8.841E−007	6.679E−014
127	−3.4E−006	2.7E−008	2.2165	9.129E−014	−4.032E−007	−1.7E−013
128	−9.9E−006	−2E−006	2.2165	−5.65E−016	−2.490E−006	7.321E−013
129	−8.4E−006	1.4E−006	2.2165	−1.13E−013	−1.256E−006	−6.1E−013
130	−3.1E−005	−8E−006	2.2164	9.916E−014	−6.995E−006	−9.7E−013
131	−1.9E−005	9.2E−006	2.2164	−2.84E−014	−3.919E−006	−7.0E−013
132	−0.000101	−3E−005	2.2162	3.658E−014	−1.949E−005	−9.3E−014
133	−3.4E−005	4.7E−005	2.2162	−5.42E−014	−1.243E−005	3.787E−014
134	−0.000335	−0.00014	2.2157	5.304E−014	−5.343E−005	−1.0E−013
135	−1.4E−005	0.0002221	2.2155	−1.19E−013	−4.095E−005	−3.1E−013
136	−0.001157	−0.00059	2.214	3.465E−014	−0.00014228	−6.9E−013
137	0.00031609	0.0010282	2.214	−1.17E−013	−0.00014616	2.069E−013
138	−0.004275	−0.00259	2.2089	−4.76E−013	−0.00036013	3.742E−014

139	0.002631	0.0050713	2.2097	−3.38E−013	−0.00060514	−7.9E−013
140	−0.017994	−0.01294	2.1929	−1.60E−012	−0.00084663	−1.6E−012
141	0.018333	0.030904	2.1937	−1.34E−012	−0.003213	−3.2E−012
142	−0.094119	−0.07982	2.181	−6.76E−012	−0.0034843	−9.0E−012
143	0.073965	0.14614	1.7645	−1.07E−011	−0.053813	1.514E−011
144	−0.05959	−0.19284	2.4502	3.798E−011	0.28885	−5.4E−014
145	2.732	2.7235	2.9466	−8.92E−012	7.4401E−011	0.67014
146	2.2543	2.2088	2.5136	0.0050237	0.14985	0.64668
147	1.9985	1.9808	2.2709	0.01887	0.33185	0.49479
148	1.9808	1.9985	2.2709	0.01887	0.49479	0.33185
149	2.2088	2.2543	2.5136	0.0050237	0.64668	0.14985
150	−0.19284	−0.05959	2.4502	−3.82E−011	−3.537E−014	−0.28885
151	0.14614	0.073965	1.7645	1.091E−011	1.5009E−011	0.053813
152	−0.079819	−0.09412	2.181	6.660E−012	−8.552E−012	0.0034843
153	0.030904	0.018333	2.1937	1.408E−012	−2.819E−012	0.003213
154	−0.012937	−0.01799	2.1929	1.631E−012	−7.757E−013	0.00084663
155	0.0050713	0.002631	2.2097	3.008E−013	−4.896E−013	0.00060514
156	−0.002588	−0.00428	2.2089	4.824E−013	−1.704E−013	0.00036013
157	0.0010282	0.0003161	2.214	1.050E−013	−1.260E−013	0.00014616
158	−0.000589	−0.00116	2.214	1.44E−013	−7.844E−014	0.00014228
159	0.00022205	−1E−005	2.2155	3.194E−014	−7.213E−014	4.095E−005
160	−0.000142	−0.00033	2.2157	2.752E−014	−1.949E−014	5.343E−005
161	4.72E−005	−3E−005	2.2162	2.432E−014	−5.191E−014	1.243E−005
162	−3.5E−005	−0.00010	2.2162	1.585E−014	−8.512E−015	1.949E−005
163	9.21E−006	−2E−005	2.2164	5.481E−015	4.9624E−014	3.919E−006
164	−8.4E−006	−3E−005	2.2164	5.036E−015	−3.363E−014	6.995E−006
165	1.40E−006	−8E−006	2.2165	2.381E−014	5.8591E−014	1.256E−006
166	−2.0E−006	−1E−005	2.2165	−7.82E−015	3.0053E−014	2.490E−006
167	2.70E−008	−3E−006	2.2165	8.431E−015	−1.065E−013	4.032E−007
168	−4.7E−007	−3E−006	2.2165	3.653E−015	−1.499E−014	8.841E−007
169	−1.1E−007	−1E−006	2.2165	9.416E−015	8.6921E−014	1.282E−007
170	−1E−007	−1E−006	2.2165	−2.49E−015	1.3254E−013	3.140E−007
171	−7.1E−008	−5E−007	2.2165	−3.16E−016	1.7364E−013	3.991E−008
172	−1.8E−008	−4E−007	2.2165	1.774E−015	2.457E−015	1.118E−007
173	−3.3E−008	−2E−007	2.2165	−3.74E−015	3.426E−014	1.199E−008
174	−1.4E−009	−1E−007	2.2165	−4.27E−015	−4.831E−014	3.995E−008
175	−1.4E−008	−6E−008	2.2165	1.077E−014	−1.188E−013	3.392E−009
176	9.41E−010	−4E−008	2.2165	3.718E−015	6.1001E−014	1.434E−008
177	−5.7E−009	−2E−008	2.2165	7.344E−015	−2.95E−014	8.614E−010
178	7.77E−010	−1E−008	2.2165	−4.68E−016	−6.138E−014	5.175E−009
179	−2.2E−009	−8E−009	2.2165	3.547E−015	−2.482E−014	1.692E−010
180	4.19E−010	−5E−009	2.2165	2.569E−014	6.0688E−014	1.877E−009
181	−8.7E−010	−3E−009	2.2165	−2.14E−014	−5.998E−014	6.281E−012
182	1.97E−010	−2E−009	2.2165	2.545E−014	3.9368E−014	6.843E−010
183	−3.3E−010	−1E−009	2.2165	−3.38E−014	−4.701E−014	−1.9E−011
184	8.71E−011	−6E−010	2.2165	2.310E−014	−2.347E−014	2.508E−010
185	−1.3E−010	−3E−010	2.2165	2.500E−015	6.4023E−014	−1.5E−011

186	3.76E−011	−2E−010	2.2165	2.813E−014	4.4948E−014	9.236E−011
187	−4.8E−011	−1E−010	2.2165	3.359E−015	9.8522E−014	−8.6E−012
188	1.58E−011	−7E−011	2.2165	1.298E−014	−9.589E−014	3.344E−011
189	−1.9E−011	−4E−011	2.2165	−6.22E−015	−4.458E−014	−4.0E−012
190	6.33E−012	−2E−011	2.2165	−9.14E−015	−3.268E−013	1.179E−011
191	−6.8E−012	−1E−011	2.2165	3.286E−014	−1.718E−013	−1.9E−012
192	2.04E−012	−8E−012	2.2165	2.762E−014	−8.562E−014	4.699E−012
193	−2.4E−012	−5E−012	2.2165	−1.41E−014	−9.466E−014	−9.8E−013
194	8.15E−013	−3E−012	2.2165	6.189E−015	2.0141E−013	1.598E−012
195	−1.1E−012	−2E−012	2.2165	−1.76E−014	−2.137E−013	−2.8E−013
196	3.03E−013	−1E−012	2.2165	−3.50E−014	−3.840E−013	7.764E−013
197	−2.7E−013	−5E−013	2.2165	4.183E−014	−4.428E−013	−2.1E−013
198	3.92E−013	−3E−013	2.2165	2.263E−014	8.732E−014	−2.3E−015
199	−9.9E−013	−9E−013	2.2165	−1.40E−014	3.8183E−013	5.445E−013
200	5.40E−013	3.3E−013	2.2165	7.331E−014	1.6592E−013	−1.5E−013
201	−7.9E−014	1.7E−013	2.2165	−5.44E−014	−1.114E−013	2.407E−013
202	1.69E−013	2.8E−013	2.2165	1.21E−014	−5.240E−014	−3.7E−013
203	2.49E−014	3.0E−013	2.2165	3.208E−014	−2.377E−014	−2.5E−013
204	−2.9E−013	−2E−013	2.2165	1.041E−014	−2.658E−013	−4.0E−013
205	2.63E−013	1.2E−013	2.2165	2.410E−014	−3.471E−014	3.159E−013
206	3.95E−013	2.9E−013	2.2165	−2.80E−014	2.2625E−013	−3.0E−013
207	−9.9E−013	−9E−013	2.2165	−3.31E−016	3.32E−013	−2.9E−013
208	1.11E−013	4.0E−014	2.2165	−2.56E−014	2.5817E−013	8.283E−013
209	1.31E−013	−1E−013	2.2165	−7.19E−015	3.0412E−013	6.03E−013
210	3.90E−014	−1E−013	2.2165	−2.40E−014	2.5049E−014	−3.5E−013
211	1.67E−013	−1E−013	2.2165	−1.58E−014	1.0588E−013	−1.5E−013
212	−5.0E−013	−1E−013	2.2165	−5.87E−014	−1.345E−013	−5.4E−013
213	2.34E−013	−4E−013	2.2165	3.671E−014	9.7675E−014	3.090E−013
214	−5.3E−013	−4E−013	2.2165	1.019E−014	−5.309E−014	8.566E−013
215	−3.3E−014	−9E−014	2.2165	−4.11E−014	−1.171E−013	−1.7E−013
216	−3.5E−013	−3E−015	2.2165	6.880E−014	2.4433E−013	1.446E−013
217	4.68E−013	2.6E−013	2.2165	−6.29E−014	1.0556E−013	−2.0E−014
218	1.00E−013	−4E−014	2.2165	−1.42E−014	−2.639E−013	7.786E−013
219	6.66E−014	−3E−013	2.2165	4.977E−014	3.6039E−015	−2.2E−013
220	−1.9E−013	−2E−013	2.2165	−9.05E−015	7.6322E−014	−2.3E−014
221	1.50E−013	−1E−013	2.2165	2.342E−014	3.0507E−013	−7.9E−013
222	−9.1E−014	−3E−013	2.2165	−8.75E−014	1.7397E−013	8.044E−013
223	1.94E−013	2.9E−013	2.2165	2.373E−014	6.4041E−014	−3.6E−014
224	5.15E−013	−7E−014	2.2165	−3.22E−014	−1.217E−014	−6.9E−013
225	−8.0E−013	−4E−013	2.2165	3.166E−014	2.3139E−013	−2.5E−013
226	7.47E−013	−1E−013	2.2165	−8.41E−014	−3.118E−013	2.471E−013
227	4.26E−013	2.8E−013	2.2165	1.241E−013	−1.03E−013	5.938E−013
228	−5.5E−013	−4E−013	2.2165	−2.86E−014	1.0754E−013	7.570E−014
229	1.45E−013	−4E−013	2.2165	6.582E−014	3.0047E−013	1.254E−013
230	1.45E−013	−3E−013	2.2165	−5.98E−014	−6.551E−014	9.619E−013
231	−7.1E−013	−6E−013	2.2165	−2.99E−014	−7.111E−013	−7.2E−013
232	7.57E−013	4.4E−013	2.2165	2.705E−014	3.5224E−014	6.160E−013

233	−5.5E−013	−1E−013	2.2165	2.500E−014	8.046E−014	−2.1E−013
234	−2.6E−013	−3E−014	2.2165	−9.76E−014	5.388E−013	−1.7E−013
235	2.91E−013	4.1E−013	2.2165	9.277E−014	1.8193E−013	−1.9E−013
236	−2.9E−013	−6E−013	2.2165	−1.13E−013	2.0646E−013	−2.0E−013
237	3.88E−014	−4E−013	2.2165	−3.07E−014	−3.344E−013	−4.9E−013
238	−3.6E−013	−5E−013	2.2165	3.871E−014	−4.031E−013	−1.2E−012
239	−2.8E−013	−7E−013	2.2165	−1.58E−014	−2.267E−013	3.415E−014
240	1.54E−013	−1E−012	2.2165	−1.08E−014	−5.878E−013	−1.7E−012
241	−1.1E−012	−2E−012	2.2165	3.020E−015	−1.275E−013	3.151E−013
242	1.18E−012	−3E−012	2.2165	−2.55E−014	−5.421E−013	−2.0E−012
243	−3.7E−012	−5E−012	2.2165	4.576E−014	−4.461E−013	2.555E−012
244	2.82E−012	−8E−012	2.2165	3.898E−015	5.5036E−015	−5.6E−012
245	−6.3E−012	−1E−011	2.2165	−6.00E−014	1.5977E−013	2.492E−012
246	4.75E−012	−2E−011	2.2165	−1.89E−014	−2.400E−013	−1.3E−011
247	−1.8E−011	−4E−011	2.2165	6.488E−014	−6.885E−014	4.194E−012
248	1.68E−011	−7E−011	2.2165	−3.54E−014	3.4912E−013	−3.6E−011
249	−5.0E−011	−1E−010	2.2165	2.697E−014	2.5957E−013	9.211E−012
250	3.73E−011	−2E−010	2.2165	−3.74E−014	−2.332E−013	−9.4E−011
251	−1.3E−010	−3E−010	2.2165	9.356E−014	7.4852E−015	1.559E−011
252	8.62E−011	−6E−010	2.2165	2.666E−015	9.6545E−014	−2.5E−010
253	−3.3E−010	−1E−009	2.2165	−2.00E−014	−2.596E−013	2.017E−011
254	1.96E−010	−2E−009	2.2165	−5.64E−014	−7.331E−014	−6.8E−010
255	−8.7E−010	−3E−009	2.2165	−2.23E−014	−1.302E−013	−7.0E−012
256	4.20E−010	−5E−009	2.2165	3.400E−014	6.71E−014	−1.9E−009
257	−2.2E−009	−8E−009	2.2165	−4.78E−014	1.7853E−013	−1.7E−010
258	7.77E−010	−1E−008	2.2165	3.266E−014	−1.581E−013	−5.2E−009
259	−5.7E−009	−2E−008	2.2165	6.108E−015	−2.471E−013	−8.6E−010
260	9.41E−010	−4E−008	2.2165	−1.03E−013	1.1951E−013	−1.4E−008
261	−1.4E−008	−6E−008	2.2165	−3.93E−014	−2.130E−013	−3.4E−009
262	−1.4E−009	−1E−007	2.2165	1.109E−013	−9.334E−014	−4.0E−008
263	−3.3E−008	−2E−007	2.2165	−5.80E−014	−1.008E−013	−1.2E−008
264	−1.8E−008	−4E−007	2.2165	−1.46E−014	−6.655E−014	−1.1E−007
265	−7.1E−008	−5E−007	2.2165	8.709E−015	6.8552E−014	−4.0E−008
266	−1E−007	−1E−006	2.2165	5.703E−014	9.0189E−014	−3.1E−007
267	−1.1E−007	−1E−006	2.2165	−7.18E−014	−1.777E−013	−1.3E−007
268	−4.7E−007	−3E−006	2.2165	−1.14E−014	9.7653E−014	−8.8E−007
269	2.70E−008	−3E−006	2.2165	−7.20E−014	6.0063E−014	−4.0E−007
270	−2.0E−006	−1E−005	2.2165	2.070E−014	3.3079E−013	−2.5E−006
271	1.40E−006	−8E−006	2.2165	−5.65E−014	2.0217E−013	−1.3E−006
272	−8.4E−006	−3E−005	2.2164	7.499E−014	−3.432E−013	−7.0E−006
273	9.21E−006	−2E−005	2.2164	−3.58E−014	−4.860E−013	−3.9E−006
274	−3.5E−005	−0.00010	2.2162	−5.22E−014	−2.468E−013	−1.9E−005
275	4.72E−005	−3E−005	2.2162	5.038E−014	2.5734E−013	−1.2E−005
276	−0.000142	−0.00033	2.2157	−1.47E−014	1.301E−013	−5.3E−005
277	0.00022205	−1E−005	2.2155	1.762E−014	−1.178E−013	−4.1E−005
278	−0.000589	−0.00116	2.214	1.376E−013	1.7537E−013	−0.0001423
279	0.0010282	0.0003161	2.214	1.532E−013	2.9762E−013	−0.0001462

280	−0.002588	−0.00428	2.2089	4.965E−013	7.6506E−013	−0.0003601
281	0.0050713	0.002631	2.2097	2.590E−013	9.4804E−013	−0.0006051
282	−0.012937	−0.01799	2.1929	1.695E−012	9.2939E−013	−0.0008466
283	0.030904	0.018333	2.1937	1.316E−012	2.6433E−012	−0.003213
284	−0.079819	−0.09412	2.181	6.673E−012	7.3875E−012	−0.0034843
285	0.14614	0.073965	1.7645	1.095E−011	−1.517E−011	−0.053813
286	−0.19284	−0.05959	2.4502	−3.83E−011	3.0808E−013	0.28885
287	−0.026163	0.056051	2.2586	−0.0026513	−0.09443	−0.29655
288	0.080882	0.043625	2.0092	0.013804	0.0076766	0.051928
289	−0.037796	−0.04855	2.1255	0.0015614	0.012957	0.012956
290	0.012779	0.0028793	2.1793	0.0012632	0.0032786	0.0031784
291	−0.006291	−0.00969	2.1967	0.0005068	0.0024157	0.0029059
292	0.0020145	−0.00056	2.2055	0.00032964	0.00076194	0.00086209
293	−0.001295	−0.00247	2.2102	0.00015287	0.00063891	0.00084654
294	0.00039323	−0.00040	2.2128	0.00010459	0.00023494	0.00030191
295	−0.000321	−0.00073	2.2144	5.158E−005	0.00018902	0.00027006
296	8.39E−005	−0.00018	2.2153	3.503E−005	8.0208E−005	0.00011186
297	−8.7E−005	−0.00023	2.2158	1.798E−005	5.9293E−005	8.902E−005
298	1.78E−005	−7E−005	2.2161	1.195E−005	2.82E−005	4.123E−005
299	−2.5E−005	−7E−005	2.2163	6.300E−006	1.9231E−005	2.977E−005
300	3.46E−006	−3E−005	2.2164	4.106E−006	9.9711E−006	1.498E−005
301	−7.3E−006	−2E−005	2.2164	2.206E−006	6.3674E−006	1.004E−005
302	5.28E−007	−1E−005	2.2165	1.416E−006	3.5215E−006	5.380E−006
303	−2.2E−006	−8E−006	2.2165	7.704E−007	2.1367E−006	3.4E−006
304	1.44E−008	−4E−006	2.2165	4.891E−007	1.2405E−006	1.916E−006
305	−6.8E−007	−3E−006	2.2165	2.685E−007	7.2333E−007	1.156E−006
306	−3.7E−008	−1E−006	2.2165	1.693E−007	4.3588E−007	6.786E−007
307	−2.2E−007	−9E−007	2.2165	9.339E−008	2.4629E−007	3.942E−007
308	−2.3E−008	−5E−007	2.2165	5.867E−008	1.5288E−007	2.394E−007
309	−7.0E−008	−3E−007	2.2165	3.243E−008	8.4171E−008	1.346E−007
310	−1.1E−008	−2E−007	2.2165	2.036E−008	5.355E−008	8.431E−008
311	−2.3E−008	−1E−007	2.2165	1.125E−008	2.8831E−008	4.603E−008
312	−4.3E−009	−6E−008	2.2165	7.071E−009	1.8744E−008	2.964E−008
313	−7.7E−009	−4E−008	2.2165	3.899E−009	9.8871E−009	1.575E−008
314	−1.6E−009	−2E−008	2.2165	2.458E−009	6.5594E−009	1.042E−008
315	−2.6E−009	−1E−008	2.2165	1.350E−009	3.3923E−009	5.386E−009
316	−5.8E−010	−7E−009	2.2165	8.554E−010	2.2958E−009	3.659E−009
317	−9.0E−010	−4E−009	2.2165	4.668E−010	1.1636E−009	1.842E−009
318	−2E−010	−3E−009	2.2165	2.979E−010	8.0386E−010	1.286E−009
319	−3.1E−010	−2E−009	2.2165	1.613E−010	3.9901E−010	6.292E−010
320	−6.8E−011	−9E−010	2.2165	1.038E−010	2.816E−010	4.521E−010
321	−1.1E−010	−5E−010	2.2165	5.57E−011	1.3652E−010	2.147E−010
322	−2.2E−011	−3E−010	2.2165	3.622E−011	9.8644E−011	1.591E−010
323	−3.9E−011	−2E−010	2.2165	1.923E−011	4.6773E−011	7.328E−011
324	−7.1E−012	−1E−010	2.2165	1.262E−011	3.469E−011	5.604E−011
325	−1.4E−011	−6E−011	2.2165	6.621E−012	1.6052E−011	2.483E−011
326	−2.4E−012	−4E−011	2.2165	4.383E−012	1.1972E−011	1.976E−011

327	−5.1E−012	−2E−011	2.2165	2.296E−012	5.5679E−012	8.464E−012
328	−6.1E−013	−1E−011	2.2165	1.560E−012	4.3057E−012	6.993E−012
329	−1.9E−012	−8E−012	2.2165	7.914E−013	1.9758E−012	3.024E−012
330	−3.8E−013	−5E−012	2.2165	5.042E−013	1.4987E−012	2.391E−012
331	−6.3E−013	−3E−012	2.2165	3.004E−013	5.5795E−013	1.000E−012
332	5.83E−016	−2E−012	2.2165	2.231E−013	6.3939E−013	7.896E−013
333	−2.4E−013	−9E−013	2.2165	1.307E−013	2.2276E−013	3.296E−013
334	1.70E−013	−4E−013	2.2165	9.035E−014	2.5661E−013	2.794E−014
335	−3.2E−013	−6E−013	2.2165	2.922E−014	3.5099E−014	4.128E−013
336	1.61E−015	−2E−013	2.2165	3.447E−014	2.5383E−014	8.057E−014
337	−9.2E−014	−1E−013	2.2165	−6.49E−014	8.2507E−014	3.943E−014
338	−5.8E−013	−6E−013	2.2165	−4.77E−014	−7.653E−014	1.980E−013
339	−1.0E−013	−3E−013	2.2165	−1.85E−014	−4.719E−014	1.710E−013
340	−1.3E−014	−8E−015	2.2165	−5.96E−014	−8.002E−014	−5.3E−015
341	−6.4E−014	2.4E−014	2.2165	−5.97E−015	−6.079E−014	1.856E−013
342	−1.1E−013	−2E−013	2.2165	4.102E−014	−2.321E−014	−3.4E−013
343	7.02E−014	6.8E−014	2.2165	1.485E−014	4.2541E−014	5.548E−013
344	−4.0E−014	1.4E−013	2.2165	−5.93E−015	4.1283E−014	1.052E−013
345	1.21E−013	1.6E−013	2.2165	−2.17E−015	−7.774E−014	−3.8E−013
346	−1.2E−013	5.9E−014	2.2165	5.560E−014	−5.253E−015	−5.7E−014
347	−3.0E−013	−2E−013	2.2165	1.048E−014	5.8888E−014	−5.0E−013
348	−1.4E−013	−2E−013	2.2165	−5.41E−014	−1.868E−013	1.843E−014
349	1.39E−013	2.8E−013	2.2165	2.644E−014	7.4186E−014	−1.3E−014
350	−1.1E−013	−1E−014	2.2165	1.006E−014	−4.860E−014	−2.4E−013
351	−5.6E−014	−2E−013	2.2165	4.518E−014	3.4446E−014	−1.1E−013
352	2.63E−013	1.6E−013	2.2165	−3.88E−014	−3.757E−014	−8.2E−014
353	3.53E−014	1.7E−013	2.2165	1.821E−014	−1.593E−013	−1.0E−014
354	2.22E−013	2.0E−013	2.2165	−5.37E−014	2.0065E−014	3.269E−014
355	−3.8E−013	−4E−013	2.2165	5.540E−014	−1.520E−013	4.362E−013
356	−5.6E−013	−5E−013	2.2165	3.99E−014	6.631E−015	4.997E−014
357	−3.6E−013	−3E−013	2.2165	7.919E−015	1.1801E−015	−3.0E−013
358	2.11E−013	3.2E−013	2.2165	4.888E−014	2.3453E−013	−1.7E−013
359	9.84E−014	3.6E−013	2.2165	−1.27E−014	1.0085E−014	−2.6E−014
360	6.21E−014	1.4E−013	2.2165	−3.99E−014	−2.938E−014	−1.3E−013
361	−3.7E−014	2.3E−013	2.2165	−1.20E−014	−4.657E−013	1.400E−013
362	−1.3E−013	−2E−013	2.2165	5.564E−014	−1.031E−013	−9.2E−014
363	−2.8E−013	−2E−013	2.2165	4.055E−014	−2.553E−013	−5.8E−014
364	5.10E−014	1.7E−013	2.2165	3.851E−014	−3.897E−014	−3.4E−013
365	−1.5E−013	−2E−013	2.2165	2.533E−015	8.8871E−014	2.369E−013
366	−3.3E−014	−2E−014	2.2165	4.222E−015	1.0795E−013	−3.0E−013
367	2.37E−014	−1E−013	2.2165	2.58E−014	−2.558E−014	−1.4E−013
368	−3.4E−013	−2E−013	2.2165	1.536E−014	5.9268E−014	5.84E−013
369	1.94E−013	1.8E−013	2.2165	−3.77E−014	2.6469E−014	−2.4E−013
370	−1.3E−013	9.8E−014	2.2165	−7.39E−015	−2.584E−013	−4.9E−013
371	8.98E−014	3.3E−013	2.2165	−7.10E−014	−2.293E−013	−1.8E−013
372	−6.3E−014	−5E−016	2.2165	4.967E−014	−2.042E−013	−4.2E−013
373	−1.1E−013	−2E−013	2.2165	6.638E−014	−6.462E−014	2.350E−013

374	3.93E−013	2.0E−013	2.2165	−1.91E−014	−2.479E−013	4.163E−013
375	−1.4E−013	−3E−013	2.2165	9.641E−014	−2.174E−013	1.211E−013
376	−1.5E−013	−7E−013	2.2165	7.510E−014	−1.778E−013	−7.8E−013
377	2.77E−013	−4E−013	2.2165	9.200E−014	−2.106E−013	2.923E−013
378	6.47E−014	−1E−012	2.2165	1.741E−013	−4.173E−013	−1.2E−012
379	−1.1E−014	−2E−012	2.2165	3.053E−013	−6.137E−013	−8.5E−013
380	−1.4E−013	−4E−012	2.2165	5.827E−013	−1.516E−012	−3.4E−012
381	−1.6E−012	−7E−012	2.2165	7.403E−013	−1.761E−012	−2.5E−012
382	−2.7E−013	−1E−011	2.2165	1.611E−012	−4.205E−012	−6.5E−012
383	−5.5E−012	−2E−011	2.2165	2.307E−012	−5.457E−012	−8.4E−012
384	−2.3E−012	−4E−011	2.2165	4.532E−012	−1.226E−011	−1.9E−011
385	−1.4E−011	−6E−011	2.2165	6.591E−012	−1.588E−011	−2.5E−011
386	−7.0E−012	−1E−010	2.2165	1.269E−011	−3.498E−011	−5.6E−011
387	−3.9E−011	−2E−010	2.2165	1.918E−011	−4.669E−011	−7.3E−011
388	−2.3E−011	−3E−010	2.2165	3.621E−011	−9.865E−011	−1.6E−010
389	−1.1E−010	−5E−010	2.2165	5.578E−011	−1.364E−010	−2.1E−010
390	−6.8E−011	−9E−010	2.2165	1.039E−010	−2.817E−010	−4.5E−010
391	−3.1E−010	−2E−009	2.2165	1.614E−010	−3.987E−010	−6.3E−010
392	−2.0E−010	−3E−009	2.2165	2.979E−010	−8.038E−010	−1.3E−009
393	−9E−010	−4E−009	2.2165	4.668E−010	−1.164E−009	−1.8E−009
394	−5.8E−010	−7E−009	2.2165	8.552E−010	−2.296E−009	−3.7E−009
395	−2.6E−009	−1E−008	2.2165	1.350E−009	−3.393E−009	−5.4E−009
396	−1.6E−009	−2E−008	2.2165	2.458E−009	−6.560E−009	−1.0E−008
397	−7.7E−009	−4E−008	2.2165	3.899E−009	−9.888E−009	−1.6E−008
398	−4.3E−009	−6E−008	2.2165	7.071E−009	−1.874E−008	−3.0E−008
399	−2.3E−008	−1E−007	2.2165	1.125E−008	−2.883E−008	−4.6E−008
400	−1.1E−008	−2E−007	2.2165	2.036E−008	−5.355E−008	−8.4E−008
401	−7.0E−008	−3E−007	2.2165	3.243E−008	−8.417E−008	−1.3E−007
402	−2.3E−008	−5E−007	2.2165	5.867E−008	−1.529E−007	−2.4E−007
403	−2.2E−007	−9E−007	2.2165	9.339E−008	−2.463E−007	−3.9E−007
404	−3.7E−008	−1E−006	2.2165	1.693E−007	−4.359E−007	−6.8E−007
405	−6.8E−007	−3E−006	2.2165	2.685E−007	−7.233E−007	−1.2E−006
406	1.44E−008	−4E−006	2.2165	4.891E−007	−1.241E−006	−1.9E−006
407	−2.2E−006	−8E−006	2.2165	7.704E−007	−2.137E−006	−3.4E−006
408	5.28E−007	−1E−005	2.2165	1.416E−006	−3.522E−006	−5.4E−006
409	−7.3E−006	−2E−005	2.2164	2.206E−006	−6.367E−006	−1.0E−005
410	3.46E−006	−3E−005	2.2164	4.106E−006	−9.971E−006	−1.5E−005
411	−2.5E−005	−7E−005	2.2163	6.300E−006	−1.923E−005	−3.0E−005
412	1.78E−005	−7E−005	2.2161	1.195E−005	−2.82E−005	−4.1E−005
413	−8.7E−005	−0.00023	2.2158	1.798E−005	−5.929E−005	−8.9E−005
414	8.39E−005	−0.00018	2.2153	3.503E−005	−8.021E−005	−0.0001119
415	−0.000321	−0.00073	2.2144	5.158E−005	−0.00018902	−0.0002701
416	0.00039323	−0.00040	2.2128	0.00010459	−0.00023494	−0.0003019
417	−0.001295	−0.00247	2.2102	0.00015287	−0.00063891	−0.0008465
418	0.0020145	−0.00056	2.2055	0.00032964	−0.00076194	−0.0008621
419	−0.006291	−0.00969	2.1967	0.0005068	−0.0024157	−0.0029059
420	0.012779	0.0028793	2.1793	0.0012632	−0.0032786	−0.0031784

421	−0.037796	−0.04855	2.1255	0.0015614	−0.012957	−0.012956
422	0.080882	0.043625	2.0092	0.013804	−0.0076766	−0.051928
423	−0.026163	0.056051	2.2586	−0.0026513	0.09443	0.29655
424	0.12476	0.14775	2.2317	0.00059656	−0.17378	−0.24358
425	0.031499	0.024142	2.0661	0.019639	0.017146	0.030482
426	−0.028413	−0.03356	2.122	0.0064352	0.015209	0.020131
427	0.005954	0.0024288	2.1767	0.002803	0.0077935	0.0080758
428	−0.005728	−0.00743	2.1914	0.001504	0.0043544	0.0043194
429	0.0012625	0.0003362	2.2053	0.00096815	0.0020294	0.0022117
430	−0.001467	−0.00204	2.2089	0.00049055	0.0012679	0.0012878
431	0.00028079	−7E−006	2.2129	0.00033353	0.0006382	0.00070694
432	−0.000413	−0.00061	2.2141	0.00016992	0.00040537	0.00042664
433	5.74E−005	−4E−005	2.2153	0.00011529	0.00021449	0.00023973
434	−0.000121	−0.00019	2.2157	5.956E−005	0.00013508	0.00014592
435	9.13E−006	−2E−005	2.2161	3.984E−005	7.3687E−005	8.271E−005
436	−3.6E−005	−6E−005	2.2162	2.09E−005	4.5831E−005	5.037E−005
437	2.47E−007	−1E−005	2.2164	1.376E−005	2.55E−005	2.867E−005
438	−1.1E−005	−2E−005	2.2164	7.323E−006	1.5688E−005	1.743E−005
439	−7.1E−007	−5E−006	2.2165	4.748E−006	8.8455E−006	9.948E−006
440	−3.4E−006	−6E−006	2.2165	2.562E−006	5.3963E−006	6.037E−006
441	−4.6E−007	−2E−006	2.2165	1.639E−006	3.0702E−006	3.453E−006
442	−1.1E−006	−2E−006	2.2165	8.949E−007	1.8618E−006	2.092E−006
443	−2.2E−007	−7E−007	2.2165	5.661E−007	1.0656E−006	1.198E−006
444	−3.4E−007	−7E−007	2.2165	3.122E−007	6.4362E−007	7.250E−007
445	−9.3E−008	−3E−007	2.2165	1.956E−007	3.6978E−007	4.159E−007
446	−1.1E−007	−2E−007	2.2165	1.088E−007	2.2279E−007	2.513E−007
447	−3.7E−008	−9E−008	2.2165	6.764E−008	1.2828E−007	1.443E−007
448	−3.5E−008	−8E−008	2.2165	3.791E−008	7.7195E−008	8.716E−008
449	−1.4E−008	−3E−008	2.2165	2.340E−008	4.4488E−008	5.005E−008
450	−1.2E−008	−3E−008	2.2165	1.319E−008	2.6766E−008	3.023E−008
451	−5.3E−009	−1E−008	2.2165	8.101E−009	1.5424E−008	1.736E−008
452	−3.8E−009	−9E−009	2.2165	4.589E−009	9.2862E−009	1.049E−008
453	−2.0E−009	−4E−009	2.2165	2.805E−009	5.3465E−009	6.019E−009
454	−1.2E−009	−3E−009	2.2165	1.595E−009	3.2231E−009	3.641E−009
455	−7.2E−010	−1E−009	2.2165	9.720E−010	1.8528E−009	2.087E−009
456	−4.1E−010	−1E−009	2.2165	5.544E−010	1.1191E−009	1.264E−009
457	−2.6E−010	−5E−010	2.2165	3.369E−010	6.4192E−010	7.231E−010
458	−1.4E−010	−4E−010	2.2165	1.926E−010	3.8864E−010	4.390E−010
459	−9.4E−011	−2E−010	2.2165	1.169E−010	2.2248E−010	2.505E−010
460	−4.6E−011	−1E−010	2.2165	6.681E−011	1.3517E−010	1.524E−010
461	−3.4E−011	−6E−011	2.2165	4.053E−011	7.6944E−011	8.679E−011
462	−1.5E−011	−4E−011	2.2165	2.324E−011	4.6916E−011	5.305E−011
463	−1.2E−011	−2E−011	2.2165	1.404E−011	2.6755E−011	2.996E−011
464	−4.9E−012	−1E−011	2.2165	8.110E−012	1.6279E−011	1.855E−011
465	−4.4E−012	−8E−012	2.2165	4.884E−012	9.2683E−012	1.043E−011
466	−1.8E−012	−5E−012	2.2165	2.862E−012	5.5223E−012	6.325E−012
467	−1.6E−012	−3E−012	2.2165	1.715E−012	3.1461E−012	3.679E−012

468	−6.2E−013	−2E−012	2.2165	1.001E−012	1.9161E−012	2.154E−012
469	−7.6E−013	−1E−012	2.2165	5.718E−013	1.0433E−012	1.197E−012
470	−2.1E−013	−6E−013	2.2165	3.269E−013	6.5633E−013	7.832E−013
471	−9.6E−014	−2E−013	2.2165	2.668E−013	4.6078E−013	4.689E−013
472	−2.4E−013	−4E−013	2.2165	5.685E−014	3.4788E−013	4.954E−013
473	−1.4E−013	−2E−013	2.2165	1.344E−013	2.1352E−013	3.257E−013
474	1.11E−013	1.0E−013	2.2165	3.491E−014	1.6856E−013	1.436E−013
475	−8.7E−014	−1E−013	2.2165	−5.80E−015	3.8478E−015	2.886E−015
476	9.46E−014	−4E−015	2.2165	−3.13E−014	−6.377E−014	−6.8E−014
477	3.21E−014	1.4E−013	2.2165	4.089E−014	−7.837E−014	−7.5E−014
478	4.07E−014	−2E−014	2.2165	1.496E−013	5.6146E−014	2.828E−014
479	−2.8E−013	−3E−013	2.2165	8.777E−015	2.5231E−013	2.418E−013
480	−3.8E−013	−5E−013	2.2165	−5.61E−014	−3.964E−014	−1.2E−014
481	−2.0E−013	−1E−013	2.2165	−3.90E−014	−1.864E−014	−7.8E−014
482	7.91E−015	1.2E−013	2.2165	−3.55E−014	−5.239E−014	−1.1E−013
483	1.71E−013	1.6E−013	2.2165	8.276E−015	−3.109E−014	5.377E−014
484	−1.7E−013	−1E−013	2.2165	−1.07E−013	4.9724E−014	7.762E−014
485	7.27E−014	9.6E−014	2.2165	−8.44E−014	−1.768E−013	−8.8E−014
486	2.86E−014	6.0E−014	2.2165	6.115E−014	1.7371E−014	−1.5E−013
487	−3.5E−015	−3E−014	2.2165	−3.25E−015	−1.891E−014	2.408E−014
488	−3.7E−013	−3E−013	2.2165	4.462E−014	1.2056E−013	−5.2E−014
489	−1.2E−013	−1E−013	2.2165	−3.29E−014	−8.605E−014	1.265E−014
490	3.09E−014	−6E−014	2.2165	7.291E−014	7.9635E−016	−2.6E−013
491	−2.4E−013	−2E−013	2.2165	−5.41E−014	−1.301E−014	1.520E−013
492	1.74E−014	7.6E−014	2.2165	−3.67E−015	5.3658E−014	−1.4E−013
493	−3.3E−013	−3E−013	2.2165	6.031E−014	−9.436E−014	3.396E−014
494	1.20E−013	7.7E−014	2.2165	3.064E−014	9.2671E−014	1.543E−013
495	−1.5E−013	−1E−013	2.2165	−4.95E−014	−1.169E−014	1.606E−013
496	−2.6E−013	−3E−013	2.2165	−1.04E−013	7.7317E−015	1.245E−013
497	1.12E−013	1.2E−013	2.2165	−9.38E−014	−2.668E−014	3.033E−013
498	−1.7E−013	−3E−013	2.2165	−8.03E−014	−7.251E−014	−3.4E−013
499	−8.0E−014	6.9E−014	2.2165	2.993E−015	−1.507E−013	−9.1E−014
500	1.97E−014	−4E−014	2.2165	−2.15E−014	5.2773E−014	−1.5E−013
501	3.16E−014	5.1E−014	2.2165	3.719E−014	−2.376E−013	−6.4E−014
502	−3.8E−013	−4E−013	2.2165	−7.87E−015	4.7867E−014	−4.7E−014
503	−4.1E−014	6.7E−014	2.2165	1.899E−014	2.1619E−013	2.443E−013
504	−4.0E−014	−1E−013	2.2165	−1.78E−014	2.7149E−013	2.645E−013
505	−8.4E−014	−7E−014	2.2165	1.291E−014	−2.227E−014	−3.3E−013
506	−5.2E−014	−2E−013	2.2165	−1.34E−013	−2.213E−013	4.854E−014
507	−6.1E−013	−6E−013	2.2165	−9.48E−014	−2.124E−013	1.326E−013
508	8.93E−014	2.1E−013	2.2165	−9.93E−015	−1.881E−013	−2.3E−013
509	1.81E−013	1.5E−013	2.2165	−3.53E−014	1.454E−014	−5.6E−016
510	−2.8E−014	−2E−013	2.2165	−3.97E−014	−2.518E−013	−3.1E−013
511	−2.6E−014	9.6E−014	2.2165	9.156E−014	−6.466E−014	−1.7E−013
512	−2.8E−013	−3E−013	2.2165	2.096E−013	−9.699E−014	−4.5E−013
513	−4.6E−013	−6E−013	2.2165	2.030E−013	−2.358E−013	−7.9E−013
514	−1.7E−013	−6E−013	2.2165	2.669E−013	−4.683E−013	−5.9E−013

515	−1.1E−012	−1E−012	2.2165	5.007E−013	−1.080E−012	−1.5E−012
516	−1.9E−013	−1E−012	2.2165	9.238E−013	−1.942E−012	−2.1E−012
517	−1.9E−012	−3E−012	2.2165	1.643E−012	−3.250E−012	−3.6E−012
518	−1.7E−012	−5E−012	2.2165	2.781E−012	−5.865E−012	−7E−012
519	−4.1E−012	−8E−012	2.2165	4.822E−012	−8.979E−012	−1.0E−011
520	−5.3E−012	−1E−011	2.2165	8.271E−012	−1.612E−011	−1.9E−011
521	−1.2E−011	−2E−011	2.2165	1.412E−011	−2.654E−011	−3.0E−011
522	−1.5E−011	−4E−011	2.2165	2.301E−011	−4.675E−011	−5.3E−011
523	−3.4E−011	−6E−011	2.2165	4.054E−011	−7.687E−011	−8.7E−011
524	−4.6E−011	−1E−010	2.2165	6.69E−011	−1.354E−010	−1.5E−010
525	−9.4E−011	−2E−010	2.2165	1.168E−010	−2.222E−010	−2.5E−010
526	−1.4E−010	−4E−010	2.2165	1.926E−010	−3.887E−010	−4.4E−010
527	−2.6E−010	−5E−010	2.2165	3.369E−010	−6.417E−010	−7.2E−010
528	−4.1E−010	−1E−009	2.2165	5.544E−010	−1.119E−009	−1.3E−009
529	−7.2E−010	−1E−009	2.2165	9.719E−010	−1.853E−009	−2.1E−009
530	−1.2E−009	−3E−009	2.2165	1.595E−009	−3.223E−009	−3.6E−009
531	−2.0E−009	−4E−009	2.2165	2.805E−009	−5.347E−009	−6.0E−009
532	−3.8E−009	−9E−009	2.2165	4.589E−009	−9.286E−009	−1.0E−008
533	−5.3E−009	−1E−008	2.2165	8.101E−009	−1.542E−008	−1.7E−008
534	−1.2E−008	−3E−008	2.2165	1.319E−008	−2.677E−008	−3.0E−008
535	−1.4E−008	−3E−008	2.2165	2.340E−008	−4.449E−008	−5.0E−008
536	−3.5E−008	−8E−008	2.2165	3.791E−008	−7.719E−008	−8.7E−008
537	−3.7E−008	−9E−008	2.2165	6.764E−008	−1.283E−007	−1.4E−007
538	−1.1E−007	−2E−007	2.2165	1.088E−007	−2.228E−007	−2.5E−007
539	−9.3E−008	−3E−007	2.2165	1.956E−007	−3.698E−007	−4.2E−007
540	−3.4E−007	−7E−007	2.2165	3.122E−007	−6.436E−007	−7.2E−007
541	−2.2E−007	−7E−007	2.2165	5.661E−007	−1.066E−006	−1.2E−006
542	−1.1E−006	−2E−006	2.2165	8.949E−007	−1.862E−006	−2.1E−006
543	−4.6E−007	−2E−006	2.2165	1.639E−006	−3.070E−006	−3.5E−006
544	−3.4E−006	−6E−006	2.2165	2.562E−006	−5.396E−006	−6.0E−006
545	−7.1E−007	−5E−006	2.2165	4.748E−006	−8.846E−006	−9.9E−006
546	−1.1E−005	−2E−005	2.2164	7.323E−006	−1.569E−005	−1.7E−005
547	2.47E−007	−1E−005	2.2164	1.376E−005	−2.55E−005	−2.9E−005
548	−3.6E−005	−6E−005	2.2162	2.09E−005	−4.583E−005	−5.0E−005
549	9.13E−006	−2E−005	2.2161	3.984E−005	−7.369E−005	−8.3E−005
550	−0.000121	−0.00019	2.2157	5.956E−005	−0.00013508	−0.0001459
551	5.74E−005	−4E−005	2.2153	0.00011529	−0.00021449	−0.0002397
552	−0.000413	−0.00061	2.2141	0.00016992	−0.00040537	−0.0004266
553	0.00028079	−7E−006	2.2129	0.00033353	−0.0006382	−0.0007069
554	−0.001467	−0.00204	2.2089	0.00049055	−0.0012679	−0.0012878
555	0.0012625	0.0003362	2.2053	0.00096815	−0.0020294	−0.0022117
556	−0.005728	−0.00743	2.1914	0.001504	−0.0043544	−0.0043194
557	0.005954	0.0024288	2.1767	0.002803	−0.0077935	−0.0080758
558	−0.028413	−0.03356	2.122	0.0064352	−0.015209	−0.020131
559	0.031499	0.024142	2.0661	0.019639	−0.017146	−0.030482
560	0.12476	0.14775	2.2317	0.00059656	0.17378	0.24358
561	0.14775	0.12476	2.2317	0.00059656	−0.24358	−0.17378

562	0.024142	0.031499	2.0661	0.019639	0.030482	0.017146
563	−0.033561	−0.02841	2.122	0.0064352	0.020131	0.015209
564	0.0024288	0.005954	2.1767	0.002803	0.0080758	0.0077935
565	−0.007430	−0.00573	2.1914	0.001504	0.0043194	0.0043544
566	0.00033624	0.0012625	2.2053	0.00096815	0.0022117	0.0020294
567	−0.002043	−0.00147	2.2089	0.00049055	0.0012878	0.0012679
568	−7.1E−006	0.0002808	2.2129	0.00033353	0.00070694	0.0006382
569	−0.000611	−0.00041	2.2141	0.00016992	0.00042664	0.00040537
570	−3.9E−005	5.7E−005	2.2153	0.00011529	0.00023973	0.00021449
571	−0.000189	−0.00012	2.2157	5.956E−005	0.00014592	0.00013508
572	−2.4E−005	9.1E−006	2.2161	3.984E−005	8.2705E−005	7.369E−005
573	−6.0E−005	−4E−005	2.2162	2.09E−005	5.0368E−005	4.583E−005
574	−1.1E−005	2.5E−007	2.2164	1.376E−005	2.8667E−005	2.55E−005
575	−1.9E−005	−1E−005	2.2164	7.323E−006	1.7431E−005	1.569E−005
576	−4.6E−006	−7E−007	2.2165	4.748E−006	9.9483E−006	8.846E−006
577	−6.2E−006	−3E−006	2.2165	2.562E−006	6.0371E−006	5.396E−006
578	−1.8E−006	−5E−007	2.2165	1.639E−006	3.453E−006	3.070E−006
579	−2.1E−006	−1E−006	2.2165	8.949E−007	2.0918E−006	1.862E−006
580	−6.9E−007	−2E−007	2.2165	5.661E−007	1.1984E−006	1.066E−006
581	−6.8E−007	−3E−007	2.2165	3.122E−007	7.2498E−007	6.436E−007
582	−2.6E−007	−9E−008	2.2165	1.956E−007	4.1585E−007	3.698E−007
583	−2.3E−007	−1E−007	2.2165	1.088E−007	2.5134E−007	2.228E−007
584	−9.3E−008	−4E−008	2.2165	6.764E−008	1.4428E−007	1.283E−007
585	−7.7E−008	−4E−008	2.2165	3.791E−008	8.7161E−008	7.719E−008
586	−3.3E−008	−1E−008	2.2165	2.340E−008	5.0048E−008	4.449E−008
587	−2.6E−008	−1E−008	2.2165	1.319E−008	3.0235E−008	2.677E−008
588	−1.2E−008	−5E−009	2.2165	8.101E−009	1.7357E−008	1.542E−008
589	−8.9E−009	−4E−009	2.2165	4.589E−009	1.0491E−008	9.286E−009
590	−4.2E−009	−2E−009	2.2165	2.805E−009	6.0188E−009	5.346E−009
591	−3.0E−009	−1E−009	2.2165	1.595E−009	3.6413E−009	3.223E−009
592	−1.5E−009	−7E−010	2.2165	9.720E−010	2.0866E−009	1.853E−009
593	−1.0E−009	−4E−010	2.2165	5.544E−010	1.2642E−009	1.119E−009
594	−5.2E−010	−3E−010	2.2165	3.369E−010	7.2322E−010	6.420E−010
595	−3.6E−010	−1E−010	2.2165	1.926E−010	4.3912E−010	3.888E−010
596	−1.8E−010	−9E−011	2.2165	1.168E−010	2.5072E−010	2.224E−010
597	−1.2E−010	−5E−011	2.2165	6.685E−011	1.5253E−010	1.351E−010
598	−6.4E−011	−3E−011	2.2165	4.052E−011	8.6832E−011	7.690E−011
599	−4.3E−011	−1E−011	2.2165	2.328E−011	5.3208E−011	4.701E−011
600	−2.2E−011	−1E−011	2.2165	1.403E−011	3.0101E−011	2.667E−011
601	−1.5E−011	−5E−012	2.2165	8.034E−012	1.8262E−011	1.620E−011
602	−7.7E−012	−4E−012	2.2165	4.940E−012	1.0307E−011	9.156E−012
603	−5.3E−012	−2E−012	2.2165	2.822E−012	6.3096E−012	5.636E−012
604	−2.8E−012	−2E−012	2.2165	1.744E−012	3.6857E−012	3.200E−012
605	−1.8E−012	−6E−013	2.2165	9.788E−013	2.3062E−012	2.064E−012
606	−1.2E−012	−8E−013	2.2165	5.442E−013	1.2967E−012	1.275E−012
607	−7.9E−013	−3E−013	2.2165	3.035E−013	6.5862E−013	6.481E−013
608	−4.5E−013	−3E−013	2.2165	2.256E−013	5.4104E−013	3.645E−013

609	−2.1E−013	−2E−014	2.2165	1.480E−013	3.5137E−013	2.965E−013
610	−3.1E−013	−4E−013	2.2165	7.287E−014	1.9097E−013	1.699E−013
611	6.51E−014	1.7E−013	2.2165	1.065E−013	5.1419E−014	9.180E−014
612	−4.7E−014	−6E−014	2.2165	7.127E−014	1.6521E−013	−6.2E−014
613	−4.4E−013	−5E−013	2.2165	−5.18E−014	−1.408E−013	1.330E−013
614	−1.8E−013	−8E−014	2.2165	1.004E−013	8.1656E−014	−1.1E−013
615	−1.6E−013	−1E−013	2.2165	8.377E−014	1.3664E−013	−9.0E−014
616	1.18E−013	−5E−014	2.2165	7.998E−014	8.2134E−014	8.279E−014
617	1.35E−013	1.4E−013	2.2165	−2.90E−014	2.0291E−013	1.040E−013
618	−1.9E−013	−1E−013	2.2165	−6.18E−014	2.6581E−013	3.739E−015
619	−1.0E−013	−2E−013	2.2165	1.373E−014	1.7857E−013	−1.7E−013
620	4.77E−015	−1E−013	2.2165	−8.32E−014	−1.188E−013	1.097E−013
621	1.16E−013	5.7E−014	2.2165	−6.87E−014	−1.366E−013	−1.1E−013
622	7.52E−015	4.0E−014	2.2165	−4.61E−015	−3.911E−014	−9.0E−015
623	1.52E−013	4.5E−014	2.2165	−4.95E−014	−9.830E−014	2.882E−013
624	−6.1E−014	5.5E−014	2.2165	−1.19E−015	1.7084E−013	−3.2E−014
625	6.74E−014	6.6E−014	2.2165	−1.91E−014	−1.558E−013	9.716E−014
626	−1.7E−014	2.9E−014	2.2165	4.665E−014	−5.609E−014	1.699E−013
627	−3.9E−014	−8E−014	2.2165	5.579E−014	5.2135E−015	2.035E−013
628	−1.4E−013	−5E−014	2.2165	4.238E−014	6.9858E−014	9.113E−014
629	−1.9E−013	−2E−013	2.2165	−8.17E−014	2.2505E−013	1.281E−016
630	3.48E−014	2.3E−013	2.2165	−6.83E−017	9.392E−014	−2.4E−013
631	−2.9E−013	−5E−013	2.2165	−1.85E−014	2.933E−013	1.789E−013
632	−1.2E−013	−9E−014	2.2165	1.092E−014	−5.169E−014	−9.4E−014
633	1.46E−013	1.0E−013	2.2165	8.689E−015	3.541E−013	1.465E−013
634	−5.5E−014	−1E−013	2.2165	−6.18E−015	1.2748E−013	−2.5E−013
635	−7.3E−014	8.9E−014	2.2165	−1.80E−014	−2.204E−013	2.308E−013
636	−1.5E−013	−3E−013	2.2165	−1.92E−014	1.2362E−015	−1.4E−013
637	−2E−014	6.8E−014	2.2165	3.119E−014	−1.218E−013	−3.3E−013
638	4.19E−013	3.4E−013	2.2165	6.056E−014	3.4695E−014	−2.8E−013
639	−1.0E−013	−9E−014	2.2165	5.482E−014	−6.928E−014	−2.5E−013
640	−3.0E−013	−3E−013	2.2165	5.879E−014	−8.940E−014	−1.7E−013
641	2.04E−013	1.3E−013	2.2165	3.168E−014	1.0864E−013	1.486E−013
642	3.23E−013	3.1E−013	2.2165	−1.06E−014	8.2752E−014	3.489E−013
643	−9.3E−014	−2E−013	2.2165	2.135E−014	−1.840E−013	4.074E−013
644	−4.1E−013	−4E−013	2.2165	1.841E−014	−1.926E−013	1.526E−013
645	−9.5E−014	−2E−014	2.2165	5.950E−014	−2.541E−013	−1.2E−014
646	−4.7E−013	−6E−013	2.2165	6.505E−014	1.3685E−013	1.267E−013
647	3.11E−014	−7E−014	2.2165	6.287E−014	−2.914E−013	−2.0E−013
648	−1.2E−015	2.4E−013	2.2165	6.719E−014	−2.682E−014	−3.1E−013
649	−3.8E−014	8.9E−014	2.2165	1.148E−013	−1.257E−013	1.930E−013
650	−4.7E−013	−4E−013	2.2165	1.153E−013	−4.026E−013	−3.0E−013
651	−4.2E−013	−9E−014	2.2165	3.680E−013	−1.189E−012	−5.3E−013
652	−1E−012	−5E−013	2.2165	6.006E−013	−1.029E−012	−9.7E−013
653	−1.7E−012	−7E−013	2.2165	9.362E−013	−2.299E−012	−2.1E−012
654	−2.3E−012	−1E−012	2.2165	1.833E−012	−4.108E−012	−3.5E−012
655	−5.2E−012	−1E−012	2.2165	2.830E−012	−6.274E−012	−5.7E−012

656	−7.4E−012	−4E−012	2.2165	4.954E−012	−1.057E−011	−9.2E−012
657	−1.5E−011	−5E−012	2.2165	8.011E−012	−1.781E−011	−1.6E−011
658	−2.3E−011	−1E−011	2.2165	1.395E−011	−3.026E−011	−2.7E−011
659	−4.3E−011	−2E−011	2.2165	2.322E−011	−5.329E−011	−4.7E−011
660	−6.4E−011	−3E−011	2.2165	4.057E−011	−8.712E−011	−7.7E−011
661	−1.2E−010	−5E−011	2.2165	6.699E−011	−1.527E−010	−1.4E−010
662	−1.8E−010	−9E−011	2.2165	1.169E−010	−2.505E−010	−2.2E−010
663	−3.6E−010	−1E−010	2.2165	1.925E−010	−4.394E−010	−3.9E−010
664	−5.2E−010	−3E−010	2.2165	3.368E−010	−7.230E−010	−6.4E−010
665	−1.0E−009	−4E−010	2.2165	5.544E−010	−1.264E−009	−1.1E−009
666	−1.5E−009	−7E−010	2.2165	9.720E−010	−2.087E−009	−1.9E−009
667	−3.0E−009	−1E−009	2.2165	1.595E−009	−3.642E−009	−3.2E−009
668	−4.2E−009	−2E−009	2.2165	2.806E−009	−6.018E−009	−5.3E−009
669	−8.9E−009	−4E−009	2.2165	4.589E−009	−1.049E−008	−9.3E−009
670	−1.2E−008	−5E−009	2.2165	8.101E−009	−1.736E−008	−1.5E−008
671	−2.6E−008	−1E−008	2.2165	1.319E−008	−3.023E−008	−2.7E−008
672	−3.3E−008	−1E−008	2.2165	2.340E−008	−5.005E−008	−4.4E−008
673	−7.7E−008	−4E−008	2.2165	3.791E−008	−8.716E−008	−7.7E−008
674	−9.3E−008	−4E−008	2.2165	6.764E−008	−1.443E−007	−1.3E−007
675	−2.3E−007	−1E−007	2.2165	1.088E−007	−2.513E−007	−2.2E−007
676	−2.6E−007	−9E−008	2.2165	1.956E−007	−4.159E−007	−3.7E−007
677	−6.8E−007	−3E−007	2.2165	3.122E−007	−7.250E−007	−6.4E−007
678	−6.9E−007	−2E−007	2.2165	5.661E−007	−1.198E−006	−1.1E−006
679	−2.1E−006	−1E−006	2.2165	8.949E−007	−2.092E−006	−1.9E−006
680	−1.8E−006	−5E−007	2.2165	1.639E−006	−3.453E−006	−3.1E−006
681	−6.2E−006	−3E−006	2.2165	2.562E−006	−6.037E−006	−5.4E−006
682	−4.6E−006	−7E−007	2.2165	4.748E−006	−9.948E−006	−8.8E−006
683	−1.9E−005	−1E−005	2.2164	7.323E−006	−1.743E−005	−1.6E−005
684	−1.1E−005	2.5E−007	2.2164	1.376E−005	−2.867E−005	−2.6E−005
685	−6.0E−005	−4E−005	2.2162	2.09E−005	−5.037E−005	−4.6E−005
686	−2.4E−005	9.1E−006	2.2161	3.984E−005	−8.271E−005	−7.4E−005
687	−0.000189	−0.00012	2.2157	5.956E−005	−0.00014592	−0.0001351
688	−3.9E−005	5.7E−005	2.2153	0.00011529	−0.00023973	−0.0002145
689	−0.000611	−0.00041	2.2141	0.00016992	−0.00042664	−0.0004054
690	−7.1E−006	0.0002808	2.2129	0.00033353	−0.00070694	−0.0006382
691	−0.002043	−0.00147	2.2089	0.00049055	−0.0012878	−0.0012679
692	0.00033624	0.0012625	2.2053	0.00096815	−0.0022117	−0.0020294
693	−0.007430	−0.00573	2.1914	0.001504	−0.0043194	−0.0043544
694	0.0024288	0.005954	2.1767	0.002803	−0.0080758	−0.0077935
695	−0.033561	−0.02841	2.122	0.0064352	−0.020131	−0.015209
696	0.024142	0.031499	2.0661	0.019639	−0.030482	−0.017146
697	0.14775	0.12476	2.2317	0.00059656	0.24358	0.17378
698	0.056051	−0.02616	2.2586	−0.0026513	−0.29655	−0.09443
699	0.043625	0.080882	2.0092	0.013804	0.051928	0.0076766
700	−0.048546	−0.03780	2.1255	0.0015614	0.012956	0.012957
701	0.0028793	0.012779	2.1793	0.0012632	0.0031784	0.0032786
702	−0.009690	−0.00629	2.1967	0.0005068	0.0029059	0.0024157

703	−0.000557	0.0020145	2.2055	0.00032964	0.00086209	0.00076194
704	−0.002468	−0.00129	2.2102	0.00015287	0.00084654	0.00063891
705	−0.000402	0.0003932	2.2128	0.00010459	0.00030191	0.00023494
706	−0.000730	−0.00032	2.2144	5.158E−005	0.00027006	0.00018902
707	−0.000180	8.4E−005	2.2153	3.503E−005	0.00011186	8.021E−005
708	−0.000230	−9E−005	2.2158	1.798E−005	8.902E−005	5.929E−005
709	−7.2E−005	1.8E−005	2.2161	1.195E−005	4.123E−005	2.82E−005
710	−7.5E−005	−2E−005	2.2163	6.300E−006	2.9771E−005	1.923E−005
711	−2.7E−005	3.5E−006	2.2164	4.106E−006	1.4981E−005	9.971E−006
712	−2.5E−005	−7E−006	2.2164	2.206E−006	1.0035E−005	6.367E−006
713	−1.0E−005	5.3E−007	2.2165	1.416E−006	5.3797E−006	3.522E−006
714	−8.2E−006	−2E−006	2.2165	7.704E−007	3.4E−006	2.137E−006
715	−3.7E−006	1.4E−008	2.2165	4.891E−007	1.9159E−006	1.241E−006
716	−2.8E−006	−7E−007	2.2165	2.685E−007	1.1562E−006	7.233E−007
717	−1.3E−006	−4E−008	2.2165	1.693E−007	6.7855E−007	4.359E−007
718	−9.3E−007	−2E−007	2.2165	9.339E−008	3.9417E−007	2.463E−007
719	−4.7E−007	−2E−008	2.2165	5.867E−008	2.3945E−007	1.529E−007
720	−3.2E−007	−7E−008	2.2165	3.243E−008	1.3463E−007	8.417E−008
721	−1.7E−007	−1E−008	2.2165	2.036E−008	8.4305E−008	5.355E−008
722	−1.1E−007	−2E−008	2.2165	1.125E−008	4.603E−008	2.883E−008
723	−5.8E−008	−4E−009	2.2165	7.071E−009	2.9642E−008	1.874E−008
724	−3.7E−008	−8E−009	2.2165	3.899E−009	1.5746E−008	9.887E−009
725	−2.0E−008	−2E−009	2.2165	2.458E−009	1.0416E−008	6.559E−009
726	−1.3E−008	−3E−009	2.2165	1.350E−009	5.3861E−009	3.392E−009
727	−7.2E−009	−6E−010	2.2165	8.554E−010	3.6592E−009	2.296E−009
728	−4.4E−009	−9E−010	2.2165	4.668E−010	1.8417E−009	1.164E−009
729	−2.5E−009	−2E−010	2.2165	2.979E−010	1.286E−009	8.038E−010
730	−1.5E−009	−3E−010	2.2165	1.613E−010	6.2917E−010	3.988E−010
731	−8.7E−010	−7E−011	2.2165	1.038E−010	4.5231E−010	2.816E−010
732	−5.3E−010	−1E−010	2.2165	5.568E−011	2.1466E−010	1.366E−010
733	−3.0E−010	−2E−011	2.2165	3.619E−011	1.5918E−010	9.884E−011
734	−1.8E−010	−4E−011	2.2165	1.921E−011	7.3069E−011	4.673E−011
735	−1.1E−010	−7E−012	2.2165	1.262E−011	5.5946E−011	3.469E−011
736	−6.3E−011	−1E−011	2.2165	6.596E−012	2.4936E−011	1.587E−011
737	−3.7E−011	−2E−012	2.2165	4.392E−012	1.9648E−011	1.225E−011
738	−2.2E−011	−5E−012	2.2165	2.217E−012	8.302E−012	5.456E−012
739	−1.3E−011	−5E−013	2.2165	1.554E−012	6.9005E−012	4.185E−012
740	−7.8E−012	−2E−012	2.2165	7.990E−013	2.7721E−012	1.778E−012
741	−4.5E−012	−3E−013	2.2165	5.572E−013	2.6296E−012	1.654E−012
742	−2.6E−012	−7E−013	2.2165	2.774E−013	7.0591E−013	6.902E−013
743	−1.7E−012	−1E−013	2.2165	1.499E−013	8.3254E−013	6.661E−013
744	−9.5E−013	−3E−013	2.2165	1.187E−013	1.5617E−013	1.213E−013
745	−8.1E−013	−2E−013	2.2165	5.572E−014	4.739E−013	2.386E−013
746	−3.7E−013	−6E−014	2.2165	1.029E−013	1.8932E−013	6.154E−014
747	−4.5E−013	−2E−013	2.2165	−1.32E−014	−1.505E−013	3.733E−014
748	−3.9E−013	−3E−013	2.2165	−4.21E−014	−2.370E−013	−6.8E−014
749	−1.7E−014	2.5E−014	2.2165	−1.70E−014	2.0613E−013	7.565E−014

750	−1.4E−013	7.0E−014	2.2165	3.788E−014	−1.106E−013	−1.2E−013
751	1.58E−013	2.6E−013	2.2165	7.189E−014	−2.350E−013	2.326E−013
752	−3.1E−013	−2E−013	2.2165	5.712E−014	3.7206E−013	−6.8E−014
753	−3E−013	−2E−013	2.2165	5.009E−014	−9.487E−014	2.833E−015
754	2.96E−013	2.1E−013	2.2165	−2.01E−015	2.6004E−013	1.264E−013
755	1.17E−013	−4E−014	2.2165	3.997E−014	−2.353E−013	−8.9E−014
756	2.56E−014	9.9E−014	2.2165	−2.55E−014	−2.166E−013	−2.1E−014
757	−1.5E−013	−1E−013	2.2165	−6.02E−014	−2.170E−013	1.336E−013
758	−1.2E−013	−2E−013	2.2165	−6.71E−014	−6.505E−014	−1.3E−013
759	1.68E−013	1.4E−014	2.2165	−3.25E−014	−2.077E−013	−2.3E−014
760	4.19E−013	3.4E−013	2.2165	−3.21E−014	2.1267E−013	−1.2E−013
761	1.37E−013	8.9E−014	2.2165	7.131E−015	2.314E−013	1.722E−013
762	−1.1E−013	−5E−014	2.2165	−4.40E−014	−2.639E−013	−2.4E−013
763	−2.5E−013	−1E−013	2.2165	−1.26E−014	−5.727E−015	−1.0E−013
764	−3.5E−013	−3E−013	2.2165	3.950E−016	−1.934E−013	4.711E−014
765	−3.8E−014	4.8E−014	2.2165	4.210E−014	−4.008E−014	−1.7E−013
766	−6.1E−014	−7E−015	2.2165	3.932E−014	−3.095E−013	−1.5E−015
767	4.42E−013	2.1E−013	2.2165	−1.68E−014	2.3609E−013	−1.1E−013
768	−8.3E−014	−9E−014	2.2165	1.989E−014	−6.032E−013	−4.3E−013
769	−1.5E−013	−3E−013	2.2165	5.040E−014	3.5171E−013	−3.8E−014
770	4.15E−013	3.4E−013	2.2165	−5.87E−016	−3.232E−013	−3.2E−013
771	1.60E−013	−1E−014	2.2165	8.475E−014	1.2054E−013	−1.1E−013
772	−1.5E−013	−3E−013	2.2165	3.917E−014	2.2225E−013	−2.5E−013
773	1.09E−013	8.1E−014	2.2165	7.128E−014	1.1422E−013	1.496E−013
774	−3.6E−013	−5E−013	2.2165	6.915E−014	−1.162E−014	−5.1E−014
775	−4.5E−013	−3E−013	2.2165	8.141E−014	6.3872E−013	2.543E−013
776	−2.6E−013	−1E−013	2.2165	6.985E−014	1.348E−013	2.004E−013
777	1.87E−013	1.8E−013	2.2165	−1.54E−014	−4.413E−013	2.740E−015
778	−1.7E−014	−8E−014	2.2165	3.617E−014	−5.147E−013	2.457E−013
779	−2.0E−013	−1E−013	2.2165	−5.49E−014	3.4391E−013	2.542E−013
780	−1.4E−013	−1E−013	2.2165	−2.34E−014	−5.173E−014	6.947E−014
781	−6.2E−013	−6E−013	2.2165	−2.11E−014	−2.840E−013	−2.4E−014
782	−2.7E−013	−1E−013	2.2165	1.231E−013	−1.036E−014	2.707E−015
783	−3.1E−013	−2E−013	2.2165	1.110E−013	−3.334E−013	8.871E−014
784	−4.9E−013	−4E−013	2.2165	6.574E−014	−3.892E−013	1.594E−013
785	−7.9E−013	−5E−013	2.2165	4.265E−014	−6.688E−014	2.716E−013
786	−1.2E−013	6.0E−014	2.2165	2.391E−014	−3.091E−013	−1.7E−013
787	−8.2E−013	−3E−013	2.2165	1.622E−014	1.749E−013	−2.3E−013
788	−9.2E−013	−4E−013	2.2165	9.082E−014	−8.263E−013	−2.2E−013
789	−1.9E−012	−4E−013	2.2165	1.942E−013	−1.349E−012	−4.1E−013
790	−2.9E−012	−7E−013	2.2165	2.067E−013	−7.033E−013	−2.6E−013
791	−4.6E−012	−3E−013	2.2165	5.462E−013	−2.409E−012	−1.5E−012
792	−8.1E−012	−2E−012	2.2165	7.189E−013	−2.641E−012	−1.7E−012
793	−1.3E−011	−7E−013	2.2165	1.511E−012	−7.162E−012	−4.4E−012
794	−2.2E−011	−5E−012	2.2165	2.288E−012	−8.046E−012	−5.7E−012
795	−3.7E−011	−2E−012	2.2165	4.448E−012	−2.000E−011	−1.2E−011
796	−6.3E−011	−1E−011	2.2165	6.679E−012	−2.446E−011	−1.6E−011

797	−1.1E−010	−7E−012	2.2165	1.269E−011	−5.620E−011	−3.5E−011
798	−1.8E−010	−4E−011	2.2165	1.917E−011	−7.350E−011	−4.7E−011
799	−3.0E−010	−2E−011	2.2165	3.619E−011	−1.595E−010	−9.9E−011
800	−5.3E−010	−1E−010	2.2165	5.578E−011	−2.137E−010	−1.4E−010
801	−8.7E−010	−7E−011	2.2165	1.039E−010	−4.523E−010	−2.8E−010
802	−1.5E−009	−3E−010	2.2165	1.614E−010	−6.283E−010	−4.0E−010
803	−2.5E−009	−2E−010	2.2165	2.978E−010	−1.286E−009	−8.0E−010
804	−4.4E−009	−9E−010	2.2165	4.668E−010	−1.842E−009	−1.2E−009
805	−7.2E−009	−6E−010	2.2165	8.555E−010	−3.660E−009	−2.3E−009
806	−1.3E−008	−3E−009	2.2165	1.350E−009	−5.386E−009	−3.4E−009
807	−2.0E−008	−2E−009	2.2165	2.458E−009	−1.042E−008	−6.6E−009
808	−3.7E−008	−8E−009	2.2165	3.899E−009	−1.575E−008	−9.9E−009
809	−5.8E−008	−4E−009	2.2165	7.071E−009	−2.964E−008	−1.9E−008
810	−1.1E−007	−2E−008	2.2165	1.125E−008	−4.603E−008	−2.9E−008
811	−1.7E−007	−1E−008	2.2165	2.036E−008	−8.431E−008	−5.4E−008
812	−3.2E−007	−7E−008	2.2165	3.243E−008	−1.346E−007	−8.4E−008
813	−4.7E−007	−2E−008	2.2165	5.867E−008	−2.394E−007	−1.5E−007
814	−9.3E−007	−2E−007	2.2165	9.339E−008	−3.942E−007	−2.5E−007
815	−1.3E−006	−4E−008	2.2165	1.693E−007	−6.786E−007	−4.4E−007
816	−2.8E−006	−7E−007	2.2165	2.685E−007	−1.156E−006	−7.2E−007
817	−3.7E−006	1.4E−008	2.2165	4.891E−007	−1.916E−006	−1.2E−006
818	−8.2E−006	−2E−006	2.2165	7.704E−007	−3.4E−006	−2.1E−006
819	−1.0E−005	5.3E−007	2.2165	1.416E−006	−5.380E−006	−3.5E−006
820	−2.5E−005	−7E−006	2.2164	2.206E−006	−1.004E−005	−6.4E−006
821	−2.7E−005	3.5E−006	2.2164	4.106E−006	−1.498E−005	−1.0E−005
822	−7.5E−005	−2E−005	2.2163	6.300E−006	−2.977E−005	−1.9E−005
823	−7.2E−005	1.8E−005	2.2161	1.195E−005	−4.123E−005	−2.8E−005
824	−0.000230	−9E−005	2.2158	1.798E−005	−8.902E−005	−5.9E−005
825	−0.000180	8.4E−005	2.2153	3.503E−005	−0.00011186	−8.0E−005
826	−0.000730	−0.00032	2.2144	5.158E−005	−0.00027006	−0.0001890
827	−0.000402	0.0003932	2.2128	0.00010459	−0.00030191	−0.0002349
828	−0.002468	−0.00129	2.2102	0.00015287	−0.00084654	−0.0006389
829	−0.000557	0.0020145	2.2055	0.00032964	−0.00086209	−0.0007619
830	−0.009690	−0.00629	2.1967	0.0005068	−0.0029059	−0.0024157
831	0.0028793	0.012779	2.1793	0.0012632	−0.0031784	−0.0032786
832	−0.048546	−0.03780	2.1255	0.0015614	−0.012956	−0.012957
833	0.043625	0.080882	2.0092	0.013804	−0.051928	−0.0076766
834	0.056051	−0.02616	2.2586	−0.0026513	0.29655	0.09443
835	2.732	2.7235	2.9466	−8.86E−012	7.4298E−011	0.67014
836	2.2088	2.2543	2.5136	−0.0050237	−0.64668	0.14985
837	1.9808	1.9985	2.2709	−0.01887	−0.49479	0.33185
838	1.9985	1.9808	2.2709	−0.01887	−0.33185	0.49479
839	2.2543	2.2088	2.5136	−0.0050237	−0.14985	0.64668
840	2.732	2.7235	2.9466	−9.07E−012	−7.509E−011	−0.67014
841	−0.19284	−0.05959	2.4502	−3.82E−011	8.0804E−015	0.28885
842	0.14614	0.073965	1.7645	1.091E−011	−1.501E−011	−0.053813
843	−0.079819	−0.09412	2.181	6.670E−012	8.5707E−012	−0.0034843

844	0.030904	0.018333	2.1937	1.408E−012	2.8146E−012	−0.003213
845	−0.012937	−0.01799	2.1929	1.637E−012	8E−013	−0.0008466
846	0.0050713	0.002631	2.2097	2.858E−013	5.3663E−013	−0.0006051
847	−0.002588	−0.00428	2.2089	4.782E−013	2.0402E−013	−0.0003601
848	0.0010282	0.0003161	2.214	1.052E−013	1.7162E−013	−0.0001462
849	−0.000589	−0.00116	2.214	1.447E−013	4.7372E−014	−0.0001423
850	0.00022205	−1E−005	2.2155	3.228E−014	4.9678E−014	−4.1E−005
851	−0.000142	−0.00033	2.2157	4.366E−014	2.9426E−014	−5.3E−005
852	4.72E−005	−3E−005	2.2162	2.468E−014	−1.392E−014	−1.2E−005
853	−3.5E−005	−0.00010	2.2162	1.145E−014	−1.629E−014	−1.9E−005
854	9.21E−006	−2E−005	2.2164	1.630E−014	7.2938E−016	−3.9E−006
855	−8.4E−006	−3E−005	2.2164	−9.74E−016	−3.923E−014	−7.0E−006
856	1.40E−006	−8E−006	2.2165	−1.50E−014	4.6838E−015	−1.3E−006
857	−2.0E−006	−1E−005	2.2165	9.292E−015	2.6008E−014	−2.5E−006
858	2.70E−008	−3E−006	2.2165	−2.90E−014	−3.205E−014	−4.0E−007
859	−4.7E−007	−3E−006	2.2165	5.877E−016	1.1512E−013	−8.8E−007
860	−1.1E−007	−1E−006	2.2165	6.395E−015	1.4946E−015	−1.3E−007
861	−1E−007	−1E−006	2.2165	1.668E−014	−1.697E−013	−3.1E−007
862	−7.1E−008	−5E−007	2.2165	−1.74E−014	−6.949E−015	−4.0E−008
863	−1.8E−008	−4E−007	2.2165	5.096E−015	9.786E−014	−1.1E−007
864	−3.3E−008	−2E−007	2.2165	8.280E−015	8.7689E−014	−1.2E−008
865	−1.4E−009	−1E−007	2.2165	−9.76E−015	−3.476E−014	−4.0E−008
866	−1.4E−008	−6E−008	2.2165	−1.15E−015	3.0219E−014	−3.4E−009
867	9.41E−010	−4E−008	2.2165	−9.25E−015	−4.845E−014	−1.4E−008
868	−5.7E−009	−2E−008	2.2165	−5.66E−014	1.3973E−013	−8.6E−010
869	7.76E−010	−1E−008	2.2165	2.823E−014	6.8067E−014	−5.2E−009
870	−2.2E−009	−8E−009	2.2165	−3.05E−014	6.9868E−014	−1.7E−010
871	4.19E−010	−5E−009	2.2165	2.356E−014	−2.813E−014	−1.9E−009
872	−8.7E−010	−3E−009	2.2165	−2.65E−014	−6.415E−014	−5.8E−012
873	1.97E−010	−2E−009	2.2165	4.220E−014	8.144E−014	−6.8E−010
874	−3.3E−010	−9E−010	2.2165	−1.99E−014	3.5962E−014	1.905E−011
875	8.72E−011	−6E−010	2.2165	9.540E−015	1.4925E−013	−2.5E−010
876	−1.3E−010	−3E−010	2.2165	2.828E−014	1.4005E−013	1.552E−011
877	3.78E−011	−2E−010	2.2165	−2.03E−014	3.8559E−014	−9.3E−011
878	−4.9E−011	−1E−010	2.2165	2.054E−014	1.3819E−013	8.688E−012
879	1.55E−011	−7E−011	2.2165	−6.48E−015	1.1053E−013	−3.4E−011
880	−1.9E−011	−4E−011	2.2165	−2.51E−014	−1.555E−013	4.003E−012
881	5.80E−012	−2E−011	2.2165	1.907E−014	−7.957E−014	−1.3E−011
882	−6.8E−012	−1E−011	2.2165	−3.49E−014	−2.346E−014	1.551E−012
883	2.37E−012	−8E−012	2.2165	1.425E−014	−1.185E−013	−4.2E−012
884	−2.7E−012	−5E−012	2.2165	−4.00E−014	−1.372E−014	6.898E−013
885	9.25E−013	−3E−012	2.2165	−9.80E−015	1.0322E−013	−1.5E−012
886	−7.4E−013	−1E−012	2.2165	−1.37E−014	3.1525E−013	1.617E−013
887	2.41E−013	−9E−013	2.2165	5.243E−014	2.433E−013	1.152E−013
888	−3.7E−013	−9E−013	2.2165	−2.08E−014	5.7143E−014	8.027E−013
889	5.48E−013	4.7E−014	2.2165	5.723E−014	−2.030E−013	−9.3E−013
890	−1.2E−013	−7E−014	2.2165	−4.18E−014	−2.371E−013	7.114E−013

891	−4.2E−014	−3E−013	2.2165	−2.41E−014	−1.918E−013	4.486E−015
892	−2.0E−013	−1E−013	2.2165	−1.45E−014	−9.421E−014	2.723E−013
893	4.44E−013	5.5E−013	2.2165	−1.41E−014	2.8273E−013	−1.0E−012
894	−6.8E−015	8.8E−014	2.2165	−7.15E−014	4.3134E−013	2.636E−013
895	−1.4E−014	−1E−013	2.2165	7.173E−014	4.0309E−013	3.725E−013
896	−5.3E−013	−4E−013	2.2165	4.862E−014	−6.636E−014	−2.9E−013
897	4.39E−013	4.3E−013	2.2165	−9.58E−014	2.0939E−014	1.105E−013
898	−4.4E−014	−3E−013	2.2165	8.032E−014	4.3623E−014	−1.6E−013
899	−4.0E−013	−4E−013	2.2165	−4.30E−014	−2.257E−013	3.181E−013
900	4.80E−013	1.4E−013	2.2165	5.138E−014	−2.825E−013	−4.7E−013
901	−1.8E−013	2.2E−014	2.2165	−1.31E−015	−2.643E−013	7.353E−014
902	−3.7E−013	−4E−013	2.2165	−3.97E−014	−6.391E−014	−6.8E−013
903	6.99E−014	5.1E−013	2.2165	6.706E−015	−9.570E−014	−1.9E−013
904	−2.0E−013	−3E−013	2.2165	4.807E−014	−2.42E−013	6.866E−013
905	−1.8E−013	−2E−013	2.2165	−2.88E−014	−4.181E−013	−6.3E−013
906	−3.6E−013	−6E−015	2.2165	−7.00E−014	3.5172E−014	−3.1E−013
907	2.95E−013	−1E−013	2.2165	4.671E−014	−2.617E−013	1.415E−013
908	5.61E−013	3.3E−013	2.2165	−1.02E−013	−1.559E−013	−7.4E−014
909	−2.4E−013	−4E−013	2.2165	4.073E−014	−9.711E−014	5.323E−013
910	8.95E−013	3.9E−013	2.2165	−2.11E−014	−2.707E−013	−1.5E−013
911	−8.1E−013	−4E−013	2.2165	5.625E−014	6.7963E−014	−1.6E−012
912	−5.3E−013	−6E−013	2.2165	−6.82E−014	−8.083E−014	1.827E−012
913	5.61E−013	1.7E−013	2.2165	6.83E−016	−5.277E−013	−1.9E−012
914	−7.2E−013	−3E−013	2.2165	−1.42E−014	−2.343E−013	1.151E−012
915	1.37E−013	−5E−013	2.2165	−3.93E−014	8.317E−014	3.458E−013
916	−4.3E−013	−3E−013	2.2165	2.468E−014	−2.773E−013	7.975E−013
917	−1.7E−013	−6E−013	2.2165	−3.35E−014	−3.424E−013	6.391E−013
918	7.15E−013	7.9E−013	2.2165	2.047E−014	−3.301E−013	−3.4E−013
919	−3.4E−013	1.2E−013	2.2165	−5.61E−015	−4.301E−013	−2.0E−013
920	−1.6E−013	−3E−013	2.2165	3.708E−014	−1.620E−013	1.167E−012
921	1.24E−013	4.7E−014	2.2165	−1.63E−014	−3.578E−013	−8.3E−013
922	−4.0E−013	−9E−014	2.2165	8.785E−015	1.256E−013	−4.3E−013
923	−9.7E−014	3.9E−013	2.2165	−6.59E−014	−5.439E−014	−6.7E−014
924	6.97E−013	5.7E−013	2.2165	−1.42E−015	−2.013E−013	−2.6E−013
925	−5.1E−013	−5E−013	2.2165	−7.16E−014	−4.049E−013	4.982E−013
926	−6.9E−013	−4E−013	2.2165	3.482E−014	2.8848E−013	2.357E−013
927	9.46E−013	6.8E−013	2.2165	−4.71E−014	4.1395E−013	−3.8E−013
928	1.09E−014	7.3E−013	2.2165	1.315E−013	7.4885E−013	−7.4E−013
929	5.41E−013	3.8E−013	2.2165	−5.61E−014	4.0112E−013	1.245E−013
930	−6.6E−013	−1E−012	2.2165	−2.09E−015	6.8412E−013	−6.8E−013
931	3.54E−013	−1E−012	2.2165	−2.62E−014	2.6535E−013	1.414E−012
932	−3.1E−013	−2E−012	2.2165	3.473E−014	1.1582E−013	−1E−012
933	2.03E−013	−4E−012	2.2165	−2.87E−014	2.9919E−013	6.270E−013
934	−2.4E−012	−5E−012	2.2165	5.997E−014	−1.576E−014	−4.8E−013
935	3.21E−012	−8E−012	2.2165	−3.35E−014	−2.213E−013	3.906E−012
936	−7.4E−012	−1E−011	2.2165	1.413E−015	−4.258E−013	−1.6E−012
937	5.71E−012	−2E−011	2.2165	−2.70E−014	−7.984E−013	1.295E−011

938	−1.8E−011	−4E−011	2.2165	−2.20E−014	−3.779E−013	−4.3E−012
939	1.62E−011	−6E−011	2.2165	−3.92E−014	2.6329E−013	3.438E−011
940	−4.9E−011	−1E−010	2.2165	−8.67E−014	3.6944E−013	−9.4E−012
941	3.66E−011	−2E−010	2.2165	8.701E−014	3.6663E−013	9.099E−011
942	−1.3E−010	−3E−010	2.2165	6.246E−014	2.7718E−014	−1.4E−011
943	8.66E−011	−6E−010	2.2165	−1.56E−013	1.8895E−013	2.502E−010
944	−3.3E−010	−1E−009	2.2165	1.282E−013	−9.895E−014	−1.8E−011
945	1.98E−010	−2E−009	2.2165	5.502E−014	−2.930E−013	6.844E−010
946	−8.6E−010	−3E−009	2.2165	−3.14E−014	−3.471E−013	6.127E−012
947	4.19E−010	−5E−009	2.2165	2.140E−014	−1.422E−013	1.875E−009
948	−2.2E−009	−8E−009	2.2165	−1.55E−013	2.5807E−013	1.694E−010
949	7.78E−010	−1E−008	2.2165	6.362E−014	4.6778E−013	5.176E−009
950	−5.7E−009	−2E−008	2.2165	2.536E−014	−5.393E−014	8.605E−010
951	9.40E−010	−4E−008	2.2165	3.422E−014	−1.771E−013	1.434E−008
952	−1.4E−008	−6E−008	2.2165	−8.77E−014	−2.920E−013	3.391E−009
953	−1.4E−009	−1E−007	2.2165	1.917E−013	−4.604E−013	3.995E−008
954	−3.3E−008	−2E−007	2.2165	−8.21E−014	−6.125E−013	1.199E−008
955	−1.8E−008	−4E−007	2.2165	9.192E−016	6.8235E−014	1.118E−007
956	−7.1E−008	−5E−007	2.2165	8.375E−014	3.2519E−013	3.992E−008
957	−1E−007	−1E−006	2.2165	−4.31E−014	−2.095E−013	3.140E−007
958	−1.1E−007	−1E−006	2.2165	−4.07E−014	−1.495E−013	1.282E−007
959	−4.7E−007	−3E−006	2.2165	1.617E−014	1.2048E−013	8.841E−007
960	2.70E−008	−3E−006	2.2165	−2.27E−014	4.176E−013	4.032E−007
961	−2.0E−006	−1E−005	2.2165	−9.06E−014	−4.654E−013	2.490E−006
962	1.40E−006	−8E−006	2.2165	6.420E−014	−2.120E−014	1.256E−006
963	−8.4E−006	−3E−005	2.2164	−1.56E−013	−1.697E−013	6.995E−006
964	9.21E−006	−2E−005	2.2164	1.495E−014	4.1905E−014	3.919E−006
965	−3.5E−005	−0.00010	2.2162	−5.58E−014	3.9386E−013	1.949E−005
966	4.72E−005	−3E−005	2.2162	−5.03E−014	1.3514E−013	1.243E−005
967	−0.000142	−0.00033	2.2157	4.277E−014	6.2771E−013	5.343E−005
968	0.00022205	−1E−005	2.2155	2.543E−013	3.3093E−015	4.095E−005
969	−0.000589	−0.00116	2.214	1.511E−014	−5.992E−013	0.00014228
970	0.0010282	0.0003161	2.214	1.629E−013	3.9072E−013	0.00014616
971	−0.002588	−0.00428	2.2089	3.511E−013	8.6822E−013	0.00036013
972	0.0050713	0.002631	2.2097	2.632E−013	−1.396E−013	0.00060514
973	−0.012937	−0.01799	2.1929	1.704E−012	−6.626E−013	0.00084663
974	0.030904	0.018333	2.1937	1.527E−012	−2.733E−012	0.003213
975	−0.079819	−0.09412	2.181	6.617E−012	−8.482E−012	0.0034843
976	0.14614	0.073965	1.7645	1.090E−011	1.5539E−011	0.053813
977	−0.19284	−0.05959	2.4502	−3.82E−011	−1.679E−013	−0.28885
978	2.2088	2.2543	2.5136	−0.0050237	0.64668	−0.14985
979	1.9808	1.9985	2.2709	−0.01887	0.49479	−0.33185
980	1.9985	1.9808	2.2709	−0.01887	0.33185	−0.49479
981	2.2543	2.2088	2.5136	−0.0050237	0.14985	−0.64668
982	0.056051	−0.02616	2.2586	0.0026513	−0.29655	0.09443
983	0.043625	0.080882	2.0092	−0.013804	0.051928	−0.0076766
984	−0.048546	−0.03780	2.1255	−0.0015614	0.012956	−0.012957

985	0.0028793	0.012779	2.1793	−0.0012632	0.0031784	−0.0032786
986	−0.009690	−0.00629	2.1967	−0.0005068	0.0029059	−0.0024157
987	−0.000557	0.0020145	2.2055	−0.0003296	0.00086209	−0.0007619
988	−0.002468	−0.00129	2.2102	−0.0001529	0.00084654	−0.0006389
989	−0.000402	0.0003932	2.2128	−0.0001046	0.00030191	−0.0002349
990	−0.000730	−0.00032	2.2144	−5.16E−005	0.00027006	−0.0001890
991	−0.000180	8.4E−005	2.2153	−3.50E−005	0.00011186	−8.0E−005
992	−0.000230	−9E−005	2.2158	−1.80E−005	8.902E−005	−5.9E−005
993	−7.2E−005	1.8E−005	2.2161	−1.20E−005	4.123E−005	−2.8E−005
994	−7.5E−005	−2E−005	2.2163	−6.30E−006	2.9771E−005	−1.9E−005
995	−2.7E−005	3.5E−006	2.2164	−4.11E−006	1.4981E−005	−1.0E−005
996	−2.5E−005	−7E−006	2.2164	−2.21E−006	1.0035E−005	−6.4E−006
997	−1.0E−005	5.3E−007	2.2165	−1.42E−006	5.3797E−006	−3.5E−006
998	−8.2E−006	−2E−006	2.2165	−7.70E−007	3.4E−006	−2.1E−006
999	−3.7E−006	1.4E−008	2.2165	−4.89E−007	1.9159E−006	−1.2E−006
1000	−2.8E−006	−7E−007	2.2165	−2.68E−007	1.1562E−006	−7.2E−007
1001	−1.3E−006	−4E−008	2.2165	−1.69E−007	6.7855E−007	−4.4E−007
1002	−9.3E−007	−2E−007	2.2165	−9.34E−008	3.9417E−007	−2.5E−007
1003	−4.7E−007	−2E−008	2.2165	−5.87E−008	2.3945E−007	−1.5E−007
1004	−3.2E−007	−7E−008	2.2165	−3.24E−008	1.3463E−007	−8.4E−008
1005	−1.7E−007	−1E−008	2.2165	−2.04E−008	8.4305E−008	−5.4E−008
1006	−1.1E−007	−2E−008	2.2165	−1.13E−008	4.603E−008	−2.9E−008
1007	−5.8E−008	−4E−009	2.2165	−7.07E−009	2.9642E−008	−1.9E−008
1008	−3.7E−008	−8E−009	2.2165	−3.90E−009	1.5746E−008	−9.9E−009
1009	−2.0E−008	−2E−009	2.2165	−2.46E−009	1.0416E−008	−6.6E−009
1010	−1.3E−008	−3E−009	2.2165	−1.35E−009	5.3862E−009	−3.4E−009
1011	−7.2E−009	−6E−010	2.2165	−8.55E−010	3.6594E−009	−2.3E−009
1012	−4.4E−009	−9E−010	2.2165	−4.67E−010	1.8415E−009	−1.2E−009
1013	−2.5E−009	−2E−010	2.2165	−2.98E−010	1.2858E−009	−8.0E−010
1014	−1.5E−009	−3E−010	2.2165	−1.61E−010	6.2904E−010	−4.0E−010
1015	−8.7E−010	−7E−011	2.2165	−1.04E−010	4.5233E−010	−2.8E−010
1016	−5.3E−010	−1E−010	2.2165	−5.57E−011	2.1467E−010	−1.4E−010
1017	−3.0E−010	−2E−011	2.2165	−3.62E−011	1.5918E−010	−9.9E−011
1018	−1.8E−010	−4E−011	2.2165	−1.92E−011	7.2979E−011	−4.7E−011
1019	−1.1E−010	−7E−012	2.2165	−1.26E−011	5.6238E−011	−3.5E−011
1020	−6.3E−011	−1E−011	2.2165	−6.61E−012	2.4755E−011	−1.6E−011
1021	−3.7E−011	−2E−012	2.2165	−4.41E−012	1.9998E−011	−1.2E−011
1022	−2.2E−011	−5E−012	2.2165	−2.26E−012	8.4232E−012	−5.7E−012
1023	−1.3E−011	−7E−013	2.2165	−1.50E−012	6.9371E−012	−4.3E−012
1024	−7.7E−012	−2E−012	2.2165	−8.08E−013	2.5036E−012	−1.9E−012
1025	−4.5E−012	−4E−013	2.2165	−5.29E−013	2.4525E−012	−1.7E−012
1026	−2.6E−012	−5E−013	2.2165	−2.55E−013	9.2892E−013	−6.6E−013
1027	−1.6E−012	−2E−013	2.2165	−2.09E−013	8.3121E−013	−5.2E−013
1028	−1.0E−012	−5E−013	2.2165	−7.87E−014	1.655E−013	−2.2E−013
1029	−6.5E−013	−1E−013	2.2165	−1.04E−013	3.5694E−013	−2.5E−013
1030	−5.6E−013	−3E−013	2.2165	−4.49E−014	−7.392E−014	−4.0E−014
1031	1.04E−013	2.7E−013	2.2165	−8.49E−014	−2.273E−015	−1.9E−013

1032	−2.2E−013	−2E−013	2.2165	3.116E−014	2.4514E−013	−3.6E−014
1033	2.11E−014	4.5E−014	2.2165	4.074E−014	−2.368E−013	−1.4E−013
1034	−8.8E−014	−1E−013	2.2165	−3.82E−014	−2.624E−013	7.183E−014
1035	7.30E−014	2.4E−013	2.2165	−4.85E−015	8.7846E−015	−1.4E−015
1036	1.31E−013	6.7E−014	2.2165	−6.49E−014	1.07E−013	2.112E−013
1037	4.39E−013	4.3E−013	2.2165	−1.26E−013	1.4142E−013	1.523E−013
1038	−6.6E−015	9.1E−014	2.2165	−9.23E−015	3.5518E−013	−8.0E−014
1039	−2.6E−013	−4E−013	2.2165	8.170E−015	−4.712E−013	6.801E−015
1040	−2.2E−013	−6E−014	2.2165	−2.72E−015	−1.663E−013	−3.3E−014
1041	−3.7E−013	−2E−013	2.2165	5.606E−014	6.4806E−014	−4.7E−014
1042	−1.4E−013	−2E−013	2.2165	2.926E−014	−2.537E−013	1.701E−013
1043	2.44E−013	2.3E−013	2.2165	3.977E−014	4.9173E−013	6.844E−014
1044	−3.1E−015	8.4E−014	2.2165	1.220E−014	−1.647E−014	1.932E−013
1045	−3.3E−013	−3E−013	2.2165	2.803E−014	1.6558E−013	3.118E−014
1046	−3.6E−014	1.6E−014	2.2165	−7.10E−015	2.96E−013	9.652E−016
1047	1.19E−013	8.4E−015	2.2165	4.184E−014	−3.736E−013	−1.8E−013
1048	3.95E−014	−1E−013	2.2165	1.030E−014	−4.788E−014	8.236E−014
1049	1.39E−013	1.5E−014	2.2165	−5.41E−014	1.0776E−013	1.811E−013
1050	2.02E−013	2.3E−013	2.2165	−7.24E−014	−2.462E−014	−3.8E−013
1051	−3.3E−014	−7E−014	2.2165	2.135E−014	−4.715E−014	−2.1E−013
1052	−3.1E−013	−1E−013	2.2165	1.759E−014	−2.410E−013	−2.9E−013
1053	3.29E−013	2.3E−013	2.2165	−2.53E−014	−4.716E−014	3.292E−014
1054	−1.1E−013	−9E−014	2.2165	−6.14E−014	−1.352E−013	−1.1E−013
1055	−4.6E−013	−3E−013	2.2165	1.020E−014	1.2583E−013	−1.1E−013
1056	−6.9E−013	−6E−013	2.2165	8.554E−016	3.7714E−013	2.811E−013
1057	−3.7E−013	−7E−014	2.2165	−8.37E−014	1.3793E−013	−2.3E−013
1058	−2.3E−013	−2E−013	2.2165	−5.65E−014	−8.296E−014	−4.1E−014
1059	−8.0E−013	−4E−013	2.2165	2.775E−014	4.1498E−014	−1.4E−013
1060	−1.4E−013	−2E−013	2.2165	−1.53E−015	−6.734E−014	2.339E−013
1061	−1.6E−014	−3E−013	2.2165	−8.57E−014	1.124E−013	1.752E−013
1062	−3.5E−013	−3E−013	2.2165	−1.41E−014	−4.547E−013	−3.7E−013
1063	1.10E−013	−3E−014	2.2165	−3.77E−014	1.0287E−013	−4.5E−013
1064	2.60E−013	1.7E−013	2.2165	5.117E−014	−5.453E−013	−3.4E−013
1065	−6.3E−014	−2E−013	2.2165	−6.62E−014	−5.258E−013	−4.1E−013
1066	−4.2E−013	−3E−013	2.2165	−5.16E−014	−4.359E−013	−3.6E−013
1067	−1.3E−013	4.4E−014	2.2165	−1.18E−013	1.576E−013	−4.2E−013
1068	−6.1E−013	−2E−013	2.2165	5.053E−014	−9.997E−014	−9.0E−014
1069	−6.7E−014	1.1E−013	2.2165	2.579E−014	1.8516E−013	1.537E−013
1070	−2.5E−013	3.7E−014	2.2165	−5.73E−014	9.9524E−014	4.758E−013
1071	−4.1E−013	9.1E−014	2.2165	−1.00E−013	−9.622E−013	4.967E−013
1072	−1.1E−012	−4E−013	2.2165	−5.37E−014	−1.473E−014	5.340E−013
1073	−2E−012	−5E−013	2.2165	−1.10E−013	−7.495E−013	7.133E−013
1074	−2.6E−012	−5E−013	2.2165	−2.54E−013	−6.032E−013	6.691E−013
1075	−5.5E−012	−1E−012	2.2165	−5.68E−013	−2.011E−012	1.370E−012
1076	−8.1E−012	−2E−012	2.2165	−6.31E−013	−3.472E−012	1.806E−012
1077	−1.3E−011	−9E−013	2.2165	−1.57E−012	−6.721E−012	4.552E−012
1078	−2.2E−011	−5E−012	2.2165	−2.25E−012	−9.085E−012	5.424E−012

1079	−3.6E−011	−2E−012	2.2165	−4.47E−012	−1.926E−011	1.231E−011
1080	−6.3E−011	−1E−011	2.2165	−6.63E−012	−2.475E−011	1.607E−011
1081	−1.1E−010	−8E−012	2.2165	−1.26E−011	−5.652E−011	3.491E−011
1082	−1.8E−010	−4E−011	2.2165	−1.92E−011	−7.285E−011	4.702E−011
1083	−3.0E−010	−2E−011	2.2165	−3.62E−011	−1.593E−010	9.927E−011
1084	−5.3E−010	−1E−010	2.2165	−5.59E−011	−2.146E−010	1.368E−010
1085	−8.7E−010	−7E−011	2.2165	−1.04E−010	−4.519E−010	2.814E−010
1086	−1.5E−009	−3E−010	2.2165	−1.61E−010	−6.293E−010	3.986E−010
1087	−2.5E−009	−2E−010	2.2165	−2.98E−010	−1.285E−009	8.037E−010
1088	−4.4E−009	−9E−010	2.2165	−4.67E−010	−1.842E−009	1.164E−009
1089	−7.2E−009	−6E−010	2.2165	−8.55E−010	−3.659E−009	2.295E−009
1090	−1.3E−008	−3E−009	2.2165	−1.35E−009	−5.386E−009	3.392E−009
1091	−2.0E−008	−2E−009	2.2165	−2.46E−009	−1.042E−008	6.560E−009
1092	−3.7E−008	−8E−009	2.2165	−3.90E−009	−1.575E−008	9.887E−009
1093	−5.8E−008	−4E−009	2.2165	−7.07E−009	−2.964E−008	1.874E−008
1094	−1.1E−007	−2E−008	2.2165	−1.13E−008	−4.603E−008	2.883E−008
1095	−1.7E−007	−1E−008	2.2165	−2.04E−008	−8.431E−008	5.355E−008
1096	−3.2E−007	−7E−008	2.2165	−3.24E−008	−1.346E−007	8.417E−008
1097	−4.7E−007	−2E−008	2.2165	−5.87E−008	−2.394E−007	1.529E−007
1098	−9.3E−007	−2E−007	2.2165	−9.34E−008	−3.942E−007	2.463E−007
1099	−1.3E−006	−4E−008	2.2165	−1.69E−007	−6.786E−007	4.359E−007
1100	−2.8E−006	−7E−007	2.2165	−2.68E−007	−1.156E−006	7.233E−007
1101	−3.7E−006	1.4E−008	2.2165	−4.89E−007	−1.916E−006	1.241E−006
1102	−8.2E−006	−2E−006	2.2165	−7.70E−007	−3.4E−006	2.137E−006
1103	−1.0E−005	5.3E−007	2.2165	−1.42E−006	−5.380E−006	3.522E−006
1104	−2.5E−005	−7E−006	2.2164	−2.21E−006	−1.004E−005	6.367E−006
1105	−2.7E−005	3.5E−006	2.2164	−4.11E−006	−1.498E−005	9.971E−006
1106	−7.5E−005	−2E−005	2.2163	−6.30E−006	−2.977E−005	1.923E−005
1107	−7.2E−005	1.8E−005	2.2161	−1.20E−005	−4.123E−005	2.82E−005
1108	−0.000230	−9E−005	2.2158	−1.80E−005	−8.902E−005	5.929E−005
1109	−0.000180	8.4E−005	2.2153	−3.50E−005	−0.00011186	8.021E−005
1110	−0.000730	−0.00032	2.2144	−5.16E−005	−0.00027006	0.00018902
1111	−0.000402	0.0003932	2.2128	−0.0001046	−0.00030191	0.00023494
1112	−0.002468	−0.00129	2.2102	−0.0001529	−0.00084654	0.00063891
1113	−0.000557	0.0020145	2.2055	−0.0003296	−0.00086209	0.00076194
1114	−0.009690	−0.00629	2.1967	−0.0005068	−0.0029059	0.0024157
1115	0.0028793	0.012779	2.1793	−0.0012632	−0.0031784	0.0032786
1116	−0.048546	−0.03780	2.1255	−0.0015614	−0.012956	0.012957
1117	0.043625	0.080882	2.0092	−0.013804	−0.051928	0.0076766
1118	0.056051	−0.02616	2.2586	0.0026513	0.29655	−0.09443
1119	0.14775	0.12476	2.2317	−0.0005966	−0.24358	0.17378
1120	0.024142	0.031499	2.0661	−0.019639	0.030482	−0.017146
1121	−0.033561	−0.02841	2.122	−0.0064352	0.020131	−0.015209
1122	0.0024288	0.005954	2.1767	−0.002803	0.0080758	−0.0077935
1123	−0.007430	−0.00573	2.1914	−0.001504	0.0043194	−0.0043544
1124	0.00033624	0.0012625	2.2053	−0.0009681	0.0022117	−0.0020294
1125	−0.002043	−0.00147	2.2089	−0.0004905	0.0012878	−0.0012679

1126	−7.1E−006	0.0002808	2.2129	−0.0003335	0.00070694	−0.0006382
1127	−0.000611	−0.00041	2.2141	−0.0001699	0.00042664	−0.0004054
1128	−3.9E−005	5.7E−005	2.2153	−0.0001153	0.00023973	−0.0002145
1129	−0.000189	−0.00012	2.2157	−5.96E−005	0.00014592	−0.0001351
1130	−2.4E−005	9.1E−006	2.2161	−3.98E−005	8.2705E−005	−7.4E−005
1131	−6.0E−005	−4E−005	2.2162	−2.09E−005	5.0368E−005	−4.6E−005
1132	−1.1E−005	2.5E−007	2.2164	−1.38E−005	2.8667E−005	−2.6E−005
1133	−1.9E−005	−1E−005	2.2164	−7.32E−006	1.7431E−005	−1.6E−005
1134	−4.6E−006	−7E−007	2.2165	−4.75E−006	9.9483E−006	−8.8E−006
1135	−6.2E−006	−3E−006	2.2165	−2.56E−006	6.0371E−006	−5.4E−006
1136	−1.8E−006	−5E−007	2.2165	−1.64E−006	3.453E−006	−3.1E−006
1137	−2.1E−006	−1E−006	2.2165	−8.95E−007	2.0918E−006	−1.9E−006
1138	−6.9E−007	−2E−007	2.2165	−5.66E−007	1.1984E−006	−1.1E−006
1139	−6.8E−007	−3E−007	2.2165	−3.12E−007	7.2498E−007	−6.4E−007
1140	−2.6E−007	−9E−008	2.2165	−1.96E−007	4.1585E−007	−3.7E−007
1141	−2.3E−007	−1E−007	2.2165	−1.09E−007	2.5134E−007	−2.2E−007
1142	−9.3E−008	−4E−008	2.2165	−6.76E−008	1.4428E−007	−1.3E−007
1143	−7.7E−008	−4E−008	2.2165	−3.79E−008	8.7161E−008	−7.7E−008
1144	−3.3E−008	−1E−008	2.2165	−2.34E−008	5.0048E−008	−4.4E−008
1145	−2.6E−008	−1E−008	2.2165	−1.32E−008	3.0235E−008	−2.7E−008
1146	−1.2E−008	−5E−009	2.2165	−8.10E−009	1.7357E−008	−1.5E−008
1147	−8.9E−009	−4E−009	2.2165	−4.59E−009	1.0491E−008	−9.3E−009
1148	−4.2E−009	−2E−009	2.2165	−2.81E−009	6.0188E−009	−5.3E−009
1149	−3.0E−009	−1E−009	2.2165	−1.60E−009	3.6414E−009	−3.2E−009
1150	−1.5E−009	−7E−010	2.2165	−9.72E−010	2.0865E−009	−1.9E−009
1151	−1.0E−009	−4E−010	2.2165	−5.54E−010	1.2641E−009	−1.1E−009
1152	−5.2E−010	−3E−010	2.2165	−3.37E−010	7.2328E−010	−6.4E−010
1153	−3.6E−010	−1E−010	2.2165	−1.93E−010	4.3905E−010	−3.9E−010
1154	−1.8E−010	−9E−011	2.2165	−1.17E−010	2.5056E−010	−2.2E−010
1155	−1.2E−010	−5E−011	2.2165	−6.69E−011	1.5252E−010	−1.4E−010
1156	−6.4E−011	−3E−011	2.2165	−4.05E−011	8.6886E−011	−7.7E−011
1157	−4.2E−011	−1E−011	2.2165	−2.32E−011	5.2955E−011	−4.7E−011
1158	−2.2E−011	−1E−011	2.2165	−1.41E−011	3.0095E−011	−2.7E−011
1159	−1.5E−011	−5E−012	2.2165	−8.00E−012	1.8426E−011	−1.6E−011
1160	−7.9E−012	−5E−012	2.2165	−4.89E−012	1.0406E−011	−9.3E−012
1161	−5.2E−012	−2E−012	2.2165	−2.77E−012	6.28E−012	−5.7E−012
1162	−2.7E−012	−2E−012	2.2165	−1.69E−012	3.5941E−012	−3.2E−012
1163	−1.8E−012	−5E−013	2.2165	−9.24E−013	2.1691E−012	−2.2E−012
1164	−1.0E−012	−6E−013	2.2165	−5.37E−013	1.3464E−012	−1.0E−012
1165	−6.3E−013	−2E−013	2.2165	−3.72E−013	6.7358E−013	−5.6E−013
1166	−2.0E−013	−6E−014	2.2165	−1.66E−013	5.2096E−013	−1.8E−013
1167	−3.9E−013	−3E−013	2.2165	−1.61E−013	3.6073E−013	−2.8E−013
1168	−3.5E−014	4.5E−014	2.2165	−7.85E−014	2.8352E−014	8.316E−014
1169	−2.5E−013	−1E−013	2.2165	−5.47E−014	−1.434E−014	−1.7E−013
1170	−2.5E−013	−3E−013	2.2165	3.378E−014	7.5565E−014	−2.7E−013
1171	−3.2E−013	−4E−013	2.2165	−2.64E−015	−3.884E−014	−2.4E−013
1172	−2.2E−013	−2E−013	2.2165	3.204E−014	3.1926E−014	−6.8E−014

1173	−5.0E−013	−5E−013	2.2165	8.174E−016	−8.078E−014	8.947E−014
1174	−9.8E−014	−1E−013	2.2165	−4.00E−014	6.9885E−014	1.150E−013
1175	−1.4E−013	−2E−013	2.2165	8.385E−015	3.8004E−014	2.613E−013
1176	−5.6E−014	−2E−013	2.2165	−1.07E−013	−1.043E−013	−2.3E−013
1177	3.40E−013	2.9E−013	2.2165	3.710E−014	−6.106E−014	−6.8E−014
1178	−2.1E−013	−4E−014	2.2165	−4.21E−015	−6.411E−014	2.837E−013
1179	−4.9E−014	−1E−013	2.2165	−7.82E−014	−3.253E−014	−9.7E−014
1180	−3.7E−013	−4E−013	2.2165	−2.46E−015	−1.587E−013	2.802E−013
1181	−5.7E−014	−9E−015	2.2165	−1.73E−014	9.6997E−014	−1.1E−013
1182	−2.2E−013	−1E−013	2.2165	−3.50E−014	3.054E−013	6.749E−014
1183	−2.4E−013	−2E−013	2.2165	1.052E−014	−6.576E−014	3.365E−013
1184	−6.1E−015	−1E−013	2.2165	−3.59E−015	1.5662E−013	6.477E−014
1185	−2.5E−013	−3E−013	2.2165	2.73E−014	−7.291E−014	−1.8E−013
1186	5.35E−014	9.3E−014	2.2165	5.020E−014	9.4875E−015	2.954E−013
1187	−1.4E−013	−2E−013	2.2165	−8.62E−014	−1.421E−013	4.851E−014
1188	−2.5E−013	−3E−013	2.2165	7.664E−014	−2.120E−013	−2.1E−013
1189	−4.8E−013	−4E−013	2.2165	5.002E−014	−1.225E−013	−2.1E−013
1190	3.01E−013	1.7E−013	2.2165	2.2E−014	4.2846E−014	1.284E−013
1191	−1.1E−013	−4E−014	2.2165	−3.37E−014	−1.150E−013	−1.7E−013
1192	7.66E−014	−2E−013	2.2165	1.257E−014	8.8407E−014	−9.3E−014
1193	3.05E−013	4.5E−013	2.2165	−7.51E−014	−1.159E−013	−4.3E−013
1194	2.19E−013	1.5E−013	2.2165	−1.90E−014	1.331E−014	−8.6E−014
1195	3.99E−014	1.4E−013	2.2165	4.370E−014	5.9154E−014	6.727E−014
1196	−1.2E−013	−2E−014	2.2165	−2.57E−014	1.4884E−013	8.781E−014
1197	9.34E−014	−9E−014	2.2165	−4.79E−014	2.7894E−013	1.223E−013
1198	1.63E−013	−5E−014	2.2165	−7.80E−015	−2.117E−014	1.767E−013
1199	1.10E−013	6.3E−014	2.2165	5.635E−014	−2.271E−013	−5.3E−013
1200	−7.7E−014	−2E−013	2.2165	−2.51E−015	−5.438E−014	−1.0E−013
1201	1.03E−013	1.4E−013	2.2165	1.995E−014	−2.326E−013	−4.7E−013
1202	−1.0E−013	−2E−013	2.2165	2.693E−014	−4.371E−014	−5.6E−013
1203	−1.3E−013	−7E−014	2.2165	6.225E−014	−3.220E−013	−3.8E−013
1204	−4.5E−013	−3E−013	2.2165	−1.16E−014	−1.301E−013	1.395E−013
1205	−2.6E−013	−5E−014	2.2165	4.017E−014	−1.176E−013	5.114E−013
1206	−3.6E−013	−3E−013	2.2165	−3.25E−014	−5.316E−014	5.823E−013
1207	−6.4E−013	−6E−013	2.2165	−1.72E−013	2.1514E−013	3.617E−013
1208	−5.3E−014	2.4E−014	2.2165	−3.00E−013	−1.583E−013	5.166E−013
1209	−4.4E−013	6.7E−015	2.2165	−3.74E−013	−5.809E−013	6.657E−013
1210	−1.0E−012	−8E−013	2.2165	−5.80E−013	−9.621E−013	1.685E−012
1211	−1.2E−012	−3E−014	2.2165	−9.15E−013	−2.671E−012	2.014E−012
1212	−2.6E−012	−2E−012	2.2165	−1.75E−012	−3.442E−012	3.394E−012
1213	−4.8E−012	−1E−012	2.2165	−2.78E−012	−6.316E−012	5.335E−012
1214	−7.6E−012	−4E−012	2.2165	−4.85E−012	−1.021E−011	8.998E−012
1215	−1.5E−011	−5E−012	2.2165	−8.00E−012	−1.822E−011	1.618E−011
1216	−2.2E−011	−1E−011	2.2165	−1.40E−011	−3.003E−011	2.701E−011
1217	−4.3E−011	−1E−011	2.2165	−2.32E−011	−5.328E−011	4.682E−011
1218	−6.4E−011	−3E−011	2.2165	−4.05E−011	−8.692E−011	7.689E−011
1219	−1.2E−010	−4E−011	2.2165	−6.69E−011	−1.525E−010	1.352E−010

1220	−1.8E−010	−9E−011	2.2165	−1.17E−010	−2.506E−010	2.227E−010
1221	−3.6E−010	−1E−010	2.2165	−1.93E−010	−4.387E−010	3.887E−010
1222	−5.2E−010	−3E−010	2.2165	−3.37E−010	−7.233E−010	6.421E−010
1223	−1.0E−009	−4E−010	2.2165	−5.54E−010	−1.264E−009	1.119E−009
1224	−1.5E−009	−7E−010	2.2165	−9.72E−010	−2.087E−009	1.853E−009
1225	−3.0E−009	−1E−009	2.2165	−1.60E−009	−3.641E−009	3.223E−009
1226	−4.2E−009	−2E−009	2.2165	−2.81E−009	−6.019E−009	5.346E−009
1227	−8.9E−009	−4E−009	2.2165	−4.59E−009	−1.049E−008	9.286E−009
1228	−1.2E−008	−5E−009	2.2165	−8.10E−009	−1.736E−008	1.542E−008
1229	−2.6E−008	−1E−008	2.2165	−1.32E−008	−3.023E−008	2.677E−008
1230	−3.3E−008	−1E−008	2.2165	−2.34E−008	−5.005E−008	4.449E−008
1231	−7.7E−008	−4E−008	2.2165	−3.79E−008	−8.716E−008	7.719E−008
1232	−9.3E−008	−4E−008	2.2165	−6.76E−008	−1.443E−007	1.283E−007
1233	−2.3E−007	−1E−007	2.2165	−1.09E−007	−2.513E−007	2.228E−007
1234	−2.6E−007	−9E−008	2.2165	−1.96E−007	−4.159E−007	3.698E−007
1235	−6.8E−007	−3E−007	2.2165	−3.12E−007	−7.250E−007	6.436E−007
1236	−6.9E−007	−2E−007	2.2165	−5.66E−007	−1.198E−006	1.066E−006
1237	−2.1E−006	−1E−006	2.2165	−8.95E−007	−2.092E−006	1.862E−006
1238	−1.8E−006	−5E−007	2.2165	−1.64E−006	−3.453E−006	3.070E−006
1239	−6.2E−006	−3E−006	2.2165	−2.56E−006	−6.037E−006	5.396E−006
1240	−4.6E−006	−7E−007	2.2165	−4.75E−006	−9.948E−006	8.846E−006
1241	−1.9E−005	−1E−005	2.2164	−7.32E−006	−1.743E−005	1.569E−005
1242	−1.1E−005	2.5E−007	2.2164	−1.38E−005	−2.867E−005	2.55E−005
1243	−6.0E−005	−4E−005	2.2162	−2.09E−005	−5.037E−005	4.583E−005
1244	−2.4E−005	9.1E−006	2.2161	−3.98E−005	−8.271E−005	7.369E−005
1245	−0.000189	−0.00012	2.2157	−5.96E−005	−0.00014592	0.00013508
1246	−3.9E−005	5.7E−005	2.2153	−0.0001153	−0.00023973	0.00021449
1247	−0.000611	−0.00041	2.2141	−0.0001699	−0.00042664	0.00040537
1248	−7.1E−006	0.0002808	2.2129	−0.0003335	−0.00070694	0.0006382
1249	−0.002043	−0.00147	2.2089	−0.0004905	−0.0012878	0.0012679
1250	0.00033624	0.0012625	2.2053	−0.0009681	−0.0022117	0.0020294
1251	−0.007430	−0.00573	2.1914	−0.001504	−0.0043194	0.0043544
1252	0.0024288	0.005954	2.1767	−0.002803	−0.0080758	0.0077935
1253	−0.033561	−0.02841	2.122	−0.0064352	−0.020131	0.015209
1254	0.024142	0.031499	2.0661	−0.019639	−0.030482	0.017146
1255	0.14775	0.12476	2.2317	−0.0005966	0.24358	−0.17378
1256	0.12476	0.14775	2.2317	−0.0005966	−0.17378	0.24358
1257	0.031499	0.024142	2.0661	−0.019639	0.017146	−0.030482
1258	−0.028413	−0.03356	2.122	−0.0064352	0.015209	−0.020131
1259	0.005954	0.0024288	2.1767	−0.002803	0.0077935	−0.0080758
1260	−0.005728	−0.00743	2.1914	−0.001504	0.0043544	−0.0043194
1261	0.0012625	0.0003362	2.2053	−0.0009681	0.0020294	−0.0022117
1262	−0.001467	−0.00204	2.2089	−0.0004905	0.0012679	−0.0012878
1263	0.00028079	−7E−006	2.2129	−0.0003335	0.0006382	−0.0007069
1264	−0.000413	−0.00061	2.2141	−0.0001699	0.00040537	−0.0004266
1265	5.74E−005	−4E−005	2.2153	−0.0001153	0.00021449	−0.0002397
1266	−0.000121	−0.00019	2.2157	−5.96E−005	0.00013508	−0.0001459

1267	9.13E−006	−2E−005	2.2161	−3.98E−005	7.3687E−005	−8.3E−005
1268	−3.6E−005	−6E−005	2.2162	−2.09E−005	4.5831E−005	−5.0E−005
1269	2.47E−007	−1E−005	2.2164	−1.38E−005	2.55E−005	−2.9E−005
1270	−1.1E−005	−2E−005	2.2164	−7.32E−006	1.5688E−005	−1.7E−005
1271	−7.1E−007	−5E−006	2.2165	−4.75E−006	8.8455E−006	−9.9E−006
1272	−3.4E−006	−6E−006	2.2165	−2.56E−006	5.3963E−006	−6.0E−006
1273	−4.6E−007	−2E−006	2.2165	−1.64E−006	3.0702E−006	−3.5E−006
1274	−1.1E−006	−2E−006	2.2165	−8.95E−007	1.8618E−006	−2.1E−006
1275	−2.2E−007	−7E−007	2.2165	−5.66E−007	1.0656E−006	−1.2E−006
1276	−3.4E−007	−7E−007	2.2165	−3.12E−007	6.4362E−007	−7.2E−007
1277	−9.3E−008	−3E−007	2.2165	−1.96E−007	3.6978E−007	−4.2E−007
1278	−1.1E−007	−2E−007	2.2165	−1.09E−007	2.2279E−007	−2.5E−007
1279	−3.7E−008	−9E−008	2.2165	−6.76E−008	1.2828E−007	−1.4E−007
1280	−3.5E−008	−8E−008	2.2165	−3.79E−008	7.7195E−008	−8.7E−008
1281	−1.4E−008	−3E−008	2.2165	−2.34E−008	4.4488E−008	−5.0E−008
1282	−1.2E−008	−3E−008	2.2165	−1.32E−008	2.6766E−008	−3.0E−008
1283	−5.3E−009	−1E−008	2.2165	−8.10E−009	1.5424E−008	−1.7E−008
1284	−3.8E−009	−9E−009	2.2165	−4.59E−009	9.2861E−009	−1.0E−008
1285	−2.0E−009	−4E−009	2.2165	−2.81E−009	5.3465E−009	−6.0E−009
1286	−1.2E−009	−3E−009	2.2165	−1.60E−009	3.2232E−009	−3.6E−009
1287	−7.2E−010	−1E−009	2.2165	−9.72E−010	1.8529E−009	−2.1E−009
1288	−4.1E−010	−1E−009	2.2165	−5.54E−010	1.1191E−009	−1.3E−009
1289	−2.6E−010	−5E−010	2.2165	−3.37E−010	6.4196E−010	−7.2E−010
1290	−1.4E−010	−4E−010	2.2165	−1.93E−010	3.8869E−010	−4.4E−010
1291	−9.4E−011	−2E−010	2.2165	−1.17E−010	2.2239E−010	−2.5E−010
1292	−4.5E−011	−1E−010	2.2165	−6.69E−011	1.3503E−010	−1.5E−010
1293	−3.4E−011	−6E−011	2.2165	−4.05E−011	7.7101E−011	−8.7E−011
1294	−1.5E−011	−4E−011	2.2165	−2.32E−011	4.6903E−011	−5.3E−011
1295	−1.2E−011	−2E−011	2.2165	−1.41E−011	2.6739E−011	−3.0E−011
1296	−5.1E−012	−1E−011	2.2165	−8.05E−012	1.6233E−011	−1.8E−011
1297	−4.3E−012	−8E−012	2.2165	−4.88E−012	9.0418E−012	−1.1E−011
1298	−1.6E−012	−5E−012	2.2165	−2.78E−012	5.7449E−012	−6.2E−012
1299	−1.6E−012	−3E−012	2.2165	−1.67E−012	3.0832E−012	−3.7E−012
1300	−6.9E−013	−2E−012	2.2165	−9.85E−013	1.8814E−012	−2.2E−012
1301	−8.2E−013	−1E−012	2.2165	−5.97E−013	1.0734E−012	−1.2E−012
1302	−2.0E−013	−6E−013	2.2165	−3.80E−013	9.0687E−013	−7.5E−013
1303	−1.3E−013	−3E−013	2.2165	−1.90E−013	4.7033E−013	−5.6E−013
1304	−1.0E−013	−3E−013	2.2165	−1.52E−013	3.32E−013	−2.7E−013
1305	−2.2E−013	−3E−013	2.2165	−4.71E−014	2.7676E−014	−7.6E−014
1306	−3.0E−013	−3E−013	2.2165	−5.49E−014	−9.649E−014	−2.3E−013
1307	1.52E−013	1.7E−014	2.2165	−8.11E−014	1.0561E−015	9.110E−014
1308	−2.4E−013	−2E−013	2.2165	−5.10E−014	−1.695E−015	−2.6E−013
1309	−1.0E−013	−1E−013	2.2165	−1.74E−014	−1.310E−013	−1.6E−013
1310	−3.5E−013	−3E−013	2.2165	1.435E−014	−5.811E−014	−2.6E−013
1311	−1E−013	2.7E−014	2.2165	7.05E−014	2.8065E−014	−6.1E−015
1312	3.37E−013	2.7E−013	2.2165	−9.34E−015	1.0452E−013	5.262E−014
1313	−3.8E−013	−4E−013	2.2165	1.546E−014	1.7613E−013	4.250E−014

1314	−3.2E−014	−1E−013	2.2165	6.571E−014	2.7035E−014	−9.4E−014
1315	1.37E−013	1.7E−013	2.2165	7.741E−015	−1.300E−014	−4.0E−014
1316	−2.3E−013	−2E−013	2.2165	−8.06E−014	2.3335E−013	1.834E−013
1317	−1.9E−013	−4E−013	2.2165	−2.74E−014	8.5492E−014	8.255E−015
1318	1.16E−013	1.3E−013	2.2165	2.653E−014	7.852E−014	−2.0E−013
1319	−1.1E−013	−4E−014	2.2165	−2.32E−014	1.8101E−014	2.394E−014
1320	−3.4E−013	−4E−013	2.2165	−3.54E−014	1.7577E−013	−1.1E−013
1321	3.05E−013	2.1E−013	2.2165	−8.22E−014	−1.037E−013	5.124E−014
1322	1.54E−013	8.8E−014	2.2165	−2.93E−014	−3.231E−013	−3.1E−013
1323	6.81E−014	8.9E−014	2.2165	−4.76E−014	6.8448E−014	3.003E−014
1324	3.12E−014	1.1E−013	2.2165	−7.52E−014	−9.226E−014	−2.2E−014
1325	3.62E−013	1.7E−013	2.2165	9.022E−014	−8.770E−014	−3.3E−013
1326	−4.2E−013	−1E−013	2.2165	1.027E−013	−6.937E−015	8.931E−014
1327	−3.4E−013	−4E−013	2.2165	6.937E−015	−2.208E−013	−8.5E−014
1328	−3.1E−013	−4E−013	2.2165	1.239E−013	−2.780E−013	1.321E−013
1329	1.25E−013	7.3E−014	2.2165	−5.31E−014	−2.748E−013	1.768E−013
1330	6.59E−014	3.7E−014	2.2165	2.725E−014	−2.450E−013	−3.8E−013
1331	−4.3E−013	−2E−013	2.2165	5.631E−014	−1.288E−013	1.388E−013
1332	9.94E−014	−8E−015	2.2165	5.897E−014	−3.818E−013	−2.3E−013
1333	−4.0E−013	−2E−013	2.2165	−4.42E−014	−1.182E−013	2.418E−013
1334	−3.8E−013	−4E−013	2.2165	3.579E−014	5.6869E−014	1.205E−013
1335	−2.3E−013	−3E−013	2.2165	−2.90E−014	4.2994E−014	2.823E−013
1336	1.69E−013	7E−014	2.2165	5.781E−015	−2.174E−013	3.911E−014
1337	−2.5E−014	3.4E−014	2.2165	5.841E−014	5.6595E−014	1.529E−015
1338	4.29E−014	1.5E−013	2.2165	5.293E−014	−4.569E−014	1.089E−013
1339	−4.5E−014	−1E−013	2.2165	4.880E−014	−1.910E−013	3.784E−013
1340	−6.9E−014	4.6E−014	2.2165	1.624E−015	−1.309E−013	−4.9E−013
1341	−6.4E−013	−5E−013	2.2165	−1.38E−014	1.7363E−013	3.211E−013
1342	7.53E−014	2.5E−014	2.2165	1.791E−014	7.5542E−014	2.586E−013
1343	3.04E−013	1.7E−013	2.2165	−3.55E−014	−1.588E−013	4.681E−013
1344	−4.0E−013	−4E−013	2.2165	−1.75E−013	8.5943E−014	5.371E−013
1345	9.71E−014	−2E−013	2.2165	−2.02E−013	−4.895E−013	3.130E−013
1346	−8.2E−014	−6E−013	2.2165	−3.80E−013	−1.799E−013	9.406E−013
1347	−1.0E−012	−1E−012	2.2165	−5.68E−013	−1.124E−012	1.766E−012
1348	−5.3E−013	−2E−012	2.2165	−9.12E−013	−2.108E−012	1.769E−012
1349	−2.0E−012	−3E−012	2.2165	−1.65E−012	−3.311E−012	3.756E−012
1350	−1.2E−012	−5E−012	2.2165	−2.84E−012	−5.697E−012	6.481E−012
1351	−4.6E−012	−8E−012	2.2165	−4.83E−012	−9.465E−012	1.030E−011
1352	−5.0E−012	−1E−011	2.2165	−8.06E−012	−1.657E−011	1.858E−011
1353	−1.2E−011	−2E−011	2.2165	−1.40E−011	−2.681E−011	3.003E−011
1354	−1.5E−011	−4E−011	2.2165	−2.32E−011	−4.687E−011	5.315E−011
1355	−3.4E−011	−6E−011	2.2165	−4.06E−011	−7.706E−011	8.665E−011
1356	−4.6E−011	−1E−010	2.2165	−6.69E−011	−1.354E−010	1.523E−010
1357	−9.3E−011	−2E−010	2.2165	−1.17E−010	−2.220E−010	2.509E−010
1358	−1.4E−010	−4E−010	2.2165	−1.93E−010	−3.886E−010	4.393E−010
1359	−2.6E−010	−5E−010	2.2165	−3.37E−010	−6.419E−010	7.235E−010
1360	−4.1E−010	−1E−009	2.2165	−5.54E−010	−1.119E−009	1.264E−009

1361	−7.2E−010	−1E−009	2.2165	−9.72E−010	−1.853E−009	2.087E−009
1362	−1.2E−009	−3E−009	2.2165	−1.60E−009	−3.223E−009	3.641E−009
1363	−2.0E−009	−4E−009	2.2165	−2.81E−009	−5.347E−009	6.019E−009
1364	−3.8E−009	−9E−009	2.2165	−4.59E−009	−9.286E−009	1.049E−008
1365	−5.3E−009	−1E−008	2.2165	−8.10E−009	−1.543E−008	1.736E−008
1366	−1.2E−008	−3E−008	2.2165	−1.32E−008	−2.677E−008	3.023E−008
1367	−1.4E−008	−3E−008	2.2165	−2.34E−008	−4.449E−008	5.005E−008
1368	−3.5E−008	−8E−008	2.2165	−3.79E−008	−7.719E−008	8.716E−008
1369	−3.7E−008	−9E−008	2.2165	−6.76E−008	−1.283E−007	1.443E−007
1370	−1.1E−007	−2E−007	2.2165	−1.09E−007	−2.228E−007	2.513E−007
1371	−9.3E−008	−3E−007	2.2165	−1.96E−007	−3.698E−007	4.159E−007
1372	−3.4E−007	−7E−007	2.2165	−3.12E−007	−6.436E−007	7.250E−007
1373	−2.2E−007	−7E−007	2.2165	−5.66E−007	−1.066E−006	1.198E−006
1374	−1.1E−006	−2E−006	2.2165	−8.95E−007	−1.862E−006	2.092E−006
1375	−4.6E−007	−2E−006	2.2165	−1.64E−006	−3.070E−006	3.453E−006
1376	−3.4E−006	−6E−006	2.2165	−2.56E−006	−5.396E−006	6.037E−006
1377	−7.1E−007	−5E−006	2.2165	−4.75E−006	−8.846E−006	9.948E−006
1378	−1.1E−005	−2E−005	2.2164	−7.32E−006	−1.569E−005	1.743E−005
1379	2.47E−007	−1E−005	2.2164	−1.38E−005	−2.55E−005	2.867E−005
1380	−3.6E−005	−6E−005	2.2162	−2.09E−005	−4.583E−005	5.037E−005
1381	9.13E−006	−2E−005	2.2161	−3.98E−005	−7.369E−005	8.271E−005
1382	−0.000121	−0.00019	2.2157	−5.96E−005	−0.00013508	0.00014592
1383	5.74E−005	−4E−005	2.2153	−0.0001153	−0.00021449	0.00023973
1384	−0.000413	−0.00061	2.2141	−0.0001699	−0.00040537	0.00042664
1385	0.00028079	−7E−006	2.2129	−0.0003335	−0.0006382	0.00070694
1386	−0.001467	−0.00204	2.2089	−0.0004905	−0.0012679	0.0012878
1387	0.0012625	0.0003362	2.2053	−0.0009681	−0.0020294	0.0022117
1388	−0.005728	−0.00743	2.1914	−0.001504	−0.0043544	0.0043194
1389	0.005954	0.0024288	2.1767	−0.002803	−0.0077935	0.0080758
1390	−0.028413	−0.03356	2.122	−0.0064352	−0.015209	0.020131
1391	0.031499	0.024142	2.0661	−0.019639	−0.017146	0.030482
1392	0.12476	0.14775	2.2317	−0.0005966	0.17378	−0.24358
1393	−0.026163	0.056051	2.2586	0.0026513	−0.09443	0.29655
1394	0.080882	0.043625	2.0092	−0.013804	0.0076766	−0.051928
1395	−0.037796	−0.04855	2.1255	−0.0015614	0.012957	−0.012956
1396	0.012779	0.0028793	2.1793	−0.0012632	0.0032786	−0.0031784
1397	−0.006291	−0.00969	2.1967	−0.0005068	0.0024157	−0.0029059
1398	0.0020145	−0.00056	2.2055	−0.0003296	0.00076194	−0.0008621
1399	−0.001295	−0.00247	2.2102	−0.0001529	0.00063891	−0.0008465
1400	0.00039323	−0.00040	2.2128	−0.0001046	0.00023494	−0.0003019
1401	−0.000321	−0.00073	2.2144	−5.16E−005	0.00018902	−0.0002701
1402	8.39E−005	−0.00018	2.2153	−3.50E−005	8.0208E−005	−0.0001119
1403	−8.7E−005	−0.00023	2.2158	−1.80E−005	5.9293E−005	−8.9E−005
1404	1.78E−005	−7E−005	2.2161	−1.20E−005	2.82E−005	−4.1E−005
1405	−2.5E−005	−7E−005	2.2163	−6.30E−006	1.9231E−005	−3.0E−005
1406	3.46E−006	−3E−005	2.2164	−4.11E−006	9.9711E−006	−1.5E−005
1407	−7.3E−006	−2E−005	2.2164	−2.21E−006	6.3674E−006	−1.0E−005

1408	5.28E−007	−1E−005	2.2165	−1.42E−006	3.5215E−006	−5.4E−006
1409	−2.2E−006	−8E−006	2.2165	−7.70E−007	2.1367E−006	−3.4E−006
1410	1.44E−008	−4E−006	2.2165	−4.89E−007	1.2405E−006	−1.9E−006
1411	−6.8E−007	−3E−006	2.2165	−2.68E−007	7.2333E−007	−1.2E−006
1412	−3.7E−008	−1E−006	2.2165	−1.69E−007	4.3588E−007	−6.8E−007
1413	−2.2E−007	−9E−007	2.2165	−9.34E−008	2.4629E−007	−3.9E−007
1414	−2.3E−008	−5E−007	2.2165	−5.87E−008	1.5288E−007	−2.4E−007
1415	−7.0E−008	−3E−007	2.2165	−3.24E−008	8.4171E−008	−1.3E−007
1416	−1.1E−008	−2E−007	2.2165	−2.04E−008	5.355E−008	−8.4E−008
1417	−2.3E−008	−1E−007	2.2165	−1.13E−008	2.8831E−008	−4.6E−008
1418	−4.3E−009	−6E−008	2.2165	−7.07E−009	1.8744E−008	−3.0E−008
1419	−7.7E−009	−4E−008	2.2165	−3.90E−009	9.887E−009	−1.6E−008
1420	−1.6E−009	−2E−008	2.2165	−2.46E−009	6.5594E−009	−1.0E−008
1421	−2.6E−009	−1E−008	2.2165	−1.35E−009	3.3923E−009	−5.4E−009
1422	−5.8E−010	−7E−009	2.2165	−8.55E−010	2.2957E−009	−3.7E−009
1423	−9.0E−010	−4E−009	2.2165	−4.67E−010	1.1638E−009	−1.8E−009
1424	−2E−010	−3E−009	2.2165	−2.98E−010	8.0395E−010	−1.3E−009
1425	−3.1E−010	−2E−009	2.2165	−1.61E−010	3.9884E−010	−6.3E−010
1426	−6.8E−011	−9E−010	2.2165	−1.04E−010	2.8181E−010	−4.5E−010
1427	−1.1E−010	−5E−010	2.2165	−5.57E−011	1.3658E−010	−2.1E−010
1428	−2.3E−011	−3E−010	2.2165	−3.62E−011	9.8871E−011	−1.6E−010
1429	−3.9E−011	−2E−010	2.2165	−1.92E−011	4.6502E−011	−7.3E−011
1430	−7.0E−012	−1E−010	2.2165	−1.26E−011	3.4697E−011	−5.6E−011
1431	−1.4E−011	−6E−011	2.2165	−6.60E−012	1.6026E−011	−2.5E−011
1432	−2.1E−012	−4E−011	2.2165	−4.44E−012	1.2184E−011	−2.0E−011
1433	−5.1E−012	−2E−011	2.2165	−2.30E−012	5.4391E−012	−8.6E−012
1434	−6.5E−013	−1E−011	2.2165	−1.54E−012	4.2276E−012	−7.1E−012
1435	−1.8E−012	−8E−012	2.2165	−7.74E−013	1.8258E−012	−3.0E−012
1436	−1.3E−013	−4E−012	2.2165	−5.31E−013	1.3185E−012	−2.1E−012
1437	−6.3E−013	−3E−012	2.2165	−2.57E−013	5.6372E−013	−1.0E−012
1438	−2.0E−013	−2E−012	2.2165	−1.96E−013	4.759E−013	−6.5E−013
1439	−4.4E−013	−1E−012	2.2165	−7.40E−014	2.3622E−013	−1.1E−013
1440	1.14E−013	−5E−013	2.2165	−6.05E−014	1.976E−013	−5.1E−013
1441	−1.7E−013	−3E−013	2.2165	−3.24E−014	−1.349E−013	−1.8E−013
1442	2.90E−013	−5E−014	2.2165	−3.26E−015	−2.435E−014	−1.6E−013
1443	−2.4E−013	−3E−013	2.2165	5.192E−015	−1.957E−013	1.643E−013
1444	3.88E−014	−1E−013	2.2165	−3.10E−014	7.9287E−014	1.133E−013
1445	7.14E−014	−6E−015	2.2165	−3.69E−014	−7.024E−014	7.227E−014
1446	−1.1E−013	−2E−013	2.2165	−2.91E−014	2.1252E−013	−2.7E−013
1447	−2.6E−013	−2E−013	2.2165	2.153E−014	9.6727E−014	2.497E−013
1448	1.53E−013	1.5E−013	2.2165	3.025E−014	3.5625E−014	−1.1E−013
1449	1.13E−013	2.0E−013	2.2165	−3.34E−015	2.9931E−014	3.520E−013
1450	−3.4E−014	−9E−014	2.2165	9.102E−015	−1.700E−013	1.869E−013
1451	1.46E−013	5.3E−014	2.2165	−6.50E−014	1.1109E−014	5.544E−014
1452	−2.0E−013	−1E−013	2.2165	−2.98E−015	−1.930E−013	6.423E−014
1453	−7.0E−014	−4E−014	2.2165	−3.85E−014	−6.081E−014	3.348E−013
1454	1.15E−013	−2E−015	2.2165	−2.64E−014	1.0135E−013	4.415E−014

1455	3.60E−013	2.4E−013	2.2165	1.603E−014	2.5377E−013	5.194E−014
1456	−4.6E−014	−4E−015	2.2165	8.868E−014	7.8715E−014	−3.0E−013
1457	−1.7E−013	−4E−013	2.2165	1.615E−014	−2.363E−013	−8.8E−014
1458	1.64E−013	−6E−014	2.2165	−5.37E−014	−1.772E−013	2.736E−014
1459	1.08E−013	−7E−014	2.2165	3.869E−014	−5.030E−013	−3.7E−013
1460	−3.1E−014	−2E−013	2.2165	−1.11E−013	−1.559E−013	1.376E−013
1461	−2.1E−013	−2E−013	2.2165	−6.77E−014	−1.523E−013	−3.9E−013
1462	−3.5E−014	9.6E−014	2.2165	1.434E−014	9.3454E−014	5.594E−013
1463	−1.5E−013	−7E−014	2.2165	3.050E−014	−3.362E−013	1.779E−013
1464	4.91E−014	9.6E−014	2.2165	−5.76E−014	9.6033E−015	−1.5E−014
1465	3.00E−013	8.1E−014	2.2165	−8.37E−015	−2.435E−013	−9.3E−014
1466	−1.1E−013	−2E−013	2.2165	−1.60E−014	1.3435E−013	−4.6E−013
1467	−8.8E−014	−8E−014	2.2165	5.780E−014	−2.211E−013	6.308E−013
1468	3.01E−013	3.2E−013	2.2165	−8.14E−014	−3.106E−013	−4.8E−013
1469	−1.0E−013	−2E−013	2.2165	−5.57E−014	−1.013E−013	−8.4E−014
1470	−8.4E−015	2.6E−013	2.2165	4.713E−014	−9.854E−014	2.265E−013
1471	3.50E−013	4.0E−013	2.2165	2.048E−015	−2.475E−013	3.477E−013
1472	1.98E−014	1.7E−013	2.2165	−2.67E−014	5.3084E−013	3.854E−013
1473	3.55E−013	3.0E−013	2.2165	2.456E−014	−8.488E−014	−4.8E−013
1474	−3.1E−013	−7E−014	2.2165	1.238E−013	3.094E−013	2.888E−013
1475	−8.4E−014	−5E−014	2.2165	4.945E−014	−1.226E−013	2.649E−013
1476	−1.5E−013	−4E−013	2.2165	2.660E−014	1.434E−013	6.484E−014
1477	4.32E−013	4.7E−013	2.2165	4.778E−015	7.1722E−014	1.065E−013
1478	−2.0E−013	−3E−014	2.2165	−1.90E−014	1.2696E−013	7.515E−013
1479	−2.8E−013	−3E−013	2.2165	2.867E−014	7.8976E−014	6.611E−014
1480	−3.9E−013	−4E−013	2.2165	5.986E−015	3.0683E−013	5.250E−013
1481	−2.8E−013	−6E−013	2.2165	−2.83E−014	2.8267E−013	−1.7E−013
1482	−1.4E−013	−8E−013	2.2165	−9.99E−014	4.3748E−015	6.585E−013
1483	−8.0E−014	−8E−013	2.2165	−2.01E−013	1.8815E−013	3.890E−013
1484	1.62E−013	−1E−012	2.2165	−2.49E−013	−2.120E−013	6.095E−013
1485	−1.2E−012	−3E−012	2.2165	−2.47E−013	−4.687E−013	1.249E−012
1486	−5.1E−013	−5E−012	2.2165	−5.41E−013	−1.367E−012	1.948E−012
1487	−2.0E−012	−8E−012	2.2165	−7.96E−013	−2.105E−012	3.057E−012
1488	−8.8E−013	−1E−011	2.2165	−1.48E−012	−4.567E−012	6.488E−012
1489	−4.9E−012	−2E−011	2.2165	−2.25E−012	−5.972E−012	8.343E−012
1490	−2.4E−012	−4E−011	2.2165	−4.44E−012	−1.244E−011	1.991E−011
1491	−1.4E−011	−6E−011	2.2165	−6.66E−012	−1.617E−011	2.540E−011
1492	−6.5E−012	−1E−010	2.2165	−1.28E−011	−3.470E−011	5.609E−011
1493	−4.0E−011	−2E−010	2.2165	−1.93E−011	−4.664E−011	7.348E−011
1494	−2.2E−011	−3E−010	2.2165	−3.63E−011	−9.836E−011	1.591E−010
1495	−1.1E−010	−5E−010	2.2165	−5.57E−011	−1.366E−010	2.149E−010
1496	−6.8E−011	−9E−010	2.2165	−1.04E−010	−2.816E−010	4.521E−010
1497	−3.1E−010	−2E−009	2.2165	−1.61E−010	−3.993E−010	6.298E−010
1498	−2E−010	−3E−009	2.2165	−2.98E−010	−8.039E−010	1.285E−009
1499	−9.0E−010	−4E−009	2.2165	−4.67E−010	−1.164E−009	1.842E−009
1500	−5.8E−010	−7E−009	2.2165	−8.55E−010	−2.296E−009	3.659E−009
1501	−2.6E−009	−1E−008	2.2165	−1.35E−009	−3.392E−009	5.386E−009

1502	−1.6E−009	−2E−008	2.2165	−2.46E−009	−6.560E−009	1.041E−008
1503	−7.7E−009	−4E−008	2.2165	−3.90E−009	−9.887E−009	1.575E−008
1504	−4.3E−009	−6E−008	2.2165	−7.07E−009	−1.874E−008	2.964E−008
1505	−2.3E−008	−1E−007	2.2165	−1.13E−008	−2.883E−008	4.603E−008
1506	−1.1E−008	−2E−007	2.2165	−2.04E−008	−5.355E−008	8.431E−008
1507	−7.0E−008	−3E−007	2.2165	−3.24E−008	−8.417E−008	1.346E−007
1508	−2.3E−008	−5E−007	2.2165	−5.87E−008	−1.529E−007	2.394E−007
1509	−2.2E−007	−9E−007	2.2165	−9.34E−008	−2.463E−007	3.942E−007
1510	−3.7E−008	−1E−006	2.2165	−1.69E−007	−4.359E−007	6.786E−007
1511	−6.8E−007	−3E−006	2.2165	−2.68E−007	−7.233E−007	1.156E−006
1512	1.44E−008	−4E−006	2.2165	−4.89E−007	−1.241E−006	1.916E−006
1513	−2.2E−006	−8E−006	2.2165	−7.70E−007	−2.137E−006	3.4E−006
1514	5.28E−007	−1E−005	2.2165	−1.42E−006	−3.522E−006	5.380E−006
1515	−7.3E−006	−2E−005	2.2164	−2.21E−006	−6.367E−006	1.004E−005
1516	3.46E−006	−3E−005	2.2164	−4.11E−006	−9.971E−006	1.498E−005
1517	−2.5E−005	−7E−005	2.2163	−6.30E−006	−1.923E−005	2.977E−005
1518	1.78E−005	−7E−005	2.2161	−1.20E−005	−2.82E−005	4.123E−005
1519	−8.7E−005	−0.00023	2.2158	−1.80E−005	−5.929E−005	8.902E−005
1520	8.39E−005	−0.00018	2.2153	−3.50E−005	−8.021E−005	0.00011186
1521	−0.000321	−0.00073	2.2144	−5.16E−005	−0.00018902	0.00027006
1522	0.00039323	−0.00040	2.2128	−0.0001046	−0.00023494	0.00030191
1523	−0.001295	−0.00247	2.2102	−0.0001529	−0.00063891	0.00084654
1524	0.0020145	−0.00056	2.2055	−0.0003296	−0.00076194	0.00086209
1525	−0.006291	−0.00969	2.1967	−0.0005068	−0.0024157	0.0029059
1526	0.012779	0.0028793	2.1793	−0.0012632	−0.0032786	0.0031784
1527	−0.037796	−0.04855	2.1255	−0.0015614	−0.012957	0.012956
1528	0.080882	0.043625	2.0092	−0.013804	−0.0076766	0.051928
1529	−0.026163	0.056051	2.2586	0.0026513	0.09443	−0.29655
1530	2.7235	2.732	2.9466	8.873E−012	0.67014	−7.4E−011
1531	2.2543	2.2088	2.5136	0.0050237	0.14985	0.64668
1532	1.9985	1.9808	2.2709	0.01887	0.33185	0.49479
1533	1.9808	1.9985	2.2709	0.01887	0.49479	0.33185
1534	2.2088	2.2543	2.5136	0.0050237	0.64668	0.14985
1535	2.7235	2.732	2.9466	8.538E−012	−0.67014	7.472E−011
1536	−0.05959	−0.19284	2.4502	3.824E−011	0.28885	−1.1E−014
1537	0.073965	0.14614	1.7645	−1.09E−011	−0.053813	1.499E−011
1538	−0.094119	−0.07982	2.181	−6.67E−012	−0.0034843	−8.6E−012
1539	0.018333	0.030904	2.1937	−1.41E−012	−0.003213	−2.8E−012
1540	−0.017994	−0.01294	2.1929	−1.65E−012	−0.00084663	−8.1E−013
1541	0.002631	0.0050713	2.2097	−2.76E−013	−0.00060514	−5.6E−013
1542	−0.004275	−0.00259	2.2089	−4.88E−013	−0.00036013	−2.1E−013
1543	0.00031609	0.0010282	2.214	−9.84E−014	−0.00014616	−1.3E−013
1544	−0.001157	−0.00059	2.214	−1.40E−013	−0.00014228	−6.4E−014
1545	−1.4E−005	0.0002221	2.2155	−2.48E−014	−4.095E−005	−1.7E−014
1546	−0.000335	−0.00014	2.2157	−5.93E−014	−5.343E−005	−1.6E−014
1547	−3.4E−005	4.7E−005	2.2162	−1.18E−014	−1.243E−005	3.327E−014
1548	−0.000101	−3E−005	2.2162	−1.39E−014	−1.949E−005	3.846E−014

1549	−1.9E−005	9.2E−006	2.2164	−8.94E−015	−3.919E−006	8.420E−015
1550	−3.1E−005	−8E−006	2.2164	−5.91E−015	−6.995E−006	−6.6E−015
1551	−8.4E−006	1.4E−006	2.2165	−1.57E−014	−1.256E−006	8.369E−014
1552	−9.9E−006	−2E−006	2.2165	1.823E−014	−2.490E−006	1.277E−014
1553	−3.4E−006	2.7E−008	2.2165	1.015E−014	−4.032E−007	−8.6E−014
1554	−3.2E−006	−5E−007	2.2165	−2.88E−014	−8.841E−007	9.022E−014
1555	−1.3E−006	−1E−007	2.2165	1.422E−015	−1.282E−007	2.536E−015
1556	−1.1E−006	−1E−007	2.2165	−4.47E−015	−3.140E−007	2.256E−014
1557	−4.8E−007	−7E−008	2.2165	1.101E−014	−3.991E−008	1.839E−014
1558	−3.5E−007	−2E−008	2.2165	−1.71E−014	−1.118E−007	5.589E−014
1559	−1.7E−007	−3E−008	2.2165	1.27E−014	−1.199E−008	−2.7E−014
1560	−1.2E−007	−1E−009	2.2165	−2.51E−014	−3.995E−008	3.589E−014
1561	−6.2E−008	−1E−008	2.2165	2.657E−014	−3.393E−009	9.176E−014
1562	−4E−008	9.4E−010	2.2165	−4.63E−014	−1.434E−008	1.519E−013
1563	−2.2E−008	−6E−009	2.2165	−8.68E−015	−8.613E−010	7.843E−014
1564	−1.4E−008	7.8E−010	2.2165	2.103E−014	−5.175E−009	1.084E−013
1565	−7.7E−009	−2E−009	2.2165	−2.14E−014	−1.692E−010	6.497E−015
1566	−4.7E−009	4.2E−010	2.2165	1.661E−014	−1.877E−009	4.172E−014
1567	−2.7E−009	−9E−010	2.2165	2.947E−014	−6.755E−012	−1.3E−014
1568	−1.6E−009	2.0E−010	2.2165	−2.22E−014	−6.843E−010	−1.2E−013
1569	−9.5E−010	−3E−010	2.2165	2.746E−015	1.8736E−011	7.794E−014
1570	−5.5E−010	8.7E−011	2.2165	1.076E−014	−2.51E−010	1.245E−013
1571	−3.3E−010	−1E−010	2.2165	9.087E−015	1.5268E−011	1.482E−013
1572	−1.9E−010	3.8E−011	2.2165	−2.41E−014	−9.257E−011	2.980E−013
1573	−1.2E−010	−5E−011	2.2165	5.153E−014	8.423E−012	7.869E−014
1574	−6.5E−011	1.6E−011	2.2165	1.116E−014	−3.331E−011	−8.4E−015
1575	−4.1E−011	−2E−011	2.2165	2.592E−014	4.1493E−012	−8.2E−015
1576	−2.3E−011	6.3E−012	2.2165	−1.57E−014	−1.298E−011	−1.2E−013
1577	−1.4E−011	−7E−012	2.2165	−2.78E−014	1.4476E−012	2.558E−013
1578	−7.9E−012	2.2E−012	2.2165	2.581E−014	−4.849E−012	1.123E−013
1579	−4.5E−012	−2E−012	2.2165	1.363E−014	8.1895E−013	−6.4E−014
1580	−2.6E−012	8.7E−013	2.2165	−1.37E−014	−1.659E−012	−1.0E−013
1581	−1.8E−012	−1E−012	2.2165	4.419E−015	5.8037E−013	−3.7E−013
1582	−7.6E−013	7.6E−013	2.2165	−4.25E−014	−3.540E−013	2.239E−013
1583	−8E−013	−7E−013	2.2165	9.636E−015	8.4618E−014	8.986E−014
1584	−4.0E−013	1.5E−014	2.2165	3.612E−014	1.0622E−013	−4.6E−014
1585	1.06E−013	2.9E−014	2.2165	−1.96E−014	−4.774E−013	−4.1E−014
1586	−4.0E−013	−3E−013	2.2165	−7.62E−015	−3.431E−013	−2.1E−013
1587	−2.3E−013	−4E−013	2.2165	1.423E−014	−2.191E−013	−4.8E−014
1588	4.77E−013	2.1E−013	2.2165	−5.32E−014	3.9482E−014	−1.5E−013
1589	3.37E−013	2.6E−013	2.2165	6.415E−014	2.4187E−013	1.502E−013
1590	3.78E−013	−8E−014	2.2165	−9.04E−015	2.6694E−014	−1.1E−013
1591	8.64E−013	6.7E−013	2.2165	−3.65E−014	−7.302E−013	1.014E−013
1592	2.89E−013	−7E−014	2.2165	6.364E−014	1.5871E−014	1.613E−013
1593	2.44E−013	−3E−013	2.2165	−3.11E−014	7.8102E−013	4.634E−014
1594	3.78E−013	3.8E−013	2.2165	4.202E−014	1.2984E−013	−3.2E−014
1595	−2.1E−013	−2E−013	2.2165	−3.56E−014	−4.299E−014	1.264E−013

1596	−2.0E−014	−3E−014	2.2165	−3.35E−014	2.6203E−013	1.901E−013
1597	−2.6E−013	−1E−013	2.2165	4.975E−014	3.2428E−013	−1.0E−013
1598	3.24E−013	−3E−013	2.2165	−3.24E−014	−1.182E−013	−7.0E−014
1599	6.50E−013	8.1E−013	2.2165	7.159E−015	−2.420E−013	−2.5E−013
1600	3.80E−013	−3E−014	2.2165	2.318E−014	−4.843E−013	−1.3E−013
1601	2.01E−013	1.9E−013	2.2165	1.471E−014	5.6388E−013	−4.2E−013
1602	−3.1E−013	3.2E−014	2.2165	−9.57E−014	9.5386E−014	−1.6E−014
1603	2.50E−013	4.5E−013	2.2165	−5.49E−015	−2.612E−013	2.535E−013
1604	−6.0E−013	−8E−013	2.2165	5.101E−014	3.2348E−014	1.092E−013
1605	−1.1E−013	2.5E−013	2.2165	−1.61E−014	6.9042E−013	9.506E−014
1606	8.22E−015	−1E−013	2.2165	−1.91E−014	−5.824E−013	1.011E−013
1607	−4.7E−013	−3E−013	2.2165	−1.43E−016	−4.084E−013	4.215E−013
1608	−5.6E−013	−2E−013	2.2165	−1.92E−014	−8.151E−013	2.333E−013
1609	−4.2E−013	−8E−013	2.2165	6.953E−014	1.0227E−012	−4.2E−013
1610	−7.7E−015	5.0E−013	2.2165	−1.05E−013	7.0907E−014	−4.2E−013
1611	5.18E−013	−4E−013	2.2165	4.842E−014	3.5984E−013	2.644E−013
1612	2.72E−013	−1E−013	2.2165	−3.45E−014	7.5182E−013	4.618E−014
1613	1.34E−013	−4E−013	2.2165	−5.21E−015	6.3803E−013	−1.3E−013
1614	5.39E−013	3.4E−013	2.2165	8.570E−014	−5.774E−013	−1.6E−013
1615	8.98E−014	−3E−013	2.2165	−1.18E−013	−1.200E−013	−3.1E−013
1616	1.56E−013	2.2E−013	2.2165	1.238E−013	−2.347E−013	−1.5E−013
1617	−5.2E−013	−3E−013	2.2165	−1.59E−013	−1.874E−013	3.667E−013
1618	−1.2E−013	7.7E−014	2.2165	3.203E−014	8.606E−013	4.527E−013
1619	−6.1E−013	−7E−013	2.2165	3.751E−014	−3.551E−013	2.044E−013
1620	−8.7E−013	1.0E−014	2.2165	−6.94E−014	5.3675E−013	2.690E−013
1621	−1.3E−013	−6E−014	2.2165	7.520E−014	−4.789E−013	2.582E−013
1622	−1.9E−013	4.7E−014	2.2165	−4.07E−014	7.224E−013	9.015E−014
1623	−5.2E−013	−2E−013	2.2165	1.148E−014	−7.388E−013	−3.4E−014
1624	−1.1E−013	−3E−013	2.2165	−1.95E−014	5.0313E−013	−4.4E−013
1625	1.78E−013	4.5E−013	2.2165	3.469E−015	−1.101E−012	−2.3E−013
1626	−1.4E−012	9.9E−014	2.2165	−4.88E−014	1.5274E−012	1.445E−014
1627	−1.5E−012	−4E−013	2.2165	3.356E−014	−3.046E−013	−2.9E−014
1628	−2.7E−012	4.1E−013	2.2165	1.946E−014	2.2129E−012	−5.6E−013
1629	−5.1E−012	−3E−012	2.2165	−7.06E−015	−1.777E−012	−3.4E−013
1630	−7.6E−012	3.4E−012	2.2165	2.577E−014	4.9634E−012	2.882E−014
1631	−1.4E−011	−7E−012	2.2165	6.695E−014	−1.235E−012	1.856E−013
1632	−2.3E−011	5.9E−012	2.2165	4.699E−014	1.3265E−011	−5.8E−013
1633	−3.9E−011	−2E−011	2.2165	−8.52E−014	−3.326E−012	9.448E−014
1634	−6.5E−011	1.7E−011	2.2165	3.549E−014	3.4819E−011	−1.3E−013
1635	−1.2E−010	−5E−011	2.2165	3.342E−015	−9.072E−012	−3.0E−014
1636	−1.9E−010	3.7E−011	2.2165	−1.57E−014	9.1739E−011	8.743E−014
1637	−3.3E−010	−1E−010	2.2165	7.078E−014	−1.620E−011	8.110E−014
1638	−5.5E−010	8.6E−011	2.2165	−1.22E−013	2.5133E−010	4.447E−013
1639	−9.5E−010	−3E−010	2.2165	7.199E−015	−1.736E−011	4.930E−013
1640	−1.6E−009	2.0E−010	2.2165	9.110E−014	6.8391E−010	1.282E−013
1641	−2.7E−009	−9E−010	2.2165	−6.14E−014	6.0678E−012	5.313E−014
1642	−4.7E−009	4.2E−010	2.2165	−8.26E−014	1.8765E−009	5.886E−013

1643	−7.7E−009	−2E−009	2.2165	1.290E−013	1.6894E−010	−1.8E−013
1644	−1.4E−008	7.8E−010	2.2165	9.088E−015	5.1754E−009	−3.7E−013
1645	−2.2E−008	−6E−009	2.2165	−9.99E−015	8.6032E−010	−2.7E−013
1646	−4E−008	9.4E−010	2.2165	3.753E−014	1.4342E−008	3.677E−013
1647	−6.2E−008	−1E−008	2.2165	−9.15E−015	3.3924E−009	2.208E−013
1648	−1.2E−007	−1E−009	2.2165	9.953E−014	3.9947E−008	7.624E−013
1649	−1.7E−007	−3E−008	2.2165	−2.87E−014	1.1988E−008	−7.6E−013
1650	−3.5E−007	−2E−008	2.2165	−6.33E−014	1.1178E−007	−1.8E−013
1651	−4.8E−007	−7E−008	2.2165	2.877E−014	3.9916E−008	−1.2E−013
1652	−1.1E−006	−1E−007	2.2165	−1.29E−013	3.1397E−007	4.726E−013
1653	−1.3E−006	−1E−007	2.2165	5.391E−014	1.2822E−007	8.871E−013
1654	−3.2E−006	−5E−007	2.2165	−3.29E−014	8.8405E−007	−3.8E−015
1655	−3.4E−006	2.7E−008	2.2165	−4.03E−014	4.0324E−007	−2.7E−013
1656	−9.9E−006	−2E−006	2.2165	1.431E−013	2.4903E−006	4.601E−014
1657	−8.4E−006	1.4E−006	2.2165	−1.12E−013	1.256E−006	2.825E−013
1658	−3.1E−005	−8E−006	2.2164	3.825E−014	6.9949E−006	4.568E−013
1659	−1.9E−005	9.2E−006	2.2164	−9.05E−014	3.9189E−006	−1.9E−013
1660	−0.000101	−3E−005	2.2162	−8.53E−014	1.9491E−005	3.905E−013
1661	−3.4E−005	4.7E−005	2.2162	7.642E−014	1.2427E−005	−9.7E−014
1662	−0.000335	−0.00014	2.2157	−1.86E−013	5.3435E−005	−1.9E−013
1663	−1.4E−005	0.0002221	2.2155	−1.41E−014	4.095E−005	8.070E−014
1664	−0.001157	−0.00059	2.214	−1.07E−013	0.00014228	−2.7E−013
1665	0.00031609	0.0010282	2.214	−1.62E−013	0.00014616	6.424E−013
1666	−0.004275	−0.00259	2.2089	−5.07E−013	0.00036013	−2.4E−013
1667	0.002631	0.0050713	2.2097	−1.53E−013	0.00060514	6.805E−013
1668	−0.017994	−0.01294	2.1929	−1.82E−012	0.00084663	5.557E−013
1669	0.018333	0.030904	2.1937	−1.44E−012	0.003213	3.079E−012
1670	−0.094119	−0.07982	2.181	−6.63E−012	0.0034843	9.040E−012
1671	0.073965	0.14614	1.7645	−1.09E−011	0.053813	−1.5E−011
1672	−0.05959	−0.19284	2.4502	3.813E−011	−0.28885	7.113E−013
1673	2.2543	2.2088	2.5136	0.0050237	−0.14985	−0.64668
1674	1.9985	1.9808	2.2709	0.01887	−0.33185	−0.49479
1675	1.9808	1.9985	2.2709	0.01887	−0.49479	−0.33185
1676	2.2088	2.2543	2.5136	0.0050237	−0.64668	−0.14985
1677	−0.026163	0.056051	2.2586	−0.0026513	0.09443	0.29655
1678	0.080882	0.043625	2.0092	0.013804	−0.0076766	−0.051928
1679	−0.037796	−0.04855	2.1255	0.0015614	−0.012957	−0.012956
1680	0.012779	0.0028793	2.1793	0.0012632	−0.0032786	−0.0031784
1681	−0.006291	−0.00969	2.1967	0.0005068	−0.0024157	−0.0029059
1682	0.0020145	−0.00056	2.2055	0.00032964	−0.00076194	−0.0008621
1683	−0.001295	−0.00247	2.2102	0.00015287	−0.00063891	−0.0008465
1684	0.00039323	−0.00040	2.2128	0.00010459	−0.00023494	−0.0003019
1685	−0.000321	−0.00073	2.2144	5.158E−005	−0.00018902	−0.0002701
1686	8.39E−005	−0.00018	2.2153	3.503E−005	−8.021E−005	−0.0001119
1687	−8.7E−005	−0.00023	2.2158	1.798E−005	−5.929E−005	−8.9E−005
1688	1.78E−005	−7E−005	2.2161	1.195E−005	−2.82E−005	−4.1E−005
1689	−2.5E−005	−7E−005	2.2163	6.300E−006	−1.923E−005	−3.0E−005

1690	3.46E−006	−3E−005	2.2164	4.106E−006	−9.971E−006	−1.5E−005
1691	−7.3E−006	−2E−005	2.2164	2.206E−006	−6.367E−006	−1.0E−005
1692	5.28E−007	−1E−005	2.2165	1.416E−006	−3.522E−006	−5.4E−006
1693	−2.2E−006	−8E−006	2.2165	7.704E−007	−2.137E−006	−3.4E−006
1694	1.44E−008	−4E−006	2.2165	4.891E−007	−1.241E−006	−1.9E−006
1695	−6.8E−007	−3E−006	2.2165	2.685E−007	−7.233E−007	−1.2E−006
1696	−3.7E−008	−1E−006	2.2165	1.693E−007	−4.359E−007	−6.8E−007
1697	−2.2E−007	−9E−007	2.2165	9.339E−008	−2.463E−007	−3.9E−007
1698	−2.3E−008	−5E−007	2.2165	5.867E−008	−1.529E−007	−2.4E−007
1699	−7.0E−008	−3E−007	2.2165	3.243E−008	−8.417E−008	−1.3E−007
1700	−1.1E−008	−2E−007	2.2165	2.036E−008	−5.355E−008	−8.4E−008
1701	−2.3E−008	−1E−007	2.2165	1.125E−008	−2.883E−008	−4.6E−008
1702	−4.3E−009	−6E−008	2.2165	7.071E−009	−1.874E−008	−3.0E−008
1703	−7.7E−009	−4E−008	2.2165	3.899E−009	−9.887E−009	−1.6E−008
1704	−1.6E−009	−2E−008	2.2165	2.458E−009	−6.559E−009	−1.0E−008
1705	−2.6E−009	−1E−008	2.2165	1.350E−009	−3.392E−009	−5.4E−009
1706	−5.8E−010	−7E−009	2.2165	8.554E−010	−2.296E−009	−3.7E−009
1707	−9E−010	−4E−009	2.2165	4.668E−010	−1.164E−009	−1.8E−009
1708	−2E−010	−3E−009	2.2165	2.979E−010	−8.038E−010	−1.3E−009
1709	−3.1E−010	−2E−009	2.2165	1.613E−010	−3.990E−010	−6.3E−010
1710	−6.8E−011	−9E−010	2.2165	1.038E−010	−2.818E−010	−4.5E−010
1711	−1.1E−010	−5E−010	2.2165	5.565E−011	−1.366E−010	−2.1E−010
1712	−2.3E−011	−3E−010	2.2165	3.619E−011	−9.878E−011	−1.6E−010
1713	−3.9E−011	−2E−010	2.2165	1.918E−011	−4.660E−011	−7.3E−011
1714	−7.1E−012	−1E−010	2.2165	1.264E−011	−3.462E−011	−5.6E−011
1715	−1.4E−011	−6E−011	2.2165	6.632E−012	−1.612E−011	−2.5E−011
1716	−2.0E−012	−4E−011	2.2165	4.406E−012	−1.208E−011	−2.0E−011
1717	−5.1E−012	−2E−011	2.2165	2.249E−012	−5.405E−012	−8.2E−012
1718	−6.5E−013	−1E−011	2.2165	1.551E−012	−4.159E−012	−6.7E−012
1719	−1.9E−012	−8E−012	2.2165	7.759E−013	−1.855E−012	−3.0E−012
1720	−3.1E−013	−4E−012	2.2165	5.266E−013	−1.379E−012	−2.7E−012
1721	−6.4E−013	−3E−012	2.2165	2.631E−013	−6.990E−013	−7.9E−013
1722	−3.1E−013	−2E−012	2.2165	1.523E−013	−6.197E−013	−6.2E−013
1723	−1.9E−013	−9E−013	2.2165	1.185E−013	−2.474E−013	−2.8E−013
1724	−1.2E−013	−6E−013	2.2165	8.408E−014	−2.476E−013	−3.6E−013
1725	−1.8E−013	−4E−013	2.2165	3.576E−014	3.3595E−015	−3.6E−013
1726	8.48E−014	−8E−014	2.2165	−1.04E−014	−2.259E−013	−2.2E−013
1727	1.68E−013	3.1E−013	2.2165	−6.41E−015	1.432E−016	1.995E−013
1728	2.09E−013	1.7E−013	2.2165	−6.55E−015	−4.182E−014	−3.9E−013
1729	−8.6E−014	−8E−014	2.2165	−1.54E−014	4.5214E−014	2.509E−013
1730	−2.7E−013	−1E−013	2.2165	−1.54E−014	−7.428E−015	2.420E−014
1731	7.62E−014	1.3E−013	2.2165	2.982E−015	2.3064E−014	1.406E−013
1732	−4.2E−014	−1E−013	2.2165	−6.00E−015	1.8258E−014	3.606E−014
1733	−5.2E−015	−1E−014	2.2165	−2.63E−014	−6.025E−014	2.235E−013
1734	1.52E−013	2.1E−013	2.2165	3.487E−014	−1.184E−013	1.584E−013
1735	−4.4E−014	−2E−013	2.2165	5.240E−014	1.6036E−014	1.852E−013
1736	−2.3E−013	−3E−013	2.2165	2.345E−014	7.4621E−014	2.794E−013

1737	−1.8E−013	−3E−013	2.2165	−5.18E−014	1.5709E−015	−1.8E−013
1738	5.53E−014	7.4E−014	2.2165	−3.50E−014	−5.595E−014	2.410E−013
1739	3.13E−014	−1E−013	2.2165	2.797E−014	9.3436E−015	1.038E−013
1740	−2.2E−013	−3E−013	2.2165	−1.51E−014	−2.591E−013	−5.9E−013
1741	−3.5E−014	−2E−013	2.2165	−4.14E−014	2.2008E−013	1.764E−013
1742	−3.2E−013	−3E−013	2.2165	4.311E−014	−7.021E−014	−4.3E−013
1743	1.30E−013	2.0E−013	2.2165	5.251E−014	1.522E−013	1.624E−013
1744	−1.1E−013	1.7E−014	2.2165	4.707E−014	1.8419E−013	−1.7E−013
1745	1.61E−013	1.3E−013	2.2165	1.920E−014	1.6865E−013	−2.6E−013
1746	1.95E−013	3.6E−013	2.2165	−5.02E−015	7.6203E−014	4.884E−014
1747	1.97E−013	1.8E−013	2.2165	−7.79E−015	2.9281E−013	3.504E−013
1748	−4.1E−013	−3E−013	2.2165	9.559E−014	−1.318E−013	−2.2E−013
1749	2.34E−013	3.8E−013	2.2165	2.615E−014	2.5803E−013	1.134E−013
1750	5.94E−014	−1E−013	2.2165	−6.64E−014	−1.142E−013	2.666E−013
1751	−2.7E−013	9.2E−014	2.2165	−4.05E−015	1.8763E−013	−2.6E−013
1752	3.83E−013	3.2E−013	2.2165	1.409E−013	1.0416E−013	3.296E−013
1753	−2.8E−013	−4E−013	2.2165	5.328E−015	−1.474E−013	−7.3E−014
1754	−2.5E−013	−3E−013	2.2165	−6.94E−014	−4.501E−014	−9.3E−014
1755	9.54E−014	2.2E−014	2.2165	−1.06E−014	1.1002E−013	−2.7E−013
1756	−3.1E−013	−3E−013	2.2165	−5.28E−014	−7.698E−014	−1.8E−013
1757	2.99E−013	3.5E−013	2.2165	−1.59E−014	1.106E−013	5.430E−013
1758	1.15E−013	3.1E−013	2.2165	−7.61E−014	1.464E−013	−1.7E−013
1759	1.91E−013	4.4E−013	2.2165	−5.43E−014	7.7972E−014	6.209E−013
1760	2.15E−013	3.7E−013	2.2165	−3.85E−014	2.8471E−013	8.310E−013
1761	7.83E−014	2.5E−013	2.2165	−5.87E−015	3.7384E−013	1.321E−013
1762	1.29E−013	−3E−014	2.2165	5.398E−014	2.2221E−014	−1.0E−013
1763	−2.2E−013	−3E−013	2.2165	−7.32E−014	7.0523E−014	−4.8E−013
1764	−4.5E−013	−6E−013	2.2165	1.598E−014	7.192E−014	−3.8E−013
1765	6.35E−014	−2E−013	2.2165	3.481E−014	−2.418E−014	5.337E−013
1766	−2.5E−013	−6E−013	2.2165	1.461E−013	2.0732E−013	2.097E−013
1767	−2.3E−013	−8E−013	2.2165	2.165E−013	−2.028E−014	1.512E−013
1768	−1.2E−013	−2E−012	2.2165	2.645E−013	3.3873E−013	5.204E−013
1769	−1.1E−012	−3E−012	2.2165	2.356E−013	6.2997E−013	1.047E−012
1770	−3.6E−014	−4E−012	2.2165	6.042E−013	1.3844E−012	2.839E−012
1771	−1.5E−012	−7E−012	2.2165	8.085E−013	2.1163E−012	2.936E−012
1772	−1.2E−012	−1E−011	2.2165	1.546E−012	4.357E−012	6.407E−012
1773	−4.8E−012	−2E−011	2.2165	2.301E−012	5.3901E−012	8.739E−012
1774	−1.8E−012	−4E−011	2.2165	4.319E−012	1.2203E−011	1.950E−011
1775	−1.3E−011	−6E−011	2.2165	6.711E−012	1.6333E−011	2.507E−011
1776	−6.7E−012	−1E−010	2.2165	1.264E−011	3.504E−011	5.670E−011
1777	−4.0E−011	−2E−010	2.2165	1.922E−011	4.7307E−011	7.291E−011
1778	−2.2E−011	−3E−010	2.2165	3.634E−011	9.8797E−011	1.594E−010
1779	−1.1E−010	−5E−010	2.2165	5.571E−011	1.3667E−010	2.141E−010
1780	−6.7E−011	−9E−010	2.2165	1.039E−010	2.8171E−010	4.521E−010
1781	−3.1E−010	−2E−009	2.2165	1.613E−010	3.9885E−010	6.285E−010
1782	−2E−010	−3E−009	2.2165	2.979E−010	8.0365E−010	1.286E−009
1783	−9.0E−010	−4E−009	2.2165	4.668E−010	1.164E−009	1.841E−009

1784	−5.8E−010	−7E−009	2.2165	8.553E−010	2.2958E−009	3.659E−009
1785	−2.6E−009	−1E−008	2.2165	1.350E−009	3.3923E−009	5.387E−009
1786	−1.6E−009	−2E−008	2.2165	2.458E−009	6.5598E−009	1.042E−008
1787	−7.7E−009	−4E−008	2.2165	3.899E−009	9.8869E−009	1.575E−008
1788	−4.3E−009	−6E−008	2.2165	7.071E−009	1.8744E−008	2.964E−008
1789	−2.3E−008	−1E−007	2.2165	1.125E−008	2.883E−008	4.603E−008
1790	−1.1E−008	−2E−007	2.2165	2.036E−008	5.3549E−008	8.431E−008
1791	−7.0E−008	−3E−007	2.2165	3.243E−008	8.4171E−008	1.346E−007
1792	−2.3E−008	−5E−007	2.2165	5.867E−008	1.5288E−007	2.394E−007
1793	−2.2E−007	−9E−007	2.2165	9.339E−008	2.4629E−007	3.942E−007
1794	−3.7E−008	−1E−006	2.2165	1.693E−007	4.3588E−007	6.786E−007
1795	−6.8E−007	−3E−006	2.2165	2.685E−007	7.2333E−007	1.156E−006
1796	1.44E−008	−4E−006	2.2165	4.891E−007	1.2405E−006	1.916E−006
1797	−2.2E−006	−8E−006	2.2165	7.704E−007	2.1367E−006	3.4E−006
1798	5.28E−007	−1E−005	2.2165	1.416E−006	3.5215E−006	5.380E−006
1799	−7.3E−006	−2E−005	2.2164	2.206E−006	6.3674E−006	1.004E−005
1800	3.46E−006	−3E−005	2.2164	4.106E−006	9.9711E−006	1.498E−005
1801	−2.5E−005	−7E−005	2.2163	6.300E−006	1.9231E−005	2.977E−005
1802	1.78E−005	−7E−005	2.2161	1.195E−005	2.82E−005	4.123E−005
1803	−8.7E−005	−0.00023	2.2158	1.798E−005	5.9293E−005	8.902E−005
1804	8.39E−005	−0.00018	2.2153	3.503E−005	8.0208E−005	0.00011186
1805	−0.000321	−0.00073	2.2144	5.158E−005	0.00018902	0.00027006
1806	0.00039323	−0.00040	2.2128	0.00010459	0.00023494	0.00030191
1807	−0.001295	−0.00247	2.2102	0.00015287	0.00063891	0.00084654
1808	0.0020145	−0.00056	2.2055	0.00032964	0.00076194	0.00086209
1809	−0.006291	−0.00969	2.1967	0.0005068	0.0024157	0.0029059
1810	0.012779	0.0028793	2.1793	0.0012632	0.0032786	0.0031784
1811	−0.037796	−0.04855	2.1255	0.0015614	0.012957	0.012956
1812	0.080882	0.043625	2.0092	0.013804	0.0076766	0.051928
1813	−0.026163	0.056051	2.2586	−0.0026513	−0.09443	−0.29655
1814	0.12476	0.14775	2.2317	0.00059656	0.17378	0.24358
1815	0.031499	0.024142	2.0661	0.019639	−0.017146	−0.030482
1816	−0.028413	−0.03356	2.122	0.0064352	−0.015209	−0.020131
1817	0.005954	0.0024288	2.1767	0.002803	−0.0077935	−0.0080758
1818	−0.005728	−0.00743	2.1914	0.001504	−0.0043544	−0.0043194
1819	0.0012625	0.0003362	2.2053	0.00096815	−0.0020294	−0.0022117
1820	−0.001467	−0.00204	2.2089	0.00049055	−0.0012679	−0.0012878
1821	0.00028079	−7E−006	2.2129	0.00033353	−0.0006382	−0.0007069
1822	−0.000413	−0.00061	2.2141	0.00016992	−0.00040537	−0.0004266
1823	5.74E−005	−4E−005	2.2153	0.00011529	−0.00021449	−0.0002397
1824	−0.000121	−0.00019	2.2157	5.956E−005	−0.00013508	−0.0001459
1825	9.13E−006	−2E−005	2.2161	3.984E−005	−7.369E−005	−8.3E−005
1826	−3.6E−005	−6E−005	2.2162	2.09E−005	−4.583E−005	−5.0E−005
1827	2.47E−007	−1E−005	2.2164	1.376E−005	−2.55E−005	−2.9E−005
1828	−1.1E−005	−2E−005	2.2164	7.323E−006	−1.569E−005	−1.7E−005
1829	−7.1E−007	−5E−006	2.2165	4.748E−006	−8.846E−006	−9.9E−006
1830	−3.4E−006	−6E−006	2.2165	2.562E−006	−5.396E−006	−6.0E−006

1831	−4.6E−007	−2E−006	2.2165	1.639E−006	−3.070E−006	−3.5E−006
1832	−1.1E−006	−2E−006	2.2165	8.949E−007	−1.862E−006	−2.1E−006
1833	−2.2E−007	−7E−007	2.2165	5.661E−007	−1.066E−006	−1.2E−006
1834	−3.4E−007	−7E−007	2.2165	3.122E−007	−6.436E−007	−7.2E−007
1835	−9.3E−008	−3E−007	2.2165	1.956E−007	−3.698E−007	−4.2E−007
1836	−1.1E−007	−2E−007	2.2165	1.088E−007	−2.228E−007	−2.5E−007
1837	−3.7E−008	−9E−008	2.2165	6.764E−008	−1.283E−007	−1.4E−007
1838	−3.5E−008	−8E−008	2.2165	3.791E−008	−7.719E−008	−8.7E−008
1839	−1.4E−008	−3E−008	2.2165	2.340E−008	−4.449E−008	−5.0E−008
1840	−1.2E−008	−3E−008	2.2165	1.319E−008	−2.677E−008	−3.0E−008
1841	−5.3E−009	−1E−008	2.2165	8.101E−009	−1.542E−008	−1.7E−008
1842	−3.8E−009	−9E−009	2.2165	4.589E−009	−9.286E−009	−1.0E−008
1843	−2.0E−009	−4E−009	2.2165	2.805E−009	−5.347E−009	−6.0E−009
1844	−1.2E−009	−3E−009	2.2165	1.595E−009	−3.223E−009	−3.6E−009
1845	−7.2E−010	−1E−009	2.2165	9.720E−010	−1.853E−009	−2.1E−009
1846	−4.1E−010	−1E−009	2.2165	5.544E−010	−1.119E−009	−1.3E−009
1847	−2.6E−010	−5E−010	2.2165	3.369E−010	−6.419E−010	−7.2E−010
1848	−1.4E−010	−4E−010	2.2165	1.926E−010	−3.887E−010	−4.4E−010
1849	−9.4E−011	−2E−010	2.2165	1.168E−010	−2.223E−010	−2.5E−010
1850	−4.5E−011	−1E−010	2.2165	6.687E−011	−1.351E−010	−1.5E−010
1851	−3.4E−011	−6E−011	2.2165	4.051E−011	−7.704E−011	−8.7E−011
1852	−1.5E−011	−4E−011	2.2165	2.319E−011	−4.684E−011	−5.3E−011
1853	−1.2E−011	−2E−011	2.2165	1.405E−011	−2.683E−011	−3.0E−011
1854	−5E−012	−1E−011	2.2165	8.053E−012	−1.631E−011	−1.8E−011
1855	−4.2E−012	−8E−012	2.2165	4.839E−012	−9.245E−012	−1.1E−011
1856	−1.8E−012	−5E−012	2.2165	2.766E−012	−5.475E−012	−6.4E−012
1857	−1.6E−012	−3E−012	2.2165	1.702E−012	−3.123E−012	−3.7E−012
1858	−4.2E−013	−2E−012	2.2165	9.223E−013	−2.042E−012	−2.3E−012
1859	−8.0E−013	−1E−012	2.2165	5.413E−013	−1.055E−012	−1.4E−012
1860	−3.7E−015	−5E−013	2.2165	3.134E−013	−6.366E−013	−8.9E−013
1861	−3.6E−013	−4E−013	2.2165	2.299E−013	−4.481E−013	−3.8E−013
1862	−1.6E−013	−3E−013	2.2165	1.165E−013	−3.113E−013	−3.9E−013
1863	−2.3E−013	−2E−013	2.2165	−3.14E−015	−1.633E−013	−3.9E−013
1864	−7.0E−014	−2E−013	2.2165	−2.05E−014	3.9654E−014	1.591E−014
1865	−1.4E−013	−1E−013	2.2165	−5.08E−014	1.5174E−013	−1.8E−013
1866	−3.0E−013	−2E−013	2.2165	3.110E−014	−8.338E−017	1.061E−013
1867	1.58E−013	2.8E−013	2.2165	−5.81E−015	6.5239E−014	1.267E−013
1868	−6.7E−014	−8E−014	2.2165	−3.45E−014	−1.351E−013	−1.0E−013
1869	−6.1E−013	−5E−013	2.2165	−1.38E−014	3.9704E−014	1.979E−013
1870	−1.5E−013	−1E−013	2.2165	−4.26E−014	1.4889E−013	1.533E−013
1871	−2.6E−014	6.3E−014	2.2165	9.110E−014	−1.601E−013	4.103E−014
1872	2.89E−013	2.6E−013	2.2165	−2.17E−014	−3.179E−013	−2.8E−014
1873	−2.6E−013	−2E−013	2.2165	−5.63E−015	−5.203E−014	−1.2E−013
1874	−9.8E−014	−4E−014	2.2165	6.901E−016	−2.128E−013	−5.2E−014
1875	2.34E−013	2.3E−013	2.2165	3.336E−014	1.1447E−013	1.834E−013
1876	−6.6E−014	−6E−015	2.2165	8.396E−014	−3.810E−014	1.331E−013
1877	−1.6E−013	−2E−013	2.2165	3.314E−014	2.1021E−013	−1.3E−013

1878	−1.6E−014	−7E−014	2.2165	−1.84E−014	−2.083E−013	−2.2E−013
1879	2.57E−013	2.6E−013	2.2165	2.623E−014	1.3497E−013	6.967E−014
1880	−6.1E−014	2.2E−015	2.2165	4.258E−015	3.5541E−013	−1.2E−013
1881	−5.8E−014	−4E−014	2.2165	1.705E−014	−1.250E−013	−1.9E−013
1882	−4.4E−013	−3E−013	2.2165	−1.21E−013	−2.246E−014	−1.7E−013
1883	−5.5E−014	−5E−014	2.2165	−6.94E−014	1.4344E−013	2.799E−013
1884	3.98E−013	4.9E−013	2.2165	3.324E−014	8.0801E−014	2.245E−013
1885	7.57E−014	1.3E−013	2.2165	3.579E−014	4.0129E−014	−3.3E−013
1886	−1.7E−013	−2E−013	2.2165	−8.03E−014	−1.123E−013	−2.3E−013
1887	−3.2E−013	−2E−013	2.2165	−9.55E−014	−6.685E−014	2.444E−015
1888	2.40E−013	2.7E−013	2.2165	−5.55E−015	2.0122E−014	2.249E−013
1889	−2.2E−013	−7E−014	2.2165	2.843E−014	1.183E−013	1.576E−013
1890	2.79E−014	−2E−013	2.2165	8.241E−015	1.542E−013	2.537E−013
1891	1.47E−013	2.8E−013	2.2165	4.945E−014	7.1196E−014	−1.1E−013
1892	2.90E−013	3.8E−013	2.2165	7.124E−015	8.3489E−014	1.109E−013
1893	−1.4E−013	−4E−014	2.2165	5.466E−014	−1.695E−013	−4.0E−014
1894	−2.0E−013	−1E−013	2.2165	−4.52E−014	−1.505E−014	−1.3E−013
1895	1.99E−013	1.3E−013	2.2165	−8.03E−014	−3.650E−013	−4.9E−014
1896	−4.9E−014	−8E−014	2.2165	−2.76E−014	2.9538E−014	−5.2E−014
1897	−3E−013	−2E−013	2.2165	−1.29E−013	9.6117E−014	−4.4E−014
1898	−2.7E−013	−3E−013	2.2165	−5.46E−014	7.7538E−014	−1.6E−013
1899	−6.7E−013	−8E−013	2.2165	2.337E−014	4.7143E−013	−2.0E−013
1900	−3.2E−013	−4E−013	2.2165	2.147E−014	2.2105E−013	3.208E−013
1901	−2.9E−013	−4E−013	2.2165	1.419E−013	−2.714E−013	4.118E−014
1902	−6.7E−015	−2E−013	2.2165	8.030E−014	−1.610E−013	−1.0E−013
1903	−2.7E−013	−3E−013	2.2165	6.733E−014	1.8233E−013	2.575E−013
1904	2.97E−013	−3E−013	2.2165	3.075E−013	5.99E−013	8.906E−013
1905	−5.0E−013	−9E−013	2.2165	5.239E−013	1.2074E−012	1.507E−012
1906	−7.8E−013	−2E−012	2.2165	9.416E−013	2.2323E−012	2.623E−012
1907	−1.6E−012	−3E−012	2.2165	1.758E−012	3.4622E−012	3.749E−012
1908	−1.7E−012	−5E−012	2.2165	2.887E−012	5.623E−012	6.319E−012
1909	−4.5E−012	−8E−012	2.2165	4.859E−012	8.8925E−012	1.013E−011
1910	−5.4E−012	−2E−011	2.2165	8.005E−012	1.6447E−011	1.817E−011
1911	−1.2E−011	−2E−011	2.2165	1.403E−011	2.6897E−011	3.043E−011
1912	−1.5E−011	−4E−011	2.2165	2.312E−011	4.6939E−011	5.304E−011
1913	−3.4E−011	−6E−011	2.2165	4.053E−011	7.7059E−011	8.700E−011
1914	−4.6E−011	−1E−010	2.2165	6.693E−011	1.3501E−010	1.528E−010
1915	−9.4E−011	−2E−010	2.2165	1.169E−010	2.2235E−010	2.504E−010
1916	−1.4E−010	−4E−010	2.2165	1.926E−010	3.8865E−010	4.394E−010
1917	−2.6E−010	−5E−010	2.2165	3.369E−010	6.4145E−010	7.228E−010
1918	−4.1E−010	−1E−009	2.2165	5.545E−010	1.1195E−009	1.264E−009
1919	−7.2E−010	−1E−009	2.2165	9.721E−010	1.8531E−009	2.087E−009
1920	−1.2E−009	−3E−009	2.2165	1.595E−009	3.2232E−009	3.641E−009
1921	−2.0E−009	−4E−009	2.2165	2.805E−009	5.3466E−009	6.019E−009
1922	−3.8E−009	−9E−009	2.2165	4.589E−009	9.2862E−009	1.049E−008
1923	−5.3E−009	−1E−008	2.2165	8.101E−009	1.5424E−008	1.736E−008
1924	−1.2E−008	−3E−008	2.2165	1.319E−008	2.6767E−008	3.023E−008

1925	−1.4E−008	−3E−008	2.2165	2.340E−008	4.4488E−008	5.005E−008
1926	−3.5E−008	−8E−008	2.2165	3.791E−008	7.7195E−008	8.716E−008
1927	−3.7E−008	−9E−008	2.2165	6.764E−008	1.2828E−007	1.443E−007
1928	−1.1E−007	−2E−007	2.2165	1.088E−007	2.2279E−007	2.513E−007
1929	−9.3E−008	−3E−007	2.2165	1.956E−007	3.6978E−007	4.159E−007
1930	−3.4E−007	−7E−007	2.2165	3.122E−007	6.4362E−007	7.250E−007
1931	−2.2E−007	−7E−007	2.2165	5.661E−007	1.0656E−006	1.198E−006
1932	−1.1E−006	−2E−006	2.2165	8.949E−007	1.8618E−006	2.092E−006
1933	−4.6E−007	−2E−006	2.2165	1.639E−006	3.0702E−006	3.453E−006
1934	−3.4E−006	−6E−006	2.2165	2.562E−006	5.3963E−006	6.037E−006
1935	−7.1E−007	−5E−006	2.2165	4.748E−006	8.8455E−006	9.948E−006
1936	−1.1E−005	−2E−005	2.2164	7.323E−006	1.5688E−005	1.743E−005
1937	2.47E−007	−1E−005	2.2164	1.376E−005	2.55E−005	2.867E−005
1938	−3.6E−005	−6E−005	2.2162	2.09E−005	4.5831E−005	5.037E−005
1939	9.13E−006	−2E−005	2.2161	3.984E−005	7.3687E−005	8.271E−005
1940	−0.000121	−0.00019	2.2157	5.956E−005	0.00013508	0.00014592
1941	5.74E−005	−4E−005	2.2153	0.00011529	0.00021449	0.00023973
1942	−0.000413	−0.00061	2.2141	0.00016992	0.00040537	0.00042664
1943	0.00028079	−7E−006	2.2129	0.00033353	0.0006382	0.00070694
1944	−0.001467	−0.00204	2.2089	0.00049055	0.0012679	0.0012878
1945	0.0012625	0.0003362	2.2053	0.00096815	0.0020294	0.0022117
1946	−0.005728	−0.00743	2.1914	0.001504	0.0043544	0.0043194
1947	0.005954	0.0024288	2.1767	0.002803	0.0077935	0.0080758
1948	−0.028413	−0.03356	2.122	0.0064352	0.015209	0.020131
1949	0.031499	0.024142	2.0661	0.019639	0.017146	0.030482
1950	0.12476	0.14775	2.2317	0.00059656	−0.17378	−0.24358
1951	0.14775	0.12476	2.2317	0.00059656	0.24358	0.17378
1952	0.024142	0.031499	2.0661	0.019639	−0.030482	−0.017146
1953	−0.033561	−0.02841	2.122	0.0064352	−0.020131	−0.015209
1954	0.0024288	0.005954	2.1767	0.002803	−0.0080758	−0.0077935
1955	−0.007430	−0.00573	2.1914	0.001504	−0.0043194	−0.0043544
1956	0.00033624	0.0012625	2.2053	0.00096815	−0.0022117	−0.0020294
1957	−0.002043	−0.00147	2.2089	0.00049055	−0.0012878	−0.0012679
1958	−7.1E−006	0.0002808	2.2129	0.00033353	−0.00070694	−0.0006382
1959	−0.000611	−0.00041	2.2141	0.00016992	−0.00042664	−0.0004054
1960	−3.9E−005	5.7E−005	2.2153	0.00011529	−0.00023973	−0.0002145
1961	−0.000189	−0.00012	2.2157	5.956E−005	−0.00014592	−0.0001351
1962	−2.4E−005	9.1E−006	2.2161	3.984E−005	−8.271E−005	−7.4E−005
1963	−6.0E−005	−4E−005	2.2162	2.09E−005	−5.037E−005	−4.6E−005
1964	−1.1E−005	2.5E−007	2.2164	1.376E−005	−2.867E−005	−2.6E−005
1965	−1.9E−005	−1E−005	2.2164	7.323E−006	−1.743E−005	−1.6E−005
1966	−4.6E−006	−7E−007	2.2165	4.748E−006	−9.948E−006	−8.8E−006
1967	−6.2E−006	−3E−006	2.2165	2.562E−006	−6.037E−006	−5.4E−006
1968	−1.8E−006	−5E−007	2.2165	1.639E−006	−3.453E−006	−3.1E−006
1969	−2.1E−006	−1E−006	2.2165	8.949E−007	−2.092E−006	−1.9E−006
1970	−6.9E−007	−2E−007	2.2165	5.661E−007	−1.198E−006	−1.1E−006
1971	−6.8E−007	−3E−007	2.2165	3.122E−007	−7.250E−007	−6.4E−007

1972	−2.6E−007	−9E−008	2.2165	1.956E−007	−4.159E−007	−3.7E−007
1973	−2.3E−007	−1E−007	2.2165	1.088E−007	−2.513E−007	−2.2E−007
1974	−9.3E−008	−4E−008	2.2165	6.764E−008	−1.443E−007	−1.3E−007
1975	−7.7E−008	−4E−008	2.2165	3.791E−008	−8.716E−008	−7.7E−008
1976	−3.3E−008	−1E−008	2.2165	2.340E−008	−5.005E−008	−4.4E−008
1977	−2.6E−008	−1E−008	2.2165	1.319E−008	−3.023E−008	−2.7E−008
1978	−1.2E−008	−5E−009	2.2165	8.101E−009	−1.736E−008	−1.5E−008
1979	−8.9E−009	−4E−009	2.2165	4.589E−009	−1.049E−008	−9.3E−009
1980	−4.2E−009	−2E−009	2.2165	2.805E−009	−6.019E−009	−5.3E−009
1981	−3.0E−009	−1E−009	2.2165	1.595E−009	−3.641E−009	−3.2E−009
1982	−1.5E−009	−7E−010	2.2165	9.720E−010	−2.087E−009	−1.9E−009
1983	−1.0E−009	−4E−010	2.2165	5.544E−010	−1.264E−009	−1.1E−009
1984	−5.2E−010	−3E−010	2.2165	3.369E−010	−7.232E−010	−6.4E−010
1985	−3.6E−010	−1E−010	2.2165	1.926E−010	−4.390E−010	−3.9E−010
1986	−1.8E−010	−9E−011	2.2165	1.168E−010	−2.506E−010	−2.2E−010
1987	−1.2E−010	−5E−011	2.2165	6.684E−011	−1.526E−010	−1.4E−010
1988	−6.4E−011	−3E−011	2.2165	4.054E−011	−8.681E−011	−7.7E−011
1989	−4.3E−011	−1E−011	2.2165	2.322E−011	−5.297E−011	−4.7E−011
1990	−2.2E−011	−1E−011	2.2165	1.406E−011	−3.002E−011	−2.7E−011
1991	−1.5E−011	−5E−012	2.2165	7.997E−012	−1.850E−011	−1.6E−011
1992	−7.6E−012	−4E−012	2.2165	4.859E−012	−1.038E−011	−9.3E−012
1993	−5.2E−012	−2E−012	2.2165	2.862E−012	−6.266E−012	−5.7E−012
1994	−2.7E−012	−1E−012	2.2165	1.672E−012	−3.582E−012	−3.3E−012
1995	−1.7E−012	−5E−013	2.2165	9.525E−013	−2.178E−012	−2.0E−012
1996	−1.0E−012	−7E−013	2.2165	5.858E−013	−1.228E−012	−1.2E−012
1997	−4.6E−013	−1E−014	2.2165	2.996E−013	−7.927E−013	−6.7E−013
1998	−2.3E−013	−3E−014	2.2165	2.575E−013	−4.891E−013	−5.6E−013
1999	−3.6E−013	−2E−013	2.2165	1.237E−013	−5.168E−013	−4.0E−013
2000	−3.8E−013	−3E−013	2.2165	1.627E−015	−1.301E−013	−1.7E−013
2001	−1.1E−013	−7E−014	2.2165	−3.25E−014	−2.741E−013	6.764E−014
2002	−2.4E−013	−2E−013	2.2165	−4.03E−014	1.477E−013	9.186E−014
2003	2.11E−014	−4E−014	2.2165	−3.90E−014	8.3116E−014	−6.8E−014
2004	−1.1E−013	−6E−014	2.2165	−4.27E−014	2.2971E−013	2.857E−014
2005	1.44E−013	4.5E−014	2.2165	3.977E−014	4.2545E−014	−4.8E−014
2006	−2.2E−013	−2E−013	2.2165	−5.69E−014	1.3913E−013	2.579E−014
2007	−1.8E−013	−8E−014	2.2165	1.635E−014	−1.190E−013	−4.6E−014
2008	1.35E−013	−1E−014	2.2165	8.387E−014	9.1384E−014	−1.1E−013
2009	−2.9E−013	−3E−013	2.2165	−2.03E−014	−1.873E−014	−2.4E−013
2010	2.50E−014	5.1E−014	2.2165	−1.55E−014	9.5532E−014	−1.8E−013
2011	−1.3E−013	−2E−013	2.2165	−2.04E−014	−1.286E−013	−3.8E−014
2012	7.06E−014	1.5E−013	2.2165	−4.02E−014	3.8673E−014	−6.0E−015
2013	−2.9E−013	−4E−013	2.2165	4.784E−014	2.5187E−014	2.469E−013
2014	−1.9E−013	−7E−014	2.2165	3.302E−014	−1.376E−014	−1.6E−013
2015	−3.6E−013	−3E−013	2.2165	−4.55E−014	−5.594E−014	−5.6E−014
2016	−8.7E−014	−2E−013	2.2165	−1.08E−013	8.234E−014	−7.1E−014
2017	−2.1E−013	−2E−013	2.2165	2.282E−014	−1.081E−013	8.003E−015
2018	7.38E−014	−8E−014	2.2165	−3.42E−014	1.5822E−013	−2.3E−013

2019	1.95E−014	−5E−014	2.2165	−1.12E−013	1.4967E−013	3.588E−014
2020	−3.2E−013	−2E−013	2.2165	−4.83E−014	−3.800E−014	−7.6E−014
2021	−4.3E−013	−4E−013	2.2165	−5.90E−014	2.029E−013	−1.4E−013
2022	−8.7E−015	−1E−013	2.2165	1.107E−014	−4.074E−014	3.505E−013
2023	−1.3E−013	−1E−013	2.2165	3.343E−014	3.2795E−013	−2.0E−013
2024	−1.6E−013	−3E−013	2.2165	4.199E−014	7.1205E−014	9.387E−015
2025	1.52E−013	3.4E−013	2.2165	−2.93E−014	−9.243E−014	−1.9E−014
2026	−4.6E−013	−6E−013	2.2165	−1.27E−013	−2.008E−013	−5.1E−014
2027	1.43E−014	−5E−014	2.2165	−1.23E−014	2.6082E−013	1.874E−013
2028	2.38E−013	5.0E−013	2.2165	9.004E−015	2.0425E−014	−5.9E−014
2029	−4.5E−013	−4E−013	2.2165	−1.03E−013	−1.011E−013	5.894E−014
2030	−1.7E−013	−3E−013	2.2165	−4.94E−014	−5.044E−014	−5.0E−015
2031	−1.8E−013	−4E−013	2.2165	−1.60E−013	4.764E−014	−1.4E−013
2032	8.13E−015	−1E−014	2.2165	−4.16E−014	3.8704E−013	−2.2E−013
2033	−3.4E−013	−3E−013	2.2165	−1.06E−013	2.6761E−013	3.225E−013
2034	1.60E−013	7.9E−014	2.2165	−5.42E−014	2.3458E−013	−7.5E−014
2035	5.58E−013	4.9E−013	2.2165	1.358E−014	−1.910E−013	1.206E−013
2036	2.28E−015	−3E−014	2.2165	9.702E−014	2.8955E−013	−5.7E−014
2037	−3.3E−013	−6E−014	2.2165	1.289E−013	4.4289E−014	−8.4E−015
2038	−3.1E−013	−2E−013	2.2165	6.844E−014	4.8243E−013	6.095E−013
2039	−1.3E−013	−2E−013	2.2165	8.146E−014	8.4206E−014	3.638E−013
2040	−7.1E−013	−5E−013	2.2165	1.604E−013	3.9419E−013	8.676E−014
2041	−3.2E−013	−2E−014	2.2165	2.346E−013	4.3102E−013	5.723E−013
2042	−1.2E−012	−8E−013	2.2165	5.121E−013	1.1527E−012	8.792E−013
2043	−1.9E−012	−5E−013	2.2165	8.895E−013	2.1285E−012	2.197E−012
2044	−2.8E−012	−2E−012	2.2165	1.737E−012	3.7586E−012	3.665E−012
2045	−5.4E−012	−2E−012	2.2165	2.836E−012	6.4198E−012	5.296E−012
2046	−7.6E−012	−4E−012	2.2165	4.876E−012	1.0318E−011	9.234E−012
2047	−1.5E−011	−5E−012	2.2165	7.974E−012	1.8148E−011	1.639E−011
2048	−2.2E−011	−1E−011	2.2165	1.414E−011	2.971E−011	2.669E−011
2049	−4.2E−011	−1E−011	2.2165	2.304E−011	5.3098E−011	4.703E−011
2050	−6.4E−011	−3E−011	2.2165	4.056E−011	8.6498E−011	7.749E−011
2051	−1.2E−010	−5E−011	2.2165	6.682E−011	1.5219E−010	1.351E−010
2052	−1.8E−010	−9E−011	2.2165	1.168E−010	2.5067E−010	2.226E−010
2053	−3.6E−010	−1E−010	2.2165	1.928E−010	4.3875E−010	3.884E−010
2054	−5.2E−010	−3E−010	2.2165	3.368E−010	7.2313E−010	6.419E−010
2055	−1.0E−009	−4E−010	2.2165	5.544E−010	1.2645E−009	1.119E−009
2056	−1.5E−009	−7E−010	2.2165	9.720E−010	2.0864E−009	1.853E−009
2057	−3.0E−009	−1E−009	2.2165	1.595E−009	3.6418E−009	3.223E−009
2058	−4.2E−009	−2E−009	2.2165	2.805E−009	6.0182E−009	5.346E−009
2059	−8.9E−009	−4E−009	2.2165	4.589E−009	1.0491E−008	9.286E−009
2060	−1.2E−008	−5E−009	2.2165	8.101E−009	1.7357E−008	1.542E−008
2061	−2.6E−008	−1E−008	2.2165	1.319E−008	3.0235E−008	2.677E−008
2062	−3.3E−008	−1E−008	2.2165	2.340E−008	5.0048E−008	4.449E−008
2063	−7.7E−008	−4E−008	2.2165	3.791E−008	8.7162E−008	7.719E−008
2064	−9.3E−008	−4E−008	2.2165	6.764E−008	1.4428E−007	1.283E−007
2065	−2.3E−007	−1E−007	2.2165	1.088E−007	2.5134E−007	2.228E−007

2066	−2.6E−007	−9E−008	2.2165	1.956E−007	4.1585E−007	3.698E−007
2067	−6.8E−007	−3E−007	2.2165	3.122E−007	7.2498E−007	6.436E−007
2068	−6.9E−007	−2E−007	2.2165	5.661E−007	1.1984E−006	1.066E−006
2069	−2.1E−006	−1E−006	2.2165	8.949E−007	2.0918E−006	1.862E−006
2070	−1.8E−006	−5E−007	2.2165	1.639E−006	3.453E−006	3.070E−006
2071	−6.2E−006	−3E−006	2.2165	2.562E−006	6.0371E−006	5.396E−006
2072	−4.6E−006	−7E−007	2.2165	4.748E−006	9.9483E−006	8.846E−006
2073	−1.9E−005	−1E−005	2.2164	7.323E−006	1.7431E−005	1.569E−005
2074	−1.1E−005	2.5E−007	2.2164	1.376E−005	2.8667E−005	2.55E−005
2075	−6.0E−005	−4E−005	2.2162	2.09E−005	5.0368E−005	4.583E−005
2076	−2.4E−005	9.1E−006	2.2161	3.984E−005	8.2705E−005	7.369E−005
2077	−0.000189	−0.00012	2.2157	5.956E−005	0.00014592	0.00013508
2078	−3.9E−005	5.7E−005	2.2153	0.00011529	0.00023973	0.00021449
2079	−0.000611	−0.00041	2.2141	0.00016992	0.00042664	0.00040537
2080	−7.1E−006	0.0002808	2.2129	0.00033353	0.00070694	0.0006382
2081	−0.002043	−0.00147	2.2089	0.00049055	0.0012878	0.0012679
2082	0.00033624	0.0012625	2.2053	0.00096815	0.0022117	0.0020294
2083	−0.007430	−0.00573	2.1914	0.001504	0.0043194	0.0043544
2084	0.0024288	0.005954	2.1767	0.002803	0.0080758	0.0077935
2085	−0.033561	−0.02841	2.122	0.0064352	0.020131	0.015209
2086	0.024142	0.031499	2.0661	0.019639	0.030482	0.017146
2087	0.14775	0.12476	2.2317	0.00059656	−0.24358	−0.17378
2088	0.056051	−0.02616	2.2586	−0.0026513	0.29655	0.09443
2089	0.043625	0.080882	2.0092	0.013804	−0.051928	−0.0076766
2090	−0.048546	−0.03780	2.1255	0.0015614	−0.012956	−0.012957
2091	0.0028793	0.012779	2.1793	0.0012632	−0.0031784	−0.0032786
2092	−0.009690	−0.00629	2.1967	0.0005068	−0.0029059	−0.0024157
2093	−0.000557	0.0020145	2.2055	0.00032964	−0.00086209	−0.0007619
2094	−0.002468	−0.00129	2.2102	0.00015287	−0.00084654	−0.0006389
2095	−0.000402	0.0003932	2.2128	0.00010459	−0.00030191	−0.0002349
2096	−0.000730	−0.00032	2.2144	5.158E−005	−0.00027006	−0.0001890
2097	−0.000180	8.4E−005	2.2153	3.503E−005	−0.00011186	−8.0E−005
2098	−0.000230	−9E−005	2.2158	1.798E−005	−8.902E−005	−5.9E−005
2099	−7.2E−005	1.8E−005	2.2161	1.195E−005	−4.123E−005	−2.8E−005
2100	−7.5E−005	−2E−005	2.2163	6.300E−006	−2.977E−005	−1.9E−005
2101	−2.7E−005	3.5E−006	2.2164	4.106E−006	−1.498E−005	−1.0E−005
2102	−2.5E−005	−7E−006	2.2164	2.206E−006	−1.004E−005	−6.4E−006
2103	−1.0E−005	5.3E−007	2.2165	1.416E−006	−5.380E−006	−3.5E−006
2104	−8.2E−006	−2E−006	2.2165	7.704E−007	−3.4E−006	−2.1E−006
2105	−3.7E−006	1.4E−008	2.2165	4.891E−007	−1.916E−006	−1.2E−006
2106	−2.8E−006	−7E−007	2.2165	2.685E−007	−1.156E−006	−7.2E−007
2107	−1.3E−006	−4E−008	2.2165	1.693E−007	−6.786E−007	−4.4E−007
2108	−9.3E−007	−2E−007	2.2165	9.339E−008	−3.942E−007	−2.5E−007
2109	−4.7E−007	−2E−008	2.2165	5.867E−008	−2.394E−007	−1.5E−007
2110	−3.2E−007	−7E−008	2.2165	3.243E−008	−1.346E−007	−8.4E−008
2111	−1.7E−007	−1E−008	2.2165	2.036E−008	−8.431E−008	−5.4E−008
2112	−1.1E−007	−2E−008	2.2165	1.125E−008	−4.603E−008	−2.9E−008

2113	−5.8E−008	−4E−009	2.2165	7.071E−009	−2.964E−008	−1.9E−008
2114	−3.7E−008	−8E−009	2.2165	3.899E−009	−1.575E−008	−9.9E−009
2115	−2.0E−008	−2E−009	2.2165	2.458E−009	−1.042E−008	−6.6E−009
2116	−1.3E−008	−3E−009	2.2165	1.350E−009	−5.386E−009	−3.4E−009
2117	−7.2E−009	−6E−010	2.2165	8.554E−010	−3.659E−009	−2.3E−009
2118	−4.4E−009	−9E−010	2.2165	4.668E−010	−1.842E−009	−1.2E−009
2119	−2.5E−009	−2E−010	2.2165	2.978E−010	−1.286E−009	−8.0E−010
2120	−1.5E−009	−3E−010	2.2165	1.613E−010	−6.290E−010	−4.0E−010
2121	−8.7E−010	−7E−011	2.2165	1.038E−010	−4.523E−010	−2.8E−010
2122	−5.3E−010	−1E−010	2.2165	5.565E−011	−2.145E−010	−1.4E−010
2123	−3.0E−010	−2E−011	2.2165	3.619E−011	−1.594E−010	−9.9E−011
2124	−1.8E−010	−4E−011	2.2165	1.914E−011	−7.315E−011	−4.7E−011
2125	−1.1E−010	−7E−012	2.2165	1.264E−011	−5.592E−011	−3.5E−011
2126	−6.3E−011	−1E−011	2.2165	6.623E−012	−2.471E−011	−1.6E−011
2127	−3.7E−011	−2E−012	2.2165	4.422E−012	−1.982E−011	−1.2E−011
2128	−2.2E−011	−5E−012	2.2165	2.288E−012	−8.428E−012	−5.6E−012
2129	−1.3E−011	−8E−013	2.2165	1.501E−012	−6.838E−012	−4.3E−012
2130	−7.6E−012	−2E−012	2.2165	8.054E−013	−2.915E−012	−2.0E−012
2131	−4.6E−012	−3E−013	2.2165	5.165E−013	−2.499E−012	−1.5E−012
2132	−2.7E−012	−8E−013	2.2165	3.048E−013	−9.746E−013	−6.7E−013
2133	−1.5E−012	−1E−013	2.2165	1.432E−013	−9.252E−013	−6.7E−013
2134	−9.3E−013	−2E−013	2.2165	8.358E−014	−3.767E−013	−2.6E−013
2135	−6.0E−013	−3E−014	2.2165	5.636E−014	−3.076E−013	−2.6E−014
2136	−3.8E−013	−2E−013	2.2165	3.905E−014	−1.957E−013	4.132E−015
2137	1.68E−013	3.7E−013	2.2165	3.072E−014	−2.042E−013	8.081E−014
2138	8.74E−014	8.6E−014	2.2165	2.899E−014	−1.891E−013	−1.1E−014
2139	−2.4E−014	−2E−013	2.2165	−1.20E−014	−1.491E−013	−9.9E−014
2140	2.85E−014	−1E−013	2.2165	2.016E−014	3.2905E−013	−9.3E−014
2141	−2.4E−013	−2E−013	2.2165	−3.93E−014	−7.124E−014	−1.1E−013
2142	−2.1E−013	−2E−013	2.2165	−3.23E−014	−2.965E−013	−1.3E−013
2143	−2.5E−013	−9E−014	2.2165	−7.91E−014	1.6634E−013	−3.6E−015
2144	−3.6E−013	−2E−013	2.2165	−1.78E−015	−2.933E−013	9.957E−014
2145	7.75E−014	−5E−014	2.2165	−3.86E−014	3.4406E−014	−9.2E−014
2146	−7.8E−014	3.9E−014	2.2165	−3.28E−014	1.7398E−013	4.637E−014
2147	−1.5E−013	−1E−013	2.2165	−4.75E−014	1.9087E−013	7.127E−014
2148	−3.4E−013	−4E−013	2.2165	1.387E−014	3.8145E−014	9.207E−014
2149	2.44E−013	8.5E−014	2.2165	−6.74E−015	−8.692E−014	2.692E−014
2150	2.14E−014	2.8E−014	2.2165	−3.88E−014	−1.502E−013	−6.9E−014
2151	2.83E−013	2.0E−013	2.2165	−4.82E−014	9.7114E−014	8.859E−014
2152	2.14E−013	8.0E−014	2.2165	−1.12E−014	−1.913E−013	−2.3E−013
2153	−2.4E−013	−2E−013	2.2165	−3.53E−014	1.1126E−013	1.093E−013
2154	−1.0E−015	−9E−014	2.2165	−2.36E−014	2.6328E−013	1.717E−013
2155	1.24E−013	−8E−015	2.2165	3.559E−014	−4.108E−013	−8.7E−014
2156	1.29E−013	1.3E−013	2.2165	1.859E−014	1.0808E−013	−1.1E−013
2157	2.64E−013	1.4E−013	2.2165	1.798E−014	−8.159E−014	1.846E−013
2158	−1.5E−013	−1E−013	2.2165	3.021E−015	2.0424E−013	3.053E−013
2159	1.51E−013	4.5E−014	2.2165	2.512E−014	−5.304E−015	−3.2E−014

2160	−5.8E−014	−2E−014	2.2165	5.262E−014	−5.743E−013	1.699E−013
2161	1.72E−015	−3E−014	2.2165	6.145E−014	−2.013E−013	−1.9E−013
2162	4.19E−013	2.2E−013	2.2165	−3.06E−014	−3.532E−014	−2.9E−013
2163	4.12E−014	1.2E−013	2.2165	−6.56E−014	−3.727E−013	−4.5E−013
2164	2.10E−013	−4E−014	2.2165	−1.04E−013	1.9067E−013	−2.3E−013
2165	2.56E−013	4.1E−013	2.2165	−8.60E−014	2.5532E−013	−9.3E−015
2166	−7.6E−014	8.7E−014	2.2165	−5.68E−014	−2.648E−013	5.216E−014
2167	1.32E−013	−1E−013	2.2165	−2.51E−014	1.0331E−013	−5.9E−015
2168	2.17E−013	6.8E−014	2.2165	−1.40E−014	1.1373E−013	1.128E−013
2169	3.91E−013	6.7E−014	2.2165	−1.19E−014	5.0012E−013	−1.4E−013
2170	1.34E−013	1.1E−013	2.2165	5.154E−014	−3.798E−013	2.957E−016
2171	2.71E−013	1.8E−013	2.2165	3.637E−014	1.4813E−013	1.275E−013
2172	7.31E−014	−8E−015	2.2165	−3.39E−014	4.7063E−014	2.667E−013
2173	−4.5E−013	−2E−013	2.2165	9.086E−014	5.4266E−013	3.986E−013
2174	1.38E−013	2.0E−013	2.2165	9.858E−014	−5.695E−014	−3.0E−014
2175	−1.8E−013	−5E−014	2.2165	2.456E−014	−2.635E−013	6.166E−014
2176	−7.2E−013	−6E−013	2.2165	−5.01E−014	2.9529E−013	1.309E−014
2177	−7.1E−013	−3E−013	2.2165	1.183E−013	8.1016E−013	2.476E−013
2178	−6.1E−013	−7E−014	2.2165	7.696E−014	1.9338E−013	1.086E−013
2179	−1.3E−012	1.8E−013	2.2165	1.736E−013	5.1402E−013	6.485E−013
2180	−2.7E−012	−7E−013	2.2165	3.339E−013	1.0201E−012	6.733E−013
2181	−4.4E−012	−8E−014	2.2165	4.414E−013	2.6816E−012	1.416E−012
2182	−7.8E−012	−2E−012	2.2165	8.433E−013	2.5894E−012	1.869E−012
2183	−1.3E−011	−7E−013	2.2165	1.627E−012	7.314E−012	4.413E−012
2184	−2.2E−011	−5E−012	2.2165	2.298E−012	7.9599E−012	5.709E−012
2185	−3.7E−011	−3E−012	2.2165	4.408E−012	1.9956E−011	1.209E−011
2186	−6.3E−011	−1E−011	2.2165	6.652E−012	2.4913E−011	1.573E−011
2187	−1.1E−010	−7E−012	2.2165	1.266E−011	5.5781E−011	3.499E−011
2188	−1.8E−010	−4E−011	2.2165	1.910E−011	7.3246E−011	4.722E−011
2189	−3.0E−010	−2E−011	2.2165	3.623E−011	1.5916E−010	9.965E−011
2190	−5.3E−010	−1E−010	2.2165	5.575E−011	2.1463E−010	1.368E−010
2191	−8.7E−010	−7E−011	2.2165	1.038E−010	4.5197E−010	2.818E−010
2192	−1.5E−009	−3E−010	2.2165	1.614E−010	6.2852E−010	3.990E−010
2193	−2.5E−009	−2E−010	2.2165	2.978E−010	1.2862E−009	8.037E−010
2194	−4.4E−009	−9E−010	2.2165	4.667E−010	1.8413E−009	1.164E−009
2195	−7.2E−009	−6E−010	2.2165	8.555E−010	3.6599E−009	2.296E−009
2196	−1.3E−008	−3E−009	2.2165	1.350E−009	5.3857E−009	3.393E−009
2197	−2.0E−008	−2E−009	2.2165	2.458E−009	1.0416E−008	6.559E−009
2198	−3.7E−008	−8E−009	2.2165	3.899E−009	1.5745E−008	9.887E−009
2199	−5.8E−008	−4E−009	2.2165	7.071E−009	2.9643E−008	1.874E−008
2200	−1.1E−007	−2E−008	2.2165	1.125E−008	4.6029E−008	2.883E−008
2201	−1.7E−007	−1E−008	2.2165	2.036E−008	8.4305E−008	5.355E−008
2202	−3.2E−007	−7E−008	2.2165	3.243E−008	1.3463E−007	8.417E−008
2203	−4.7E−007	−2E−008	2.2165	5.867E−008	2.3945E−007	1.529E−007
2204	−9.3E−007	−2E−007	2.2165	9.339E−008	3.9417E−007	2.463E−007
2205	−1.3E−006	−4E−008	2.2165	1.693E−007	6.7855E−007	4.359E−007
2206	−2.8E−006	−7E−007	2.2165	2.685E−007	1.1562E−006	7.233E−007

2207	−3.7E−006	1.4E−008	2.2165	4.891E−007	1.9159E−006	1.241E−006
2208	−8.2E−006	−2E−006	2.2165	7.704E−007	3.4E−006	2.137E−006
2209	−1.0E−005	5.3E−007	2.2165	1.416E−006	5.3797E−006	3.522E−006
2210	−2.5E−005	−7E−006	2.2164	2.206E−006	1.0035E−005	6.367E−006
2211	−2.7E−005	3.5E−006	2.2164	4.106E−006	1.4981E−005	9.971E−006
2212	−7.5E−005	−2E−005	2.2163	6.300E−006	2.9771E−005	1.923E−005
2213	−7.2E−005	1.8E−005	2.2161	1.195E−005	4.123E−005	2.82E−005
2214	−0.000230	−9E−005	2.2158	1.798E−005	8.902E−005	5.929E−005
2215	−0.000180	8.4E−005	2.2153	3.503E−005	0.00011186	8.021E−005
2216	−0.000730	−0.00032	2.2144	5.158E−005	0.00027006	0.00018902
2217	−0.000402	0.0003932	2.2128	0.00010459	0.00030191	0.00023494
2218	−0.002468	−0.00129	2.2102	0.00015287	0.00084654	0.00063891
2219	−0.000557	0.0020145	2.2055	0.00032964	0.00086209	0.00076194
2220	−0.009690	−0.00629	2.1967	0.0005068	0.0029059	0.0024157
2221	0.0028793	0.012779	2.1793	0.0012632	0.0031784	0.0032786
2222	−0.048546	−0.03780	2.1255	0.0015614	0.012956	0.012957
2223	0.043625	0.080882	2.0092	0.013804	0.051928	0.0076766
2224	0.056051	−0.02616	2.2586	−0.0026513	−0.29655	−0.09443
2225	2.2088	2.2543	2.5136	−0.0050237	0.64668	−0.14985
2226	1.9808	1.9985	2.2709	−0.01887	0.49479	−0.33185
2227	1.9985	1.9808	2.2709	−0.01887	0.33185	−0.49479
2228	2.2543	2.2088	2.5136	−0.0050237	0.14985	−0.64668
2229	2.2088	2.2543	2.5136	−0.0050237	−0.64668	0.14985
2230	1.9808	1.9985	2.2709	−0.01887	−0.49479	0.33185
2231	1.9985	1.9808	2.2709	−0.01887	−0.33185	0.49479
2232	2.2543	2.2088	2.5136	−0.0050237	−0.14985	0.64668
2233	0.056051	−0.02616	2.2586	0.0026513	0.29655	−0.09443
2234	0.043625	0.080882	2.0092	−0.013804	−0.051928	0.0076766
2235	−0.048546	−0.03780	2.1255	−0.0015614	−0.012956	0.012957
2236	0.0028793	0.012779	2.1793	−0.0012632	−0.0031784	0.0032786
2237	−0.009690	−0.00629	2.1967	−0.0005068	−0.0029059	0.0024157
2238	−0.000557	0.0020145	2.2055	−0.0003296	−0.00086209	0.00076194
2239	−0.002468	−0.00129	2.2102	−0.0001529	−0.00084654	0.00063891
2240	−0.000402	0.0003932	2.2128	−0.0001046	−0.00030191	0.00023494
2241	−0.000730	−0.00032	2.2144	−5.16E−005	−0.00027006	0.00018902
2242	−0.000180	8.4E−005	2.2153	−3.50E−005	−0.00011186	8.021E−005
2243	−0.000230	−9E−005	2.2158	−1.80E−005	−8.902E−005	5.929E−005
2244	−7.2E−005	1.8E−005	2.2161	−1.20E−005	−4.123E−005	2.82E−005
2245	−7.5E−005	−2E−005	2.2163	−6.30E−006	−2.977E−005	1.923E−005
2246	−2.7E−005	3.5E−006	2.2164	−4.11E−006	−1.498E−005	9.971E−006
2247	−2.5E−005	−7E−006	2.2164	−2.21E−006	−1.004E−005	6.367E−006
2248	−1.0E−005	5.3E−007	2.2165	−1.42E−006	−5.380E−006	3.522E−006
2249	−8.2E−006	−2E−006	2.2165	−7.70E−007	−3.4E−006	2.137E−006
2250	−3.7E−006	1.4E−008	2.2165	−4.89E−007	−1.916E−006	1.241E−006
2251	−2.8E−006	−7E−007	2.2165	−2.68E−007	−1.156E−006	7.233E−007
2252	−1.3E−006	−4E−008	2.2165	−1.69E−007	−6.786E−007	4.359E−007
2253	−9.3E−007	−2E−007	2.2165	−9.34E−008	−3.942E−007	2.463E−007

2254	−4.7E−007	−2E−008	2.2165	−5.87E−008	−2.394E−007	1.529E−007
2255	−3.2E−007	−7E−008	2.2165	−3.24E−008	−1.346E−007	8.417E−008
2256	−1.7E−007	−1E−008	2.2165	−2.04E−008	−8.431E−008	5.355E−008
2257	−1.1E−007	−2E−008	2.2165	−1.13E−008	−4.603E−008	2.883E−008
2258	−5.8E−008	−4E−009	2.2165	−7.07E−009	−2.964E−008	1.874E−008
2259	−3.7E−008	−8E−009	2.2165	−3.90E−009	−1.575E−008	9.887E−009
2260	−2.0E−008	−2E−009	2.2165	−2.46E−009	−1.042E−008	6.559E−009
2261	−1.3E−008	−3E−009	2.2165	−1.35E−009	−5.386E−009	3.392E−009
2262	−7.2E−009	−6E−010	2.2165	−8.55E−010	−3.660E−009	2.296E−009
2263	−4.4E−009	−9E−010	2.2165	−4.67E−010	−1.841E−009	1.164E−009
2264	−2.5E−009	−2E−010	2.2165	−2.98E−010	−1.286E−009	8.037E−010
2265	−1.5E−009	−3E−010	2.2165	−1.61E−010	−6.291E−010	3.990E−010
2266	−8.7E−010	−7E−011	2.2165	−1.04E−010	−4.523E−010	2.817E−010
2267	−5.3E−010	−1E−010	2.2165	−5.57E−011	−2.147E−010	1.365E−010
2268	−3.0E−010	−2E−011	2.2165	−3.62E−011	−1.594E−010	9.889E−011
2269	−1.8E−010	−4E−011	2.2165	−1.92E−011	−7.321E−011	4.682E−011
2270	−1.1E−010	−7E−012	2.2165	−1.26E−011	−5.576E−011	3.456E−011
2271	−6.3E−011	−1E−011	2.2165	−6.63E−012	−2.482E−011	1.603E−011
2272	−3.7E−011	−2E−012	2.2165	−4.44E−012	−1.972E−011	1.214E−011
2273	−2.2E−011	−5E−012	2.2165	−2.28E−012	−8.392E−012	5.574E−012
2274	−1.3E−011	−8E−013	2.2165	−1.54E−012	−6.799E−012	4.451E−012
2275	−7.6E−012	−2E−012	2.2165	−7.34E−013	−2.623E−012	1.883E−012
2276	−4.7E−012	−5E−013	2.2165	−5.28E−013	−2.472E−012	1.523E−012
2277	−2.8E−012	−9E−013	2.2165	−2.05E−013	−9.741E−013	5.009E−013
2278	−1.8E−012	−4E−013	2.2165	−2.22E−013	−1.095E−012	4.955E−013
2279	−9.0E−013	−3E−013	2.2165	−1.00E−013	−2.853E−013	1.386E−013
2280	−7.1E−013	−2E−013	2.2165	−3.67E−014	−4.4E−013	5.111E−014
2281	−2.5E−013	−2E−013	2.2165	−1.09E−014	−2.152E−013	−7.9E−015
2282	−1.3E−013	1.3E−013	2.2165	−5.41E−014	1.8513E−013	−7.9E−015
2283	−3.0E−013	−3E−013	2.2165	1.801E−014	−1.231E−013	−3.3E−014
2284	−1.9E−013	−2E−013	2.2165	2.739E−015	1.0296E−013	2.214E−013
2285	−8.4E−014	−2E−014	2.2165	4.076E−014	−1.407E−013	1.036E−013
2286	2.62E−014	−7E−014	2.2165	5.310E−014	−1.494E−013	−8.8E−014
2287	8.63E−015	−1E−013	2.2165	1.640E−014	2.9151E−013	3.045E−014
2288	−2.1E−015	2E−013	2.2165	3.290E−014	−1.798E−013	−2.1E−013
2289	−4.8E−013	−5E−013	2.2165	5.451E−014	1.2483E−013	1.471E−014
2290	−9.8E−014	−2E−013	2.2165	−1.13E−014	1.4586E−014	−5.6E−014
2291	−1.1E−013	−2E−013	2.2165	3.181E−014	−2.638E−013	7.352E−014
2292	−1.1E−014	−3E−014	2.2165	1.749E−014	−1.237E−013	1.132E−013
2293	−1.2E−014	3.1E−016	2.2165	5.308E−014	5.2089E−013	−1.1E−014
2294	−1.6E−013	−2E−013	2.2165	3.535E−014	−3.388E−013	−1.9E−013
2295	−1.1E−013	−2E−013	2.2165	−8.81E−015	3.7427E−014	−7.3E−014
2296	−1.4E−013	−9E−014	2.2165	1.142E−013	1.9668E−014	−1.7E−013
2297	−9.8E−014	−1E−013	2.2165	−3.40E−015	−2.214E−013	−1.8E−013
2298	−2.3E−013	−2E−013	2.2165	2.052E−014	−1.031E−013	−2.6E−013
2299	−3.6E−013	−3E−013	2.2165	−2.25E−014	3.599E−013	−5.7E−014
2300	−1.2E−013	−1E−013	2.2165	−6.04E−014	1.7959E−014	−1.3E−013

2301	2.28E−013	1.5E−013	2.2165	−2.94E−014	−2.454E−015	5.404E−014
2302	−3.5E−014	−3E−014	2.2165	2.193E−014	−3.308E−013	3.694E−013
2303	1.55E−013	1.2E−013	2.2165	−5.76E−014	3.1759E−013	1.644E−013
2304	3.21E−013	2.7E−013	2.2165	−3.31E−014	−3.546E−013	2.011E−013
2305	−9.7E−014	−2E−013	2.2165	4.849E−014	9.7048E−014	−1.1E−013
2306	1.67E−013	1.8E−013	2.2165	3.282E−015	−4.503E−013	2.016E−013
2307	−3.4E−014	−7E−014	2.2165	2.003E−014	−1.793E−015	−5.0E−014
2308	−4.1E−015	3.8E−014	2.2165	3.382E−014	−8.558E−014	1.190E−013
2309	3.82E−013	3.0E−013	2.2165	1.542E−013	1.1567E−013	−1.8E−013
2310	3.66E−013	3.1E−013	2.2165	−1.74E−014	−2.852E−013	−9.9E−014
2311	1.10E−013	1.2E−013	2.2165	7.704E−014	1.0861E−013	−2.2E−013
2312	3.91E−015	−1E−013	2.2165	−8.63E−015	−6.604E−014	7.022E−014
2313	2.15E−013	1.1E−013	2.2165	−2.50E−014	4.9258E−013	3.412E−014
2314	6.07E−014	2.2E−014	2.2165	−7.11E−014	−9.089E−014	3.452E−013
2315	−2.8E−013	−1E−013	2.2165	−4.01E−014	8.998E−014	2.383E−013
2316	−3.0E−014	−1E−014	2.2165	−7.77E−015	5.6418E−013	3.075E−013
2317	1.63E−014	−2E−014	2.2165	−4.09E−014	1.6147E−013	1.070E−013
2318	1.32E−013	2.0E−013	2.2165	−2.19E−014	1.0419E−013	−7.4E−014
2319	−2.7E−013	−9E−015	2.2165	5.375E−014	2.0108E−014	1.891E−014
2320	−3.0E−013	5.9E−014	2.2165	9.732E−014	−8.221E−014	−5.3E−013
2321	−2.3E−013	−2E−013	2.2165	−6.54E−014	−1.385E−013	−4.2E−013
2322	−4.4E−013	−2E−013	2.2165	−1.09E−013	7.9706E−013	−4.6E−013
2323	−1.0E−012	−2E−013	2.2165	−1.81E−013	4.226E−013	−1.8E−014
2324	−1.1E−012	3.3E−013	2.2165	−1.54E−013	7.2584E−013	−4.3E−013
2325	−2.5E−012	−5E−013	2.2165	−1.78E−013	8.7835E−013	−5.4E−013
2326	−3.8E−012	2.6E−013	2.2165	−6.61E−013	3.0014E−012	−1.4E−012
2327	−7.7E−012	−2E−012	2.2165	−7.48E−013	2.2081E−012	−1.8E−012
2328	−1.3E−011	−9E−013	2.2165	−1.56E−012	6.5931E−012	−4.3E−012
2329	−2.2E−011	−5E−012	2.2165	−2.38E−012	9.0353E−012	−5.5E−012
2330	−3.7E−011	−2E−012	2.2165	−4.41E−012	1.9864E−011	−1.2E−011
2331	−6.4E−011	−1E−011	2.2165	−6.54E−012	2.4315E−011	−1.6E−011
2332	−1.1E−010	−7E−012	2.2165	−1.27E−011	5.681E−011	−3.5E−011
2333	−1.8E−010	−4E−011	2.2165	−1.93E−011	7.2842E−011	−4.7E−011
2334	−3.0E−010	−2E−011	2.2165	−3.62E−011	1.5961E−010	−9.9E−011
2335	−5.3E−010	−1E−010	2.2165	−5.57E−011	2.142E−010	−1.4E−010
2336	−8.7E−010	−7E−011	2.2165	−1.04E−010	4.5206E−010	−2.8E−010
2337	−1.5E−009	−3E−010	2.2165	−1.61E−010	6.2887E−010	−4.0E−010
2338	−2.5E−009	−2E−010	2.2165	−2.98E−010	1.2855E−009	−8.0E−010
2339	−4.4E−009	−9E−010	2.2165	−4.67E−010	1.8423E−009	−1.2E−009
2340	−7.2E−009	−6E−010	2.2165	−8.55E−010	3.6597E−009	−2.3E−009
2341	−1.3E−008	−3E−009	2.2165	−1.35E−009	5.3867E−009	−3.4E−009
2342	−2.0E−008	−2E−009	2.2165	−2.46E−009	1.0415E−008	−6.6E−009
2343	−3.7E−008	−8E−009	2.2165	−3.90E−009	1.5746E−008	−9.9E−009
2344	−5.8E−008	−4E−009	2.2165	−7.07E−009	2.9642E−008	−1.9E−008
2345	−1.1E−007	−2E−008	2.2165	−1.13E−008	4.6031E−008	−2.9E−008
2346	−1.7E−007	−1E−008	2.2165	−2.04E−008	8.4305E−008	−5.4E−008
2347	−3.2E−007	−7E−008	2.2165	−3.24E−008	1.3463E−007	−8.4E−008

2348	−4.7E−007	−2E−008	2.2165	−5.87E−008	2.3945E−007	−1.5E−007
2349	−9.3E−007	−2E−007	2.2165	−9.34E−008	3.9417E−007	−2.5E−007
2350	−1.3E−006	−4E−008	2.2165	−1.69E−007	6.7855E−007	−4.4E−007
2351	−2.8E−006	−7E−007	2.2165	−2.68E−007	1.1562E−006	−7.2E−007
2352	−3.7E−006	1.4E−008	2.2165	−4.89E−007	1.9159E−006	−1.2E−006
2353	−8.2E−006	−2E−006	2.2165	−7.70E−007	3.4E−006	−2.1E−006
2354	−1.0E−005	5.3E−007	2.2165	−1.42E−006	5.3797E−006	−3.5E−006
2355	−2.5E−005	−7E−006	2.2164	−2.21E−006	1.0035E−005	−6.4E−006
2356	−2.7E−005	3.5E−006	2.2164	−4.11E−006	1.4981E−005	−1.0E−005
2357	−7.5E−005	−2E−005	2.2163	−6.30E−006	2.9771E−005	−1.9E−005
2358	−7.2E−005	1.8E−005	2.2161	−1.20E−005	4.123E−005	−2.8E−005
2359	−0.000230	−9E−005	2.2158	−1.80E−005	8.902E−005	−5.9E−005
2360	−0.000180	8.4E−005	2.2153	−3.50E−005	0.00011186	−8.0E−005
2361	−0.000730	−0.00032	2.2144	−5.16E−005	0.00027006	−0.0001890
2362	−0.000402	0.0003932	2.2128	−0.0001046	0.00030191	−0.0002349
2363	−0.002468	−0.00129	2.2102	−0.0001529	0.00084654	−0.0006389
2364	−0.000557	0.0020145	2.2055	−0.0003296	0.00086209	−0.0007619
2365	−0.009690	−0.00629	2.1967	−0.0005068	0.0029059	−0.0024157
2366	0.0028793	0.012779	2.1793	−0.0012632	0.0031784	−0.0032786
2367	−0.048546	−0.03780	2.1255	−0.0015614	0.012956	−0.012957
2368	0.043625	0.080882	2.0092	−0.013804	0.051928	−0.0076766
2369	0.056051	−0.02616	2.2586	0.0026513	−0.29655	0.09443
2370	0.14775	0.12476	2.2317	−0.0005966	0.24358	−0.17378
2371	0.024142	0.031499	2.0661	−0.019639	−0.030482	0.017146
2372	−0.033561	−0.02841	2.122	−0.0064352	−0.020131	0.015209
2373	0.0024288	0.005954	2.1767	−0.002803	−0.0080758	0.0077935
2374	−0.007430	−0.00573	2.1914	−0.001504	−0.0043194	0.0043544
2375	0.00033624	0.0012625	2.2053	−0.0009681	−0.0022117	0.0020294
2376	−0.002043	−0.00147	2.2089	−0.0004905	−0.0012878	0.0012679
2377	−7.1E−006	0.0002808	2.2129	−0.0003335	−0.00070694	0.0006382
2378	−0.000611	−0.00041	2.2141	−0.0001699	−0.00042664	0.00040537
2379	−3.9E−005	5.7E−005	2.2153	−0.0001153	−0.00023973	0.00021449
2380	−0.000189	−0.00012	2.2157	−5.96E−005	−0.00014592	0.00013508
2381	−2.4E−005	9.1E−006	2.2161	−3.98E−005	−8.271E−005	7.369E−005
2382	−6.0E−005	−4E−005	2.2162	−2.09E−005	−5.037E−005	4.583E−005
2383	−1.1E−005	2.5E−007	2.2164	−1.38E−005	−2.867E−005	2.55E−005
2384	−1.9E−005	−1E−005	2.2164	−7.32E−006	−1.743E−005	1.569E−005
2385	−4.6E−006	−7E−007	2.2165	−4.75E−006	−9.948E−006	8.846E−006
2386	−6.2E−006	−3E−006	2.2165	−2.56E−006	−6.037E−006	5.396E−006
2387	−1.8E−006	−5E−007	2.2165	−1.64E−006	−3.453E−006	3.070E−006
2388	−2.1E−006	−1E−006	2.2165	−8.95E−007	−2.092E−006	1.862E−006
2389	−6.9E−007	−2E−007	2.2165	−5.66E−007	−1.198E−006	1.066E−006
2390	−6.8E−007	−3E−007	2.2165	−3.12E−007	−7.250E−007	6.436E−007
2391	−2.6E−007	−9E−008	2.2165	−1.96E−007	−4.159E−007	3.698E−007
2392	−2.3E−007	−1E−007	2.2165	−1.09E−007	−2.513E−007	2.228E−007
2393	−9.3E−008	−4E−008	2.2165	−6.76E−008	−1.443E−007	1.283E−007
2394	−7.7E−008	−4E−008	2.2165	−3.79E−008	−8.716E−008	7.719E−008

2395	−3.3E−008	−1E−008	2.2165	−2.34E−008	−5.005E−008	4.449E−008
2396	−2.6E−008	−1E−008	2.2165	−1.32E−008	−3.023E−008	2.677E−008
2397	−1.2E−008	−5E−009	2.2165	−8.10E−009	−1.736E−008	1.542E−008
2398	−8.9E−009	−4E−009	2.2165	−4.59E−009	−1.049E−008	9.286E−009
2399	−4.2E−009	−2E−009	2.2165	−2.81E−009	−6.019E−009	5.346E−009
2400	−3.0E−009	−1E−009	2.2165	−1.60E−009	−3.641E−009	3.223E−009
2401	−1.5E−009	−7E−010	2.2165	−9.72E−010	−2.087E−009	1.853E−009
2402	−1.0E−009	−4E−010	2.2165	−5.54E−010	−1.264E−009	1.119E−009
2403	−5.2E−010	−3E−010	2.2165	−3.37E−010	−7.232E−010	6.418E−010
2404	−3.6E−010	−1E−010	2.2165	−1.93E−010	−4.391E−010	3.887E−010
2405	−1.8E−010	−9E−011	2.2165	−1.17E−010	−2.507E−010	2.224E−010
2406	−1.2E−010	−5E−011	2.2165	−6.68E−011	−1.526E−010	1.350E−010
2407	−6.4E−011	−3E−011	2.2165	−4.05E−011	−8.686E−011	7.692E−011
2408	−4.3E−011	−1E−011	2.2165	−2.32E−011	−5.302E−011	4.688E−011
2409	−2.2E−011	−1E−011	2.2165	−1.41E−011	−3.001E−011	2.667E−011
2410	−1.5E−011	−5E−012	2.2165	−8.03E−012	−1.828E−011	1.633E−011
2411	−7.8E−012	−4E−012	2.2165	−4.93E−012	−1.028E−011	9.219E−012
2412	−5.0E−012	−2E−012	2.2165	−2.78E−012	−6.361E−012	5.861E−012
2413	−2.8E−012	−2E−012	2.2165	−1.68E−012	−3.626E−012	3.233E−012
2414	−1.6E−012	−4E−013	2.2165	−9.49E−013	−2.155E−012	2.073E−012
2415	−1.1E−012	−7E−013	2.2165	−5.55E−013	−1.269E−012	1.018E−012
2416	−7.7E−013	−3E−013	2.2165	−3.25E−013	−6.747E−013	6.269E−013
2417	−1.5E−013	−4E−014	2.2165	−1.73E−013	−6.831E−013	4.949E−013
2418	−3.3E−013	−1E−013	2.2165	−9.61E−014	−2.224E−013	3.462E−013
2419	−4.7E−013	−5E−013	2.2165	−2.56E−014	−4.019E−014	2.602E−013
2420	−1.3E−013	−5E−014	2.2165	−6.67E−014	−2.357E−013	−8.6E−014
2421	−1.6E−013	−1E−013	2.2165	2.912E−015	−2.254E−013	8.303E−014
2422	1.73E−013	1.2E−013	2.2165	5.194E−014	−1.340E−013	−7.4E−014
2423	−7.4E−014	−2E−013	2.2165	−1.62E−014	7.2789E−014	−1.7E−013
2424	1.28E−013	1.7E−013	2.2165	−3.75E−014	6.337E−014	1.977E−013
2425	4.25E−014	2.7E−014	2.2165	2.265E−014	−5.218E−014	1.497E−013
2426	−1.2E−013	−2E−014	2.2165	−5.87E−015	7.5606E−014	4.462E−016
2427	1.05E−013	1.0E−013	2.2165	−1.61E−014	−2.228E−013	−1.8E−013
2428	−1.4E−013	−3E−013	2.2165	5.353E−014	5.4027E−015	−1.9E−014
2429	2.10E−013	1.8E−013	2.2165	7.564E−014	9.8007E−014	1.205E−014
2430	−4.3E−014	−5E−014	2.2165	2.729E−014	8.0197E−014	6.887E−014
2431	−2.8E−013	−2E−013	2.2165	1.016E−013	1.2959E−013	−2.2E−013
2432	1.04E−013	1.3E−013	2.2165	−3.07E−014	−3.176E−014	−1.2E−013
2433	−9.9E−014	2.3E−014	2.2165	2.288E−014	−2.108E−014	−4.9E−014
2434	−1.7E−014	−1E−013	2.2165	2.661E−014	−3.534E−013	−1.8E−013
2435	1.42E−013	8.1E−014	2.2165	−3.39E−014	2.8517E−013	−1.9E−013
2436	−7.7E−014	−1E−013	2.2165	−1.27E−013	−2.234E−013	2.285E−013
2437	2.54E−013	2.2E−013	2.2165	3.308E−014	−3.240E−013	−1.6E−014
2438	−3.1E−014	−2E−013	2.2165	−9.71E−015	−2.324E−013	1.751E−013
2439	−2.4E−013	−1E−013	2.2165	8.693E−014	−2.036E−014	1.731E−014
2440	−2.1E−013	−2E−013	2.2165	−1.51E−015	1.4903E−013	2.297E−013
2441	−1.4E−013	−1E−013	2.2165	6.426E−014	3.4414E−014	−9.7E−014

2442	1.84E−013	1.1E−013	2.2165	3.671E−014	1.1512E−013	1.847E−013
2443	1.37E−013	−7E−014	2.2165	2.278E−014	7.095E−014	1.603E−013
2444	3.19E−013	5.9E−013	2.2165	2.294E−014	4.0125E−014	5.780E−014
2445	−3.7E−013	−5E−013	2.2165	3.448E−014	2.477E−013	7.931E−014
2446	−2.5E−013	−2E−013	2.2165	7.736E−014	1.8696E−013	−9.8E−015
2447	−9.2E−014	1.7E−013	2.2165	3.774E−014	9.7657E−014	1.100E−013
2448	−4.2E−013	−3E−013	2.2165	−3.57E−014	1.1291E−013	1.206E−013
2449	−4.6E−013	−4E−013	2.2165	7.753E−014	−1.085E−013	−4.5E−013
2450	1.10E−013	1.9E−014	2.2165	−7.16E−015	2.406E−013	−6.5E−014
2451	−2.2E−013	−3E−013	2.2165	−1.14E−014	−2.261E−013	−1.9E−013
2452	2.98E−014	1.5E−013	2.2165	−5.49E−014	−1.207E−014	2.431E−013
2453	−2.5E−013	−3E−013	2.2165	−4.90E−014	1.266E−013	9.157E−014
2454	−3.5E−013	−2E−013	2.2165	5.655E−014	1.6126E−013	3.524E−013
2455	1.44E−013	1.7E−013	2.2165	−5.48E−014	−3.979E−013	1.883E−014
2456	8.88E−016	1.2E−013	2.2165	1.730E−014	2.9934E−013	−3.7E−013
2457	−1.3E−013	2.6E−014	2.2165	−3.30E−015	−1.598E−014	−3.0E−013
2458	1.65E−013	1.0E−013	2.2165	−1.14E−013	2.8283E−014	−2.6E−013
2459	−5.0E−013	−6E−013	2.2165	−1.71E−013	5.2905E−013	−3.8E−013
2460	−5.7E−013	−6E−014	2.2165	−3.26E−013	7.8085E−013	−3.0E−013
2461	−1.0E−012	−7E−013	2.2165	−5.15E−013	9.0049E−013	−1.2E−012
2462	−2.1E−012	−8E−013	2.2165	−8.76E−013	2.6401E−012	−1.8E−012
2463	−2.7E−012	−2E−012	2.2165	−1.76E−012	3.5675E−012	−3.2E−012
2464	−4.8E−012	−1E−012	2.2165	−2.79E−012	6.1348E−012	−5.9E−012
2465	−7.8E−012	−4E−012	2.2165	−4.81E−012	1.0508E−011	−8.8E−012
2466	−1.5E−011	−5E−012	2.2165	−8.02E−012	1.8342E−011	−1.6E−011
2467	−2.2E−011	−1E−011	2.2165	−1.39E−011	3.0238E−011	−2.6E−011
2468	−4.3E−011	−2E−011	2.2165	−2.32E−011	5.2842E−011	−4.7E−011
2469	−6.4E−011	−3E−011	2.2165	−4.06E−011	8.6525E−011	−7.7E−011
2470	−1.2E−010	−5E−011	2.2165	−6.68E−011	1.5247E−010	−1.4E−010
2471	−1.8E−010	−9E−011	2.2165	−1.17E−010	2.5052E−010	−2.2E−010
2472	−3.6E−010	−1E−010	2.2165	−1.93E−010	4.3884E−010	−3.9E−010
2473	−5.2E−010	−3E−010	2.2165	−3.37E−010	7.2312E−010	−6.4E−010
2474	−1.0E−009	−4E−010	2.2165	−5.54E−010	1.2636E−009	−1.1E−009
2475	−1.5E−009	−7E−010	2.2165	−9.72E−010	2.0868E−009	−1.9E−009
2476	−3.0E−009	−1E−009	2.2165	−1.60E−009	3.6418E−009	−3.2E−009
2477	−4.2E−009	−2E−009	2.2165	−2.81E−009	6.0189E−009	−5.3E−009
2478	−8.9E−009	−4E−009	2.2165	−4.59E−009	1.0491E−008	−9.3E−009
2479	−1.2E−008	−5E−009	2.2165	−8.10E−009	1.7358E−008	−1.5E−008
2480	−2.6E−008	−1E−008	2.2165	−1.32E−008	3.0235E−008	−2.7E−008
2481	−3.3E−008	−1E−008	2.2165	−2.34E−008	5.0048E−008	−4.4E−008
2482	−7.7E−008	−4E−008	2.2165	−3.79E−008	8.7161E−008	−7.7E−008
2483	−9.3E−008	−4E−008	2.2165	−6.76E−008	1.4428E−007	−1.3E−007
2484	−2.3E−007	−1E−007	2.2165	−1.09E−007	2.5134E−007	−2.2E−007
2485	−2.6E−007	−9E−008	2.2165	−1.96E−007	4.1585E−007	−3.7E−007
2486	−6.8E−007	−3E−007	2.2165	−3.12E−007	7.2498E−007	−6.4E−007
2487	−6.9E−007	−2E−007	2.2165	−5.66E−007	1.1984E−006	−1.1E−006
2488	−2.1E−006	−1E−006	2.2165	−8.95E−007	2.0918E−006	−1.9E−006

2489	−1.8E−006	−5E−007	2.2165	−1.64E−006	3.453E−006	−3.1E−006
2490	−6.2E−006	−3E−006	2.2165	−2.56E−006	6.0371E−006	−5.4E−006
2491	−4.6E−006	−7E−007	2.2165	−4.75E−006	9.9483E−006	−8.8E−006
2492	−1.9E−005	−1E−005	2.2164	−7.32E−006	1.7431E−005	−1.6E−005
2493	−1.1E−005	2.5E−007	2.2164	−1.38E−005	2.8667E−005	−2.6E−005
2494	−6.0E−005	−4E−005	2.2162	−2.09E−005	5.0368E−005	−4.6E−005
2495	−2.4E−005	9.1E−006	2.2161	−3.98E−005	8.2705E−005	−7.4E−005
2496	−0.000189	−0.00012	2.2157	−5.96E−005	0.00014592	−0.0001351
2497	−3.9E−005	5.7E−005	2.2153	−0.0001153	0.00023973	−0.0002145
2498	−0.000611	−0.00041	2.2141	−0.0001699	0.00042664	−0.0004054
2499	−7.1E−006	0.0002808	2.2129	−0.0003335	0.00070694	−0.0006382
2500	−0.002043	−0.00147	2.2089	−0.0004905	0.0012878	−0.0012679
2501	0.00033624	0.0012625	2.2053	−0.0009681	0.0022117	−0.0020294
2502	−0.007430	−0.00573	2.1914	−0.001504	0.0043194	−0.0043544
2503	0.0024288	0.005954	2.1767	−0.002803	0.0080758	−0.0077935
2504	−0.033561	−0.02841	2.122	−0.0064352	0.020131	−0.015209
2505	0.024142	0.031499	2.0661	−0.019639	0.030482	−0.017146
2506	0.14775	0.12476	2.2317	−0.0005966	−0.24358	0.17378
2507	0.12476	0.14775	2.2317	−0.0005966	0.17378	−0.24358
2508	0.031499	0.024142	2.0661	−0.019639	−0.017146	0.030482
2509	−0.028413	−0.03356	2.122	−0.0064352	−0.015209	0.020131
2510	0.005954	0.0024288	2.1767	−0.002803	−0.0077935	0.0080758
2511	−0.005728	−0.00743	2.1914	−0.001504	−0.0043544	0.0043194
2512	0.0012625	0.0003362	2.2053	−0.0009681	−0.0020294	0.0022117
2513	−0.001467	−0.00204	2.2089	−0.0004905	−0.0012679	0.0012878
2514	0.00028079	−7E−006	2.2129	−0.0003335	−0.0006382	0.00070694
2515	−0.000413	−0.00061	2.2141	−0.0001699	−0.00040537	0.00042664
2516	5.74E−005	−4E−005	2.2153	−0.0001153	−0.00021449	0.00023973
2517	−0.000121	−0.00019	2.2157	−5.96E−005	−0.00013508	0.00014592
2518	9.13E−006	−2E−005	2.2161	−3.98E−005	−7.369E−005	8.271E−005
2519	−3.6E−005	−6E−005	2.2162	−2.09E−005	−4.583E−005	5.037E−005
2520	2.47E−007	−1E−005	2.2164	−1.38E−005	−2.55E−005	2.867E−005
2521	−1.1E−005	−2E−005	2.2164	−7.32E−006	−1.569E−005	1.743E−005
2522	−7.1E−007	−5E−006	2.2165	−4.75E−006	−8.846E−006	9.948E−006
2523	−3.4E−006	−6E−006	2.2165	−2.56E−006	−5.396E−006	6.037E−006
2524	−4.6E−007	−2E−006	2.2165	−1.64E−006	−3.070E−006	3.453E−006
2525	−1.1E−006	−2E−006	2.2165	−8.95E−007	−1.862E−006	2.092E−006
2526	−2.2E−007	−7E−007	2.2165	−5.66E−007	−1.066E−006	1.198E−006
2527	−3.4E−007	−7E−007	2.2165	−3.12E−007	−6.436E−007	7.250E−007
2528	−9.3E−008	−3E−007	2.2165	−1.96E−007	−3.698E−007	4.159E−007
2529	−1.1E−007	−2E−007	2.2165	−1.09E−007	−2.228E−007	2.513E−007
2530	−3.7E−008	−9E−008	2.2165	−6.76E−008	−1.283E−007	1.443E−007
2531	−3.5E−008	−8E−008	2.2165	−3.79E−008	−7.719E−008	8.716E−008
2532	−1.4E−008	−3E−008	2.2165	−2.34E−008	−4.449E−008	5.005E−008
2533	−1.2E−008	−3E−008	2.2165	−1.32E−008	−2.677E−008	3.023E−008
2534	−5.3E−009	−1E−008	2.2165	−8.10E−009	−1.542E−008	1.736E−008
2535	−3.8E−009	−9E−009	2.2165	−4.59E−009	−9.286E−009	1.049E−008

2536	−2.0E−009	−4E−009	2.2165	−2.81E−009	−5.347E−009	6.019E−009
2537	−1.2E−009	−3E−009	2.2165	−1.60E−009	−3.223E−009	3.641E−009
2538	−7.2E−010	−1E−009	2.2165	−9.72E−010	−1.853E−009	2.087E−009
2539	−4.1E−010	−1E−009	2.2165	−5.54E−010	−1.119E−009	1.264E−009
2540	−2.6E−010	−5E−010	2.2165	−3.37E−010	−6.421E−010	7.232E−010
2541	−1.4E−010	−4E−010	2.2165	−1.93E−010	−3.888E−010	4.391E−010
2542	−9.4E−011	−2E−010	2.2165	−1.17E−010	−2.223E−010	2.505E−010
2543	−4.5E−011	−1E−010	2.2165	−6.68E−011	−1.350E−010	1.526E−010
2544	−3.4E−011	−6E−011	2.2165	−4.05E−011	−7.699E−011	8.665E−011
2545	−1.5E−011	−4E−011	2.2165	−2.31E−011	−4.7E−011	5.299E−011
2546	−1.2E−011	−2E−011	2.2165	−1.41E−011	−2.663E−011	2.993E−011
2547	−5.1E−012	−1E−011	2.2165	−8.06E−012	−1.608E−011	1.855E−011
2548	−4.2E−012	−8E−012	2.2165	−4.84E−012	−9.178E−012	1.049E−011
2549	−1.7E−012	−5E−012	2.2165	−2.77E−012	−5.568E−012	6.433E−012
2550	−1.8E−012	−3E−012	2.2165	−1.70E−012	−3.093E−012	3.789E−012
2551	−6.8E−013	−2E−012	2.2165	−1.01E−012	−1.960E−012	2.312E−012
2552	−8E−013	−1E−012	2.2165	−5.58E−013	−9.261E−013	1.273E−012
2553	−3.0E−013	−7E−013	2.2165	−3.34E−013	−5.424E−013	8.889E−013
2554	1.24E−014	1.2E−014	2.2165	−1.83E−013	−2.329E−013	4.644E−013
2555	−1.5E−013	−3E−013	2.2165	−8.03E−014	−2.468E−013	4.019E−013
2556	−2.0E−013	−1E−013	2.2165	−4.86E−014	−3.137E−013	8.79E−014
2557	3.48E−013	2.3E−013	2.2165	4.537E−014	−1.194E−013	9.172E−014
2558	−5.7E−014	1.5E−015	2.2165	−3.83E−015	−1.914E−013	−8.1E−014
2559	9.71E−014	1.0E−013	2.2165	3.180E−014	−1.463E−013	−1.2E−013
2560	−4.3E−014	−1E−013	2.2165	1.197E−014	−3.061E−015	2.551E−013
2561	−1.3E−013	−1E−013	2.2165	6.873E−014	−1.199E−013	−1.4E−013
2562	−5.5E−014	4.3E−014	2.2165	1.1E−014	−7.603E−014	−9.6E−014
2563	−6.4E−014	2.2E−015	2.2165	4.309E−014	3.2166E−014	3.574E−014
2564	−8.2E−014	−7E−015	2.2165	−7.91E−014	−7.657E−014	−1.3E−013
2565	−9.1E−014	−2E−013	2.2165	−2.19E−014	−1.014E−013	2.303E−013
2566	−1.3E−013	−2E−013	2.2165	4.533E−014	1.405E−013	−4.8E−014
2567	5.45E−014	9.5E−014	2.2165	−2.34E−014	3.3571E−013	−6.7E−014
2568	−2.6E−013	−2E−013	2.2165	−3.32E−014	8.4911E−014	8.710E−014
2569	−9.1E−014	4.4E−014	2.2165	−4.58E−014	−5.353E−014	4.003E−015
2570	6.10E−014	−1E−014	2.2165	−1.90E−014	−1.048E−013	−9.6E−014
2571	−2.8E−013	−3E−013	2.2165	−9.98E−015	−2.467E−013	2.917E−014
2572	−3.0E−013	−4E−013	2.2165	3.078E−014	8.0782E−014	9.541E−014
2573	−3.8E−013	−3E−013	2.2165	2.104E−014	2.4716E−014	−3.1E−013
2574	3.36E−015	−6E−014	2.2165	7.018E−015	1.5329E−015	−1.5E−014
2575	−2.7E−013	−2E−013	2.2165	4.094E−014	6.6086E−014	1.971E−013
2576	5.21E−014	−5E−014	2.2165	1.003E−014	1.419E−013	−2.5E−013
2577	1.39E−014	2.4E−013	2.2165	−2.58E−014	9.2573E−014	3.436E−014
2578	−1.2E−013	−9E−014	2.2165	4.455E−014	−7.094E−014	2.767E−013
2579	4.19E−014	1.2E−013	2.2165	−3.77E−014	−6.401E−014	−1.6E−013
2580	−1.6E−013	−3E−013	2.2165	7.405E−015	1.484E−014	1.651E−013
2581	1.32E−014	3.5E−015	2.2165	−3.58E−014	−6.306E−014	1.182E−013
2582	−1.4E−013	−4E−014	2.2165	3.347E−015	2.3929E−013	1.056E−013

2583	−1.6E−014	−7E−014	2.2165	−3.28E−014	9.8536E−014	−4.8E−014
2584	3.62E−013	4.7E−013	2.2165	1.35E−014	2.6328E−013	2.633E−014
2585	−2.0E−013	8.0E−014	2.2165	−1.79E−013	−2.206E−013	9.955E−014
2586	6.12E−014	−6E−015	2.2165	8.770E−014	−4.134E−013	−4.4E−013
2587	−6.8E−014	−1E−013	2.2165	9.405E−014	2.0691E−014	−2.5E−013
2588	−2.4E−013	−5E−013	2.2165	−1.64E−013	−7.548E−014	−1.3E−013
2589	−5.2E−014	−1E−013	2.2165	−1.16E−014	2.6007E−013	1.444E−013
2590	4.02E−014	−3E−014	2.2165	−8.82E−014	2.1344E−013	3.522E−013
2591	1.22E−013	7.5E−014	2.2165	1.41E−015	4.1171E−013	1.290E−013
2592	−1.0E−013	2.3E−013	2.2165	−6.98E−014	−3.103E−014	−2.9E−013
2593	−8.4E−014	−6E−014	2.2165	−1.25E−013	−1.441E−013	−1.7E−013
2594	−2.5E−014	−4E−015	2.2165	−1.79E−014	−3.739E−013	−4.2E−013
2595	−1.6E−013	−3E−013	2.2165	−1.58E−013	2.368E−013	−4.2E−013
2596	−1.8E−013	−4E−013	2.2165	−2.01E−013	4.7981E−014	−4.7E−013
2597	−1.6E−013	−4E−013	2.2165	−3.01E−013	6.488E−013	−9.2E−013
2598	−2.1E−013	−5E−013	2.2165	−6.86E−013	7.9877E−013	−1.3E−012
2599	−4.1E−013	−2E−012	2.2165	−8.37E−013	1.7166E−012	−1.9E−012
2600	−2.2E−012	−3E−012	2.2165	−1.62E−012	3.397E−012	−3.8E−012
2601	−1.3E−012	−5E−012	2.2165	−2.73E−012	5.5953E−012	−6.4E−012
2602	−4.1E−012	−7E−012	2.2165	−4.90E−012	9.7889E−012	−1.0E−011
2603	−5.3E−012	−1E−011	2.2165	−8.13E−012	1.6268E−011	−1.9E−011
2604	−1.2E−011	−2E−011	2.2165	−1.39E−011	2.7054E−011	−3.0E−011
2605	−1.5E−011	−4E−011	2.2165	−2.31E−011	4.7359E−011	−5.3E−011
2606	−3.4E−011	−6E−011	2.2165	−4.06E−011	7.7076E−011	−8.7E−011
2607	−4.5E−011	−1E−010	2.2165	−6.68E−011	1.3488E−010	−1.5E−010
2608	−9.4E−011	−2E−010	2.2165	−1.17E−010	2.2202E−010	−2.5E−010
2609	−1.4E−010	−4E−010	2.2165	−1.93E−010	3.8834E−010	−4.4E−010
2610	−2.6E−010	−5E−010	2.2165	−3.37E−010	6.4185E−010	−7.2E−010
2611	−4.1E−010	−1E−009	2.2165	−5.55E−010	1.1192E−009	−1.3E−009
2612	−7.2E−010	−1E−009	2.2165	−9.72E−010	1.8533E−009	−2.1E−009
2613	−1.2E−009	−3E−009	2.2165	−1.60E−009	3.2234E−009	−3.6E−009
2614	−2.0E−009	−4E−009	2.2165	−2.81E−009	5.3466E−009	−6.0E−009
2615	−3.8E−009	−9E−009	2.2165	−4.59E−009	9.2864E−009	−1.0E−008
2616	−5.3E−009	−1E−008	2.2165	−8.10E−009	1.5424E−008	−1.7E−008
2617	−1.2E−008	−3E−008	2.2165	−1.32E−008	2.6767E−008	−3.0E−008
2618	−1.4E−008	−3E−008	2.2165	−2.34E−008	4.4488E−008	−5.0E−008
2619	−3.5E−008	−8E−008	2.2165	−3.79E−008	7.7195E−008	−8.7E−008
2620	−3.7E−008	−9E−008	2.2165	−6.76E−008	1.2828E−007	−1.4E−007
2621	−1.1E−007	−2E−007	2.2165	−1.09E−007	2.2279E−007	−2.5E−007
2622	−9.3E−008	−3E−007	2.2165	−1.96E−007	3.6978E−007	−4.2E−007
2623	−3.4E−007	−7E−007	2.2165	−3.12E−007	6.4362E−007	−7.2E−007
2624	−2.2E−007	−7E−007	2.2165	−5.66E−007	1.0656E−006	−1.2E−006
2625	−1.1E−006	−2E−006	2.2165	−8.95E−007	1.8618E−006	−2.1E−006
2626	−4.6E−007	−2E−006	2.2165	−1.64E−006	3.0702E−006	−3.5E−006
2627	−3.4E−006	−6E−006	2.2165	−2.56E−006	5.3963E−006	−6.0E−006
2628	−7.1E−007	−5E−006	2.2165	−4.75E−006	8.8455E−006	−9.9E−006
2629	−1.1E−005	−2E−005	2.2164	−7.32E−006	1.5688E−005	−1.7E−005

2630	2.47E−007	−1E−005	2.2164	−1.38E−005	2.55E−005	−2.9E−005
2631	−3.6E−005	−6E−005	2.2162	−2.09E−005	4.5831E−005	−5.0E−005
2632	9.13E−006	−2E−005	2.2161	−3.98E−005	7.3687E−005	−8.3E−005
2633	−0.000121	−0.00019	2.2157	−5.96E−005	0.00013508	−0.0001459
2634	5.74E−005	−4E−005	2.2153	−0.0001153	0.00021449	−0.0002397
2635	−0.000413	−0.00061	2.2141	−0.0001699	0.00040537	−0.0004266
2636	0.00028079	−7E−006	2.2129	−0.0003335	0.0006382	−0.0007069
2637	−0.001467	−0.00204	2.2089	−0.0004905	0.0012679	−0.0012878
2638	0.0012625	0.0003362	2.2053	−0.0009681	0.0020294	−0.0022117
2639	−0.005728	−0.00743	2.1914	−0.001504	0.0043544	−0.0043194
2640	0.005954	0.0024288	2.1767	−0.002803	0.0077935	−0.0080758
2641	−0.028413	−0.03356	2.122	−0.0064352	0.015209	−0.020131
2642	0.031499	0.024142	2.0661	−0.019639	0.017146	−0.030482
2643	0.12476	0.14775	2.2317	−0.0005966	−0.17378	0.24358
2644	−0.026163	0.056051	2.2586	0.0026513	0.09443	−0.29655
2645	0.080882	0.043625	2.0092	−0.013804	−0.0076766	0.051928
2646	−0.037796	−0.04855	2.1255	−0.0015614	−0.012957	0.012956
2647	0.012779	0.0028793	2.1793	−0.0012632	−0.0032786	0.0031784
2648	−0.006291	−0.00969	2.1967	−0.0005068	−0.0024157	0.0029059
2649	0.0020145	−0.00056	2.2055	−0.0003296	−0.00076194	0.00086209
2650	−0.001295	−0.00247	2.2102	−0.0001529	−0.00063891	0.00084654
2651	0.00039323	−0.00040	2.2128	−0.0001046	−0.00023494	0.00030191
2652	−0.000321	−0.00073	2.2144	−5.16E−005	−0.00018902	0.00027006
2653	8.39E−005	−0.00018	2.2153	−3.50E−005	−8.021E−005	0.00011186
2654	−8.7E−005	−0.00023	2.2158	−1.80E−005	−5.929E−005	8.902E−005
2655	1.78E−005	−7E−005	2.2161	−1.20E−005	−2.82E−005	4.123E−005
2656	−2.5E−005	−7E−005	2.2163	−6.30E−006	−1.923E−005	2.977E−005
2657	3.46E−006	−3E−005	2.2164	−4.11E−006	−9.971E−006	1.498E−005
2658	−7.3E−006	−2E−005	2.2164	−2.21E−006	−6.367E−006	1.004E−005
2659	5.28E−007	−1E−005	2.2165	−1.42E−006	−3.522E−006	5.380E−006
2660	−2.2E−006	−8E−006	2.2165	−7.70E−007	−2.137E−006	3.4E−006
2661	1.44E−008	−4E−006	2.2165	−4.89E−007	−1.241E−006	1.916E−006
2662	−6.8E−007	−3E−006	2.2165	−2.68E−007	−7.233E−007	1.156E−006
2663	−3.7E−008	−1E−006	2.2165	−1.69E−007	−4.359E−007	6.786E−007
2664	−2.2E−007	−9E−007	2.2165	−9.34E−008	−2.463E−007	3.942E−007
2665	−2.3E−008	−5E−007	2.2165	−5.87E−008	−1.529E−007	2.394E−007
2666	−7.0E−008	−3E−007	2.2165	−3.24E−008	−8.417E−008	1.346E−007
2667	−1.1E−008	−2E−007	2.2165	−2.04E−008	−5.355E−008	8.431E−008
2668	−2.3E−008	−1E−007	2.2165	−1.13E−008	−2.883E−008	4.603E−008
2669	−4.3E−009	−6E−008	2.2165	−7.07E−009	−1.874E−008	2.964E−008
2670	−7.7E−009	−4E−008	2.2165	−3.90E−009	−9.887E−009	1.575E−008
2671	−1.6E−009	−2E−008	2.2165	−2.46E−009	−6.559E−009	1.041E−008
2672	−2.6E−009	−1E−008	2.2165	−1.35E−009	−3.392E−009	5.386E−009
2673	−5.8E−010	−7E−009	2.2165	−8.55E−010	−2.296E−009	3.659E−009
2674	−9.0E−010	−4E−009	2.2165	−4.67E−010	−1.164E−009	1.842E−009
2675	−2E−010	−3E−009	2.2165	−2.98E−010	−8.039E−010	1.286E−009
2676	−3.1E−010	−2E−009	2.2165	−1.61E−010	−3.989E−010	6.292E−010

2677	−6.8E−011	−9E−010	2.2165	−1.04E−010	−2.818E−010	4.520E−010
2678	−1.1E−010	−5E−010	2.2165	−5.57E−011	−1.365E−010	2.146E−010
2679	−2.2E−011	−3E−010	2.2165	−3.62E−011	−9.867E−011	1.593E−010
2680	−3.9E−011	−2E−010	2.2165	−1.92E−011	−4.671E−011	7.315E−011
2681	−7.1E−012	−1E−010	2.2165	−1.26E−011	−3.478E−011	5.581E−011
2682	−1.4E−011	−6E−011	2.2165	−6.61E−012	−1.603E−011	2.486E−011
2683	−2.1E−012	−4E−011	2.2165	−4.45E−012	−1.214E−011	1.968E−011
2684	−4.8E−012	−2E−011	2.2165	−2.28E−012	−5.636E−012	8.661E−012
2685	−7.1E−013	−1E−011	2.2165	−1.50E−012	−4.357E−012	7.004E−012
2686	−1.8E−012	−7E−012	2.2165	−7.53E−013	−1.736E−012	2.782E−012
