# Supplementary figures and images for: A unified approach to model peripheral nerves across different animal species
Source: PeerJ. 2017 Nov 10;5:e4005. doi: 10.7717/peerj.4005 (PMC5683050; doi:10.7717/peerj.4005)

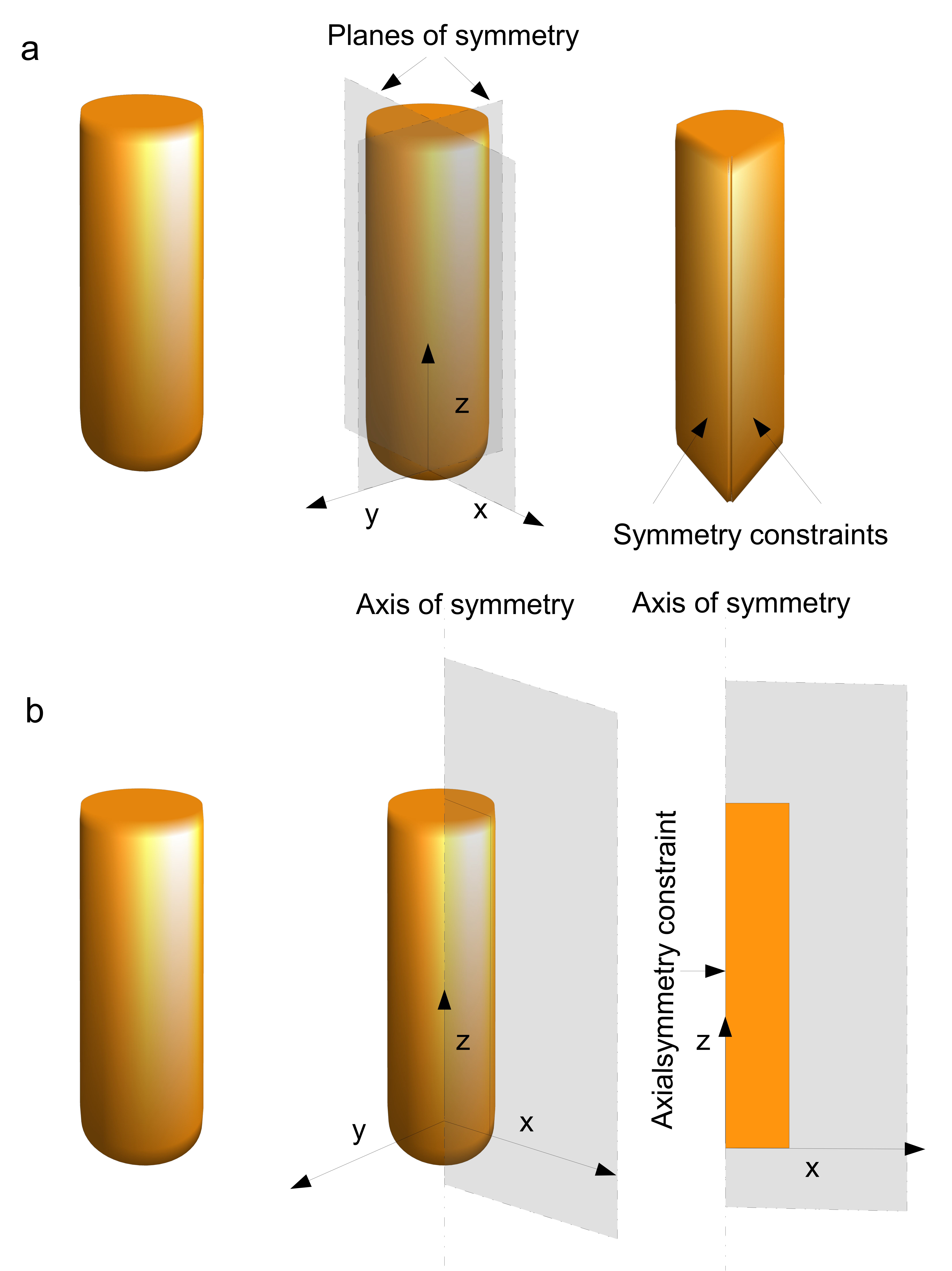

Supplement: Figure S4 — This cylinder is axisymmetric, thus a single bidimensional slice is representative for the global behaviour of the solid. [file peerj-05-4005-s005.png]

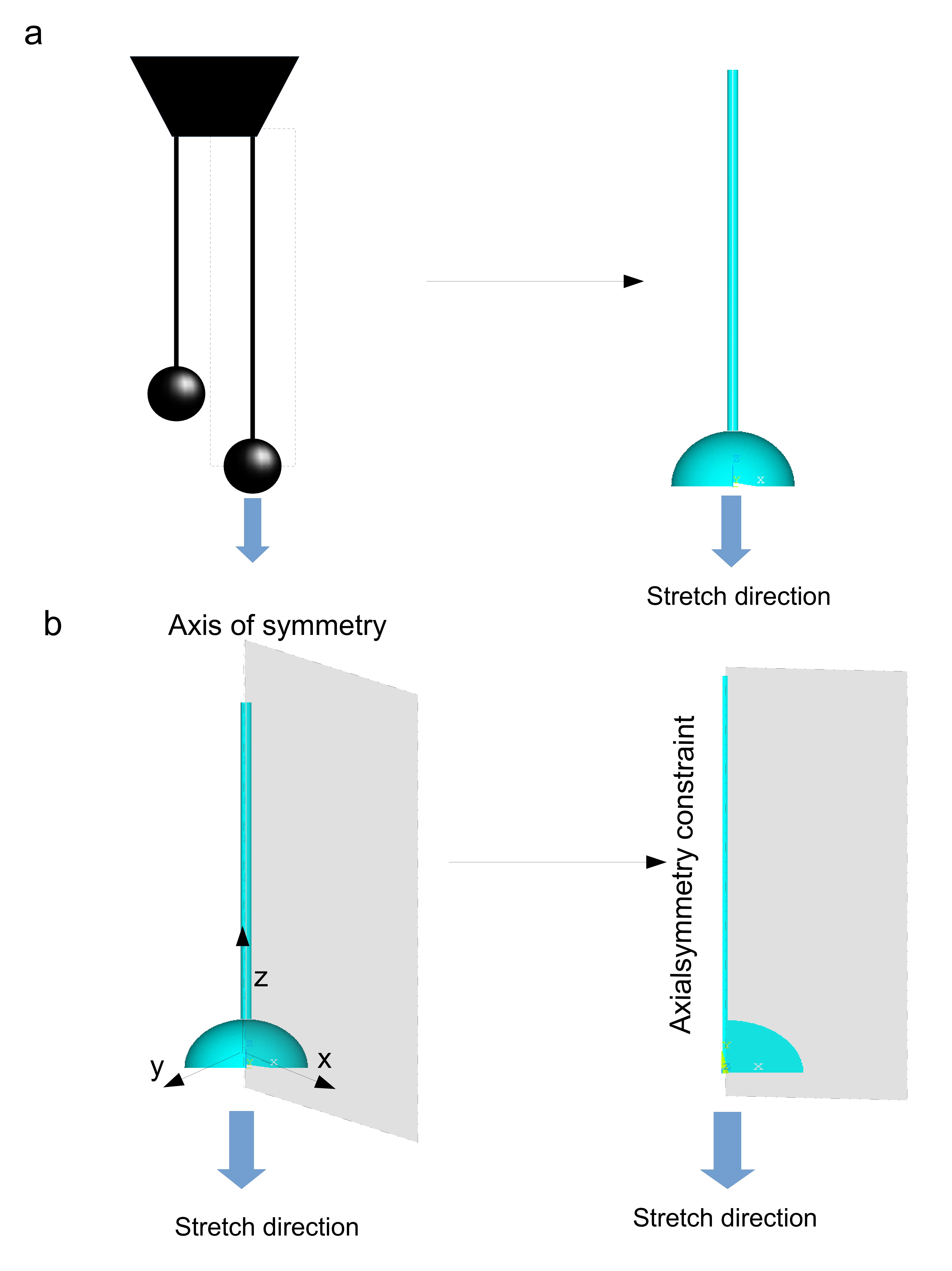

Supplement: Figure S5 [file peerj-05-4005-s006.png]
